# Novel Mitochondria-Targeted Amphiphilic Aminophosphonium Salts and Lipids Nanoparticles: Synthesis, Antitumor Activity and Toxicity

**DOI:** 10.3390/nano13212840

**Published:** 2023-10-26

**Authors:** Vladimir F. Mironov, Mudaris N. Dimukhametov, Andrey V. Nemtarev, Tatiana N. Pashirova, Olga V. Tsepaeva, Alexandra D. Voloshina, Alexandra B. Vyshtakalyuk, Igor A. Litvinov, Anna P. Lyubina, Anastasiia S. Sapunova, Dinara F. Abramova, Vladimir V. Zobov

**Affiliations:** 1 Arbuzov Institute of Organic and Physical Chemistry, FRC Kazan Scientific Center of RAS, 8 Arbuzov St., 420088 Kazan, Russia; mudaris@iopc.ru (M.N.D.); a.nemtarev@mail.ru (A.V.N.); tatyana_pashirova@mail.ru (T.N.P.); tsepaeva@iopc.ru (O.V.T.); sobaka-1968@mail.ru (A.D.V.); alex.vysh@mail.ru (A.B.V.); litvinov@iopc.ru (I.A.L.); aplyubina@gmail.com (A.P.L.); anastasiastrobykina@yandex.ru (A.S.S.); dinara.gumarowa@yandex.ru (D.F.A.); zobov@iopc.ru (V.V.Z.); 2 Alexander Butlerov Institute of Chemistry, Kazan (Volga Region) Federal University, 18 Kremlevskaya St., 420008 Kazan, Russia

**Keywords:** aminophosphonium salt, liposome, solid lipid nanoparticle, anticancer activity, apoptosis, antimicrobial activity, hemolytic activity, acute toxicity

## Abstract

The creation of mitochondria-targeted vector systems is a new tool for the treatment of socially significant diseases. Phosphonium groups provide targeted delivery of drugs through biological barriers to organelles. For this purpose, a new class of alkyl(diethylAmino)(Phenyl) Phosphonium halides (APPs) containing one, two, or three diethylamino groups was obtained by the reaction of alkyl iodides (bromides) with (diethylamino)(phenyl)phosphines under mild conditions (20 °C) and high yields (93–98%). The structure of APP was established by NMR and XRD. A high in vitro cytotoxicity of APPs against M-HeLa, HuTu 80, PC3, DU-145, PANC-1, and MCF-7 lines was found. The selectivity index is in the range of 0.06–4.0 μM (SI 17-277) for the most active APPs. The effect of APPs on cancer cells is characterized by hyperproduction of ROS and depolarization of the mitochondrial membrane. APPs induce apoptosis, proceeding along the mitochondrial pathway. Incorporation of APPs into lipid systems (liposomes and solid lipid nanoparticles) improves cytotoxicity toward tumor cells and decrease toxicity against normal cell lines. The IC_50_s of lipid systems are lower than for the reference drug DOX, with a high SI (30–56) toward MCF-7 and DU-145. APPs exhibit high selective activity against Gram-positive bacteria *S. aureus 209P* and *B. segeus 8035*, including methicillin-resistant *S. aureus* (*MRSA-1, MRSA-2*), comparable to the activity of the fluoroquinolone antibiotic norfloxacin. A moderate in vivo toxicity in CD-1 mice was established for the lead APP.

## 1. Introduction

To date, cardiovascular, neurodegenerative, and tumor diseases, as well as chronic inflammation, occupy leading positions as causes of death for people around the world. The occurrence of these pathologies and their progression are largely associated with mitochondrial dysfunctions [1,2,3,4,5,6,7,8,9]. Mitochondria, being one of the most important organelles of eukaryotic cells, are involved in the transformation of energy-rich molecules, such as carbohydrates, lipids, and amino acids, into a macroergic ATP molecule through oxidative phosphorylation (OXPHOS) [10]. They play a key role in many metabolic processes, such as the tricarboxylic acid cycle, oxidative decarboxylation of pyruvate by the pyruvate dehydrogenase complex [11], the biosynthesis of iron-sulfur enzymes [12], and steroids [13]. Reactive oxygen (ROS) and nitrogen (RNS) species arising during oxidative phosphorylation play a major role in redox cell signaling under hypoxia, cell differentiation, and innate immunity [14,15]. Mitochondria participate in the ornithine cycle (urea cycle) [16] and are responsible for calcium homeostasis [17]. The mitochondrial permeability transition (mPT) is a sensor of stress and damage, and mitochondria play a central role in cell death—apoptosis [18,19], ferroptosis [20,21,22], and necrosis [23,24]. The key role of mitochondria is the regulation of cell life and death. Taking this into account, a mitochondria-based approach for the treatment of metabolic and degenerative diseases has emerged in recent decades. This involves the use of molecules containing mitochondria-specific compounds capable of selectively accumulating in mitochondria and is being intensively studied [25]. For this purpose, delocalized lipophilic triphenylphosphonium (TPP) cations used to deliver a wide range of drugs, diagnostic cargos, and analytical probes into mitochondria are particularly effective [26,27,28]. The penetration of such cations through the hydrophobic layer of lipid membranes is often the limiting stage of their accumulation in cells and mitochondria. In tumor cells characterized by hyperpolarized (in comparison with normal cells) mitochondrial membrane (Ψ_IM_~−220 mV) [29], in the presence of sufficient oxygen, stable aerobic glycolysis (Warburg effect) is observed [30,31]. The difference in transmembrane mitochondrial potentials in tumor and normal cells (ΔΨ about 60 mV) leads to multiple (up to 1 × 10^3^ times) accumulations of lipophilic cations in tumor cells, thereby conferring the property of selectivity [32]. It is also known that some lipophilic cations cause a strong decoupling of respiration and oxidative phosphorylation, depolarizing the mitochondria of tumor cells and thereby initiating apoptosis [33].

In TPP-conjugates, the combination of a therapeutic or diagnostic load with a phosphonic group is carried out, as a rule, by means of a covalent bond through a linker, the nature of which is given great attention. Thus, data on the influence of the linker nature were presented in many works [34,35,36,37,38,39,40,41]. There is a report on the effect of the R substituent in R–P^+^Ph_3_ salts on their biological properties [42]. At the same time, there are a few publications in which the nature of substituents in aryl fragments of lipophilic R–P^+^Ar_3_ cations themselves has been demonstrated to have a great influence on their biological, including cytostatic properties [43,44,45,46]. Thus, structural modification of the phenyl rings of TPP^+^ by reducing the electron density on the phosphorus atom can lead to the disappearance of uncoupling activity compared with the original TPP^+^ fragment [43]. However, a change in the structure of the aryl substituent in the Ar_3_P^+^–R cation does not have a negative effect on the delivery of cargo to the mitochondria [45].

Numerous studies were conducted to elucidate the role of charge delocalization of lipophilic cations in the efficiency of their penetration through lipid membranes. Thus, cyclohexyl and aryl moieties are not necessary for effective penetration [47,48]. The presence of halide or methyl substituents on the phenyl ring of the triphenylphosphonium cation can accelerate the penetration of such hydrophobic cations through lipid membranes. Thus, their effect is enhanced in biological systems where penetration is the limiting step. An increase in hydrophobicity of some analogues correlates with an increase in the penetration rate constant, but there is no full agreement for this [49].

The creation of nanotherapeutic drugs aimed at certain cellular organelles is based on the advantages of nanomedicine [50]: (i) molecular therapy; (ii) an increased ratio of nanoparticle surface area to their volume that allows to increase efficiency and safety of administration at lower doses; (iii) a possibility of functionalization of NPS surface to avoid an immune response; (iv) effective accumulation of NPS through the vascular network. These advantages create prospects for future cancer therapy [51,52,53,54]. Organelle-directed delivery systems will be able to solve major problems as a high toxicity of antitumor drugs and multiple drug resistance. In addition, colloidal carriers are able to improve the solubility of medicinal substances, prevent degradation, prolong their action, and, i.e., generally increase their effectiveness. Natural and biodegradable nanomaterials are the most promising for clinical use [55,56,57]. The successful application of lipid nanoparticles for cancer therapy has been reported [58,59,60,61,62]. From a wide range of mitochondrial-directed NPS, the attention of our group is attracted by the lipid nanosystems functionalized with triphenylphosphonium fragments [63,64].

Ar_3_P^+^–R salts, despite their high efficiency in targeted delivery to tumor cells, often show high toxicity in vitro with respect to normal cells and do not undergo biodegradation. It can become a problem for elimination from the body. In this regard, the search for effective phosphorus-containing vector systems is currently intensively underway. Moreover, the question remains open about the effect of the positive charge delocalization degree in triaryl-phosphonium cations on its ability to penetrate mitochondrial membranes and on subsequent biological properties. Heteroatoms more effectively stabilize the positive charge on carbenium ions by donating a lone pair of electrons (LPE) to the corresponding vacant orbital of electron-deficient carbon [65].

**Scheme 1:**
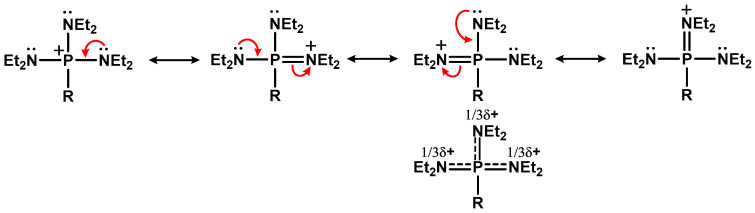
Delocalization of positive charge in aminophosphonium cations.

It was of great interest to find out how the introduction of the heteroatoms with LPE to the phosphorus atom would affect the transport and biological properties of the phosphonium cation. That should more effectively extinguish a positive charge on the phosphorus atom than aryl groups with substituents of any nature. Phosphonium salts containing at least one heteroatomic substituent with LPE are classified as quasi-phosphonium compounds with very high reactivity [66]. Among them, quasi-phosphonium salts with dialkylamine substituents have the greatest stability. It is important to note that dialkylamine substituents are more hydrophilic, and the corresponding amino-quasi-phosphonium salts with a methyl group at the phosphorus atom are even soluble in water [67]. Furthermore, the amine substituent contributes to the effective delocalization of the positive charge in phosphonium salts due to the *p*_π_–*d*_π_-conjugation of the nitrogen LPE with the *d*-orbitals of the phosphorus atom [68,69,70] (Scheme 1).

In the present work, we synthesized various amphiphilic salts of phosphonium bearing a diethylamine substituent, evaluated their cytotoxicity in vitro and toxicity in vivo, and developed nanotherapeutic forms for their delivery.

## 2. Materials and Methods

### 2.1. General

The NMR spectra were recorded at 25 °C using a Bruker Avance-400 NMR spectrometer (Bruker, Bremen, Germany) (400.0 MHz, ^1^H; 100.6 MHz, ^13^C; 162.0 MHz, ^31^P), a Bruker Avance-500 NMR spectrometer (Bruker, Bremen, Germany) (500.0 MHz, ^1^H; 125.8 MHz, ^13^C; 202.4 MHz, ^31^P), and a Bruker Avance-600 NMR spectrometer (Bruker, Bremen, Germany) (600.0 MHz, ^1^H; 150.9 MHz, ^13^C; 242.94 MHz, ^31^P). The chemical shifts were measured on the δ scale relative to TMS, using residual protons or carbon signals of CDCl_3_ or another solvent (^1^H and ^13^C) as an internal standard or H_3_PO_4_ as an external standard. Coupling constant (*J*) values are given in Hz. The ESI MS measurements were performed using an Amazon X ion trap mass spectrometer (Bruker Daltonic GmbH, Bremen, Germany) in positive mode in the mass range of 70–3000. The capillary voltage was −3500 V, nitrogen drying gas—10 L·min^–1^, desolvation temperature—250 °C. Data processing was performed by Data Analysis 4.0 SP4 software (Bruker Daltonik GmbH, Germany). Melting points were determined on a Melting Point Apparatus Stuart SMP10. Elemental analysis was accomplished with an automated EuroVector EA3000 CHNS-O elemental analyzer (Euro-Vector, Pavia, Italy). The reactions course was monitored by the ^31^P-{^1^H} NMR spectra of the reaction mixtures. The solvents were purified and dried according to standard protocols. Starting P(III) derivatives were synthesized in accordance with literature data [71] (compound **1**, δ_P_ 119.0 ppm, MeCN), [72] (compound **2**, δ_P_ 99.4 ppm, Et_2_O), [73] (compound **3**, δ_P_ 62.9 ppm, CH_2_Cl_2_). Compound **7** was obtained in acetonitrile according to the data for triethyl(octyl)ammonium bromide [74]. The interpretation of its ^13^C NMR spectrum was made taking into account the data from the work [75]. The interpretation of the ^13^C NMR spectrum of compound **8** was made taking into account the data for octyltriphenylphosphonium bromide [40].

XRD of **6a** was performed on a Bruker D8 QUEST automatic three-circle diffractometer at 130 (graphite monochromator, λMoK_α_ = 0.71073Å, ω- and φ-scan with a step of 0.5°) at the Distributed Spectral-Analytical Center of Shared Facilities for Study of Structure, Composition, and Properties of Substances and Materials of FRC Kazan Scientific Center of RAS. Single crystals of a suitable size were glued to the top of a glass fiber in a random orientation. The preliminary unit cell parameters were determined using three runs at different φ angle positions with 12 frames per run (φ-scan technique). The X-ray diffraction data were collected and indexed, and the unit cell parameters were determined and refined using the APEX2 software package [76]. The empirical absorption correction based on the crystal shape and an additional spherical correction were applied, and systematic errors were corrected using the SADABS software [77]. The structure was solved by the direct method using the SHELXT-2014/5 program [78] and refined by the full-matrix least-squares method based on *F*^2^ using the SHELXL-2018/3 program [79] as implemented in WinGX-2020.1 [80]. Non-hydrogen atoms were refined with anisotropic displacement parameters. Hydrogen atoms at carbon atoms were positioned geometrically and refined using a riding model. Intermolecular interactions were analyzed, and the figures were generated with the PLATON [81] and Mercury 2020.3 [82] programs, respectively. Crystallographic data for the structures of **6a** were deposited with the Cambridge Crystallographic Data Centre (No. CCDC 2293515). The X-ray diffraction data collection and structure refinement statistics are given in Table 1.

### 2.2. Chemistry

#### General Procedure for the Synthesis of Alkyl Diethylaminophoshonium Derivatives **4a***–***e**, **5a***–***d**, **6a***–***e**, **7**

Equmolar amounts of the corresponding P(III)-derivatives 1, 2 or 3 (4.0 mmol) and alkyl iodide (4.0 mmol) in 5 mL of anhydrous acetonitrile were stirred at the room temperature under a dry argon atmosphere. After the disappearance of the signal of the phosphorus (III) atom in the ^31^P NMR spectra of in the reaction mixtures the solvent was evaporated under reduced pressure. The residue was washed with dry diethyl ether (5 × 5 mL) (**4a**–**d**, **5a**–**d**, **6a**–**d**) or with hexane (5 × 5 mL) (**4e**, **6e**). Furthermore, the resulting oil dried under reduced pressure (0.01 mmHg) at 40–50 °C for 1–2 h. Compound 6a crystallized upon keeping it at room temperature. Its structure was confirmed by single crystal X-ray diffraction.

Tris(diethylamino)(hexyl)phosphonium iodide **4a**, light yellow oil, yield was 1.80 g (98%). Anal. C, 47.36; H, 9.71; N, 8.92; P, 6.93%, calcd for C_18_H_43_IN_3_P, C, 47.06; H, 9.43; N, 9.15; P, 6.74%. ESI-MS, *m*/*z*: 332.29 [M–I]^+^; calcd for C_18_H_43_N_3_P^+^ 332.32. ^1^H NMR spectrum (400.0 MHz, CDCl_3_, δ ppm, *J* Hz): 3.18 dq (NCH_2_, 12H, ^3^*J*_PNCH_ 10.6, ^3^*J*_HH_ 7.1), 2.55 m (C^1^H_2_, 2H), 1.56 m (C^2^H_2_, C^3^H_2_, 4H), 1.32–1,33 m (C^4^H_2_, C^5^H_2_, 4H), 1.24 t (NCCH_3_, 18H, ^3^*J*_HH_ 7.1), 0.90 t (C^6^H_3_, 3H, ^3^*J*_HH_ 7.0). ^13^C-{^1^H} NMR spectrum (100.6 MHz, CDCl_3_, δ_C_ ppm, *J* Hz): 39.43 d (NCH_2_, ^2^*J*_PNC_ 3.8), 30.99 s (C^4^), 30.27 d (C^2^, ^2^*J*_PCC_ 18.0), 25.74 d (C^1^, ^1^*J*_PC_ 104.6), 22.21 d (C^3^, ^3^*J*_PCCC_ 4.3), 22.03 s (C^5^), 13.71 s (C^6^), 13.24 d (NCCH_3_, 3H, ^3^*J*_PNCC_ 2.8). ^31^P-{^1^H} NMR spectrum (162.0 MHz, CDCl_3_): δ_P_ 59.2 ppm.

Tris(diethylamino)(octyl)phosphonium iodide **4b**, light yellow oil, yield was 1.87 g (96%). Anal. C, 49.52; H, 9.81; N, 8.55; P, 6.73%, calcd for C_20_H_47_IN_3_P, C, 49.28; H, 9.72; N, 8.62; P, 6.35%. ESI-MS, *m*/*z*: 360.34 [M–I]^+^; calcd for C_20_H_47_N_3_P^+^ 360.35. ^1^H NMR spectrum (400.0 MHz, CDCl_3_, δ ppm, *J* Hz): 3.19 dq (NCH_2_, 12H, ^3^*J*_PNCH_ 10.5, ^3^*J*_HH_ 7.1), 2.57 m (C^1^H_2_, 2H), 1.56 m (C^2^H_2_, C^3^H_2_, 4H), 1.29 m (C^4^H_2_, C^5^H_2_, C^6^H_2_, C^7^H_2_, 8H), 1.25 t (NCCH_3_, 18H, ^3^*J*_HH_ 7.1), 0.89 t (C^8^H_3_, 3H, ^3^*J*_HH_ 7.1). ^13^C-{^1^H} NMR spectrum (100.6 MHz, CDCl_3_, δ_C_ ppm, *J* Hz): 30.70 d (NCH_2_, ^2^*J*_PNC_ 3.7), 31.64 s (C^6^), 30.87 d (C^2^, ^2^*J*_PCC_ 18.0), 29.13 d (C^4^, ^4^*J*_PCCCC_ 1.7), 28.90 s (C^5^), 26.02 d (C^1^, ^1^*J*_PC_ 104.5), 22.54 s (C^7^), 14.04 s (C^8^), 13.50 d (NCCH_3_, ^3^*J*_PNCC_ 2.9). ^31^P-{^1^H} NMR spectrum (162.0 MHz, CDCl_3_): δ_P_ 58.9 ppm.

Tris(diethylamino)(nonyl)phosphonium iodide **4c**, light yellow oil, yield was 1.94 g (97%). Anal. C, 50.08; H, 9.57; N, 8.54; P, 6.39%, calcd for C_21_H_49_IN_3_P, C, 50.30; H, 9.78; N, 8.38; P, 6.18%. ESI-MS, *m*/*z*: 374.37 [M–I]^+^; calcd for C_21_H_49_N_3_P^+^ 374.37. ^1^H NMR spectrum (400.0 MHz, CDCl_3_, δ ppm, *J* Hz): 2.93 m (NCH_2_, 12H, ^3^*J*_PNCH_ 10.6, ^3^*J*_HH_ 7.1), 2.67 m (C^1^H_2_, 2H), 1.31 m (C^2^H_2_, C^3^H_2_, 4H), 0.99–1.03 m (C^4^H_2_, C^5^H_2_, C^6^H_2_, C^7^H_2_, C^8^H_2_, 10H, NCCH_3_, 18H, ^3^*J*_HH_ 7.1), 0.63 t (C^9^H_3_, 3H, ^3^*J*_HH_ 7.0). ^13^C-{^1^H} NMR spectrum (100.6 MHz, CDCl_3_, δ_C_ ppm, *J* Hz): 39.65 d (NCH_2_, ^2^*J*_PNC_ 3.3), 31.68 s (C^7^), 30.81 d (C^2^, ^2^*J*_PCC_ 18.0), 29.13 s (C^5^, C^6^), 22.06 br. s (C^4^), 25.96 d (C^1^, ^1^*J*_PC_ 104.6), 22.71 s (C^8^), 14.04 s (C^8^), 22.48 d (C^3^, ^3^*J*_PCCC_ 4.4), 14.0 s (C^9^), 13.44 d (NCCH_3_, ^3^*J*_PNCC_ 2.4). ^31^P-{^1^H} NMR spectrum (162.0 MHz, CDCl_3_): δ_P_ 58.8 ppm.

Decyltris(diethylamino)phosphonium iodide **4d**, light yellow oil, yield was 1.96 g (95%). Anal. C, 50.93; H, 9.67; N, 7.92; P, 6.25%, calcd for C_22_H_51_IN_3_P, C, 51.25; H, 9.97; N, 8.15; P, 6.01%. ESI-MS, *m*/*z*: 388.37 [M–I]^+^; calcd for C_21_H_49_N_3_P^+^ 388.38. ^1^H NMR spectrum (400.0 MHz, CDCl_3_, δ ppm, *J* Hz): 3.16 dq (NCH_2_, 12H, ^3^*J*_PNCH_ 10.6, ^3^*J*_HH_ 7.1), 2.52 m (C^1^H_2_, 2H), 1.52 m (C^2^H_2_, C^3^H_2_, 4H), 1.22–1.24 m (C^4^H_2_, C^5^H_2_, C^6^H_2_, C^7^H_2_, C^8^H_2_, C^9^H_2_, 12H), 1.22 t (NCCH_3_, 18H, ^3^*J*_HH_ 7.1), 0.86 t (C^10^H_3_, 3H, ^3^*J*_HH_ 7.1). ^13^C-{^1^H} NMR spectrum (100.6 MHz, CDCl_3_, δ_C_ ppm, *J* Hz): 39.48 d (NCH_2_, ^2^*J*_PNC_ 3.4), 31.58 s (C^8^), 30.67 d (C^2^, ^2^*J*_PCC_ 17.9), 22.37 d (C^3^, ^3^*J*_PCCC_ 3.7), 29.22 s (C^5^), 29.04 s and 29.00 s (C^6^, C^7^), 28.97 br. s (C^4^), 25.80 d (C^1^, ^1^*J*_PC_ 104.5), 22.31 s (C^9^), 13.90 s (C^10^), 13.30 d (NCCH_3_, ^3^*J*_PNCC_ 2.3). ^31^P-{^1^H} NMR spectrum (162.0 MHz, CDCl_3_): δ_P_ 58.7 ppm.

Tris(diethylamino)(tetradecyl)phosphonium bromide **4e**, light yellow oil, yield was 1.91 g (91%). Anal. C, 59.81; H, 11.47; N, 8.29; P, 6.22%, calcd for C_26_H_59_BrN_3_P, C, 59.52; H, 11.33; N, 8.01; P, 5.90%. ESI-MS, *m*/*z*: 444.38 [M–Br]^+^; calcd for C_26_H_59_N_3_P^+^ 444.44. ^1^H NMR spectrum (400.0 MHz, CDCl_3_, δ ppm, *J* Hz): 3.15 dq (NCH_2_, 12H, ^3^*J*_PNCH_ 10.6, ^3^*J*_HH_ 7.1), 2.54 m (C^1^H_2_, 2H), 1.51 m (C^2^H_2_, C^3^H_2_, 4H), 1.23 m and 0.85 t (C^4^H_2_-C^13^H_2_, NCCH_3_, 38H), 0.85 t (C^14^H_3_, 3H, ^3^*J*_HH_ 7.0). ^1^H NMR spectrum (500.0 MHz, CD_3_CN, δ ppm, *J* Hz): 2.95 dq (NCH_2_, 12H, ^3^*J*_PNCH_ 10.7, ^3^*J*_HH_ 7.0), 2.31 m (C^1^H_2_, 2H), 1.32 m (C^2^H_2_, C^3^H_2_, 4H), 1.07–1.09 m, 1.03 br.m, 1.02 t (C^4^H_2_-C^13^H_2_, NCCH_3_, 38H), 0.66 t (C^14^H_3_, 3H, ^3^*J*_HH_ 7.1). ^13^C NMR spectrum (125.8 MHz, CDCl_3_, δ_C_ ppm, *J* Hz) (here and further, the signal type in the ^13^C-{^1^H} NMR spectrum is shown in parentheses): 39.30 tqd (d) (NCH_2_, ^1^*J*_HC_ 137.9, ^2^*J*_PNC_ 3.5, ^2^*J*_HCC_ 4.0), 31.43 tm (s) (C^12^, ^1^*J*_HC_ 130.3,), 30.47 tdm (d) (C^2^, ^1^*J*_HC_ 124.7, ^2^*J*_PCC_ 19.7), 29.18 tm (s) (C^6^, ^1^*J*_HC_ 125.2), 29.15 tm (s) (C^7^ and C^8^, ^1^*J*_HC_ 125.2), 29.13 tm (s) (C^9^, ^1^*J*_HC_ 125.2), 29.04 tm (s) (C^10^, ^1^*J*_HC_ 125.0–126.0,), 28.85 tm (br. s) (C^5^, C^11^, ^1^*J*_HC_ 127.5–128.0), 28.75 tm (d) (C^4^, ^1^*J*_HC_ 127.0–128.0, ^4^*J*_PCCCC_ 1.5), 25.0 tdm (d) (C^1^, ^1^*J*_HC_ 128.6, ^1^*J*_PC_ 104.4, ^2^*J*_HCC_ 4.0, ^3^*J*_HCCC_ 2.6), 22.19 tm (s) (C^13^, ^1^*J*_HC_ 128.1), 22.17, tm (d) (C^3^, ^1^*J*_HC_ 127.0–128.0, ^3^*J*_PNCC_ 2.6, ^3^*J*_PCCC_ 4.5). ^31^P-{^1^H} NMR spectrum (162.0 MHz, CH_3_CN): δ_P_ 60.4 ppm. ^31^P-{^1^H} NMR spectrum (162.0 MHz, CDCl_3_): δ_P_ 59.9 ppm.

Bis(diethylamino)(hexyl)(phenyl)phosphonium iodide **5a**, light yellow oil, yield was 1.80 g (97%). Anal. C, 51.93; H, 7.90; N, 5.89; P, 6.45%, calcd for C_20_H_38_IN_2_P, C, 51.72; H, 8.25; N, 6.03; P, 6.67%. ESI-MS, *m*/*z*: 337.17 [M–I]^+^; calcd for C_20_H_38_N_2_P^+^ 337.28. ^1^H NMR spectrum (400.0 MHz, CDCl_3_, δ ppm, *J* Hz): 7.39–7.42 m (H*^o^*, H*^m^*, H*^p^*, 5H), 2.92 m (NCH_2_, 8H, ^3^*J*_PNCH_ 10.3, ^3^*J*_HH_ 7.1), 2.45 m (C^1^H_2_, 2H), 1.11 m (C^2^H_2_, C^3^H_2_, 4H), 0.87–0.88 two m, 0.87 and 0.88 two t (C^4^H_2_, C^5^H_2_, NCCH_3_, 16H, ^3^*J*_HH_ 7.1), 0.46 br. m (C^6^H_3_, 3H). ^13^C NMR spectrum (100.6 MHz, CDCl_3_, δ_C_ ppm, *J* Hz): 134.45 dtd (d) (C*^p^*, ^1^*J*_HC_ 162.6, ^3^*J*_HCCC_ 7.0, ^4^*J*_PCCCC_ 2.8), 132.11 dddd (d) (C*^o^*, ^1^*J*_HC_ 162.9, ^2^*J*_PCC_ 10.5, ^3^*J*_HCCC_ 6.6–6.8, ^3^*J*_HCCC_ 6.6), 129.93 ddd (d) (C*^m^*, ^1^*J*_HC_ 165.8, ^3^*J*_PCCC_ 13.1, ^3^*J*_HCCC_ 6.6), 120.72 dt (d) (C*^i^*, ^1^*J*_PC_ 122.9, ^3^*J*_HCCC_ 7.3), 40.20 tqd (d) (NCH_2_, ^1^*J*_HC_ 137.9, ^2^*J*_HCC_ 3.7, ^2^*J*_PNC_ 2.6), 30.63 tm (s) (C^4^, ^1^*J*_HC_ 122.0–124.0), 29.58 tdm (d) (C^2^, ^1^*J*_HC_ 125.0–126.0, ^2^*J*_PCC_ 17.1), 24.54 tdm (d) (C^1^, ^1^*J*_HC_ 129.8, ^1^*J*_PC_ 81.9, ^3^*J*_HCCC_ 3.6–3.7, ^2^*J*_HCC_ 3.6–3.7), 21.81 tm (d) (C^3^, ^1^*J*_HC_ 126.0–128.0, ^2^*J*_HCC_ 3.7–4.0, ^3^*J*_PCCC_ 3.4), 21.69 tm (s) (C^5^, ^1^*J*_HC_ 126.0–127.0), 13.44 qm (s) (C^6^, ^1^*J*_HC_ 124.0–125.0), 13.38 qdt (d) (NCCH_3_, ^1^*J*_HC_ 126.9, ^3^*J*_PNCC_ 3.0, ^2^*J*_HCC_ 2.8–3.0). ^31^P-{^1^H} NMR spectrum (162.0 MHz, CDCl_3_): δ_P_ 58.9 ppm.

Bis(diethylamino)(octyl)(phenyl)phosphonium iodide **5b**, light yellow oil, yield was 1.87 g (95%). Anal. C, 53.93; H, 8.70; P, 6.55%, calcd for C_22_H_42_IN_2_P, C, 53.66; H, 8.60; N, 5.69; P, 6.29%. ESI-MS, *m*/*z*: 365.28 [M–I]^+^; calcd for C_22_H_42_N_2_P^+^ 365.31. ^31^P-{^1^H} NMR spectrum (162.0 MHz, CDCl_3_): δ_P_ 59.4 ppm. ^1^H NMR spectrum (400.0 MHz, CDCl_3_, δ ppm, *J* Hz): 7.54–7.60 m (H*^o^*, H*^m^*, H*^p^*, 5H), 3.10 m (NCH_2_, 8H, ^3^*J*_PNCH_ 10.5, ^3^*J*_HH_ 7.1), 2.63 m (C^1^H_2_, 2H), 1.20–1.29 m (C^2^H_2_, C^3^H_2_, 4H), 1.06–1.01 t and br. m (NCCH_3_, 12H, ^3^*J*_HH_ 7.1, C^4^H_2_-C^7^H_2_, 8H), 0.63 t (C^8^H_3_, 3H, ^3^*J*_HH_ 7.0). ^13^C NMR spectrum (100.6 MHz, CDCl_3_, δ_C_ ppm, *J* Hz): 134.44 dtd (d) (C*^p^*, ^1^*J*_HC_ 164.2, ^3^*J*_HCCC_ 7.0, ^4^*J*_PCCCC_ 2.9), 132.15 dddd (d) (C*^o^*, ^1^*J*_HC_ 162.7, ^2^*J*_PCC_ 10.5, ^3^*J*_HCCC_ 7.3, ^3^*J*_HCCC_ 7.1), 129.94 ddd (d) (C*^m^*, ^1^*J*_HC_ 165.6, ^3^*J*_PCCC_ 13.1, ^3^*J*_HCCC_ 7.1), 120.88 dt (d) (C*^i^*, ^1^*J*_PC_ 122.5, ^3^*J*_HCCC_ 6.6), 40.32 tqd (d) (NCH_2_, ^1^*J*_HC_ 138.0, ^2^*J*_HCC_ 4.0, ^2^*J*_PNC_ 2.9), 31.07 tm (s) (C^6^, ^1^*J*_HC_ 126.5), 29.92 tdm (d) (C^2^, ^1^*J*_HC_ 125.0–126.0, ^2^*J*_PCC_ 16.9), 28.47 tm (d) (C^4^, ^1^*J*_HC_ 122.0, ^1^*J*_HC_ 126.0, ^4^*J*_PCCCC_ 1.3, ^3^*J*_HCCC_ 3.4, ^2^*J*_HCC_ 3.4), 28.28 tm (s) (C^5^, ^1^*J*_HC_ 126.3) 24.69 tdm (d) (C^1^, ^1^*J*_HC_ 129.8, ^1^*J*_PC_ 81.7, ^3^*J*_HCCC_ 4.0–4.1, ^2^*J*_HCC_ 3.4–3.7,), 21.99 tm (s) (C^7^, ^1^*J*_HC_ 124.4), 21.92 tm (d) (C^3^, ^1^*J*_HC_ 126.0–128.0, ^3^*J*_PCCC_ 3.6), 13.51 qm (s) (C^8^, ^1^*J*_HC_ 124.4, 128.0, ^3^*J*_HCCC_ 2.8–2.9, ^2^*J*_HCC_ 2.8–2.9), 13.41 qdt (d) (NCCH_3_, ^1^*J*_HC_ 127.0, ^3^*J*_PNCC_ 3.0, ^2^*J*_HCC_ 2.9–3.0).

Bis(diethylamino)(nonyl)(phenyl)phosphonium iodide **5c**, light yellow oil, yield was 1.94 g (96%). C, 54.79; H, 8.90; P, 5.85%, calcd for C_23_H_44_IN_2_P, C, 54.54; H, 8.70; N, 5.53; P, 6.12%. ESI-MS, *m*/*z*: 379.30 [M–I]^+^; calcd for C_23_H_44_N_2_P^+^ 379.32. ^1^H NMR spectrum (400.0 MHz, CDCl_3_, δ ppm, *J* Hz): 7.68–7.78 m (H*^o^*, H*^m^*, H*^p^*, 5H), 3.27 m, (NCH_2_, 8H, ^3^*J*_PNCH_ 10.5, ^3^*J*_HH_ 7.2), 2.84 m (C^1^H_2_, 2H), 1.37–1.46 m (C^2^H_2_, C^3^H_2_, 4H), 1.23 and 1.18 two br. m (C^4^H_2_-C^8^H_2_, 10 H), 1.23 t (NCCH_3_, 12H, ^3^*J*_HH_ 7.2), 0.82 t (C^9^H_3_, 3H, ^3^*J*_HH_ 7.1). ^13^C NMR spectrum (100.6 MHz, CDCl_3_, δ_C_ ppm, *J* Hz): 134.33 dtd (d) (C*^p^*, ^1^*J*_HC_ 163.7, ^3^*J*_HCCC_ 7.3, ^4^*J*_PCCCC_ 2.9), 132.07 dddd (d) (C*^o^*, ^1^*J*_HC_ 162.8, ^2^*J*_PCC_ 10.5, ^3^*J*_HCCC_ 7.1, ^3^*J*_HCCC_ 6.8), 129.85 ddd (d) (C*^m^*, ^1^*J*_HC_ 165.2, ^3^*J*_PCCC_ 13.1, ^3^*J*_HCCC_ 7.0), 120.78 dt (d) (C*^i^*, ^1^*J*_PC_ 122.5, ^3^*J*_HCCC_ 7.7), 40.23 tdq (d) (NCH_2_, ^1^*J*_HC_ 137.8, ^2^*J*_HCC_ 4.0, ^2^*J*_PNC_ 2.8), 31.10 tm (s) (C^7^, ^1^*J*_HC_ 126.8), 29.82 tdm (d) (C^3^, ^1^*J*_HC_ 125.0–126.0, ^3^*J*_PCCC_ 16.9), 28.49 tm (br. s) (C^4^, ^1^*J*_HC_ 122.0–124.0, 28.44 tm (s) (C^5^, C^6^, ^1^*J*_HC_ 124.0–125.0), 24.0 tdm (d) (C^1^, ^1^*J*_HC_ 130.0, ^1^*J*_PC_ 81.7), 21.92 tm (s) (C^8^, ^1^*J*_HC_ 124.0), 21.87 tm (d) (C^3^, ^1^*J*_HC_ 126.0–128.0, ^3^*J*_PCCC_ 3.3), 13.45 tm (s) (C^9^, ^1^*J*_HC_ 124.7, ^3^*J*_HCCC_ 3.0, ^2^*J*_HCC_ 3.0), 13.33 qdt (d) (NCCH_3_, ^1^*J*_HC_ 127.2, ^3^*J*_PNCC_ 3.1, ^2^*J*_HCC_ 2.8). ^31^P-{^1^H} NMR spectrum (162.0 MHz, CDCl_3_): δ_P_ 59.4 ppm.

Decylbis(diethylamino)(phenyl)phosphonium iodide **5d**, light yellow oil, yield was 1.98 g (95%). Anal. C, 55.65; H, 8.99; N, 5.02; P, 6.24%, calcd for C_24_H_46_IN_2_P, C, 55.38; H, 8.91; N, 5.38; P, 5.95%. ESI-MS, *m*/*z*: 393.31 [M–I]^+^; calcd for C_24_H_46_N_2_P^+^ 393.34. ^1^H NMR spectrum (400.0 MHz, CDCl_3_, δ ppm, *J* Hz): 7.59–7.65 m (H*^o^*, H*^m^*, H*^p^*, 5H), 3.15 m, (NCH_2_, 8H, ^3^*J*_PNCH_ 10.8, ^3^*J*_HH_ 7.1), 2.70 m (C^1^H_2_, 2H), 1.29–1.32 m (C^2^H_2_, C^3^H_2_, 4H), 1.11 t (NCCH_3_, 12H, ^3^*J*_HH_ 7.1), 1.11 and 1.06 two m (C^4^H_2_-C^9^H_2_, 12H), 0.71 t (C^2^H_2_ and 1.18 two br. m (C^4^H_2_-C^8^H_2_, 10 H), 1.23 t (NCH_3_, 12H, ^3^*J*_HH_ 7.2), 0.71 t (C^10^H_3_, 3H, ^3^*J*_HH_ 7.0). ^13^C NMR spectrum (100.6 MHz, CDCl_3_, δ_C_ ppm, *J* Hz): 134.48 dtd (d) (C*^p^*, ^1^*J*_HC_ 163.4, ^3^*J*_HCCC_ 6.3, ^4^*J*_PCCCC_ 2.9), 132.20 dddd (d) (C*^o^*, ^1^*J*_HC_ 162.1, ^2^*J*_PCC_ 10.6, ^3^*J*_HCCC_ 7.2, ^3^*J*_HCCC_ 7.1), 129.98 ddd (d) (C*^m^*, ^1^*J*_HC_ 165.4, ^3^*J*_PCCC_ 13.1, ^3^*J*_HCCC_ 7.0), 120.93 dt (d) (C*^i^*, ^1^*J*_PC_ 122.5, ^3^*J*_HCCC_ 7.5), 40.37 tqd (d) (NCH_2_, ^1^*J*_HC_ 127.9, ^2^*J*_HCC_ 4.0, ^2^*J*_PNC_ 2.9), 31.29 tm (s) (C^8^, ^1^*J*_HC_ 124.4, ^3^*J*_HCCC_ 3.2–3.5, ^2^*J*_HCC_ 3.5–4.0), 29.97 tdm (d) (C^2^, ^1^*J*_HC_ 126.0–127.0, ^2^*J*_PCC_ 16.8), 28.89 tm (s) (C^5^, ^1^*J*_HC_ 126.0–127.0), 28.68 (C^6^, C^7^, ^1^*J*_HC_ 127.0–1280), 28.38 tm (d) (C^4^, ^1^*J*_HC_ 127.0–128.0, ^4^*J*_PCCCC_ 1.2), 24.75 tm (d) (C^1^, ^1^*J*_HC_ 129.2, ^1^*J*_PC_ 81.7), 22.10 tm (s) (C^9^, ^1^*J*_HC_ 124.4, ^3^*J*_HCCC_ 3.7, ^2^*J*_HCC_ 3.7), 21.98 tdm (d) (C^3^, ^1^*J*_HC_ 128.0, ^3^*J*_PCCC_ 3.5), 13.60 qm (C^10^, ^1^*J*_HC_ 124.3, ^3^*J*_HCCC_ 3.3–3.4, ^2^*J*_HCC_ 3.5–4.0), 13.45 qdt (NCCH_3_, ^1^*J*_HC_ 127.1, ^3^*J*_PNCC_ 3.0, ^2^*J*_HCC_ 3.0). ^31^P-{^1^H} NMR spectrum (162.0 MHz, CDCl_3_): δ_P_ 59.2 ppm.

(Diethylamino)(hexyl)diphenylphosphonium iodide **6a**, colorless crystals, yield was 1.84 g (98%), mp 91–93 °C. Anal. C, 56.55; H, 6.89; N, 3.21; P, 6.44%, calcd for C_22_H_33_INP, C, 56.29; H, 7.09; N, 2.98; P, 6.60%. ESI-MS, *m*/*z*: 342.13 [M–I]^+^; calcd for C_22_H_33_NP^+^ 342.24. ^1^H NMR spectrum (400.0 MHz, CDCl_3_, δ ppm, *J* Hz): 7.85 m and 7.81 m (H*^o^*, H*^p^*, 6H, ^3^*J*_PCCH*o*_ 12.4, ^3^*J*_H*o*H*m*_ 7.7, ^3^*J*_H*m*H*p*_ 7.7, ^5^*J*_PCCCCH*p*_ 1.5, ^4^*J*_H*m*CCCH*p*_ 1.4), 7.73 m (H*^m^*, 4H, ^3^*J*_H*p*H*m*_ 7.7, ^3^*J*_H*o*H*m*_ 7.7, ^4^*J*_PCCCH*m*_ 3.5), 3.31 dq and 3.30 m (NCH_2_, C^1^H_2_, 6H), 1.50–1.54 m (C^2^H_2_, C^3^H_2_, 4H), 1.21–1.23 m (C^4^H_2_, C^5^H_2_, 4H), 1.14 t (NCCH_3_, 6H), 0.81 br. t (C^6^H_3_, 3H, ^3^*J*_HH_ 7.1). ^13^C NMR spectrum (100.6 MHz, CDCl_3_, δ_C_ ppm, *J* Hz): 134.33 dtd (d) (C*^p^*, ^1^*J*_HC_ 163.3, ^3^*J*_HCCC_ 7.4, ^4^*J*_PCCCC_ 2.6), 132.14 dddd (d) (C*^o^*, ^1^*J*_HC_ 163.5, ^3^*J*_HCCC_ 7.4, ^2^*J*_PCC_ 10.4), 129.61 ddd (d) (C*^m^*, ^1^*J*_HC_ 165.6, ^3^*J*_PCCC_ 12.6, ^3^*J*_HCCC_ 7.3), 119.40 dt (d) (C*^i^*, ^1^*J*_PC_ 96.4, ^3^*J*_HCCC_ 7.4), 40.95 tqd (d) (NCH_2_, ^1^*J*_HC_ 138.3, ^2^*J*_HCC_ 4.1, ^2^*J*_PNC_ 2.3), 30.24 tm (s) (C^4^, ^1^*J*_HC_ 124.7), 29.22 tdm (d) (C^2^, ^1^*J*_HC_ 127.0–128.0, ^2^*J*_PCC_ 16.0), 23.71 tdm (d) (C^1^, ^1^*J*_HC_ 130.6, ^1^*J*_PC_ 63.1, ^3^*J*_HCCC_ 3.0–4.0, ^2^*J*_HCC_ 3.0–4.0), 21.47 tm (d) (C^3^, ^1^*J*_HC_ 124.3, ^2^*J*_HCC_ 3.7–4.0, ^3^*J*_HCCC_ 3.7–4.0, ^3^*J*_PCCC_ 3.5), 21.31 tm (s) (C^5^, ^1^*J*_HC_ 125.3), 13.12 qdt (d) (NCCH_3_, ^1^*J*_HC_ 127.1, ^3^*J*_PNCC_ 2.4, ^2^*J*_HCC_ 2.7), 13.08 qm (s) (C^6^, ^1^*J*_HC_ 124.5, ^3^*J*_HCCC_ 3.7–4.0, ^2^*J*_HCC_ 3.7–4.0). ^31^P-{^1^H} NMR spectrum (162.0 MHz, CDCl_3_): δ_P_ 52.0 ppm.

(Diethylamino)(octyl)diphenylphosphonium iodide **6b**, light yellow oil, yield was 1.91 g (96%). Anal. C, 58.35; H, 7.19; N, 3.03; P, 6.44%, calcd for C_24_H_37_INP, C, 57.95; H, 7.50; N, 2.82; P, 6.24%. ESI-MS, *m*/*z*: 370.22 [M–I]^+^. calcd for C_24_H_37_NP^+^ 370.27. ^1^H NMR spectrum (400.0 MHz, CDCl_3_, δ ppm, *J* Hz): 7.78–7.83 m and 7.79 m (H*^o^*, H*^p^*, 6H, ^3^*J*_PCCH*o*_ 12.4, ^3^*J*_H*o*H*m*_ 7.7, ^3^*J*_H*m*H*p*_ 7.7, ^5^*J*_PCCCCH*p*_ 1.5, ^4^*J*_H*m*CCCH*p*_ 1.4), 7.70 m (H*^m^*, 4H, ^3^*J*_H*p*H*m*_ 7.7, ^3^*J*_H*o*H*m*_ 7.7, ^4^*J*_PCCCH*m*_ 3.5), 3.27 dk and 3.23 m (NCH_2_, C^1^H_2_, 6H, ^3^*J*_PNCH_ 11.3, ^3^*J*_HH_ 7.1), 1.45–1.48 m (C^2^H_2_, C^3^H_2_, 4H), 1.14–1.18 m and 1.10 t (C^4^H_2_, C^5^H_2_, C^6^H_2_, C^7^H_2_, NCCH_3_, 14H, ^3^*J*_HH_ 7.1), 0.77 t (C^8^H_3_, 3H, ^3^*J*_HH_ 7.1). ^13^C NMR spectrum (100.6 MHz, CDCl_3_, δ_C_ ppm, *J* Hz): 134.68 dtd (d) (C*^p^*, ^1^*J*_HC_ 163.3, ^3^*J*_HCCC_ 7.1, ^4^*J*_PCCCC_ 2.8), 132.48 dddd (d) (C*^o^*, ^1^*J*_HC_ 163.6, ^3^*J*_HCCC_ 7.0, ^2^*J*_PCC_ 10.4), 129.96 ddd (d) (C*^m^*, ^1^*J*_HC_ 165.8, ^3^*J*_PCCC_ 12.6, ^3^*J*_HCCC_ 7.4), 119.77 dt (d) (C*^i^*, ^1^*J*_PC_ 96.4, ^3^*J*_HCCC_ 7.2), 41.31 tdq (d) (NCH_2_, ^1^*J*_HC_ 138.5, ^2^*J*_HCC_ 4.2, ^2^*J*_PNC_ 2.3), 31.07 tm (s) (C^6^, ^1^*J*_HC_ 126.5), 29.92 tdm (d) (C^2^, ^1^*J*_HC_ 125.0–126.0, ^2^*J*_PCC_ 16.0), 28.46 tm (br. s) (C^4^, ^1^*J*_HC_ 124.0–126.0), 28.25 tm (s) (C^6^, ^1^*J*_HC_ 126.4), 24.09 tdm (d) (C^1^, ^1^*J*_HC_ 131.2, ^1^*J*_PC_ 63.0, ^3^*J*_HCCC_ 3.0–4.0, ^2^*J*_HCC_ 3.0–4.0), 21.98 tm (s) (C^7^, ^1^*J*_HC_ 124.5), 21.87 tm (d) (C^3^, ^1^*J*_HC_ 126.0–128.0, ^3^*J*_PCCC_ 3.5), 13.51 qm (s) (C^8^, ^1^*J*_HC_ 124.4, ^3^*J*_HCCC_ 3.1–3.6, ^2^*J*_HCC_ 3.1–3.6), 13.44 qdt (d) (NCCH_3_, ^1^*J*_HC_ 127.3, ^3^*J*_PNCC_ 2.6, ^2^*J*_HCC_ 2.8). ^31^P-{^1^H} NMR spectrum (162.0 MHz, CDCl_3_): δ_P_ 51.9 ppm.

(Diethylamino)(nonyl)diphenylphosphonium iodide **6c**, light yellow oil, yield was 1.94 g (95%). Anal. C, 58.45; H, 7.89; N, 2.96; P, 5.84%, calcd for C_25_H_39_INP, C, 58.71; H, 7.69; N, 2.74; P, 6.07%. ESI-MS, *m*/*z*: 384.18 [M–I]^+^; calcd for C_25_H_39_NP^+^ 384.28. ^31^P-{^1^H} NMR spectrum (162.0 MHz, CH_3_CN): δ_P_ 52.2 ppm. ^1^H NMR spectrum (400.0 MHz, CDCl_3_, δ ppm, *J* Hz): 7.70 m and 7.68 m (H*^o^*, H*^p^*, 6H, ^3^*J*_PCCH*o*_ 12.4, ^3^*J*_H*o*H*m*_ 7.7, ^3^*J*_H*m*H*p*_ 7.7, ^5^*J*_PCCCCH*p*_ 1.5, ^4^*J*_H*m*CCCH*p*_ 1.4), 7.60 m (H*^m^*, 4H, ^3^*J*_H*p*H*m*_ 7.7, ^3^*J*_H*o*H*m*_ 7.7, ^4^*J*_PCCCH*m*_ 3.5), 3.16 dq (NCH_2_, 4H, ^3^*J*_PNCH_ 11.3, ^3^*J*_HH_ 7.1), 3.10 m (C^1^H_2_, 2H), 1.35–1.38 m (C^2^H_2_, C^3^H_2_, 4H), 1.05–1.08 m and 1.04 br. m (C^4^H_2_, C^5^H_2_, C^6^H_2_, C^7^H_2_, C^8^H_2_, 10H), 1.0 t (NCCH_3_, 6H, ^3^*J*_HH_ 7.1), 0.67 t (C^9^H_3_, 3H, ^3^*J*_HH_ 7.1).^13^C NMR spectrum (100.6 MHz, CDCl_3_, δ_C_ ppm, *J* Hz): 134.79 dtd (d) (C*^p^*, ^1^*J*_HC_ 164.2, ^3^*J*_HCCC_ 7.1, ^4^*J*_PCCCC_ 2.9), 132.59 dddd (d) (C*^o^*, ^1^*J*_HC_ 163.6, ^3^*J*_HCCC_ 7.4, ^2^*J*_PCC_ 10.6), 130.07 ddd (d) (C*^m^*, ^1^*J*_HC_ 165.8, ^3^*J*_PCCC_ 12.6, ^3^*J*_HCCC_ 7.3), 119.88 dt (d) (C*^i^*, ^1^*J*_PC_ 96.4, ^3^*J*_HCCC_ 8.4), 41.42 tqd (d) (NCH_2_, ^1^*J*_HC_ 138.4, ^2^*J*_HCC_ 4.0, ^2^*J*_PNC_ 3.0), 31.31 tm (s) (C^7^, ^1^*J*_HC_ 125.0–126.0), 30.04 tdm (d) (C^2^, ^1^*J*_HC_ 125.0–126.0, ^2^*J*_PCC_ 16.0), 28.68 and 28.66 two tm (two s) (C^5^, C^6^, ^1^*J*_HC_ 124.0–125.0), 28.64 tm (br. s) (C^4^, ^1^*J*_HC_ 122.0–124.0), 24.23 tdm (d) (C^1^, ^1^*J*_HC_ 131.1, ^1^*J*_PC_ 63.0), 22.14 tm (s) (C^8^, ^1^*J*_HC_ 124.2), 21.99 tm (d) (C^3^, ^1^*J*_HC_ 126.0–128.0, ^3^*J*_PCCC_ 3.7), 13.66 qm (s) (C^9^, ^1^*J*_HC_ 124.5, ^3^*J*_HCCC_ 3.5–4.0, ^2^*J*_HCC_ 3.5–4.0), 13.55 qdt (d) (NCCH_3_, ^1^*J*_HC_ 127.3, ^3^*J*_PNCC_ 2.6, ^2^*J*_HCC_ 2.7). ^31^P-{^1^H} NMR spectrum (162.0 MHz, CDCl_3_): δ_P_ 52.0 ppm.

Decyl(diethylamino)diphenylphosphonium iodide **6d**, light yellow oil, yield was 1.97 g (94%). Anal. C, 59.65; H, 8.18; N, 2.83; P, 6.14%, calcd for C_26_H_41_INP, C, 59.43; H, 7.86; N, 2.67; P, 5.90%. ESI-MS, *m*/*z*: 398.27 [M–I]^+^; calcd for C_26_H_41_NP^+^ 398.30. ^1^H NMR spectrum (400.0 MHz, CDCl_3_, δ ppm, *J* Hz): 7.79 m and 7.76 m (H*^o^*, H*^p^*, 6H, ^3^*J*_PCCH*o*_ 12.4, ^3^*J*_H*o*H*m*_ 7.7, ^3^*J*_H*m*H*p*_ 7.7, ^5^*J*_PCCCCH*p*_ 1.4, ^4^*J*_H*m*CCCH*p*_ 1.3), 7.68 m (H*^m^*, 4H, ^3^*J*_H*p*H*m*_ 7.7, ^3^*J*_H*o*H*m*_ 7.7, ^4^*J*_PCCCH*m*_ 3.5), 3.25 dq (NCH_2_, 4H, ^3^*J*_PNCH_ 11.3, ^3^*J*_HH_ 7.1), 3.20 m (C^1^H_2_, 2H), 1.44–1.48 m (C^2^H_2_, C^3^H_2_, 4H), 1.15–1.17 m and 1.12 br. m (C^4^H_2_, C^5^H_2_, C^6^H_2_, C^7^H_2_, C^8^H_2_, C^9^H_2_, 12H), 1.08 t (NCCH_3_, 6H, ^3^*J*_HH_ 7.1), 0.77 t (C^10^H_3_, 3H, ^3^*J*_HH_ 7.1). ^13^C NMR spectrum (100.6 MHz, CDCl_3_, δ_C_ ppm, *J* Hz): 135.07 dtd (d) (C*^p^*, ^1^*J*_HC_ 163.3, ^3^*J*_HCCC_ 7.3, ^4^*J*_PCCCC_ 2.9), 132.90 dddd (d) (C*^o^*, ^1^*J*_HC_ 163.5, ^3^*J*_HCCC_ 7.1, ^2^*J*_PCC_ 10.5), 130.34 ddd (d) (C*^m^*, ^1^*J*_HC_ 165.8, ^3^*J*_PCCC_ 12.6, ^3^*J*_HCCC_ 7.4), 120.33 dt (d) (C*^i^*, ^1^*J*_PC_ 96.6, ^3^*J*_HCCC_ 7.4), 41.73 tqd (d) (NCH_2_, ^1^*J*_HC_ 139.5, ^2^*J*_HCC_ 4.2, ^2^*J*_PNC_ 2.7), 31.67 tm (s) (C^8^, ^1^*J*_HC_ 126.0–127.0), 30.36 tdm (d) (C^2^, ^1^*J*_HC_ 125.0–126.0, ^2^*J*_PCC_ 16.0), 29.28 tm (s) (C^7^, ^1^*J*_HC_ 127.1), 29.07 and 29.04 two tm (two s) (C^5^, C^6^, ^1^*J*_HC_ 124.0–125.0), 28.97 tm (br. s) (C^4^, ^1^*J*_HC_ 122.0–124.0), 24.55 tdm (d) (C^1^, ^1^*J*_HC_ 132.1, ^1^*J*_PC_ 62.9), 22.48 tm (s) (C^9^, ^1^*J*_HC_ 124.0–125.0), 22.31 tm (d) (C^3^, ^1^*J*_HC_ 126.0–128.0, ^3^*J*_PCCC_ 3.5), 13.96 qm (s) (C^10^, ^1^*J*_HC_ 125.1), 13.82 qdt (d) (NCCH_3_, ^1^*J*_HC_ 127.4, ^3^*J*_PNCC_ 2.6, ^2^*J*_HCC_ 2.8). ^31^P-{^1^H} NMR spectrum (162.0 MHz, CDCl_3_): δ_P_ 51.1 ppm.

(Diethylamino)diphenyl(tetradecyl)phosphonium bromide **6e**, light yellow oil, yield was 1.99 g (93%). Anal. C, 67.19; H, 8.89; N, 2.73; P, 6.14%, calcd for C_30_H_49_BrNP, C, 67.40; H, 9.24; N, 2.62; P, 5.80%. ESI-MS, *m*/*z*: 454.35 [M–Br]^+^; calcd for C_30_H_49_NP^+^ 454.36. ^1^H NMR spectrum (400.0 MHz, CDCl_3_, δ ppm, *J* Hz): 7.85 m (H*^o^*, 4H, ^3^*J*_PCCH*o*_ 12.3–13.4, ^3^*J*_H*o*H*m*_ 7.7, ^4^*J*_H*o*H*p*_ 1.4), 7.79 m (H*^p^*, 2H, ^3^*J*_H*p*H*m*_ 7.7, ^4^*J*_H*p*H*o*_ 1.4, ^5^*J*_PH*p*_ 1.6), 7.70 m (H*^m^*, 4H, ^3^*J*_H*m*H*o*_ 7.7, ^3^*J*_HH_ 7.7, ^4^*J*_PH_ 3.5), 3.37 m (C^1^H_2_, 2H), 3.30 dq (d) (NCH_2_, 4H, ^3^*J*_PNCH_ 11.3, ^3^*J*_HH_ 7.1), 1.48–1.49 m (C^2^H_2_, C^3^H_2_, 4H), 1.21 and 1.16 two br. m (C^4^H_2_-C^13^H_2_, 20H), 1.12 t (NCCH_3_, 6H, ^3^*J*_HH_ 7.1), 0.84 t (C^14^H_3_, 3H, ^3^*J*_HH_ 7.1). ^1^H NMR spectrum (500.0 MHz, CDCl_3_, δ ppm, *J* Hz): 7.54 m (H*^o^*, 4H, ^3^*J*_PH*o*_ 12.4, ^3^*J*_H*o*H*m*_ 7.7), 7.47 m (H*^p^*, 2H, ^3^*J*_H*p*H*m*_ 7.1), 7.40 m (H*^m^*, 4H, ^3^*J*_H*m*H*o*_ 7.7, ^3^*J*_HH_ 7.7, ^4^*J*_PH_ 3.5), 2.97–3.00 m (NCH_2_, C^1^H_2_, 6H, ^3^*J*_PNCH_ 11.3, ^3^*J*_HH_ 7.1), 1.16–1.17 m (C^2^H_2_, C^3^H_2_, 4H), 0.86–0.88 m, 0.83 br. m, 0.79 t (C^4^H_2_-C^13^H_2_, NCCH_3_, 26H, ^3^*J*_HH_ 7.1), 0.48 t (C^14^H_3_, 3H, ^3^*J*_HH_ 7.1). ^13^C NMR spectrum (125.8 MHz, CDCl_3_, δ_C_ ppm, *J* Hz): 134.24 dtd (d) (C*^p^*, ^1^*J*_HC_ 163.7, ^3^*J*_HCCC_ 7.5, ^4^*J*_PCCCC_ 2.5), 132.12 dddd (d) (C*^o^*, ^1^*J*_HC_ 164.1, ^2^*J*_PCC_ 10.4, ^3^*J*_HCCC_ 7.2, ^3^*J*_HCCC_ 6.9), 129.53 ddd (d) (C*^m^*, ^1^*J*_HC_ 166.0, ^3^*J*_PCCC_ 12.6, ^3^*J*_HCCC_ 7.4), 119.54 dt (d) (C*^i^*, ^1^*J*_PC_ 96.3, ^3^*J*_HCCC_ 8.2), 40.78 tqd (d) (NCH_2_, ^1^*J*_HC_ 139.3, ^2^*J*_HCC_ 4.0, ^2^*J*_PNC_ 1.9), 30.95 tm (s) (C^12^, ^1^*J*_HC_ 126.0), 29.55 tdm (d) (C^2^, ^1^*J*_HC_ 124.0–125.0, ^2^*J*_PCC_ 16.0), 28.70 tm (s) (C^6^, ^1^*J*_HC_ 124.0–125.0), 28.67 tm (s) (C^7^, C^8^, ^1^*J*_HC_ 124.0–125.0), 28.63 tm (br. s) (C^4^, ^1^*J*_HC_ 124.0–125.0), 28.54 tm (s) (C^10^, ^1^*J*_HC_ 124.0–125.0), 28.37 tm (s) (C^11^, ^1^*J*_HC_ 124.0–125.0), 28.26 tm (s) (C^5^, ^1^*J*_HC_ 124.0–125.0), 28.17 tm (br. s) (C^4^, ^1^*J*_HC_ 124.0–125.0), 23.42 tdm (d) (C^1^, ^1^*J*_HC_ 130.9, ^1^*J*_PC_ 62.8, ^3^*J*_HCCC_ 3.0–4.0), 21.72 tm (s) (C^13^, ^1^*J*_HC_ 126.5), 21.52 tm (d) (C^3^, ^1^*J*_HC_ 128–129.0, ^3^*J*_PCCC_ 3.4), 13.22 qm (s) (C^14^, ^1^*J*_HC_ 124.5, ^3^*J*_HCCC_ 3.3–4.0), 12.97 qdt (d) (NCCH_3_, ^1^*J*_HC_ 127.3, ^2^*J*_HCC_ 2.7, ^3^*J*_PNCC_ 2.2). ^31^P-{^1^H} NMR spectrum (162.0 MHz, CDCl_3_): δ_P_ 52.0 ppm. ^31^P-{^1^H} NMR spectrum (162.0 MHz, CH_3_CN): δ_P_ 51.9 ppm.

Triethyloctylammonium iodide **7**. The mixture of triethylamine (3 g, 29.7 mmol) and octyl iodide (6.42 g, 26.8 mmol) in 5 mL of dry acetonitrile was refluxed for 1 h. Acetonitrile was removed in vacuo, the remaining powder was washed with diethyl ether (3 × 4 mL) and was dried in vacuo. Yield was 8.66 g (95%), mp 122–123 °C. (Lit. 94–97 °C [83]). Anal. C, 49.58; H, 9.55; N, 4.39%, calcd for C_14_H_32_IN, C, 49.27; H, 9.45, N, 4.10%. ESI-MS, *m*/*z*: 214.22 [M–I]^+^; calcd for C_14_H_32_N^+^ 214.25. ^1^H NMR spectrum (400.0 MHz, CDCl_3_, δ ppm, *J* Hz): 3.47 q (NCH_2_, 6H, ^3^*J*_HH_ 7.3), 3.26 m (C^1^H_2_, 2H), 1.68 m (C^2^H_2_, 2H), 1.34–1.36 m and 1.38 t (C^3^H_2_, C^4^H_2_, NCCH_3_, 13H), 1.25–1.27 (C^5^H_2_, C^6^H_2_, C^7^H_2_, 6H), 0.87 t (C^8^H_3_, 3H, ^3^*J*_HH_ 7.1). ^13^C NMR spectrum (100.6 MHz, CDCl_3_, δ_C_ ppm, *J* Hz): 56.74 br. t (s) (C^1^, ^1^*J*_HC_ 142.0), 52.81 br. t (s) (NCH_2_, ^1^*J*_HC_ 133.5), 30.54 tm (s) (C^6^, ^1^*J*_HC_ 125.2), 28.66 tm (s) (C^4^, ^1^*J*_HC_ 128.3), 28.61 tm (s) (C^5^, ^1^*J*_HC_ 128.3), 25.38 (C^3^, ^1^*J*_HC_ 125.1), 21.49 (C^2^, ^1^*J*_HC_ 124.7), 21.17 (C^7^, ^1^*J*_HC_ 127.6), 13.07 (C^8^, ^1^*J*_HC_ 124.6), 7.43 (NCCH_3_, ^1^*J*_HC_ 128.7, ^2^*J*_HCC_ 3.5).

Octyltriphenylphosphonium iodide **8**. The mixture of triphenylphosphine (1.09 g, 4.16 mmol) and octyl iodide (1 g, 4.17 mmol) in 5 mL of dry acetonitrile was refluxed for 1 h. Acetonitrile was removed under reduced pressure, and the obtained oil was washed with diethyl ether (4 × 5 mL) and was dried in vacuo. Yield was 2 g (96%). Anal. C, 61.89; H, 6.03; P, 5.89%, calcd for C_26_H_32_IP, C, 62.16; H, 6.42; P, 6.17%. ESI-MS, *m*/*z*: 375.25 [M–I]^+^; calcd for C_26_H_32_P^+^ 375.22. ^1^H NMR spectrum (400.0 MHz, CDCl_3_, δ ppm, *J* Hz): 7.76–7.78 m (H*^o^*, H*^p^*, 9H), 7.69 m (H*^m^*, 6H, ^3^*J*_HH_ 7.4, ^3^*J*_HH_ 7.4, ^4^*J*_PCCCH_ 3.5), 3.55 br. m (PCH_2_, 2H), 1.59 m (C^2^H_2_, C^3^H_2_, 4H), 1.16–1.21 m (C^4^H_2_-C^7^H_2_, 8H), 0.79 t (C^8^H_3_, 3H, ^3^*J*_HH_ 7.0). ^13^C NMR spectrum (here and further, the signal type in the ^13^C-{^1^H} NMR spectrum is shown in parentheses) (125.8 MHz, CDCl_3_, δ ppm, *J* Hz): 134.71 dtd (d) (C*^p^*, ^1^*J*_HC_ 163.8, ^3^*J*_HCCC_ 7.4, ^4^*J*_PCCCC_ 2.4), 133.07 dddd (d) (C*^o^*, ^1^*J*_HC_ 163.5, ^2^*J*_PCC_ 9.8, ^3^*J*_HCCC_ 7.7, ^3^*J*_HCCC_ 7.6), 130.13 ddd (d) (C*^m^*, ^1^*J*_HC_ 166.1, ^3^*J*_PCCC_ 12.3, ^3^*J*_HCCC_ 7.2), 117.45 dt (d) (C*^i^*, ^1^*J*_PC_ 85.8, ^3^*J*_HCCC_ 8.8), 31.05 tm (s) (C^6^, ^1^*J*_HC_ 128.2), 29.88 tdm (d) (C^2^, ^1^*J*_HC_ 128.0–129.0, ^2^*J*_PCC_ 15.7), 28.45 tm (br. s) (C^4^, ^1^*J*_HC_ 124.7), 28.43 tm (s) (C^5^, ^1^*J*_HC_ 124.0–125.0), 22.60 tdm (d) (C^1^, ^1^*J*_HC_ 131.1, ^1^*J*_PC_ 49.3), 21.95 tm (s) (C^7^, ^1^*J*_HC_ 124.0–125.0), 21.96 tm (d) (C^3^, ^1^*J*_HC_ 124.0–125.0, ^2^*J*_PCC_ 4.0–5.0), 13.50 qm (s) (C^8^, ^1^*J*_HC_ 124.1). ^31^P-{^1^H} NMR spectrum (162.0 MHz, CDCl_3_): δ_P_ 24.9 ppm.

1,6-Hexanediyl-bis(hexaethyltriaminophosphonium)dibromide **9**. The mixture of hexaethyltriaminophosphine (1.98 g, 8 mmol) and 1,6-dibromohexane (0.98 g, 4 mmol) in 7 mL of anhydrous acetonitrile was stirred for 10 h. Acetonitrile was removed under reduced pressure, and the obtained oil was washed with diethyl ether (4 × 5 mL) and was dried in vacuo. Light yellow oil, yield was 2.83 g (96%). Anal. C, 49.03; H, 9.55; N, 11.02; P, 8.79%, calcd for C_30_H_72_Br_2_N_6_P_2_, C, 48.78; H, 9.82; N, 11.38; P, 8.40%. ESI-MS, *m*/*z*: 289.12 [M–2Br]^2+^; calcd for C_30_H_72_N_6_P^2+^ 289.26. ^1^H NMR spectrum (400.0 MHz, CDCl_3_, δ ppm, *J* Hz): 2.66 br. m (NCH_2_, 24H, ^3^*J*_PNCH_ 8.1, ^3^*J*_HH_ 6.5), 2.46 br. m (C^1^H_B_, 2H), 2.18 br. m (C^1^H_A_, 2H), 1.15–1,16 m (C^2^H_2_, 4H), 0.88 m (C^3^H_2_, 4H), 0.73 br. t (NCCH_3_, 36H). ^13^C NMR spectrum (100.6 MHz, CDCl_3_, δ_C_ ppm, *J* Hz): 38.42 tdq (d) (NCH_2_, ^1^*J*_HC_ 138.0, ^2^*J*_PNC_ 3.8, ^2^*J*_HCC_ 4.0), 28.62 tdm (d) (C^2^, ^1^*J*_HC_ 128.5, ^2^*J*_PCC_ 18.7), 24.32 tdm (d) (C^1^, ^1^*J*_HC_ 129.6, ^1^*J*_PC_ 104.1, ^3^*J*_HCCC_ 2.0, ^2^*J*_HCC_ 1.8–2.0,), 21.15 tm (d) (C^3^, ^1^*J*_HC_ 134.4, ^3^*J*_PCCC_ 4.0), 12.29 qdt (d) (NCCH_3_, ^1^*J*_HC_ 127.0, ^3^*J*_PNCC_ 2.6, ^2^*J*_HCC_ 2.7). ^31^P-{^1^H} NMR spectrum (162.0 MHz, CDCl_3_): δ_P_ 60.3 ppm.

### 2.3. Preparation and Characterization of Lipid Nanoparticles

#### 2.3.1. Chemicals

*L*-α-phosphatidylcholine (PC) (Soy, 95%, Avanti polar lipids), Precirol^®^ ATO 5 (glyceryl palmitostearate) was a gift from Gattefossé (St-Priest, France), Kolliphor P 188 (Lutrol^®^ F68, Poloxamer 188) (oxyethylene, 79.9–83.7%, Sigma-Aldrich, St. Luis, MO, USA), Rhodamine B (99%, ACROS Organics, Morris Plains, NJ, USA), Curcumine (Sigma-Aldrich, Chengdu, China), Ultra-purified water (18.2 MΩcm resistivity at 25 °C) was produced from Direct-Q 5 UV equipment (Millipore S.A.S. 67120 Molsheim, France). All reagents were used without further treatment.

#### 2.3.2. Preparation of Liposomes Modified by Aminophosphonium Salts

*L*-α-phosphatidylcholine (PC) (5% *w*/*w*) and compounds **4**, **5**, **7,** and **8** were dissolved in 1 mL of ethanol. The homogeneous solution was kept in a water bath at 60 °C until complete alcohol evaporation to obtain a thin lipid film. Ultra-purified water was pre-heated to 60 °C and added to rehydrate the lipids at 60 °C in the absence. The solution was stirred under magnetic stirring (750 rpm) (Ika, Staufen, Germany) for 30 min at the same temperature. Furthermore, the solution was kept for 1.5 h in a water bath at 37 °C. The multilamellar liposome systems were extruded 15 times by passage through a polycarbonate membrane of 100 nm pore size (Mini-Extruder Extrusion Technique, Avanti Polar Lipids, Inc., Alabaster, AL, USA). The same method was used for rhodamine B (0.05% *w*/*w*)-labeled liposomes.

#### 2.3.3. Preparation of SLN Modified by Aminophosphonium Salts

Briefly, Precirol^®^ ATO 5 (1.5% *w*/*w*) was melted at 70 °C, and compound 5 (0.01% *w*/*w*) was added to the melted lipid and dissolved. A pre-emulsion was formed after dispersing the hot lipid phase in a Poloxamer 188 surfactant solution (1% *w*/*w*) using Ultra-Turrax^®^ (IKA, T18, Germany) at 8000 rpm for 5 min. The obtained pre-emulsion was passed through a high-pressure homogenizer (Avestin Emulsiflex C5, Avestin, Ottawa, ON, Canada) at 70 °C, applying a pressure of 1200 bar for 15 min. The obtained aqueous dispersions were filled in glass vials, which were immediately sealed and stored at room temperature (20 °C). The total volume is 50 mL. The same method was used for curcumine-labeled SLN and TPP-SLN by adding 50 μL curcumin (C = 0.01 M) in methanol to the melted lipid and keeping it until the evaporation of methanol and the dissolution of curcumine.

#### 2.3.4. Characterization by DLS

The mean particle size, zeta potential, and polydispersity index were determined by dynamic light scattering (DLS), using a Malvern Instrument Zetasizer Nano (Malvern, Worcestershire, UK). The size (hydrodynamic diameter, nm) was calculated according to the Einstein–Stokes relationship *D = k_B_T/3πηx*, where *D* is the diffusion coefficient, *k_B_* is the Boltzmann’s constant, *T* is the absolute temperature, *η* is the viscosity, and *x* is the hydrodynamic diameter of nanoparticles. The diffusion coefficient was determined at least in triplicate for each sample. The average error of measurements was approximately 10%. All lipid samples were diluted with ultra-purified water to a suitable concentration (2.5 mg/mL) and analyzed in triplicate.

#### 2.3.5. In Vitro Rhodamine B Release Profile

The monitoring of rhodamine B release from liposomes was performed using the dialysis bag diffusion method. Dialysis bags retain liposomes and allow the released rhodamine B to diffuse into the medium. The bags were soaked in Milli-Q water for 12 h before use. Furthermore, 0.5 mL of liposomes were poured into the dialysis bag. The two bag ends were sealed with clamps. The bags were then placed in a vessel containing 100 mL of 0.025 M sodium phosphate buffer, pH 7.4, for the receiving phase. The vessel was placed in a thermostatic shaker at 37 °C with a stirring rate of 150 rpm. At predetermined time intervals, 0.5 mL of samples were withdrawn, and their absorbance at 554 nm was measured using Perkin Elmer λ35 (PerkinElmer Instruments, Norwalk, CT, USA). All samples were analyzed in triplicate. The extinction coefficient of rhodamine B is 106,089 M^–1^ cm^–1^ at pH = 7.4.

### 2.4. Biological Study

#### 2.4.1. Cells and Materials

For experiments, we used tumor cell cultures M-HeLa clone 11, a human epithelioid carcinoma of the cervix (subline HeLa., clone M-HeLa); T 98G, a human glioblastoma; Hep G2, a human liver carcinoma; PANC-1, a human pancreatic carcinoma; HuTu 80, a human duodenal adenocarcinoma; MCF7, a human breast adenocarcinoma (pleural fluid); A549, a human lung carcinoma; WI38, VA 13 subline 2RA, a human embryonic lung from the collection of the Institute of Cytology (Russian Academy of Sciences, St. Petersburg, Russia); PC3, a human prostate adenocarcinoma cell line from ATCC (American Type Cell Culture Collection, Manassas, VA, USA; CRL 1435); human liver cells (Chang liver) from the collection Research Institute of Virology (Russian Academy of Medical Sciences, Moscow, Russia); SK-OV-3, a human ovarian adenocarcinoma; and DU-145, a human prostate carcinoma from the CLS Cell Lines Service cell repository. The cell lines presented above were cultured in a standard Eagle’s nutrient medium manufactured at the Chumakov Institute of Poliomyelitis and Virus Encephalitis (PanEco company, Moscow, Russia) and supplemented with 10% fetal calf serum and 1% nonessential amino acids. SK-OV-3, a human ovarian adenocarcinoma from the CLS Cell Lines Service cell repository, was cultured in DMEM/F-12 nutrient medium manufactured at the Chumakov Institute of Poliomyelitis and Virus Encephalitis (PanEco company) and supplemented with 2 mM L-glutamine and 5% FBS.

#### 2.4.2. Cell Toxicity Assay (MTT-Test)

The cytotoxic effect on cells was determined using the colorimetric method of cell proliferation—the MTT test. Cells were seeded onto a 96-well Nunc plate at a concentration of 5 × 10^3^ cells per well in a volume of 100 μL of medium and cultured in a CO_2_ incubator at 37 °C until a monolayer formed for approximately 24 h. Then the nutrient medium was removed, and the cells were washed with PBS. Dilutions of the test compounds, prepared directly in the culture medium with the addition of DMSO (5% by volume) to improve solubility, were added in 100 μL to each well of a 96-well plate. DMSO (5% *v*/*v*) was also added to control wells. This concentration did not inhibit cell viability. After 48-h incubation of cells with test compounds, the culture medium was removed from the dishes, and 100 μL of serum-free culture medium containing MTT at a concentration of 0.5 mg/mL was added and incubated for 4 h at 37 °C. 100 μL of DMSO was added to each well to form formazan crystals. Absorbance was recorded at a wavelength of 540 nm on an InvitroLogic microplate reader (Medico-Biological Union, Novosibirsk, Russia). Experiments for all compounds were repeated three times.

#### 2.4.3. Induction of Apoptotic Effects by Test Compounds and Flow Cytometry Assay

##### Cell Culture

HuTu 80 cells at a rate of 1 × 10^6^ cells/well were seeded into six-well plates and incubated until a monolayer formed for approximately 24 h. After 48 h of incubation, various concentrations of compounds **4b**, **5b**, **6a,** and **8** were added to the wells.

##### Cell Apoptosis Assay

Cells were detached with a trypsin-versene solution (1:3), suspended in PBS, and collected at 2000 rpm for 5 min. Cells were then washed twice with ice-cold PBS, followed by resuspension in binding buffer. Next, the samples were incubated with 5 μL of Annexin V Alexa Fluor 647 (Sigma-Aldrich, St. Louis, MO, USA) and 5 μL of propidium iodide for 15 min at room temperature in the dark. Finally, the cells were analyzed by flow cytometry in the Red-R (661/15 nm)—annexin and Near IR-B (785/70 nm)—PI channels on a Guava easyCyte flow cytometer (Merck Millipore, Burlington, MA, USA) for 1 h. The experiments were repeated three times.

##### Mitochondrial Membrane Potential

The cells are prepared as in the Cell Apoptosis Assay section. Cells were detached with trypsin/versene solution (1:3), suspended in PBS, collected at 2000 rpm for 5 min, then washed twice with ice-cold PBS, followed by resuspension in JC-10 (10 μg/mL) and incubation at 37 °C for 10 min. The cells were then washed three times and suspended in PBS, and JC-10 fluorescence was observed by flow cytometry (Guava Easy Cyte).

##### Detection of Intracellular ROS

HuTu 80 cells were incubated with compounds **4b**, **5b**, **6a,** and **8** at IC_50_/2 and IC_50_ concentrations for 48 h. ROS generation was investigated using a flow cytometry assay and the CellROX^®^ Deep Red flow cytometry kit. For this, HuTu 80 cells were harvested at 2000 rpm for 5 min and then washed twice with ice-cold PBS, followed by resuspension in 0.1 mL of medium without FBS, to which was added 0.2 μL of CellROX^®^ Deep Red and incubated at 37 °C for 30 min. After three washes, cells were suspended in PBS, and the cell production of ROS was immediately monitored using a flow cytometer (Guava easy Cyte).

##### ELISA Assay

HuTu 80 cells were incubated for 48 h with **4b**, **5b**, **6a,** and **8** at IC_50_/2 and IC_50_ concentrations. In vitro quantitative measurement of caspase-9 and caspase-8 was performed using ELISA kits (ELISA Kit for Caspase 9 and ELISA Kit for Caspase 8, Human Cloud-Clone Corp., Wuhan, China). The analysis was carried out according to the manufacturer’s instructions. Samples were standardized for total protein content (1 mg/mL). Protein concentration was determined with Coomassie-based assays using Bredford Dye Reagent (Bio-Rad, Hercules, CA, USA). The absorbance was measured using a microplate reader, EPOCH (BioTek Instruments, Inc., Winooski, VT, USA), at a wavelength of 450 ± 10 nm. Lysates of untreated HuTu 80 cells were used as controls.

##### Cell Cycle Analysis

The DNA content and cell-cycle distribution after compounds **4b**, **5b**, **6a,** and **8** IC_50_ concentrations were estimated by flow cytometry. After washing with PBS, treated cells were suspended in 150 μL of PBS, then 0.5 mL of phosphate-citrate buffer (0.05 M, pH 4.0) was added, and the suspension was incubated at room temperature for 5 min to facilitate the extraction of low-molecular-weight DNA. Following centrifugation, the cells were resuspended in 150 μL DNA staining solution (20 μg/mL propidium iodide, 200 μg/mL DNase (RNase-free)) and incubated in the CO_2_ incubator (37 °C for 30 min). The distribution of the cell cycle was determined by fluorescence analysis of HuTu 80 cells stained with propidium iodide using Guava easyCyte (Guava easyCyte) [65].

##### Hemolytic Assay

The hemolytic activity of Aminophosphonium Salts was assessed by comparing the optical density of a solution containing the test compound with the optical density of blood at 100% hemolysis. Healthy human volunteer donor erythrocytes were used as the object of the study. Red blood cells with EDTA (1.8 mg/mL) were washed three times with saline (0.9% NaCl), centrifuged for 10 min at 800× *g* rpm, and resuspended in saline (0.9% NaCl) to a concentration of 10%. The tested concentrations (0.1–100 μM) were prepared in physiological solution (0.9% NaCl) (with the addition of 5% DMSO). Compounds at the appropriate dilution (450 μL) were added to 50 μL of a 10% red blood cell suspension. Samples were incubated for 1 h at 37 °C and centrifuged for 10 min at 2000 rpm. The release of hemoglobin was controlled by measuring the optical density of the supernatant on an InvitroLogic microplate reader at λ = 540 nm. A control sample corresponding to zero hemolysis (blank experiment) was prepared by adding 50 μL of a 10% red blood cell suspension to 450 μL of saline (0.9% NaCl). A control sample corresponding to 100% hemolysis was prepared by adding 50 μL of a 10% red blood cell suspension to 450 μL of distilled water [84]. Blood was taken at the Clinic of the Federal Research Center “Kazan Scientific Center of the Russian Academy of Sciences” from healthy donors with their personal consent.

##### Cellular Uptake Study

HuTu80 cells in the number of 1 × 10^5^ cells/well in a final volume of 500 mL were sown in 24-well plates (Eppendorf). Cells were cultured until a monolayer formed (approximately 24 h). Then the nutrient medium was taken, the cells were washed with PBS, and solutions of PC-liposomes and PC/**5d**-liposomes at a concentration of IC_50_/2 were added to the wells and incubated for 1 h, 6 h, and 24 h in a CO_2_ incubator. PC-liposomes and PC/**5d**-liposomes contained RdB and coumarin as fluorescent probes. Next, the cells were detached with a trypsin/versene solution (1:3), suspended in PBS, and detected using a flow cytometer (Guava easyCyte). Flow cytometry was used to set up statistics on the uptake of drugs by cancer cells. Untreated cells were used as a negative control.

##### Fluorescence Microscopy

HuTu80 cells at 1 × 10^6^ cells/well in a final volume of 2 mL were seeded in 6-well plates at the bottom of each well. Cells were cultured until a monolayer formed (about 24 h). Then the nutrient medium was taken, the cells were washed with PBS, and solutions of PC-liposomes and PC/**5d**-liposomes at a concentration of IC_50_/2 were added to the wells and incubated for 1, 6, and 24 h in a CO_2_ incubator. PC liposomes and PC/**5d** liposomes contained RdB and coumarin as fluorescent probes. The fluorescent dye Hoechst 33342 (blue) was used to stain the nuclei of HuTu80 cells. Research has been carried out using a Nikon Eclipse Ci-S fluorescence microscope (Nikon, Tokyo, Japan) at 400× *g* and 1000× *g* magnification.

##### Antimicrobial Activity

The antimicrobial activity of test compounds was determined by serial microdilutions in 96-well plates using Mueller-Hinton broth for bacterial culture and Sabouraud broth for yeast culture. Cultures of Gram-positive bacteria were used in the experiment: *Staphylococcus aureus* ATCC 6538 P FDA 209P, *Bacillus cereus* ATCC 10702 NCTC 8035, *Enterococcus faecalis* ATCC 29212; Gram-negative bacteria: *Escherichia coli* ATCC 25922, *Pseudomonas aeruginosa* ATCC 9027; and yeast: *Candida albicans* ATCC 10231, purchased from the State Collection of Pathogenic Microorganisms and Cell Cultures “SCPM-Obolensk” (Obolensk, Russia). Methicillin-resistant strains of S. aureus (MRSA) were isolated from patients with chronic tonsillitis (MRSA-1) and sinusitis (MRSA-2) in the bacteriological laboratory of the Republican Clinical Hospital (Kazan, Russia). The experiments were carried out in triplicate.

##### Statistical Analysis

The IC_50_ values were calculated using the MLA—Quest Graph™ IC_50_ Calculator (AAT Bioquest, Inc., Pleasanton, CA, USA). Statistical analysis was performed using the Mann-Whitney test (*p* < 0.05). Tabular and graphical data contain averages and standard errors.

##### In Vivo Study of Acute Toxicity of Aminophosphonic Salts

The study of acute toxicity was performed on males of outbred white mice of the CD-1 line at the age of 2–3 months, weighing 20–30 g. The total number of mice participating in the experiment was 300. Mice were obtained from the Research and Production Enterprise Laboratory Animal Farm based at the Branch of Shemyakin and Ovchinnikov Institute of Bioorganic Chemistry of the Russian Academy of Sciences (Pushchino, Russia). The animals were kept in accordance with [85,86,87] in standard conditions in a vivarium with 12 h of daylight and free access to food and water. The animals were fed complete feed made according to specifications (protein 22%, fiber 4% max., fat 5% max, ash 9% max, humidity 13.5% max, caloric value 295 kcal/100 g). The studies were conducted with a single parenteral–intraperitoneal (intra-peritoneal) injection, according to the method [87].

The substances were introduced in a sterile saline DMSO (40% vol.) solution. Solutions of the substances were prepared immediately before administration. The total volume of the injected liquid was 0.1 mL/10 g of animal weight. As a control, a group of mice was administered under similar conditions an equivalent amount (0.1 mL/10 g, or 10 mL/kg) of a solvent—40% DMSO on sterile saline solution. The substances were administered in doses ranging from 1 to 100 mg/kg; there were six animals in each group. To assess acute toxicity, the animals were monitored 14 days after administration of the substance, assessing their general condition and recording deaths.

All animal experiments and protocols were approved by the Local Ethics Committee of Kazan Federal University (Protocol No. 4 dated 18 May 2017).

## 3. Results and Discussion

### 3.1. Chemistry

#### 3.1.1. Synthesis of Alkyl Diethylaminophoshonium Salts

**Scheme 2:**
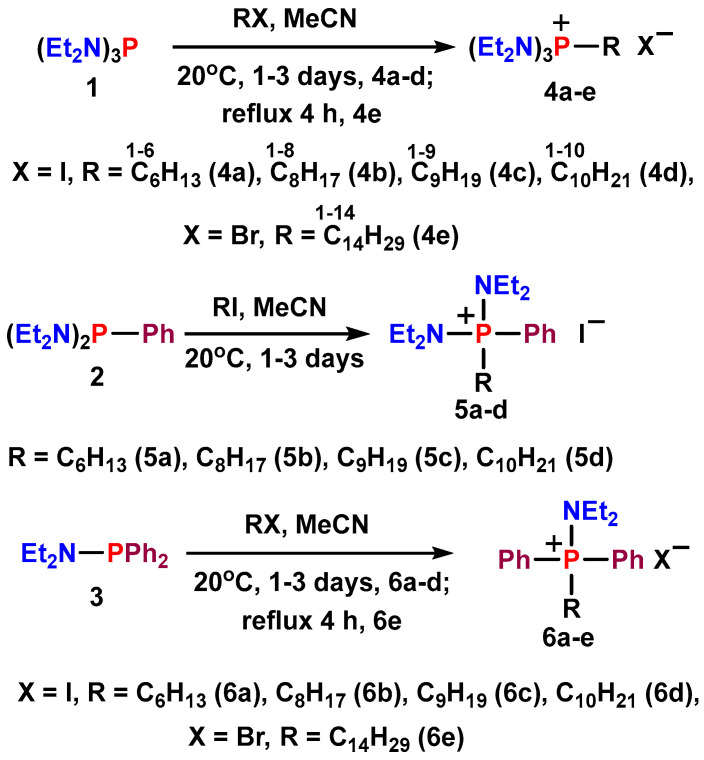
Synthesis of Alkyl Diethylaminophoshonium salts.

Aminophosphonium salts (APP) containing three, two, or one diethylamine group were synthesized using hexaethyltriaminophosphine **1**, bis(tetraethyldiamino) phenylphosphine **2,** and diethylaminodiphenylphosphine **3** in the reactions with alkyl halides (C_6_, C_8_, C_9_, C_10_, and C_14_). In works [88,89], the reaction of alkyl bromides C_6_–C_22_ with the analog of compound **1**—hexamethyltriaminophosphine—was carried out in the absence of solvent at 140–150 °C. Furthermore, reactions of aminophosphin-1 with decyl chloride and a mixture of C_16_-C_18_ bromides were carried out under hard conditions (130 °C) [90]. Meanwhile, the reaction of aminophosphine **1** with methyliodide [67] can proceed at room temperature. Using ^31^P NMR, we experimentally determined that reactions of **1**–**3** with alkyl iodides can be carried out at room temperature in a polar solvent; we used acetonitrile (Scheme 2). The heating is required for tetradecyl bromide using this solvent. Quasi-phosphonium salts **4**–**6**, light yellow thick oils, were obtained with yields close to quantitative (see Section 2.2 and Supplementary Materials). Only compound **6a** was obtained in crystalline form.

**Figure 1:**
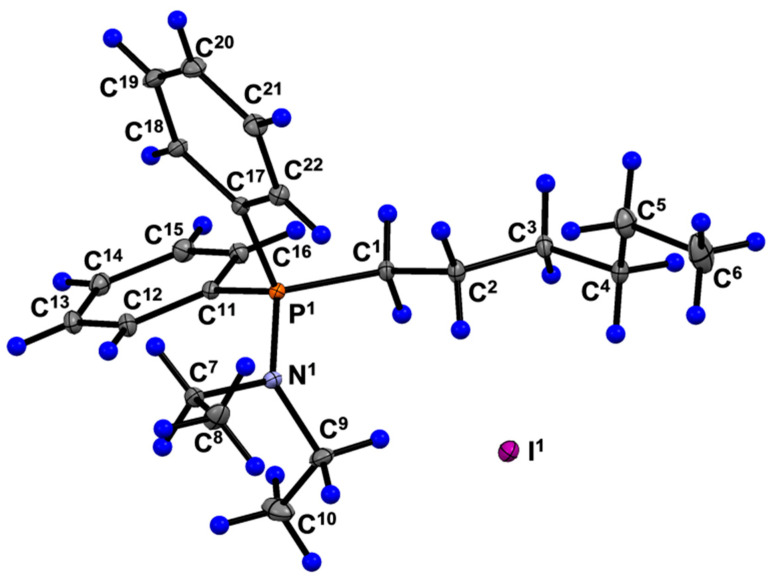
Geometry of **6a** in crystal. Non-hydrogen atoms are shown in view of thermal ellipsoids with a probability of 50%. Hydrogen atoms are shown as spheres of arbitrary radius. Selected bond lengths (Å) and bond angles (deg): P^1^–N^1^ 1.634(1), P^1^–C^1^ 1.797(1), P^1^–C^11^ 1.802(1), P^1^–C^17^ 1.793(1), N^1^–P^1^–C^1^ 110.64(6), N^1^–P^1^–C^11^ 115.35(6), N^1^–P^1^–C^17^ 106.83(5), C^1^–P^1^–C^11^ 106.75(6), C^1^–P^1^–C^17^ 109.67(6), C^11^–P^1^–C^17^ 107.49(6).

The structure of all the compounds obtained was determined by ^1^H, ^13^C, ^13^C-{^1^H}, ^13^C-{^1^H}-dept, ^31^P-{^1^H} NMR, and the composition was confirmed by ESI mass spectroscopy and elemental analysis. The most characteristic for quasi-phosphonium structures **4**–**6** are chemical shifts in the ^31^P-{^1^H} NMR spectra. Depending on the number of amine groups, they are in two intervals—δ_P_ 58–59 ppm (**4**, **5**) and 51–52 ppm (**6**). In the NMR spectra of ^13^C-{^1^H} quasi-phosphonium salts **5**, **6** containing phenyl groups, the most characteristic is the position of the carbon resonance C*^i^* (δ_C_ 119–123 ppm), and the values of the constants ^1^*J*_PC*i*_ are sensitive to the number of amino groups at the phosphonium center—122–123 Hz (for **5**) and ~96.4 Hz (for **6**). It is interesting to note that the values of the constants ^1^*J*_PC1_ are also very sensitive to the number of amino groups: they are ~104.5 Hz (for **4**), 81–82 Hz (for **5**), and ~63.0 Hz (for **6**). The structure of **6a** was proved by the XRD. Figure 1 shows the geometry of the molecule in a crystal. The phosphorus atom has a slightly distorted tetrahedral coordination; the valent angles near it vary from 106.76 (6) to 115.35 (6)°. The nitrogen atom has a strongly flattened pyramidal coordination; the sum of the valent angles near it is 356.0 (1)°. The basic geometric parameters of the molecules (bond lengths and valent angles) are ordinary. A fragment of a hexyl substituent with a phosphorus atom P^1^–C^1^–C^2^–C^3^ (up to the C^4^ atom) has a flat zigzag conformation (corresponding torsion angle –175.86(9)°), typical for paraffin crystals. Then a reversal occurs around the C^3^–C^4^ bond: the torsion angle C^2^–C^3^–C^4^–C^5^ is –67.4(2)°, and then the fragment C^3^–C^4^–C^5^–C^6^ is also in a flat zigzag conformation (the corresponding torsion angle is 178.7(1)°).

**Figure 2:**
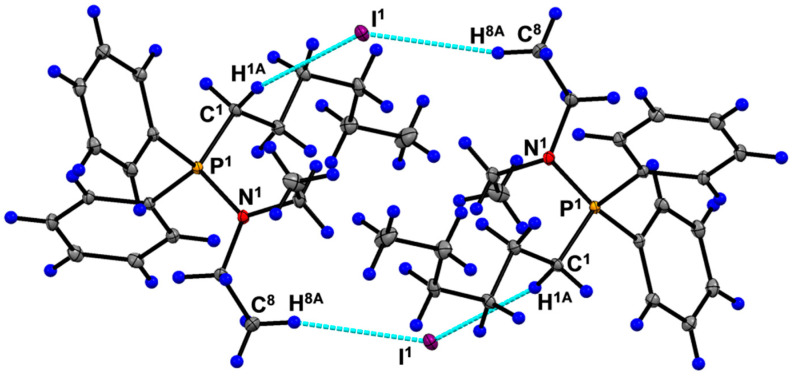
Short C–H⋯I-type contacts in crystal of **6a**.

In the crystal, the iodide anion has short C–H⋯I-type contacts with two neighboring phosphonium cations (Figure 2), and at the same time, a hexyl substituent approaches one of the phenyl substituents of the neighboring molecule. Apparently, the non-planar conformation of the hexyl substituent is determined by steric reasons.

**Scheme 3:**
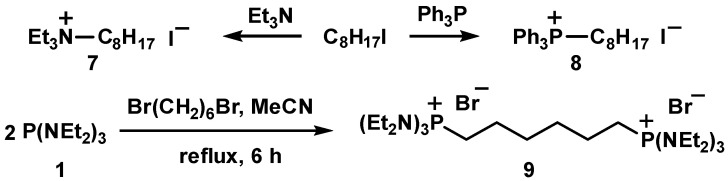
Synthesis of ammonium salt **7**, phosphonium salt **8** and diphosphonium salt **9**.

For comparison of biological activity of **4**–**6**, triethylammonium salt **7**, triphenylphosphonium salt **8** and 1,6-hexanediyl bis(hexaethyltriaminophosphonium)dibromide **9** (Scheme 3) were obtained. The structures of **7**–**9** were proven by NMR. It is interesting that the reaction of triphenylphosphine with octyl iodide in MeCN proceeds already at room temperature and without heating (95 h, reagent concentration ~0.25 mol/l, and conversion 71%). However, to accelerate the process, the reaction mass was heated for 1 h. We also carried out a competitive reaction of hexaethyltriaminophosphine **1** and triphenylphosphine with iodoctane in MeCN at room temperature. The ratio of phosphonium salts **4b** and **8** after 24 h of keeping the reaction mixture is 85:15, i.e., phosphine **1** has a higher reactivity. Since the rate-determining stage in the Arbuzov reaction is the nucleophilic attack of the P(III) atom on the electrophilic carbon atom of alkyl halide. Thus, aminophosphine, despite having more electro-negative nitrogen atoms, is a strong nucleophile compared with triphenylphosphine.

#### 3.1.2. Aminophosphonium Salt Decorated Liposomes and Solid Lipid Nanoparticles

Lipid nanosystems (liposomes and solid lipid nanoparticles) decorated by APP were obtained using widely used methods, including the lipid film hydration method and high-pressure hot homogenization. The physicochemical characteristics are presented in Table 2. Previously, we found the optimal PC/cationic amphiphile ratio = 99.8/0.2 for obtaining liposomal nanosystems with a positively charged surface [91,92] and SLN [65].

**Figure 3:**
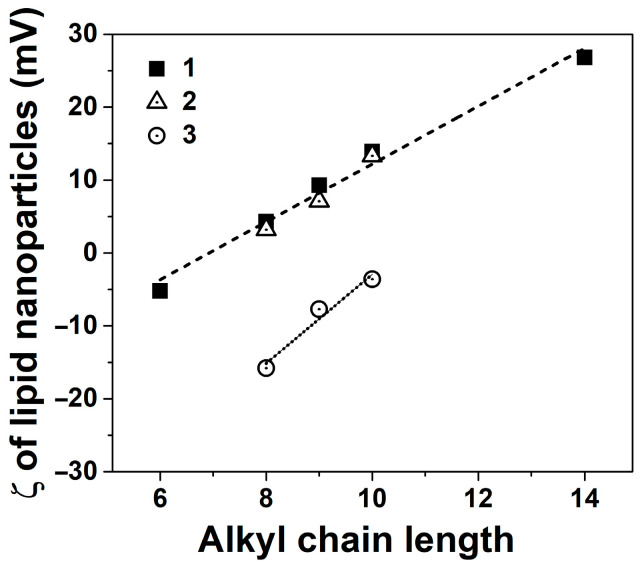
The zeta potential of liposomes (1, 2) and solid lipid nanoparticles (3) decorated with of aminophosphonium salts **4** (1) and **5** (2, 3) with different alkyl chain lengths.

The size of lipid nanoparticles is close to 100 nm. The polydispersity index is not higher than 0.15 for liposomal systems. Detailed DLS data on particle size distribution using the intensity and number parameters are presented in Supplementary Materials (Figures S166–S170). The zeta potential (ζ) of liposomal systems decorated with aminophosphonium salts 4 and 5 varies from neutral to positive values (Figure 3). This indicates a modification of the nanoparticle surface. Linearity (r^2^ = 0.995 and r^2^ = 0.991) of ζ change is observed with increasing length of the alkyl chain of aminophosphonium salts (*n*) from *n* = 6 to *n* = 14 for **4** and from *n* = 6 to *n* = 10 for **5**. This tendency does not depend on substituents at the phosphorus atom of amino-phosphonium salts (monoaminophosphonium and diaminophosphonium derivatives). The same tendency is observed for SLN/**5**. The surface charge of SLN changes from negative to positive values with linearity r^2^ = 0.983 (Figure 3). This indicates the modification of nanoparticles with phosphonium salts. An increase in the polydispersity index and the formation of smaller nanoparticles close to 20 nm occur with an increase in the alkyl chain for SLN/**5**. SLN/**4c** and SLN/**4d** systems showed that these systems were not stable at the pre-emulsion preparation stage. Therefore, only SLN/**4b** and SLN/**5b** were used for in vitro studies.

**Figure 4:**
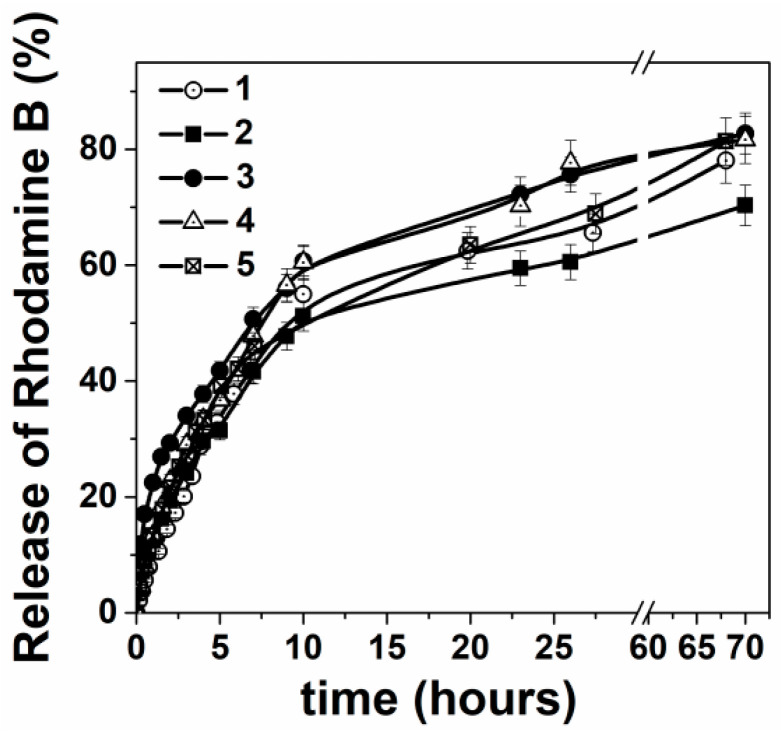
Release profile of Rhodamine B from PC-liposomes (1) and P^+^-PC-liposomes modified by aminophosphonium salt **4d** (2), **5b** (3), **5c** (4) and **5d** (5), (n = 3, 3 times were replicated), phosphate buffer (0.025 M), pH 7.4, 37 °C.

The release of the water-soluble dye rhodamine B from liposomes was studied to evaluate the release time of encapsulated drugs from liposomes and the rigidity of the phospholipid membrane. The release of rhodamine B from PC liposomes and liposomes modified with aminophosphonium salts is shown in Figure 4. Modification of liposomes with aminophosphonium salts does not affect the release of dye from liposomal systems. 50% of rhodamine B is released within 8 h, and complete release occurs within more than 70 h.

### 3.2. Biology

#### 3.2.1. Cytotoxic Effects of Aminophosphonium Salts

In primary studies, in vitro cell models of cancer and normal cell lines are widely used to evaluate the direct cytotoxic effect of the new chemical compounds proposed as potential drugs. Table 3 presents the cytotoxicity of aminophosphonium salts tested against a wide panel of human cancer and normal cell lines. The results are characterized by the values of half-inhibiting concentrations (IC_50_) as well as the selectivity index (SI). SI is calculated as the ratio between the IC_50_ for normal cells and IC_50_ for cancer cells. Most of the compounds displayed high and moderate activity toward the entire spectrum of used cell lines. In some cases, the cytotoxicity of tested compounds against normal cells was lower compared with cancer cells. At the same time, the selectivity index of the studied compounds was SI ≥ 10. This value of SI indicates high selectivity [93].

Most of the studied compounds generally exhibit high cytotoxicity against the M-HeLa cell line. Thus, for compounds **4b–e**, **5b–d**, **6b–e**, the IC_50_ values are in the range 0.24–1.8 μM (SI 1.6–17). At the same time, compounds **4d** (SI 7.1), **6a** (SI 4.8), and **6b** (SI 17) are distinguished by the SI indicator, with compound **6b** being a leader, whose IC_50_ is two times lower than that of doxorubicin.

On the HuTu 80 cell line, a pronounced dependence of SI on the aminophosphonium cation is observed: at a rather low IC_50_ value of 0.06–4.0 μM for compounds **4**–**6** (for compounds **4e** and **6e**, IC_50_ is 3 times lower than for doxorubicin), the selectivity index increases in a row **4** < **5** < **6**. Thus, the maximum SI (SI 28) is observed for **4e** among triaminophosphonium salts, SI 210 for **5a** among diaminophosphonium salts, and SI 277 for **6a** among monoaminophosphonium salts. **6b** (SI 81) and **6c** (SI 42) indicate high selectivity properties. A similar activity is observed for the PC3 cells. Their growth is rather selectively inhibited by **6b**, **6c,** and **6a,** indicating IC_50_ (μM) and SI: 0.085 ± 0.07 (19), 1.0 ± 0.08 (8.3), and 4.0 ± 0.3 (6.9), respectively. Among diaminophosphonium compounds, only salt **5a** (4.9 ± 0.4 μM, SI 13) exhibits selectivity, whereas in the series of derivatives **4,** there are no compounds with high SI values.

Monoaminophosphonium compounds **6a–c**, **e** showed higher efficiency for the Du-145 cell line compared with **4** and **6** with the following IC_50_ (μM) and SI: **6b**, 0.8 ± 0.07 (20); **6a**, 2.6 ± 0.3 (11); **6e**, 0.4 ± 0.02 (7.0); **6c**, 1.3 ± 0.1 (6.4). Among tri- and diaminophosphonium compounds, **4d**, **4c,** and **5c** with IC_50_ (μM) and SI can be noted: 1.2 ± 0.1 (10), 0.4 ± 0.03 (4.3), and 0.9 ± 0.07 (4.6). The same trends are observed in the PANC-1 cell line. The most effective compounds are **6b**, **6e**, **6c**, **6a**, **4e**, and **4c** with the following IC_50_ (μM) and SI (given in order of decreasing SI): **6b**, 1.3 ± 0.1 (12); **6e**, 0.3 ± 0.02 (9.3); **4d**, 1.6 ± 0.1 (7.5); **6c**, 1.3 ± 0.1 (6.4); **6a**, 5.5 ± 0.4 (5.50); and **4e**, 0.4 ± 0.03 (4.3).

The studied compounds showed moderate or high cytotoxicity on the MCF-7 cell line. In a series of compounds with three amino groups at the phosphorus atom, compound **4e** showed the highest cytotoxicity (IC_50_ 0.9 ± 0.07 μM, SI 1.7). **4d** (IC_50_ 2.1 ± 0.2 μM) showed a high selectivity index (SI 5.7). The replacement of one or two amino groups with a phenyl substituent in compounds **5b–d**, **6c–e**, did not lead to an increase in cytotoxicity with respect to MCF-7 cell line, which was 0.7–4.2 μM (SI 2). The IC_50_ and SI of octyltriphenylphosphonium iodide **8** (4.2 ± 0.3, 2.2) were comparable with the activity of **6c**.

For the A549 cell line, only the activity of compound **5a** can be noted. **5a** has lower efficiency (IC_50_ 17.0 ± 0.3 μM, SI 3.7), and it is 157 times less toxic toward the normal WI38 cell line compared with doxorubicin (IC_50_ 0.7 ± 0.06 μM, ns).

The analysis of IC_50_ and SI allows a preliminary conclusion about the more pronounced cytotoxicity and higher SI values of monoaminophosphonium derivatives **6** compared with tri- and diaminophosphonium salts **4** and **5** on HuTu 80, PC3, and Du-145 cell lines. The effect of alkyl chain length in aminophosphonium derivatives is expressed in an increase in cytotoxicity towards cancer cells from derivatives with a smaller alkyl chain to larger ones. This is most clearly noticeable from C_6_ to C_8_ [IC_50_ **4b** > **4a** (ΔIC_50_ 50.6 μM for M-HeLa), **5b** > **5a** (ΔIC_50_ 21.2 μM for M-HeLa), **6b** > **6a** (ΔIC_50_ 4.9 μM for M-HeLa)].

**Figure 5:**
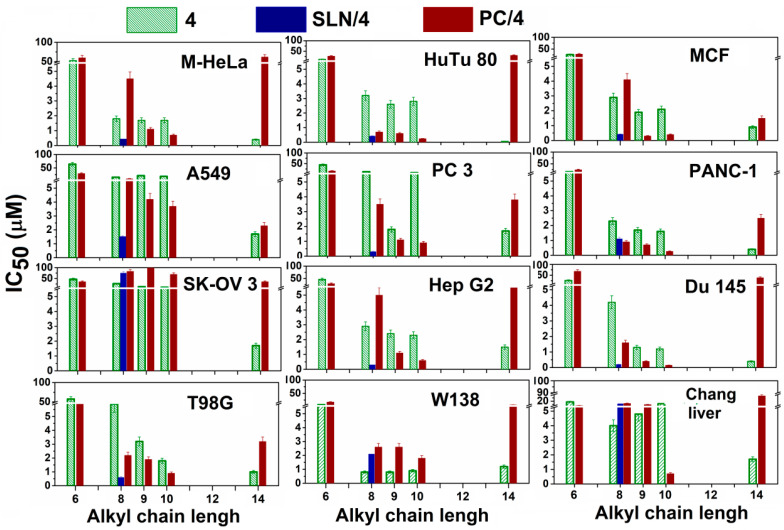
Summarized results showing the IC_50_ for compounds **4** with different alkyl chain length and lipid formulations SLN/**4** and PC/**4**-liposomes in cancer cells and normal cells after 24 h of incubation.

**Figure 6:**
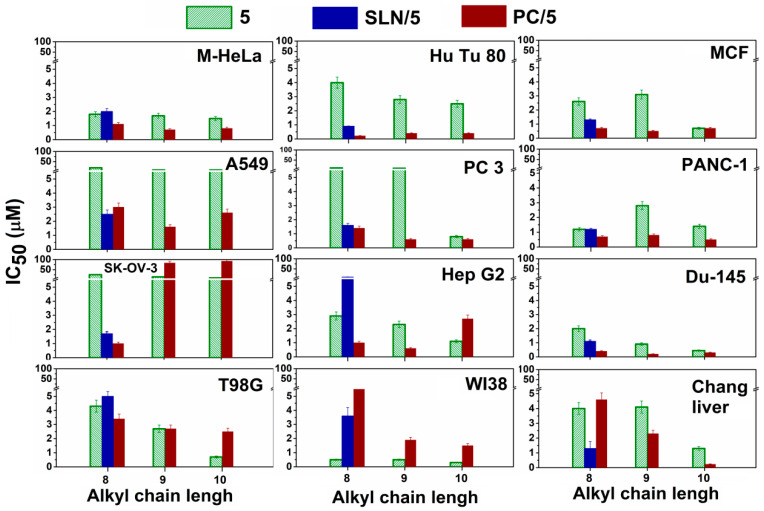
Summarized results showing the IC_50_ for compounds **5** with different alkyl chain length and lipid formulations SLN/**5** and PC/**5**-liposomes in cancer cells and normal cells after 24 h of incubation.

In vitro cytotoxicity was studied for nanotherapeutic forms (liposomes and SLN) of compounds **4**, **5b–5d**, **7,** and **8**. Lipid systems for **4b–d** and **5b–d** exhibit improved cytotoxic activity against tumor cells (except SK-OV-3) compared with individual compounds (Figure 5 and Figure 6). The maximum difference is observed for SLN/**4b**, where the cytotoxicity is 4 times (M-HeLa) and 40 times (PC3) better than for **4b**. The IC_50_ for the lipid systems PC/**4c**, PC/**4d**, PC/**5b**, PC/**5c**, PC/**5d,** and SLN/**4b** is lower than for the reference drug DOX. At the same time, all lipid nanoforms are less toxic to normal cell lines. Maximum selectivity SI 53 and SI 56 are achieved in the case of SLN/**4b** against the cell lines MCF-7 and DU-145, respectively. The maximum SI is observed for PC/**4c** and PC/**4e**; the SI values are SI 30 and 24 for the MCF-7 cell line and SI 22.8 for DU-145, as well as PC/8, where SI 36 and 24 against cell lines A549 and T98G.

It should be noted that the activity of PC/4 improves with an increase in alkyl chain length from the hexyl to the decyl derivatives in the range of 8÷456 times, in contrast to PC/**5**. There is no change in activity depending on the length of the alkyl chain for the PC/**5**. Interestingly, the surface charge of both PC/**4** and PC/**5** liposomes changes linearly with increasing length of the alkyl chain (Figure 3) and does not depend on the substituent at the phosphorus atom. Consequently, biological activity is not linearly related to the charge of liposomal systems but depends mainly on the nature of the substituent at the phosphorus atom. In addition, SLN/**4b** exhibits improved biological activity compared with both SLN/**5b** and PC/**4b** liposomal systems, which also indicates the influence of the nature of the substituent at the phosphorus atom.

#### 3.2.2. Cellular Uptake of Liposomes and SLN Modified by Aminophosphonium Salts

**Figure 7:**
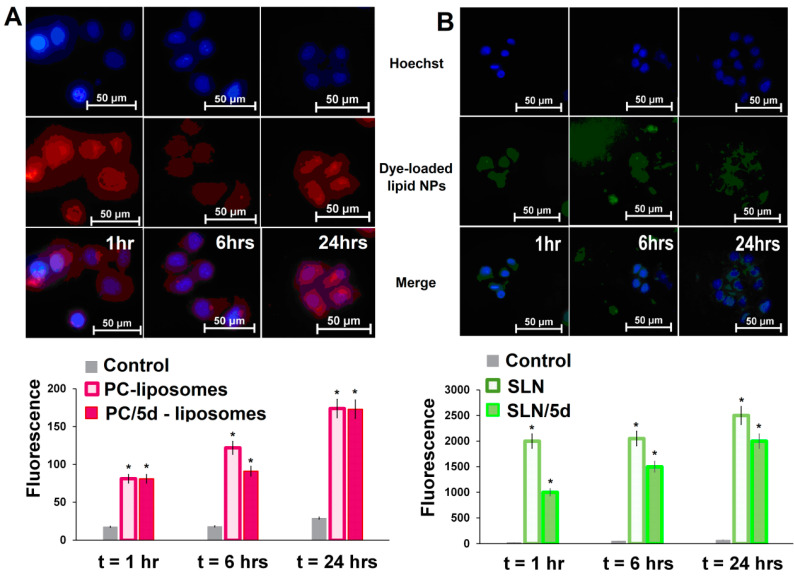
Fluorescence microscopic images (**A**) Rhodamine B labeled-PC/**5d**-liposomes (Nikon eclipse Ci, ×400), (**B**) curcumin labeled SLN/**5d** (Nikon eclipse Ci, ×1000) after incubation time (t = 0, 1, 6 and 24 h), cellular uptake study (flow cytometry) (**A**) Rhodamine B labelled-PC/**5d**-liposomes, (**B**) curcumin labeled SLN/**5d** after incubation time (t = 0, 1, 6 and 24 h); data are presented as mean ± SD of three independent experiments. * Values indicate *p* < 0.01 compared with the control.

Dye-labeled (rhodamine and curcumin) liposomes and SLN, respectively, were prepared to visualize P^+^-lipid nanoparticles and confirm their internalization by cells. The characteristics are presented in Table 2 and Supplementary Material (Figure S168). The monitoring colloid stability of Rhodamine-loaded PC/5d liposomes at 4 °C showed that the size almost did not change, but zeta-potential decreased from +14 mV to –8 mV, which is typical for cationic liposomes [91,92]. As we see in Figure 7, rhodamine-labeled liposomes and curcumin-labeled SLN were located in the cytoplasm after 1 h of incubation with cells. This indicates that the nanoparticles were internalized by tumor cells.

#### 3.2.3. Apoptosis Assay

**Figure 8:**
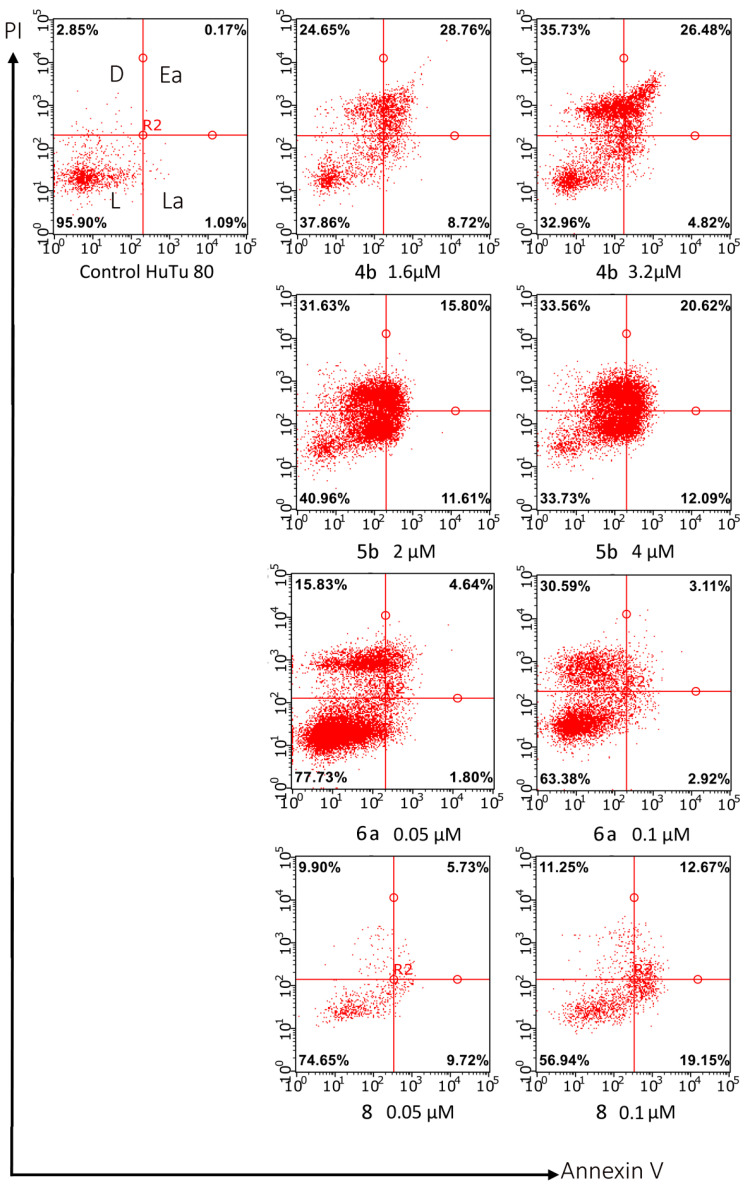
Induction of apoptosis in HuTu 80 cells incubated with compounds **4b**, **5b**, **6a** and **8** at concentration IC_50_/2 and IC_50_; L—living cells; D—dead cells; Ea.—early apoptotic cells; La.—late apoptotic cells.

One important property of cancer cells is their ability to avoid drug-induced apoptosis [94,95]. At the same time, the resistance of cancer cells increases both to conventional antitumor therapy (chemotherapy, radiation therapy) and to alternative therapeutic drugs [96]. Overcoming resistance by reactivating apoptosis is the main direction of the search for promising cancer treatment methods [97,98]. Effective induction of apoptosis using new therapeutic drugs may be an important strategy for preventing cancer recurrence and metastases [99]. The creation of drugs in this field is a difficult task since the process of apoptosis is complicated by various resistance mechanisms and a multifaceted connection with cross-molecular pathways [100]. The main directions of development in recent decades have included the design and synthesis of compounds inhibiting anti-apoptotic and activating pro-apoptotic pathways [101]. For the successful introduction of new molecules, it is necessary to study the fundamental molecular mechanisms of apoptosis and carefully design innovative drugs. In this regard, the apoptosis-inducing properties of various representatives of aminophosphonium salts **4b**, **5b**, **6a,** and **8** with high cytotoxicity and selectivity against the HuTu 80 cancer cell line were investigated. The experiments were carried out by flow cytometry at concentrations of IC_50_/2 and IC_50_ (Figure 8). It can be seen that in HuTu 80 cells, after 48-h incubation in the presence of compounds **4b** and **5b**, dose-dependent apoptosis was observed, with a predominance of apoptotic effects in the late stage. For monoaminophosphonium salt **6a**, the number of necrotic cells prevailed at both concentrations, while for triarylphosphonium salt **8**, the apoptosis-inducing effect was more active in the early stage of apoptosis. The results we obtained indicate the influence of structural modifications of aminophosphonium salts on the process of apoptosis in HuTu 80 cells.

#### 3.2.4. Effect of Aminophosphonium Salts on Mitochondrial Potential

Most forms of apoptosis in mammals are realized not through cell death receptors but through the mitochondrial pathway. In this case, the outer membrane of the mitochondria breaks, and soluble proteins of the intermembrane space enter the cytoplasm. The permeability of the outer membrane of mitochondria is a finely regulated process, and its increase is a key event in triggering apoptosis. Induction of apoptosis by compounds **4b**, **5b**, **6a,** and **8** at concentrations of IC_50_/2 and IC_50_ via the mitochondrial pathway on the HuTu 80 cell line was studied by flow cytometry using the fluorescent dye JC-10 from the Mitochondria Membrane Potential Kit [64].

**Figure 9:**
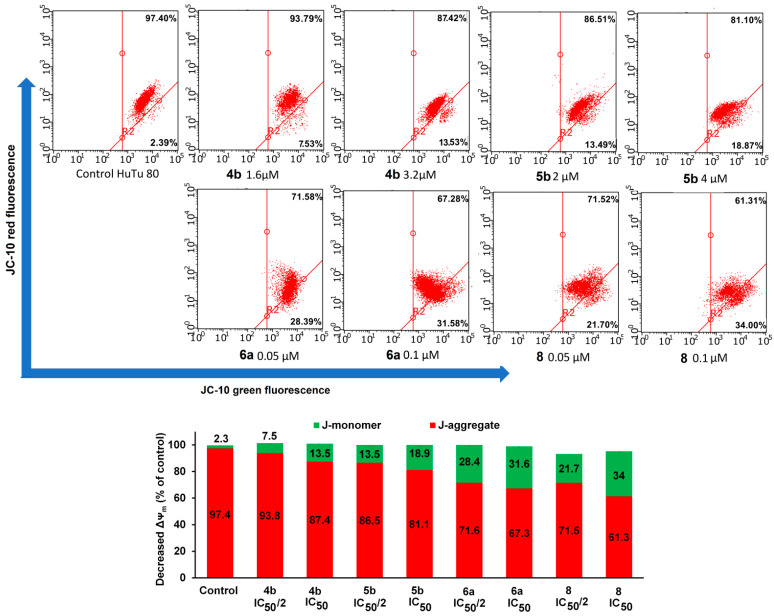
Effects on the mitochondrial membrane potential by **4b**, **5b**, **6a** and **8** at concentrations IC_50_/2 and IC_50_ in HuTu 80 cells.

JC-10 accumulates in the mitochondrial matrix and forms aggregates (J-aggregates) with red fluorescence in normal cells with a high mitochondrial membrane potential. A decrease in the mitochondrial membrane potential of HuTu 80 cells was observed after 48-h treatment with compounds **4b**, **5b**, **6a,** and **8** (Figure 9). Compounds **6a** and **8** have been shown to cause a more significant decrease in the mitochondrial membrane potential in HuTu 80 cells compared with **4b** and **5b**. For all compounds, the effect of membrane depolarization is noticeably enhanced at IC_50_. Thus, the mechanism of cytotoxic action of **4b**, **5b**, **6a,** and **8** may proceed along the internal mitochondrial apoptotic pathway.

#### 3.2.5. Effect of Aminophosphonium Salts on ROS Level in Cancer Cells

**Figure 10:**
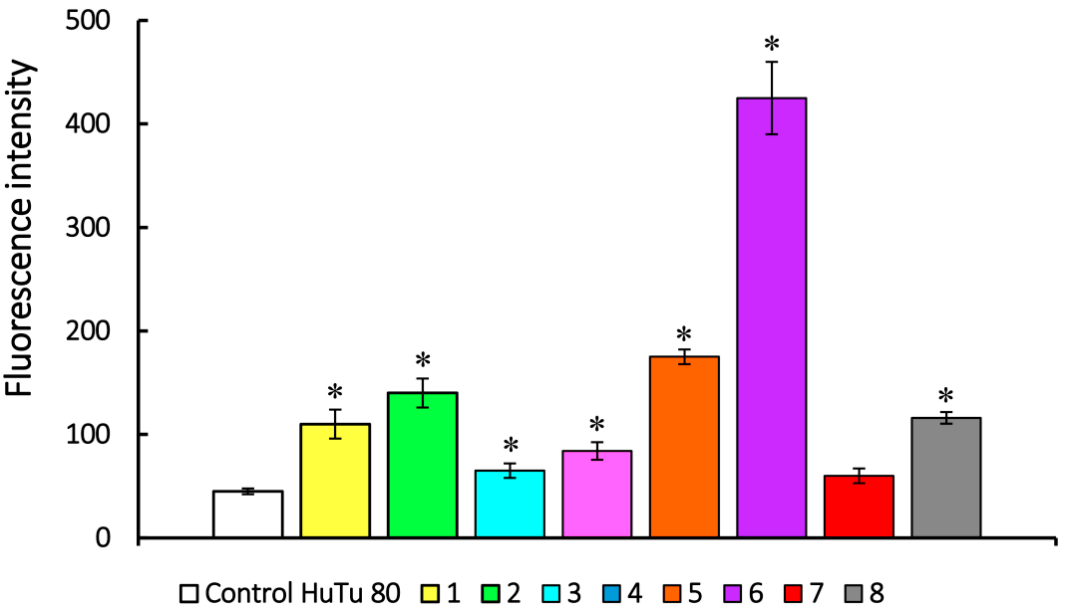
Induction of ROS production by compounds **4b**, **5b**, **6a,** and **8** on HuTu 80 cell line; (1) **4b**—at IC_50_/2 (1.6 μM); (2) **4b**—at IC_50_ (3.2 μM); (3) **5b**—at IC_50_/2 (2μM); (4) **5b**—at IC_50_ (4 μM); (5) **6a**—at IC_50_/2 (0.05 μM); (6) **6a**—at IC_50_ (0.1 μM); (7) **8**—at IC_50_/2 (0.045 μM); (8) **8**—at IC_50_ (0.09 μM). Data are presented as mean ±SD of three independent experiments. * Values indicate *p* < 0.05 compared with the control.

The evaluation of the production of reactive oxygen species (ROS) by the tested compounds complements the data on their effect on the mitochondrial membrane potential. This also illustrates the induction of apoptosis along the mitochondrial pathway. An increase in ROS production leads to mitochondrial dysfunction and, subsequently, cell death. In this regard, the effect of compounds **4b**, **5b**, **6a** and **8** at IC_50_/2 and IC_50_ on ROS production in HuTu 80 cells was investigated using flow cytometry analysis and the CellROX ^®^ Deep Red flow cytometry kit. The data presented in Figure 10 show an increase in the fluorescence intensity of CellROX ^®^ Deep Red after treatment with compounds **4b**, **5b**, **6a,** and **8** at IC_50_/2 and IC_50_ compared with the control (uncolored cells). It can be seen that HuTu 80 cells begin to produce ROS most actively in the presence of **4b** and **6a**. Compounds **5b** and **8** enhance the generation of ROS, mainly in the concentration of IC_50_.

#### 3.2.6. Measurements of Caspase-9 and Caspase-8 by ELISA

To confirm that apoptosis induced by compounds **4b**, **5b**, **6a,** and **8** proceeds along the internal (mitochondrial) pathway, studies were conducted to determine the enzymatic activity of the early apoptosis key markers—caspase 9 and caspase 8 using ELISA kits. The kits are sandwich enzyme immunoassays for in vitro quantitative measurement of Caspase 8 and Caspase 9 in cell lysates, rat tissue homogenates, and other biological fluids. Caspase 9 plays a key role in the mitochondrial signaling pathway of apoptosis induction. The activation of caspase 8 characterizes the external (receptor-dependent) pathway of apoptosis. HuTu 80 cells not treated with the tested compounds were used as a control.

**Figure 11:**
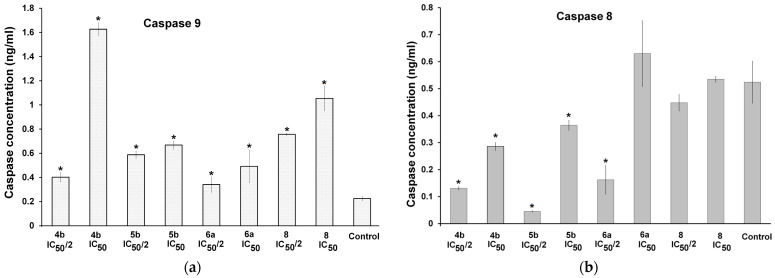
Quantitative measurement of caspase for the HuTu 80 cell lysates in vitro. (**a**)—measurement of caspase 9 after treatment at **4b**, **5b**, **6a,** and **8** at IC_50_/2 and IC_50_; (**b**)—measurement of caspase 8 after treatment at **4b**, **5b**, **6a,** and **8** at IC_50_/2 and IC_50_; data are presented as mean ± SD of three independent experiments. * Values indicate *p* < 0.05.

We calculated the concentration of caspase 9 and caspase 8 in ng/mL in experimental and control samples. The results are shown in Figure 11. The concentration of caspase 9 in samples treated with compounds **4b**, **5b**, **6a,** and **8** at IC_50_/2 and IC_50_ (Figure 11A) significantly increased compared with the control. The lowest concentration of caspase 9 is shown for compound **6a**, which is consistent with the data on the assessment of apoptosis by flow cytometry (Figure 8). The concentration of caspase 8 significantly decreased relative to the control after treatment with substances **4b** and **5b** (Figure 11B). Thus, the studied compounds have a cytotoxic effect due to the induction of apoptosis proceeding along the mitochondrial pathway.

#### 3.2.7. Effect of Aminophosphonium Salts on Cell Cycle in Cancer Cells

**Figure 12:**
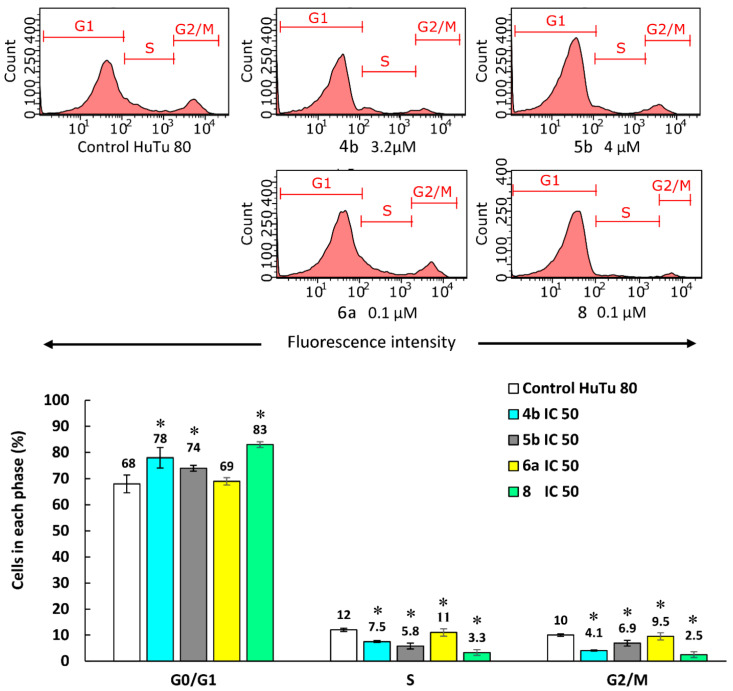
Effect of compounds **4b**, **5b**, **6a,** and **8** at concentrations IC_50_ in HuTu 80 cells. Percentage of cells in the G0/G1, S, and G2/M phases (data are presented as mean ± SD of three independent experiments). * Values indicate *p* < 0.05.

The drugs that induce ROS and mitochondrial apoptosis in cancer cells cause disruption of cell cycle phases and proliferation [102,103]. Therefore, the effect of **4b**, **5b**, **6a,** and **8** on the passage of HuTu 80 cells through the cell cycle was investigated using the classical fluorescent method, which allows determining at which phase the cell cycle was stopped. The studies were carried out using a fluorescent dye, propidium iodide, which binds in proportion to the amount of DNA present in the cell. The diagram in Figure 12 shows the number of cells in each phase of the cell cycle. Analysis of the HuTu 80 cell cycle after treatment with compounds at IC_50_ for 48 h revealed a significant delay of cells in the G0/G1 phase for **4b**, **5b,** and **8** compared with the control. Compound **6a** caused a slight change in the cell cycle.

#### 3.2.8. Antibacterial Activity of Aminophosphonium Salts

Drugs with both antitumor and antimicrobial effects are currently actively used for malignant tumors of various origins [104]. In this regard, we studied the antibacterial and antifungal activity of the newly obtained compounds, including against resistant strains of microorganisms that pose a serious threat to human health around the world.

The synthesized compounds were tested for antibacterial (bacteriostatic and bactericidal) activity against a number of Gram-positive *Staphiloccus aureus* 209P (*Sa*), *Bacillus cereus* 8035 (*Bc*), and Gram-negative bacteria *Echerishia coli* F-50 (*Ec*), and *Pseudomonas aeruginosa* 9027 (*Pa*), including against methicillin-resistant strains of *S. aureus* (*MRSA-1, MRSA-2*). Antifungal activity was studied on a culture of *Candida albican*s 10231 (*Ca*). The data are presented in Table 4.

The tested compounds showed selective activity against all Gram-positive bacteria, including MRSA strains. Most aminophosphonium salts showed antimicrobial action at the level of the fluoroquinolone antibiotic norfloxacin, and in some cases, they even significantly exceeded the activity of this comparison drug. The highest antimicrobial activity against all Gram-positive and Gram-negative bacteria and against *Candida albicans* 10231 was found for compound **4e**. Moreover, it acted bactericidal and fungicidal, i.e., MIC and MBC differ from each other by no more than 4 times.

#### 3.2.9. Hemolytic Action of Test Compounds

The hemolytic activity of chemical compounds can be used as a means for their toxicological assessment. In this regard, a concentration (HC_50_, μM) was determined for compounds **4b**, **5b,** and **6a**, causing hemolysis in 50% of erythrocytes. Data on the hemolytic activity of compounds **4b**, **5b,** and **6a** are given in Table 5. The HC_50_ values of these compounds are > 100 μM. Gramicidin C was used as a comparison drug. Results indicate the high selectivity of the tested compounds with respect to bacterial cells and low toxicity with respect to human blood cells, which may be of interest for further studies on living objects.

#### 3.2.10. In Vivo Study of Acute Toxicity of Aminophosphonic Salts

The study of the acute toxicity parameters of six aminophosphonium salts **4a**, **4b**, **4d**, **5a**, **5b,** and **6b** was performed on male outbreed white mice of the CD-1 line. Acute toxicity was determined with a single parenteral (intraperitoneal) administration in sterile saline/DMSO (40% vol.) solution at doses from 1 to 100 mg/kg. To assess acute toxicity, the animals were monitored for 14 days after the introduction of substances, assessing their general condition and recording deaths. Next, lethal doses LD_50_, LD_0_, LD_16_, LD_84_, and LD_100_ were calculated using the Probit analysis method. Calculated LD_50_ values are reported in Table 6. LD_0_, LD_16_, LD_84,_ and LD_100_ are given in Supplementary Material (Table S1).

In the control group of animals that were injected with a solvent (sterile saline/DMSO (40% vol.) in an amount of 0.1 mL/10 g (10 mL/kg), there was no death or signs of depression in the animals. Among the studied substances, **4d** (LD_50_ 6.9 mg/kg) has the highest toxicity. According to the classification [105], **4d** belongs to the class of highly toxic compounds. LD_50_ of **4a**, **4b**, **5a**, **5b,** and **6b** are in the range of 37.5 to 106.5 mg/kg. They can be classified as moderately toxic compounds. It should be noted the similarity in the manifestation of the toxic effect for **4a**, **4b**, **4d**, **5a,** and **5b**, namely, the rapid onset of animal death within 1–2 h after administration in doses of LD_100_. **6b** administration at a dose of 150 mg/kg causes 100% death in animals. Deaths were observed a day after administration. Administration of **6b** at higher doses of 200 and 400 mg/kg caused death within 10 min. Deaths of animals were observed after 1–12 days at lower doses (LD16-50). Observed symptoms as convulsions, rapid heartbeat, heavy breathing indicate effects on central and peripheral nervous systems.

## 4. Conclusions

In this work, the synthesis of various amphiphilic phosphonium salts containing a diethylamino-groups was carried out by the interaction of hexaethyltriaminophosphine **1**, bis(tetraethyldiamino)phenylphosphine **2,** and diethylaminodiphenylphosphine **3** with alkyl iodides and tetradecyl bromide under mild conditions in acetonitrile. The structure of the obtained salts was proved by NMR and XRD for (diethylamino)(octyl)diphenylphosphonium iodide **6a**. In vitro cytotoxicity and in vivo toxicity were evaluated. Nanotherapeutic forms based on lipids (liposomes and solid lipid nanoparticles) and amphiphilic phosphonium salts were developed. The zeta potential of lipid nanosystems decorated by amphiphilic aminophosphonic compounds shifted to positive values. An increase in the size (from 100 to 150 nm) and polydispersity index (from 0.24 to 0.47) of solid lipid nanoparticles were observed. The internalization of dye-labeled liposomes and solid lipid nanoparticles by tumor cells within 1 h was shown by flow cytometry. Most of the compounds exhibited high cytotoxicity towards the M-HeLa cell lines: the IC_50_ is in the range of 0.24–1.8 μM with a selectivity index (SI) of 1.6–17. The highest SI was found for **4d** (SI 7.1), **6a** (SI 4.8), and **6b** (SI 17). Among them, **6b** is a leader with a two-fold lower IC_50_ than the reference drug doxorubicin. A pronounced dependence of increasing SI on the nature of aminophosphonium cation was observed: **4** < **5** < **6** at a rather low IC_50_ of 0.06–4.0 μM for **4**–**6** on the HuTu 80 cell lines. The highest SI was for **4e** (SI 28), **5a** (SI 210), and **6a** (SI 277). A similar behavior was found for PC3, Du-145, and PANC-1 cell lines, where monoaminophosphonium compounds are the most effective with IC_50_ (SI) values in the range 0.3–5.5 μM (6.4–20). Monoaminophosphonium derivatives **6** are more cytotoxic with a higher SI compared with tri- and diaminophosphonium salts **4** and **5**. Cytotoxicity increases with the length of the R substituent at the phosphorus atom in the HuTu 80, PC3, and Du-145 cell lines. The lipid systems with aminophosphonium salts provide an improvement in cytotoxic activity against tumor cells compared with their individual compounds and a decrease in toxicity against normal cell lines. The IC_50_ of PC/**4c**, PC/**4d**, PC/**5b**, PC/**5c**, PC/**5d,** and SLN/**4b** lipid systems are lower than for the reference drug DOX, with high SI 53 and 56 and SI 30 for the SLN/**4b** and PC/**4c** toward MCF-7 and DU-145. The mechanism of cytotoxicity of **4b**, **5b**, **6a,** and **8** appears to involve the intrinsic mitochondrial apoptotic pathway. This is also consistent with the data on the production of ROS and the enzymatic activity of early apoptosis key markers—caspase 9 and caspase 8—as well as the effect on the phases of cell cycle and proliferation. Synthesized aminophosphonium salts exhibit high selective activity against Gram-positive bacteria *S. aureus* 209P, *B. segeus* 8035, including methicillin-resistant strains of *S. aureus* (*MRSA-1*, *MRSA-2*), comparable to the reference fluoroquinolone antibiotic norfloxacin. A leader tetradecyl-tris(diethylamino)phosphonium bromide **4e** shows the highest antimicrobial activity against all studied Gram-positive and Gram-negative bacteria, as well as against *Candida albicans* 10231 fungi. **4b**, **5b,** and **6a,** with their high cytotoxicity, demonstrated their low toxicity to human blood cells. A moderate in vivo toxicity in CD-1 mice was established for the lead compounds. This indicates the interest of these compounds for further research on cellular organelles.

## Figures and Tables

**Table 1:** Principal crystallographic parameters of compound **6a** based on X-ray diffraction data.

Parameter	6a	Parameter	6a
Molecular formula	C_22_H_33_NP, I	Ranges of indices	
Sum Formula	C_22_H_33_INP	*h*	–13 ≤ *h* ≤ 13
Molecular weight	469.36	*k*	–14 ≤ *k* ≤ 14
Crystal system	triclinic	*l*	–20 ≤ *l* ≤ 20
Space group	P-1 (No. 2)	Number of total reflections	119,684
*Z*	2	Independent reflections	7633
Unit cell parameters		*R_int_*	0.069
*a*/Å	9.3261(5)	Completeness up to θ = 28.0°	0.994
*b*/Å	9.9546(5)	T_max/min_	0.7456/0.5131
*c*/Å	13.5060(7)	Number of observed reflections (*I* > 2σ(*I*))	7098
*α*	69.106(2)	Number of reflections/of contraints/number of parameters	7633/0/229
*β*/deg	71.494(2)	GOOF	1.068
*γ*	80.251(2)	*R* [*I* > 2σ(*I*)]	
*V*/Å^3^	1108.61(10)	*R*_1_	0.0213
*d*_calc_/g cm^–3^	1.406	*wR*_2_	0.0553
Absorption coefficient, μ/mm^–1^	1.521	*R* (based on all reflections)	
*F*(000)	480	*R*_1_ and *wR*_2_	0.0237 and 0.0561
*Θ* (min, max)/deg	2.2, 32.0	Residual electron density (ρ_max_/ρ_min_)/e Å^–3^	1.14/–0.50

**Table 2:** Characteristics of lipid systems PC-liposomes and solid lipid nanoparticles (SLN), average particle size (Z_average_, nm), polydispersity index (PDI), Zeta potential (ξ, mV), 25 °C.

Composition	Ratio, (% *w*/*w*)	Z_average,_ nm	PDI	ξ, mV
PC [75,76]	100	119 ± 2	0.12 ± 0.02	−7.0 ± 2
PC/**4a**	99.8/0.2	121 ± 0.5	0.09 ± 0.01	−5.1 ± 0.2
PC/**4b**	99.8/0.2	124 ± 1	0.07 ± 0.01	+4.3 ± 0.4
PC/**4c**	99.8/0.2	125 ± 1	0.14 ± 0.02	+9.3 ± 0.3
PC/**4d**	99.8/0.2	134 ± 1	0.14 ± 0.01	+13.9 ± 0.4
PC/**4e**	99.8/0.2	115 ± 1	0.12 ± 0.01	+26.8 ± 3
PC/**5b**	99.8/0.2	129 ± 1	0.14 ± 0.01	+3.2 ± 0.3
PC/**5c**	99.8/0.2	116 ± 2	0.11 ± 0.01	+7.1 ± 0.2
PC/**5d**	99.8/0.2	126 ± 0.5	0.14 ± 0.02	+13.3 ± 0.5
PC/**4d**-Rhod	99.8/0.2	122 ± 0.2	0.11 ± 0.01	+19.0 ± 1.7
PC/**5b**-Rhod	99.8/0.2	116 ± 0.5	0.10 ± 0.02	+7.9 ± 0.2
PC/**5c**-Rhod	99.8/0.2	118 ± 0.3	0.09 ± 0.03	+2.6 ± 0.3
PC/**5d**-Rhod	99.8/0.2	117 ± 0.4	0.1 ± 0.01	+14.2 ± 0.6
PC/**5d**-Rhod *	99.8/0.2	115 ± 0.2	0.1 ± 0.01	−8.4 ± 0.6
PC/**8**	99.8/0.2	117 ± 0.5	0.12 ± 0.02	+16.3 ± 2.5
PC/**7**	99.8/0.2	124 ± 0.1	0.15 ± 0.01	−11.7 ± 1.6
SLN [65]	100	104 ± 1	0.24 ± 0.01	−17.2 ± 1
SLN/**4b**	99.3/0.7	108 ± 0.3	0.31 ± 0.04	−15.6 ± 0.5
SLN/**5b**	99.3/0.7	114 ± 1	0.24 ± 0.01	−15.8 ± 0.7
SLN/**5c**	99.3/0.7	132 ± 2	0.32 ± 0.03	−7.7 ± 0.5
SLN/**5d**	99.3/0.7	147 ± 2	0.47 ± 0.02	−3.6 ± 0.1
SLN-Cur	100	125 ± 1	0.2 ± 0.01	−28.0 ± 1
SLN/**5d**-Cur	99.8/0.2	123 ± 1	0.21 ± 0.01	−18.5 ± 1

* 6-month storage at 4 °C.

**Table 3:** Cytotoxic Effects (IC_50_, μM) and selectivity index values (SI) of aminophosphonium salts and their lipid formulations (liposomes and SLN) (MTT assay, 48 h).

Test Compounds	Cancer Cell Lines										Normal Cell Lines	
	M-HeLa	MCF-7	HuTu 80	PANC-1	A549	PC3	T98G	Hep G2	SK-OV-3	DU-145	WI38	Chang Liver
**4a**	52.4 ± 3.7 (ns)	26.0 ± 1.8 (ns)	11.4 ± 0.9 (1.6 ^b^)	16.1 ± 1.3 (1.1 ^b^)	64.1 ± 4.5 (ns)	44.0 ± 3.1 (ns)	57.4 ± 4.0 (ns)	46.6 ± 3.3 (ns)	46.6 ± 3.5 (ns)	26.2 ± 1.9 (ns)	7.6 ± 0.6	17.7 ± 1.5
PC/**4a**	60 ± 12 (ns)	28.3 ± 2.0 (ns)	26.1 ± 1.8 (ns)	24.6 ± 1.6 (ns)	29.2 ± 1.9 (ns)	15.5 ± 1.2 (ns)	37.3 ± 2.8 (ns)	29.1 ± 2.3 (ns)	35.5 ± 2.5 (ns)	68.5 ± 4.1 (ns)	17.2 ± 2.4	6.5 ± 0.8
**4b**	1.8 ± 0.1 (2.2 ^b^)	2.9 ± 0.2 (1.4 ^b^)	3.2 ± 0.2 (1.3 ^b^)	2.3 ± 0.1 (1.7 ^b^)	15.4 ± 1.2 (ns)	11.6 ± 0.9 (ns)	5.9 ± 0.4 (ns)	2.9 ± 0.2 (1.4 ^b^)	26.3 ± 1.8 (ns)	4.2 ± 0.3 (ns)	0.8 ± 0.06	4.0 ± 0.3
PC/**4b**	4.5 ± 0.3 (2.8 ^b^)	4.1 ± 0.3 (3.1 ^b^)	0.7 ± 0.06 (18 ^b^)	0.9 ± 0.08 (14 ^b^)	9.5 ± 0.8 (1.3 ^b^)	3.5 ± 0.4 (3.6 ^b^)	2.2 ± 0.1 (5.7 ^b^)	5.0 ± 0.4 (2.5 ^b^)	83.0 ± 5.8 (ns)	1.6 ± 0.1 (8.0 ^b^)	2.6 ± 0.1	12.6 ± 1.2
SLN/**4b**	0.42 ± 0.04 (26 ^b^)	0.21 ± 0.02 (53 ^b^)	0.42 ± 0.04 (26 ^b^)	1.1 ± 0.1 (10 ^b^)	1.5 ± 0.1 (7.4 ^b^)	0.3 ± 0.03 (37 ^b^)	0.6 ± 0.06 (19 ^b^)	0.3 ± 0.03 (37 ^b^)	75 ± 6 (ns)	0.2 ± 0.01 (56 ^b^)	2.1 ± 0.2	11.1 ± 1.2
**4c**	1.7 ± 0.1 (2.8 ^b^)	1.9 ± 0.1 (2.5 ^b^)	2.6 ± 0.2 (1.8 ^b^)	1.7 ± 0.1 (2.8 ^b^)	21.1 ± 1.5 (ns)	1.8 ± 0.1 (2.7 ^b^)	3.2 ± 0.2 (1.5 ^b^)	2.4 ± 0.1 (2.0 ^b^)	12.1 ± 1.1 (ns)	1.3 ± 0.1 (ns)	0.8 ± 0.07	4.8 ± 0.4
PC/**4c**	1.1 ± 0.09 (ns)	0.3 ± 0.02 (30 ^b^)	0.6 ± 0.05 (15.6 ^b^)	0.7 ± 0.06 (13 ^b^)	4.2 ± 0.3 (2.2 ^b^)	1.1 ± 0.08 (8.3 ^b^)	1.9 ± 0.1 (4.8 ^b^)	1.1 ± 0.08 (8.3 ^b^)	99 ± 7 (ns)	0.4 ± 0.03 (22.8 ^b^)	2.6 ± 0.2	9.1 ± 0.8
**4d**	1.7 ± 0.1 (7.1 ^b^)	2.1 ± 0.2 (5.7 ^b^)	2.8 ± 0.2 (4.3 ^b^)	1.6 ± 0.1 (7.5 ^b^)	18.4 ± 1.3 (ns)	7.3 ± 0.6 (1.6 ^b^)	1.8 ± 0.2 (6.7 ^b^)	2.3 ± 0.1 (5.2 ^b^)	10.0 ± 0.9 (1.2 ^b^)	1.2 ± 0.1 (10 ^b^)	0.9 ± 0.08	12.0 ± 0.9
PC/**4d**	0.7 ± 0.06 (2.6 ^a^)	0.4 ± 0.03 (4.5 ^a^)	0.24 ± 0.02 (7.5 ^a^)	0.26 ± 0.02 (7.0 ^a^)	3.7 ± 0.3 (ns)	0.9 ± 0.08 (2.0 ^a^)	0.9 ± 0.07 (2.0 ^a^)	0.6 ± 0.05 (3.0 ^a^)	69.4 ± 4.9 (ns)	0.15 ± 0.01 (12 ^a^)	1.8 ± 0.1	0.7 ± 0.06
**4e**	0.4 ± 0.03 (4.3 ^b^)	0.9 ± 0.07 (1.9 ^b^)	0.06 ± 0.005 (28 ^b^)	0.4 ± 0.03 (4.3 ^b^)	1.7 ± 0.1 (1.0 ^b^)	1.7 ± 0.1 (1.0 ^b^)	1.0 ± 0.09 (1.7 ^b^)	1.5 ± 0.1 (1.1 ^b^)	1.7 ± 0.1 (1.0 ^b^)	0.4 ± 0.03 (4.3 ^b^)	1.2 ± 0.1	1.7 ± 0.1
PC/**4e**	62 ± 5.3 (ns)	1.5 ± 0.8 (24 ^b^)	29.7 ± 2.4 (1.2 ^b^)	2.5 ± 0.3 (14 ^b^)	2.3 ± 0.1 (16 ^b^)	3.8 ± 0.3 (9.5 ^b^)	3.2 ± 0.2 (11 ^b^)	7.9 ± 0.6 (4.5 ^b^)	35.5 ± 2.8 (1 ^b^)	38.5 ± 3.2 (ns)	7.1 ± 0.7	36.0 ± 2.9
**5a**	23.0 ± 1.6 (2.7 ^a^)	17.1 ± 1.3 (3.7 ^a^)	0.3 ± 0.02 (210 ^a^)	17.8 ± 1.4 (3.5 ^a^)	17.0 ± 1.3 (3.7 ^a^)	4.9 ± 0.4 (13 ^a^)	30.3 ± 2.1 (3.7 ^a^)	16.5 ± 1.3 (3.8 ^a^)	20.0 ± 1.6 (3.2 ^a^)	6.3 ± 0.5 (10 ^a^)	63.0 ± 4.4	22.6 ± 1.6
**5b**	1.8 ± 0.1 (2.2 ^b^)	2.6 ± 0.2 (1.5 ^b^)	4.0 ± 0.3 (1.0 ^b^)	1.2 ± 0.1 (3.3 ^b^)	20.0 ± 1.6 (ns)	11.4 ± 0.9 (ns)	4.3 ± 0.3 (1.0 ^b^)	2.9 ± 0.3 (1.4 ^b^)	25.6 ± 1.8 (ns)	2.0 ± 0.1 (2.0 ^b^)	0.5 ± 0.4	4.0 ± 0.3
PC/**5b**	1.1 ± 0.08 (5.5 ^a^)	0.7 ± 0.06 (8.6 ^a^)	0.22 ± 0.01 (5.5 ^a^)	0.7 ± 0.06 (8.6 ^a^)	3.0 ± 0.2 (2.0 ^a^)	1.4 ± 0.1 (4.3 ^a^)	3.4 ± 0.2 (1.8 ^a^)	1.0 ± 0.09 (6.0 ^a^)	1.8 ± 0.1 (3.3 ^a^)	0.4 ± 0.03 (15 ^a^)	6.0 ± 0.5	4.6 ± 0.4
SLP/**5b**	2.0 ± 0.1 (1.8 ^a^)	1.3 ± 0.1 (2.8 ^a^)	0.9 ± 0.07 (4.0 ^a^)	1.2 ± 0.1 (3.0 ^a^)	2.5 ± 0.2 (1.4 ^a^)	1.6 ± 0.1 (2.3 ^a^)	5.0 ± 0.4 (ns)	10.0 ± 0.9 (ns)	1.7 ± 0.1 (2.1 ^a^)	1.1 ± 0.09 (3.3 ^a^)	3.6 ± 0.2	1.3 ± 0.1
**5c**	1.7 ± 0.1 (2.4 ^b^)	3.1 ± 0.2 (1.3 ^b^)	2.8 ± 0.3 (1.5 ^b^)	2.8 ± 0.2 (1.5 ^b^)	8.7 ± 0.7 (ns)	10.0 ± 0.9 (ns)	2.7 ± 0.3 (1.5 ^b^)	2.3 ± 0.1 (1.8 ^b^)	14.8 ± 1.2 (ns)	0.9 ± 0.07 (4.6 ^b^)	0.5 ± 0.4	4.1 ± 0.3
PC/**5c**	0.7 ± 0.06 (3.3 ^b^)	0.5 ± 0.04 (4.6 ^b^)	0.4 ± 0.03 (5.8 ^b^)	0.8 ± 0.06 (2.9 ^b^)	1.6 ± 0.1 (1.4 ^b^)	0.6 ± 0.05 (3.8 ^b^)	2.7 ± 0.2 (ns)	0.6 ± 0.05 (3.8 ^b^)	83.1 ± 5.8 (ns)	0.2 ± 0.01 (12 ^b^)	1.9 ± 0.1	2.3 ± 0.1
**5d**	1.5 ± 0.1 (ns)	0.7 ± 0.06 (1.9 ^b^)	2.5 ± 0.1 (ns)	1.4 ± 0.1 (ns)	8.9 ± 0.7 (ns)	0.8 ± 0.06 (1.6 ^b^)	0.7 ± 0.05 (1.9 ^b^)	1.1 ± 0.09 (1.2 ^b^)	10.0 ± 0.8 (ns)	0.45 ± 0.03 (2.9 ^b^)	0.3 ± 0.02	1.3 ± 0.1
PC/**5d**	0.8 ± 0.07 (1.9 ^a^)	0.7 ± 0.06 (2.1 ^a^)	0.4 ± 0.02 (3.8 ^a^)	0.5 ± 0.04 (3.0 ^a^)	2.6 ± 0.2 (ns)	0.6 ± 0.05 (2.5 ^a^)	2.5 ± 0.2 (ns)	2.7 ± 0.2 (ns)	92.4 ± 6.5 (ns)	0.3 ± 0.02 (5.0 ^a^)	1.5 ± 0.1	0.23 ± 0.01
**6a**	5.8 ± 0.4 (4.8 ^a^)	17.0 ± 1.3 (1.6 ^a^)	0.1 ± 0.007 (277 ^a^)	5.5 ± 0.4 (5.0 ^a^)	48.2 ± 3.4 (ns)	4.0 ± 0.3 (6.9 ^a^)	18.4 ± 1.5 (1.5 ^a^)	34.4 ± 2.4 (ns)	36.8 ± 2.6 (ns)	2.6 ± 0.3 (11 ^a^)	27.7 ± 1.9	15.2 ± 1.3
**6b**	0.95 ± 0.08 (17 ^a^)	9.5 ± 0.8 (1.7 ^a^)	0.2 ± 0.01 (81 ^a^)	1.3 ± 0.1 (12 ^a^)	13.0 ± 1.1 (1.2 ^a^)	0.85 ± 0.07 (19 ^a^)	3.4 ± 0.2 (5.0 ^a^)	11.6 ± 0.9 (1.4 ^a^)	17.6 ± 1.4 (ns)	0.8 ± 0.07 (20 ^a^)	16.1 ± 1.2	0.42 ± 0.03
**6c**	0.9 ± 0.07 (1.6 ^a^)	4.2 ± 0.3 (2.0 ^a^)	0.2 ± 0.01 (42 ^a^)	1.3 ± 0.1 (6.4 ^a^)	11.4 ± 0.9 (ns)	1.0 ± 0.08 (8.3 ^a^)	2.4 ± 0.1 (3.5 ^a^)	6.2 ± 0.5 (1.3 ^a^)	12.7 ± 1.1 (ns)	1.3 ± 0.1 (6.4 ^a^)	8.3 ± 0.7	0.5 ± 0.04
**6d**	0.24 ± 0.02 (1.6 ^a^)	1.6 ± 0.1 (ns)	0.35 ± 0.02 (1.1 ^a^)	0.4 ± 0.03 (1.0 ^a^)	8.2 ± 0.7 (ns)	1.4 ± 0.1 (ns)	0.6 ± 0.05 (ns)	1.3 ± 0.1 (ns)	3.9 ± 0.4 (ns)	0.43 ± 0.03 (1.0 ^a^)	0.4 ± 0.03	0.3 ± 0.02
**6e**	0.4 ± 0.03 (7.0 ^a^)	1.2 ± 0.1 (2.3 ^a^)	0.07 ± 0.005 (40 ^a^)	0.3 ± 0.02 (9.3 ^a^)	2.1 ± 0.1 (1.3 ^a^)	1.5 ± 0.1 (1.9 ^a^)	1.1 ± 0.09 (2.5 ^a^)	1.4 ± 0.1 (2.0 ^a^)	1.6 ± 0.1 (1.8 ^a^)	0.4 ± 0.02 (7.0 ^a^)	2.8 ± 0.2	2.5 ± 0.2
**7**	16.1 ± 1.3 (3.0 ^a^)	29.1 ± 2.0 (1.7 ^a^)	1.9 ± 0.2 (26 ^a^)	44.6 ± 3.1 (1.1 ^a^)	>100 (ns)	32.7 ± 2.3 (1.5 ^a^)	>100 (ns)	>100 (ns)	69.0 ± 4.8 (ns)	58.0 ± 4.1 (ns)	49.0 ± 3.4	34.0 ± 2.4
PC/**7**	148 ± 12 (ns)	67 ± 5.4 (ns)	54.3 ± 3.8 (ns)	134.2 ± 10 (ns)	>100 (ns)	41.7 ± (ns)	>100 (ns)	>100 (ns)	>100 (ns)	99.2 ± (ns)	12.6 ± 1.3	0.3 ± 0.02
**8**	0.45 ± 0.04 (20 ^a^)	4.2 ± 0.3 (2.2 ^a^)	0.09 ± 0.006 (102 ^a^)	0.3 ± 0.02 (31 ^a^)	8.7 ± 0.7 (1.1 ^a^)	0.4 ± 0.03 (23 ^a^)	0.6 ± 0.05 (15 ^a^)	1.6 ± 0.1 (5.8 ^a^)	9.8 ± 0.7 (ns)	0.4 ± 0.03 (23 ^a^)	9.2 ± 0.8	3.0 ± 0.2
PC/**8**	17.0 ± 1.5 (1.7 ^b^)	11.2 ± 0.9 (2.6 ^b^)	6.2 ± 0.5 (4.7 ^b^)	18.2 ± 1.4 (1.6 ^b^)	0.8 ± 0.06 (36 ^b^)	35.6 ± 2.2 (ns)	1.2 ± 0.1 (24 ^b^)	37.2 ± 2.5 (ns)	67.0 ± 4.7 (ns)	2.5 ± 0.1 (12 ^b^)	28.0 ± 1.8	29.0 ± 2.0
**9**	31.1 ± 3.9 (1.5 ^a^)	37.8 ± 4.8 (1.2 ^a^)	13.1 ± 1.7 (3.5 ^a^)	39.6 ± 5.1 (1.2 ^a^)	>100	34.9 ± 4.5 (1.3 ^a^)	>100	53.9 ± 6.9 (ns)	36.4 ± 4.7 (1.3 ^a^)	50.5 ± 6.4 (ns)	45.8 ± 5.9	37.2 ± 4.8
doxorubicin	2.1 ± 0.1 (ns)	0.4 ± 0.03 (1.3 ^b^)	0.2 ± 0.01 (2.5 ^b^)	2.2 ± 0.1 (ns)	0.7 ± 0.06 (ns)	1.4 ± 0.1 (ns)	1.0 ± 0.09 (ns)	0.2 ± 0.01 (2.5 ^b^)	6.7 ± 0.5 (ns)	0.3 ± 0.02 (1.7 ^b^)	0.4 ± 0.03	0.5 ± 0.03

The experiments were performed in triplicate. Data for the most active compounds and lead compounds are highlighted in color and depth. Results are expressed as the mean ± standard deviation (SD); ns—no selectivity. ^a^ The selectivity index was calculated relative to the cell line WI38. ^b^ The selectivity index was calculated relative to the cell line Chang liver.

**Table 4:** Antimicrobial activity of aminophosphonium salts **4**–**6**.

Compounds	Minimum Inhibitory Concentration (MIC), μM							
	Gram-Positive Bacteria					Gram-Negative Bacteria		Yeast
	*Sa*	*Bc*	*Ef*	*MRSA-1*	*MRSA-2*	*Ec*	*Pa*	*Ca*
**4a**	62.5 ± 5.2	62.5 ± 5.3	250 ± 21	125 ± 10	125 ± 11	–	–	–
**4b**	15.6 ± 1.3	7.8 ± 0.7	250 ± 19	15.6 ± 0.3	15.6 ± 1.2	–	–	–
**4c**	3.9 ± 0.3	3.9 ± 0.3	62.5 ± 5.2	3.9 ± 0.4	7.8 ± 0.6	500 ± 48	–	–
**4d**	1.9 ± 0.1	0.9 ± 0.07	15.6 ± 1.3	0.9 ± 0.07	1.9 ± 0.1	125 ± 9	500 ± 47	125 ± 11
**4e**	0.25 ± 0.01	0.5 ± 0.04	0.5 ± 0.04	0.5 ± 0.04	0.25 ± 0.02	15.6 ± 1.3	7.8 ± 0.6	1.9 ± 0.1
**9**	34.3 ± 5.4	274 ± 48	–	137 ± 19	34.3 ± 5.3	–	–	–
**5b**	7.8 ± 0.6	7.8 ± 0.6	125 ± 11	15.6 ± 1.2	15.6 ± 1.2	–	500 ± 46	–
**5c**	3.9 ± 0.3	3.9 ± 0.3	31.3 ± 2.2	3.9 ± 0.3	3.9 ± 0.4	250 ± 19	–	–
**5d**	0.9 ± 0.07	0.9 ± 0.08	15.6 ± 1.2	0.9 ± 0.08	0.9 ± 0.08	62.5 ± 5.4	500 ± 48	250 ± 21
**6a**	62.5 ± 5.5	31.3 ± 2.3	125 ± 11	62.5 ± 5.3	62.5 ± 5.6	–	–	–
**6b**	7.8 ± 0.6	3.9 ± 0.4	62.5 ± 5.5	7.8 ± 0.6	7.8 ± 0.6	–	–	–
**6c**	3.9 ± 0.3	1.9 ± 0.1	31.3 ± 5.4	3.9 ± 0.3	3.9 ± 0.4	500 ± 46	250 ± 21	500 ± 47
**6d**	0.9 ± 0.08	0.9 ± 0.08	3.9 ± 0.3	0.5 ± 0.03	0.5 ± 0.03	62.5 ± 5.3	125 ± 10	62.5 ± 5.4
**6e**	0.5 ± 0.04	0.9 ± 0.08	0.5 ± 0.03	0.5 ± 0.04	0.5 ± 0.03	31.3 ± 2.4	3.9 ± 0.3	1.9 ± 0.1
**8**	0.9 ± 0.07	0.9 ± 0.07	15.6 ± 1.2	1.9 ± 0.1	3.9 ± 0.3	500 ± 47	–	–
**7**	250 ± 20	500 ± 47	–	250 ± 19	250 ± 20	–	–	–
Norfloxacin	7.5 ± 0.5	24.4 ± 2.1	24.4 ± 2.2	–	7.5 ± 0.5	4.7 ± 0.02	12.1 ± 1.1	–
Ketoconazole	–	–	–	–	–	–	–	7.3 ± 0.5
Minimum bactericidal and fungicidal concentrations (MBC, MFC), μM								
**4a**	250 ± 20	–	500 ± 46	–	125 ± 10	–	–	–
**4b**	31.3 ± 2.3	–	–	500 ± 47	125 ± 9	–	–	–
**4c**	250 ± 18	>500	500 ± 48	125 ± 10	250 ± 20	500 ± 48	–	–
**4d**	31.3 ± 2.5	250 ± 22	>500	62.5 ± 5.4	250 ± 18	250 ± 19	500 ± 48	250 ± 18
**4e**	0.9 ± 0.07	7.8 ± 0.6	1.9 ± 0.1	0.5 ± 0.04	0.25 ± 0.02	62.5 ± 5.2	7.8 ± 0.6	7.8 ± 0.6
**9**	137 ± 21	–	–	137 ± 18	137 ± 21	–	–	–
**5b**	250 ± 20	–	–	500 ± 48	250 ± 20	500 ± 49	–	–
**5c**	125 ± 10	500 ± 48	500 ± 48	62.5 ± 5.5	62.5 ± 5.4	500 ± 48	>500	–
**5d**	125 ± 9	500 ± 47	125 ± 9	125 ± 11	62.5 ± 5.2	62.5 ± 5.3	500 ± 47	–
**6a**	125 ± 10	500 ± 46	–	500 ± 47	62.5 ± 5.6	–	–	–
**6b**	125 ± 11	–	–	31.3 ± 2.6	31.3 ± 2.5	–	–	–
**6c**	125 ± 11	>500	–	62.5 ± 5.4	7.8 ± 0.7	500 ± 47	250 ± 21	–
**6d**	62.5 ± 5.4	250 ± 21	250 ± 19	0.9 ± 0.07	0.9 ± 0.08	62.5 ± 5.5	125 ± 9	250 ± 19
**6e**	0.5 ± 0.04	31.3 ± 2.5	0.9 ± 0.1	1.9 ± 0.1	0.5 ± 0.04	125 ± 11	3.9 ± 0.4	15.6 ± 1.2
**8**	62.5 ± 5.3	500 ± 47	–	15.6 ± 1.2	15.6 ± 1.3	–	–	–
**7**	–	–	–	–	500 ± 47	–	–	–
Norfloxacin	7.5 ± 0.6	24.4 ± 2.1	24.4 ± 1.9	–	7.5 ± 0.6	24.4 ± 2.3	49.0 ± 4.2	
Ketoconazole								7.3 ± 0.5

Average of three values measured; ± standard deviation (SD); – means non-active. Data for the most active compounds are highlighted in green color.

**Table 5:** Hemolytic activity of Aminophosphonium salts *.

Test Compound	HC_50_ (μM)
**4b**	>100
**5b**	>100
**6b**	>100
Gramicidin S	9.4 ± 0.8

*—The experiments were repeated for three times. The results are expressed as the mean ± standard deviation (SD).

**Table 6:** Acute toxicity of compounds **4a**, **4b**, **4d**, **5a**, **5b** and **6b**.

Compound	LD_50_, mg/kg
**4a**	31.7 (28.3÷35.8)
**4b**	43.4 (41.8÷44.8)
**4d**	5.9 (3.7÷7.6)
**5a**	44.7 (43.0÷46.3)
**5b**	27.9 (26.9÷29.9)
**6b**	102.5 (98.7÷105.8)

## Data Availability

The data presented in this study are available on request from the corresponding author.

## Supplementary Materials

The following supporting information can be downloaded at: https://www.mdpi.com/article/10.3390/nano13212840/s1, Figures S1–S165: NMR spectra of synthesized compounds **4**–**9**; Table S1: Acute toxicity parameters LD_0–100_ of compounds **4a**, **4b**, **4d**, **5a**, **5b**, **6b**; Figures S167–S171: particle size distribution. Information on X-ray diffraction analysis (cif and checkcif files) of compound **6a** is also available.
