# Supplementary material for: Novel Mitochondria-Targeted Amphiphilic Aminophosphonium Salts and Lipids Nanoparticles: Synthesis, Antitumor Activity and Toxicity
Source: Nanomaterials (Basel). 2023 Oct 26;13(21):2840. doi: 10.3390/nano13212840 (PMC10649961; doi:10.3390/nano13212840)
Supplement: Supplementary file 1 [file nanomaterials-13-02840-s001.zip › nanomaterials-2642394-supplementary.pdf]

Electronic supplementary materials

Nanomaterials, 2023,

Novel mitochondria-targeted amphiphilic aminophosphonium salts and lipids nanoparticles: synthesis, antitumor activity and toxicity

Vladimir F. Mironov, Mudaris N. Dimukhametov, Andrey V. Nemtarev, Tatiana N. Pashirova, Olga V. Tsepaeva, Alexandra D. Voloshina, Alexandra B. Vyshtakalyuk, Igor A. Litvinov, Anna P. Lyubina, Anastasiya S. Sapunova, Dinara F. Abramova, Vladimir V. Zobov

Arbuzov Institute of Organic and Physical Chemistry, FRC Kazan Scientific Center of RAS,
Arbuzov Str. 8, 420088 Kazan, Russian Federation

Table of contents	S1
Figure 1S. ^{31}P-$\{^{1}H\}$ NMR spectrum (162.0 MHz, $CDCl_3$) of $(Et_2N)_3P^+–C_6H_{13}\ I^-$ (**4a**).	S7
Figure 2S. ^{1}H NMR spectrum (400.0 MHz, $CDCl_3$) of $(Et_2N)_3P^+–C_6H_{13}\ I^-$ (**4a**).	S8
Figure 3S. ^{13}C-$\{^{1}H\}$ NMR spectrum (100.6 MHz, $CDCl_3$) of $(Et_2N)_3P^+–C_6H_{13}\ I^-$ (**4a**).	S9
Figure 4S. ^{13}C-$\{^{1}H\}$ and ^{13}C-$\{^{1}H\}$-dept NMR spectra (100.6 MHz, $CDCl_3$) of $(Et_2N)_3P^+–C_6H_{13}\ I^-$ (**4a**.	S10
Figure 5S. ^{31}P-$\{^{1}H\}$ NMR spectrum (400.0 MHz, $CDCl_3$) of $(Et_2N)_3P^+–C_8H_{17}\ I^-$ (**4b**).	S11
Figure 6S. ^{1}H NMR spectrum (400.0 MHz, $CDCl_3$) of $(Et_2N)_3P^+–C_8H_{17}\ I^-$ (**4b**).	S12
Figure 7S. ^{13}C-$\{^{1}H\}$ NMR spectrum (100.6 MHz, $CDCl_3$) of $(Et_2N)_3P^+–C_8H_{17}\ I^-$ (**4b**).	S13
Figure 8S. ^{13}C-$\{^{1}H\}$ and ^{13}C-$\{^{1}H\}$-dept NMR spectra (100.6 MHz, $CDCl_3$) of $(Et_2N)_3P^+–C_8H_{17}\ I^-$ (**4b**).	S14
Figure 9S. ^{13}C-$\{^{1}H\}$ NMR spectra (400.0 MHz, $CDCl_3$) of $(Et_2N)_3P^+–C_8H_{17}\ I^-$ (**4b**, red) and $(Et_2N)_3P^+–C_6H_{13}\ I^-$ (**4a**, blue).	S15
Figure 10S. ^{13}C-$\{^{1}H\}$ NMR spectra (400.0 MHz, $CDCl_3$) of $(Et_2N)_3P^+–C_8H_{17}\ I^-$ (**4b**, red) and $(Et_2N)_3P^+–C_6H_{13}\ I^-$ (**4a**, blue).	S16
Figure 11S. ^{31}P-$\{^{1}H\}$ NMR spectrum (400.0 MHz, $CDCl_3$) of $(Et_2N)_3P^+–C_9H_{19}\ I^-$ (**4c**).	S17
Figure 12S. ^{1}H NMR spectrum (400.0 MHz, $CDCl_3$) of $(Et_2N)_3P^+–C_9H_{19}\ I^-$ (**4c**).	S18
Figure 13S. ^{13}C-$\{^{1}H\}$ NMR spectrum (100.6 MHz, $CDCl_3$) of $(Et_2N)_3P^+–C_9H_{19}\ I^-$ (**4c**).	S19
Figure 14S. ^{13}C-$\{^{1}H\}$ and ^{13}C-$\{^{1}H\}$-dept NMR spectra (100.6 MHz, $CDCl_3$) of $(Et_2N)_3P^+–C_9H_{19}\ I^-$ (**4c**).	S20
Figure 15S. ^{13}C-$\{^{1}H\}$ NMR spectra (400.0 MHz, $CDCl_3$) of $(Et_2N)_3P^+–C_9H_{19}\ I^-$ (**4c**, red) and $(Et_2N)_3P^+–C_8H_{17}\ I^-$ (**4b**, blue).	S21
Figure 16S. ^{13}C-$\{^{1}H\}$ NMR spectra (400.0 MHz, $CDCl_3$) of $(Et_2N)_3P^+–C_9H_{19}\ I^-$ (**4c**, red) and $(Et_2N)_3P^+–C_8H_{17}\ I^-$ (**4b**, blue).	S22

Figure 17S. $^{31}P-\{^{1}H\}$ NMR spectrum (400.0 MHz, $CDCl_3$) of $(Et_2N)_3P^{+}–C_{10}H_{21}$ $I^{–}$ (**4d**).	S23
Figure 18S. ^{1}H NMR spectrum (400.0 MHz, $CDCl_3$) of $(Et_2N)_3P^{+}–C_{10}H_{21}$ $I^{–}$ (**4d**).	S24
Figure 19S. $^{13}C-\{^{1}H\}$ NMR spectrum (100.6 MHz, $CDCl_3$) of $(Et_2N)_3P^{+}–C_{10}H_{21}$ $I^{–}$ (**4d**).	S25
Figure 20S. $^{13}C-\{^{1}H\}$ and $^{13}C-\{^{1}H\}$-dept NMR spectra (100.6 MHz, $CDCl_3$) of $(Et_2N)_3P^{+}–C_{10}H_{21}$ $I^{–}$ (**4d**).	S26
Figure 21S. $^{31}P-\{^{1}H\}$ NMR spectrum (162.0 MHz, $CDCl_3$) of $(Et_2N)_3P^{+}–C_{14}H_{29}$ $Br^{–}$ (**4d**).	S27
Figure 22S. ^{1}H NMR spectrum (400.0 MHz, $CDCl_3$) of $(Et_2N)_3P^{+}–C_{14}H_{29}$ $Br^{–}$ (**4d**).	S28
Figure 23S. ^{1}H NMR spectrum (500.0 MHz, $CDCl_3$) of $(Et_2N)_3P^{+}–C_{14}H_{29}$ $Br^{–}$ (**4d**.	S29
Figure 24S. $^{13}C-\{^{1}H\}$ NMR spectrum (125.0 MHz, $CDCl_3$) of $(Et_2N)_3P^{+}–C_{14}H_{29}$ $Br^{–}$ (**4d**).	S30
Figure 25S. ^{13}C NMR spectrum (125.0 MHz, $CDCl_3$) of $(Et_2N)_3P^{+}–C_{14}H_{29}$ $Br^{–}$ (**4d**).	S31
Figure 26S. High-field fragments of ^{13}C NMR spectrum (125.0 MHz, $CDCl_3$) of $(Et_2N)_3P^{+}–C_{14}H_{29}$ $Br^{–}$ (**4d**).	S32
Figure 27S. $^{13}C-\{^{1}H\}$ and $^{13}C-\{^{1}H\}$-dept NMR spectra (125.0 MHz, $CDCl_3$) of $(Et_2N)_3P^{+}–C_{14}H_{29}$ $Br^{–}$ (**4d**).	S33
Figure 28S. $^{13}C-\{^{1}H\}$ and ^{13}C NMR spectra (125.0 MHz, $CDCl_3$) of $(Et_2N)_3P^{+}–C_{14}H_{29}$ $Br^{–}$ (**4d**).	S34
Figure 29S. High-field fragments of $^{13}C-\{^{1}H\}$ and ^{13}C NMR spectra (125.0 MHz, $CDCl_3$) of $(Et_2N)_3P^{+}–C_{14}H_{29}$ $Br^{–}$ (**4d**).	S35
Figure 30S. $^{31}P-\{^{1}H\}$ NMR spectrum (162.0 MHz, $CDCl_3$) of $(Et_2N)_2(Ph)P^{+}–C_6H_{13}$ $I^{–}$ (**5a**).	S36
Figure 31S. ^{1}H NMR spectrum (400.0 MHz, $CDCl_3$) of $(Et_2N)_2(Ph)P^{+}–C_6H_{13}$ $I^{–}$ (**5a**).	S37
Figure 32S. High-field fragment of ^{1}H NMR spectrum (400.0 MHz, $CDCl_3$) of $(Et_2N)_2(Ph)P^{+}–C_6H_{13}$ $I^{–}$ (**5a**).	S38
Figure 33S. ^{1}H and $^{1}H-\{^{31}P\}$ NMR spectra (400.0 MHz, $CDCl_3$) of $(Et_2N)_2(Ph)P^{+}–C_6H_{13}$ $I^{–}$ (**5a**).	S39
Figure 34S. $^{13}C-\{^{1}H\}$ NMR spectrum (100.6 MHz, $CDCl_3$) of $(Et_2N)_2(Ph)P^{+}–C_6H_{13}$ $I^{–}$ (**5a**).	S40
Figure 35S. $^{13}C-\{^{1}H\}$ and $^{13}C-\{^{1}H\}$-dept NMR spectra (100.6 MHz, $CDCl_3$) of $(Et_2N)_2(Ph)P^{+}–C_6H_{13}$ $I^{–}$ (**5a**).	S41
Figure 36S. ^{13}C NMR spectrum (100.6 MHz, $CDCl_3$) of $(Et_2N)_2(Ph)P^{+}–C_6H_{13}$ $I^{–}$ (**5a**).	S42
Figure 37S. High-field fragments of ^{13}C NMR spectrum (100.6 MHz, $CDCl_3$) of $(Et_2N)_2(Ph)P^{+}–C_6H_{13}$ $I^{–}$ (**5a**)	S43
Figure 38S. $^{13}C-\{^{1}H\}$ and ^{13}C NMR spectra (100.6 MHz, $CDCl_3$) of $(Et_2N)_2(Ph)P^{+}–C_6H_{13}$ I (**5a**).	S44
Figure 39S. High-field region of $^{13}C-\{^{1}H\}$ and ^{13}C NMR spectra (100.6 MHz, $CDCl_3$) of $(Et_2N)_2(Ph)P^{+}–C_6H_{13}$ $I^{–}$ (**5a**).	S45
Figure 40S. The 121-123 and 20-32 ppm regions of $^{13}C-\{^{1}H\}$ and ^{13}C NMR spectra (100.6 MHz, $CDCl_3$) of $(Et_2N)_2(Ph)P^{+}–C_6H_{13}$ $I^{–}$ (**5a**).	S46
Figure 41S. $^{31}P-\{^{1}H\}$ NMR spectrum (162.0 MHz, $CDCl_3$) of $(Et_2N)_2(Ph)P^{+}–C_8H_{17}$ $I^{–}$ (**5b**).	S47
Figure 42S. ^{1}H NMR spectrum (400.0 MHz, $CDCl_3$) of $(Et_2N)_2(Ph)P^{+}–C_8H_{17}$ $I^{–}$ (**5b**).	S48
Figure 43S. $^{13}C-\{^{1}H\}$ NMR spectrum (100.6 MHz, $CDCl_3$) of $(Et_2N)_2(Ph)P^{+}–C_8H_{17}$ $I^{–}$ (**5b**).	S49
Figure 44S. $^{13}C-\{^{1}H\}$ and $^{13}C-\{^{1}H\}$-dept NMR spectra (100.6 MHz, $CDCl_3$) of $(Et_2N)_2(Ph)P^{+}–C_8H_{17}$ $I^{–}$ (**5b**).	S50
Figure 45S. High-field region of $^{13}C-\{^{1}H\}$ and $^{13}C-\{^{1}H\}$-dept NMR spectra (100.6 MHz, $CDCl_3$) of $(Et_2N)_2(Ph)P^{+}–C_8H_{17}$ $I^{–}$ (**5b**).	S51
Figure 46S. ^{13}C NMR spectrum (100.6 MHz, $CDCl_3$) of $(Et_2N)_2(Ph)P^{+}–C_8H_{17}$ $I^{–}$ (**5b**).	S52
Figure 47S. High-field fragments of ^{13}C NMR spectrum (100.6 MHz, $CDCl_3$) of $(Et_2N)_2(Ph)P^{+}–C_8H_{17}$ $I^{–}$ (**5b**).	S53
Figure 48S. $^{13}C-\{^{1}H\}$ and ^{13}C NMR spectra (100.6 MHz, $CDCl_3$) of $(Et_2N)_2(Ph)P^{+}–C_8H_{17}$ $I^{–}$ (**5b**).	S54

Figure 49S. High-field fragments of ^{13}C-{^{1}H} and ^{13}C NMR spectra (100.6 MHz, $CDCl_3$) of $(Et_2N)_2(Ph)P^+–C_8H_{17}$ $I^–$ (**5b**).	S55
Figure 50S. The 20-32 ppm region of ^{13}C-{^{1}H} and ^{13}C NMR spectra (100.6 MHz, $CDCl_3$) of $(Et_2N)_2(Ph)P^+–C_8H_{17}$ $I^–$ (**5b**).	S56
Figure 51S. The 11-16 ppm region of ^{13}C-{^{1}H} and ^{13}C NMR spectra (100.6 MHz, $CDCl_3$) of $(Et_2N)_2(Ph)P^+–C_8H_{17}$ $I^–$ (**5b**).	S57
Figure 52S. ^{31}P-{^{1}H} NMR spectrum (162.0 MHz, $CDCl_3$) of $(Et_2N)_2(Ph)P^+–C_9H_{19}$ $I^–$ (**5c**).	S58
Figure 53S. ^{1}H NMR spectrum (400.0 MHz, $CDCl_3$) of $(Et_2N)_2(Ph)P^+–C_9H_{19}$ $I^–$ (**5c**).	S59
Figure 54S. Fragments of ^{1}H NMR spectrum (400.0 MHz, $CDCl_3$) of $(Et_2N)_2(Ph)P^+–C_9H_{19}$ $I^–$ (**5c**).	S60
Figure 55S. ^{13}C-{^{1}H} NMR spectrum (100.6 MHz, $CDCl_3$) of $(Et_2N)_2(Ph)P^+–C_9H_{19}$ $I^–$ (**5c**).	S61
Figure 56S. ^{13}C-{^{1}H} and ^{13}C-{^{1}H}-dept NMR spectra (100.6 MHz, $CDCl_3$) of $(Et_2N)_2(Ph)P^+–C_9H_{19}$ $I^–$ (**5c**).	S62
Figure 57S. ^{13}C NMR spectrum (100.6 MHz, $CDCl_3$) of $(Et_2N)_2(Ph)P^+–C_9H_{19}$ $I^–$ (**5c**).	S63
Figure 58S. The 20-27 and 27-32 ppm regions of ^{13}C NMR spectrum (100.6 MHz, $CDCl_3$) of $(Et_2N)_2(Ph)P^+–C_9H_{19}$ $I^–$ (**5c**).	S64
Figure 59S. High-field fragment of ^{13}C NMR spectrum (100.6 MHz, $CDCl_3$) of $(Et_2N)_2(Ph)P^+–C_9H_{19}$ $I^–$ (**5c**).	S65
Figure 60S. ^{13}C and ^{13}C-{^{1}H} NMR spectra (100.6 MHz, $CDCl_3$) of $(Et_2N)_2(Ph)P^+–C_9H_{19}$ $I^–$ (**5c**).	S66
Figure 61S. Low-field fragment of ^{13}C and ^{13}C-{^{1}H} NMR spectra (100.6 MHz, $CDCl_3$) of $(Et_2N)_2(Ph)P^+–C_9H_{19}$ $I^–$ (**5c**).	S67
Figure 62S. High-field fragment of ^{13}C and ^{13}C-{^{1}H} NMR spectra (100.6 MHz, $CDCl_3$) of $(Et_2N)_2(Ph)P^+–C_9H_{19}$ $I^–$ (**5c**).	S68
Figure 63S. ^{31}P-{^{1}H} NMR spectrum (400.0 MHz, $CDCl_3$) of $(Et_2N)_2(Ph)P^+–C_{10}H_{21}$ $I^–$ (**5d**).	S69
Figure 64S. ^{1}H NMR spectrum (400.0 MHz, $CDCl_3$) of $(Et_2N)_2(Ph)P^+–C_{10}H_{21}$ $I^–$ (**5d**).	S70
Figure 65S. Figure 65S. Fragments of ^{1}H NMR spectrum (400.0 MHz, $CDCl_3$) of $(Et_2N)_2(Ph)P^+–C_{10}H_{21}$ $I^–$ (**5d**).	S71
Figure 66S. ^{13}C-{^{1}H} NMR spectrum (100.6 MHz, $CDCl_3$) of $(Et_2N)_2(Ph)P^+–C_{10}H_{21}$ $I^–$ (**5d**).	S72
Figure 67S. ^{13}C-{^{1}H} and ^{13}C-{^{1}H}-dept NMR spectra (100.6 MHz, $CDCl_3$) of $(Et_2N)_2(Ph)P^+–C_{10}H_{21}$ $I^–$ (**5d**).	S73
Figure 68S. ^{13}C NMR spectrum (100.6 MHz, $CDCl_3$) of $(Et_2N)_2(Ph)P^+–C_{10}H_{21}$ $I^–$ (**5d**).	S74
Figure 69S. High-field fragments of ^{13}C NMR spectrum (100.6 MHz, $CDCl_3$) of $(Et_2N)_2(Ph)P^+–C_{10}H_{21}$ $I^–$ (**5d**).	S75
Figure 70S. ^{13}C and ^{13}C-{^{1}H}-dept NMR spectra (100.6 MHz, $CDCl_3$) of $(Et_2N)_2(Ph)P^+–C_{10}H_{21}$ $I^–$ (**5d**).	S76
Figure 71S. Low-field region of ^{13}C and ^{13}C-{^{1}H}-dept NMR spectra (100.6 MHz, $CDCl_3$) of $(Et_2N)_2(Ph)P^+–C_{10}H_{21}$ $I^–$ (**5d**).	S77
Figure 72S. High-field region of ^{13}C and ^{13}C-{^{1}H}-dept NMR spectra (100.6 MHz, $CDCl_3$) of $(Et_2N)_2(Ph)P^+–C_{10}H_{21}$ $I^–$ (**5d**).	S78
Figure 73S. ^{13}C and ^{13}C-{^{1}H} NMR spectra (100.6 MHz, $CDCl_3$) of $(Et_2N)_2(Ph)P^+–C_{10}H_{21}$ $I^–$ (**5d**).	S79
Figure 74S. High-field region of ^{13}C and ^{13}C-{^{1}H} NMR spectra (100.6 MHz, $CDCl_3$) of $(Et_2N)_2(Ph)P^+–C_{10}H_{21}$ $I^–$ (**5d**).	S80
Figure 75S. The 20-33 ppm region of ^{13}C and ^{13}C-{^{1}H} NMR spectra (100.6 MHz, $CDCl_3$) of $(Et_2N)_2(Ph)P^+–C_{10}H_{21}$ $I^–$ (**5d**).	S81
Figure 76S. The 11-16 ppm region of ^{13}C and ^{13}C-{^{1}H} NMR spectra (100.6 MHz, $CDCl_3$) of $(Et_2N)_2(Ph)P^+–C_{10}H_{21}$ $I^–$ (**5d**).	S82
Figure 77S. ^{31}P-{^{1}H} NMR spectrum (162.0 MHz, $CDCl_3$) of $(Et_2N)Ph_2P^+–C_6H_{13}$ $I^–$ (**6a**).	S83
Figure 78S. ^{1}H NMR spectrum (400.0 MHz, $CDCl_3$) of $(Et_2N)Ph_2P^+–C_6H_{13}$ $I^–$ (**6a**).	S84
Figure 79S. ^{1}H NMR spectrum (500.0 MHz, $CDCl_3$) of $(Et_2N)Ph_2P^+–C_6H_{13}$ $I^–$ (**6a**).	S85
Figure 80S. ^{13}C-{^{1}H} NMR spectrum (125.0 MHz, $CDCl_3$) of $(Et_2N)Ph_2P^+–C_6H_{13}$ $I^–$ (**6a**).	S86
Figure 81S. ^{13}C NMR spectrum (125.8 MHz, $CDCl_3$) of $(Et_2N)Ph_2P^+–C_6H_{13}$ $I^–$ (**6a**).	S87

Figure 82S. High-field fragments of ^{13}C NMR spectrum (125.8 MHz, CDCl$_3$) of (Et$_2$N)Ph$_2$P$^+$–C$_6$H$_{13}$ I$^-$ (**6a**).	S88
Figure 83S. ^{13}C-{^{1}H} and ^{13}C-{^{1}H}-dept NMR spectra (125.8 MHz, CDCl$_3$) of (Et$_2$N)Ph$_2$P$^+$–C$_6$H$_{13}$ I$^-$ (**6a**).	S89
Figure 84S. High-field fragment of ^{13}C-{^{1}H} and ^{13}C-{^{1}H}-dept NMR spectra (125.8 MHz, CDCl$_3$) of (Et$_2$N)Ph$_2$P$^+$–C$_6$H$_{13}$ I$^-$ (**6a**).	S90
Figure 85S. ^{13}C-{^{1}H} and ^{13}C NMR spectra (125.8 MHz, CDCl$_3$) of (Et$_2$N)Ph$_2$P$^+$–C$_6$H$_{13}$ I$^-$ (**6a**).	S91
Figure 86S. Low-field fragment of ^{13}C-{^{1}H} and ^{13}C NMR spectra (125.8 MHz, CDCl$_3$) of (Et$_2$N)Ph$_2$P$^+$–C$_6$H$_{13}$ I$^-$ (**6a**).	S92
Figure 87S. High-field fragment of ^{13}C-{^{1}H} and ^{13}C NMR spectra (125.8 MHz, CDCl$_3$) of (Et$_2$N)Ph$_2$P$^+$–C$_6$H$_{13}$ I$^-$ (**6a**).	S93
Figure 88S. The 20-32 ppm region of ^{13}C-{^{1}H} and ^{13}C NMR spectra (125.8 MHz, CDCl$_3$) of (Et$_2$N)Ph$_2$P$^+$–C$_6$H$_{13}$ I$^-$ (**6a**).	S94
Figure 89S. The 11-16 ppm region of ^{13}C-{^{1}H} and ^{13}C NMR spectra (125.8 MHz, CDCl$_3$) of (Et$_2$N)Ph$_2$P$^+$–C$_6$H$_{13}$ I$^-$ (**6a**).	S95
Figure 90S. ^{31}P-{^{1}H} NMR spectrum (162.0 MHz, CDCl$_3$) of (Et$_2$N)Ph$_2$P$^+$–C$_8$H$_{17}$ I$^-$ (**6b**).	S96
Figure 91S. ^{1}H NMR spectrum (500.0 MHz, CDCl$_3$) of (Et$_2$N)Ph$_2$P$^+$–C$_8$H$_{17}$ I$^-$ (**6b**).	S97
Figure 92S. ^{13}C-{^{1}H} NMR spectrum (125.8 MHz, CDCl$_3$) of (Et$_2$N)Ph$_2$P$^+$–C$_8$H$_{17}$ I$^-$ (**6b**).	S98
Figure 93S. ^{13}C NMR spectrum (125.8 MHz, CDCl$_3$) of (Et$_2$N)Ph$_2$P$^+$–C$_8$H$_{17}$ I$^-$ (**6b**).	S99
Figure 94S. High-field fragments of ^{13}C NMR spectrum (125.8 MHz, CDCl$_3$) of (Et$_2$N)Ph$_2$P$^+$–C$_8$H$_{17}$ I$^-$ (**6b**).	S100
Figure 95S. ^{13}C-{^{1}H} and ^{13}C-{^{1}H}-dept NMR spectra (125.8 MHz, CDCl$_3$) of (Et$_2$N)Ph$_2$P$^+$–C$_8$H$_{17}$ I$^-$ (**6b**).	S101
Figure 96S. High-field fragment of ^{13}C-{^{1}H} and ^{13}C-{^{1}H}-dept NMR spectra (125.8 MHz, CDCl$_3$) of (Et$_2$N)Ph$_2$P$^+$–C$_8$H$_{17}$ I$^-$ (**6b**).	S102
Figure 97S. ^{13}C-{^{1}H} and ^{13}C NMR spectra (125.8 MHz, CDCl$_3$) of (Et$_2$N)Ph$_2$P$^+$–C$_8$H$_{17}$ I$^-$ (**6b**).	S103
Figure 98S. Low-field fragments of ^{13}C-{^{1}H} and ^{13}C NMR spectra (125.8 MHz, CDCl$_3$) of (Et$_2$N)Ph$_2$P$^+$–C$_8$H$_{17}$ I$^-$ (**6b**).	S104
Figure 99S. High-field fragment of ^{13}C-{^{1}H} and ^{13}C NMR spectra (125.8 MHz, CDCl$_3$) of (Et$_2$N)Ph$_2$P$^+$–C$_8$H$_{17}$ I$^-$ (**6b**).	S105
Figure 100S. ^{31}P-{^{1}H} NMR spectrum (162.0 MHz, CDCl$_3$) of (Et$_2$N)Ph$_2$P$^+$–C$_9$H$_{19}$ I$^-$ (**6c**).	S106
Figure 101S. ^{1}H NMR spectrum (400.0 MHz, CDCl$_3$) of (Et$_2$N)Ph$_2$P$^+$–C$_9$H$_{19}$ I$^-$ (**6c**).	S107
Figure 102S. ^{13}C-{^{1}H} NMR spectrum (100.6 MHz, CDCl$_3$) of (Et$_2$N)Ph$_2$P$^+$–C$_9$H$_{19}$ I$^-$ (**6c**).	S108
Figure 103S. ^{13}C NMR spectrum (100.6 MHz, CDCl$_3$) of (Et$_2$N)Ph$_2$P$^+$–C$_9$H$_{19}$ I$^-$ (**6c**).	S109
Figure 104S. High-field fragments of ^{13}C NMR spectrum (100.6 MHz, CDCl$_3$) of (Et$_2$N)Ph$_2$P$^+$–C$_9$H$_{19}$ I$^-$ (**6c**).	S110
Figure 105S. ^{13}C-{^{1}H} and ^{13}C-{^{1}H}-dept NMR spectra (100.6 MHz, CDCl$_3$) of (Et$_2$N)Ph$_2$P$^+$–C$_9$H$_{19}$ I$^-$ (**6c**).	S111
Figure 106S. High-field fragment of ^{13}C-{^{1}H} and ^{13}C-{^{1}H}-dept NMR spectra (100.6 MHz, CDCl$_3$) of (Et$_2$N)Ph$_2$P$^+$–C$_9$H$_{19}$ I$^-$ (**6c**).	S112
Figure 107S. ^{13}C-{^{1}H} and ^{13}C NMR spectra (100.6 MHz, CDCl$_3$) of (Et$_2$N)Ph$_2$P$^+$–C$_9$H$_{19}$ I$^-$ (**6c**).	S113
Figure 108S. Low-field fragments of ^{13}C-{^{1}H} and ^{13}C NMR spectra (100.6 MHz, CDCl$_3$) of (Et$_2$N)Ph$_2$P$^+$–C$_9$H$_{19}$ I$^-$ (**6c**).	S114
Figure 109S. High-field fragment of ^{13}C-{^{1}H} and ^{13}C NMR spectra (100.6 MHz, CDCl$_3$) of (Et$_2$N)Ph$_2$P$^+$–C$_9$H$_{19}$ I$^-$ (**6c**).	S115
Figure 110S. The 21-33 ppm region of ^{13}C-{^{1}H} and ^{13}C NMR spectra (100.6 MHz, CDCl$_3$) of (Et$_2$N)Ph$_2$P$^+$–C$_9$H$_{19}$ I$^-$ (**6c**).	S116
Figure 111S. ^{31}P-{^{1}H} NMR spectrum (162.0 MHz, CDCl$_3$) of (Et$_2$N)Ph$_2$P$^+$–C$_{10}$H$_{21}$ I$^-$ (**6c**).	S117
Figure 112S. ^{1}H NMR spectrum (400.0 MHz, CDCl$_3$) of (Et$_2$N)Ph$_2$P$^+$–C$_{10}$H$_{21}$ I$^-$ (**6d**).	S118
Figure 113S. ^{1}H NMR spectrum (600.0 MHz, CDCl$_3$) of (Et$_2$N)Ph$_2$P$^+$–C$_{10}$H$_{21}$ I$^-$ (**6d**).	S119
Figure 114S. ^{13}C-{^{1}H} NMR spectrum (100.6 MHz, CDCl$_3$) of (Et$_2$N)Ph$_2$P$^+$–C$_{10}$H$_{21}$ I$^-$ (**6d**).	S120

Figure 115S. ^{13}C NMR spectrum (100.6 MHz, $CDCl_3$) of $(Et_2N)Ph_2P^+–C_{10}H_{21}$ $I^–$ (**6d**).	S121
Figure 116S. High-field fragments of ^{13}C NMR spectrum (100.6 MHz, $CDCl_3$) of $(Et_2N)Ph_2P^+–C_{10}H_{21}$ $I^–$ (**6d**).	S122
Figure 117S. ^{13}C-{^1H} and ^{13}C-{^1H}-dept NMR spectra (100.6 MHz, $CDCl_3$) of $(Et_2N)Ph_2P^+–C_{10}H_{21}$ $I^–$ (**6d**).	S123
Figure 118S. High-field fragment of ^{13}C-{^1H} and ^{13}C-{^1H}-dept NMR spectra (100.6 MHz, $CDCl_3$) of $(Et_2N)Ph_2P^+–C_{10}H_{21}$ $I^–$ (**6d**).	S124
Figure 119S. ^{13}C-{^1H} and ^{13}C NMR spectra (100.6 MHz, $CDCl_3$) of $(Et_2N)Ph_2P^+–C_{10}H_{21}$ $I^–$ (**6d**).	S125
Figure 120S. The 119-121, 40-44 and 11-16 ppm regions of ^{13}C-{^1H} and ^{13}C NMR spectra (100.6 MHz, $CDCl_3$) of $(Et_2N)Ph_2P^+–C_{10}H_{21}$ $I^–$ (**6d**).	S126
Figure 121S. The 20-34 ppm region of ^{13}C-{^1H} and ^{13}C NMR spectra (100.6 MHz, $CDCl_3$) of $(Et_2N)Ph_2P^+–C_{10}H_{21}$ $I^–$ (**6d**).	S127
Figure 122S. ^{31}P-{^1H} NMR spectrum (162.0 MHz, $CDCl_3$) of $(Et_2N)Ph_2P^+–C_{14}H_{29}$ $Br^–$ (**6e**).	S128
Figure 123S. ^1H NMR spectrum (400.0 MHz, $CDCl_3$) of $(Et_2N)Ph_2P^+–C_{14}H_{29}$ $Br^–$ (**6e**).	S129
Figure 124S. High-field fragment of ^1H NMR spectrum (400.0 MHz, $CDCl_3$) of $(Et_2N)Ph_2P^+–C_{14}H_{29}$ $Br^–$ (**6e**).	S130
Figure 125S. ^1H NMR spectrum (500.0 MHz, $CDCl_3$) of $(Et_2N)Ph_2P^+–C_{14}H_{29}$ $Br^–$ (**6e**).	S131
Figure 126S. ^{13}C-{^1H} NMR spectrum (125.8 MHz, $CDCl_3$) of $(Et_2N)Ph_2P^+–C_{14}H_{29}$ $Br^–$ (**6e**).	S132
Figure 127S. ^{13}C NMR spectrum (125.8 MHz, $CDCl_3$) of $(Et_2N)Ph_2P^+–C_{14}H_{29}$ $Br^–$ (**6e**).	S133
Figure 128S. High-field fragments of ^{13}C NMR spectrum (125.8 MHz, $CDCl_3$) of $(Et_2N)Ph_2P^+–C_{14}H_{29}$ $Br^–$ (**6e**).	S134
Figure 129S. ^{13}C-{^1H} and ^{13}C-{^1H}-dept NMR spectra (125.8 MHz, $CDCl_3$) of $(Et_2N)Ph_2P^+–C_{14}H_{29}$ $Br^–$ (**6e**).	S135
Figure 130S. High-field fragment of ^{13}C-{^1H} and ^{13}C-{^1H}-dept NMR spectra (125.8 MHz, $CDCl_3$) of $(Et_2N)Ph_2P^+–C_{14}H_{29}$ $Br^–$ (**6e**).	S136
Figure 131S. The 21-32 ppm region of ^{13}C-{^1H} and ^{13}C-{^1H}-dept NMR spectra (125.8 MHz, $CDCl_3$) of $(Et_2N)Ph_2P^+–C_{14}H_{29}$ $Br^–$ (**6e**).	S137
Figure 132S. ^{13}C-{^1H} and ^{13}C NMR spectra (125.8 MHz, $CDCl_3$) of $(Et_2N)Ph_2P^+–C_{14}H_{29}$ $Br^–$ (**6e**).	S138
Figure 133S. Low-field fragments of ^{13}C-{^1H} and ^{13}C NMR spectra (125.8 MHz, $CDCl_3$) of $(Et_2N)Ph_2P^+–C_{14}H_{29}$ $Br^–$ (**6e**).	S139
Figure 134S. High-field fragment of ^{13}C-{^1H} and ^{13}C NMR spectra (125.8 MHz, $CDCl_3$) of $(Et_2N)Ph_2P^+–C_{14}H_{29}$ $Br^–$ (**6e**).	S140
Figure 135S. The 20-33 ppm region of ^{13}C-{^1H} and ^{13}C NMR spectra (125.8 MHz, $CDCl_3$) of $(Et_2N)Ph_2P^+–C_{14}H_{29}$ $Br^–$ (**6e**).	S141
Figure 136S. The 28-31 ppm region of ^{13}C-{^1H} spectra (125.8 MHz, $CDCl_3$) of $(Et_2N)Ph_2P^+–C_{14}H_{29}$ $Br^–$ (**6e**) and $(Et_2N)_3P^+–C_{14}H_{29}$ $Br^–$ (**4e**).	S142
Figure 137S. ^1H NMR spectrum (600.0 MHz, $CDCl_3$) of $Et_3N^+–C_8H_{17}$ $I^–$ (**7**).	S143
Figure 138S. High-field fragment of ^1H NMR spectrum (600.0 MHz, $CDCl_3$) of $Et_3N^+–C_8H_{17}$ $I^–$ (**7**).	S144
Figure 139S. ^{13}C-{^1H} NMR spectrum (100.6 MHz, $CDCl_3$) of $Et_3N^+–C_8H_{17}$ $I^–$ (**7**).	S145
Figure 140S. ^{13}C-{^1H} and ^{13}C-{^1H}-dept NMR spectra (100.6 MHz, $CDCl_3$) of $Et_3N^+–C_8H_{17}$ $I^–$ (**7**).	S146
Figure 141S. ^{13}C NMR spectrum (100.6 MHz, $CDCl_3$) of $Et_3N^+–C_8H_{17}$ $I^–$ (**7**).	S147
Figure 142S. The 50-59 and 19-33 ppm regions of ^{13}C NMR spectrum (100.6 MHz, $CDCl_3$) of $Et_3N^+–C_8H_{17}$ $I^–$ (**7**).	S148
Figure 143S. ^{13}C and ^{13}C-{^1H} NMR spectra (100.6 MHz, $CDCl_3$) of $Et_3N^+–C_8H_{17}$ $I^–$ (**7**).	S149
Figure 144S. The 11-32 ppm region of ^{13}C and ^{13}C-{^1H} NMR spectra (100.6 MHz, $CDCl_3$) of $Et_3N^+–C_8H_{17}$ $I^–$ (**7**).	S150

Figure 145S. The 5-10 and 50-59 ppm regions of ^{13}C and ^{13}C-{^1H} NMR spectra (100.6 MHz, $CDCl_3$) of $Et_3N^+C_8H_{17}$ I^- (**7**).	S151
Figure 146S. ^{31}P-{^1H} NMR spectrum (162.0 MHz, $CDCl_3$) of Ph_3P^+–C_8H_{17} I^- (**8**).	S152
Figure 147S. ^1H NMR spectrum (400.0 MHz, $CDCl_3$) of Ph_3P^+–C_8H_{17} I^- (**8**).	S153
Figure 148S. ^1H NMR spectrum (500.0 MHz, $CDCl_3$) of Ph_3P^+–C_8H_{17} I^- (**8**).	S154
Figure 149S. ^{13}C-{^1H} NMR spectrum (100.6 MHz, $CDCl_3$) of Ph_3P^+–C_8H_{17} I^- (**8**).	S155
Figure 150S. ^{13}C-{^1H} and ^{13}C-{^1H}-dept NMR spectra (100.6 MHz, $CDCl_3$) of Ph_3P^+–C_8H_{17} I^- (**8**).	S156
Figure 151S. ^{13}C-{^1H} NMR spectrum (125.8 MHz, $CDCl_3$) of Ph_3P^+–C_8H_{17} I^- (**8**).	S157
Figure 152S. ^{13}C-{^1H} and ^{13}C-{^1H}-dept NMR spectra (125.8 MHz, $CDCl_3$) of Ph_3P^+–C_8H_{17} I^- (**8**).	S158
Figure 153S. ^{13}C NMR spectrum (125.8 MHz, $CDCl_3$) of Ph_3P^+–C_8H_{17} I^- (**8**).	S159
Figure 154S. High-field fragments of ^{13}C NMR spectrum (125.8 MHz, $CDCl_3$) of Ph_3P^+–C_8H_{17} I^- (**8**).	S160
Figure 155S. ^{13}C and ^{13}C-{^1H} NMR spectra (125.8 MHz, $CDCl_3$) of Ph_3P^+–C_8H_{17} I^- (**8**).	S161
Figure 156S. The 117-119 and 26-33 ppm regions of ^{13}C and ^{13}C-{^1H} NMR spectra (125.8 MHz, $CDCl_3$) of Ph_3P^+–C_8H_{17} I^- (**8**).	S162
Figure 157S. High-field fragment of ^{13}C and ^{13}C-{^1H} NMR spectra (125.8 MHz, $CDCl_3$) of Ph_3P^+–C_8H_{17} I^- (**8**).	S163
Figure 158S. ^{31}P-{^1H} NMR spectrum (162.0 MHz, $CDCl_3$) of $(Et_2N)_3P^+$–C_6H_{12}–$P^+(NEt_2)_3$ $2Br^-$ (**9**).	S164
Figure 159S. ^1H NMR spectrum (400.0 MHz, $CDCl_3$) of $(Et_2N)_3P^+$–C_6H_{12}–$P^+(NEt_2)_3$ $2Br^-$ (**9**). Figure 159. ^{13}C-{^1H} NMR spectrum (100.6 MHz, $CDCl_3$) of $(Et_2N)_3P^+$–C_6H_{12}–$P^+(NEt_2)_3$ $2Br^-$ (**9**).	S165
Figure 160S. ^{13}C-{^1H} NMR spectrum (100.6 MHz, $CDCl_3$) of $(Et_2N)_3P^+$–C_6H_{12}–$P^+(NEt_2)_3$ $2Br^-$ (**9**).	S166
Figure 161S. ^{13}C-{^1H} and ^{13}C-{^1H}-dept NMR spectra (100.6 MHz, $CDCl_3$) of $(Et_2N)_3P^+$–C_6H_{12}–$P^+(NEt_2)_3$ $2Br^-$ (**9**).	S167
Figure 162S. High-field fragment of ^{13}C-{^1H} and ^{13}C-{^1H}-dept NMR spectra (100.6 MHz, $CDCl_3$) of $(Et_2N)_3P^+$–C_6H_{12}–$P^+(NEt_2)_3$ $2Br^-$ (**9**).	S168
Figure 163S. ^{13}C NMR spectrum (100.6 MHz, $CDCl_3$) of $(Et_2N)_3P^+$–C_6H_{12}–$P^+(NEt_2)_3$ $2Br^-$ (**9**).	S169
Figure 164S. ^{13}C and ^{13}C-{^1H} NMR spectra (100.6 MHz, $CDCl_3$) of $(Et_2N)_3P^+$–C_6H_{12}–$P^+(NEt_2)_3$ $2Br^-$ (**9**).	S170
Figure 165S. High-field fragments of ^{13}C NMR spectrum (100.6 MHz, $CDCl_3$) of $(Et_2N)_3P^+$–C_6H_{12}–$P^+(NEt_2)_3$ $2Br^-$ (**9**).	S171
Table S1. Acute toxicity parameters LD_{0-100} of compounds **4a**, **4b**, **4d**, **5a**, **5b**, **6b**.	S172

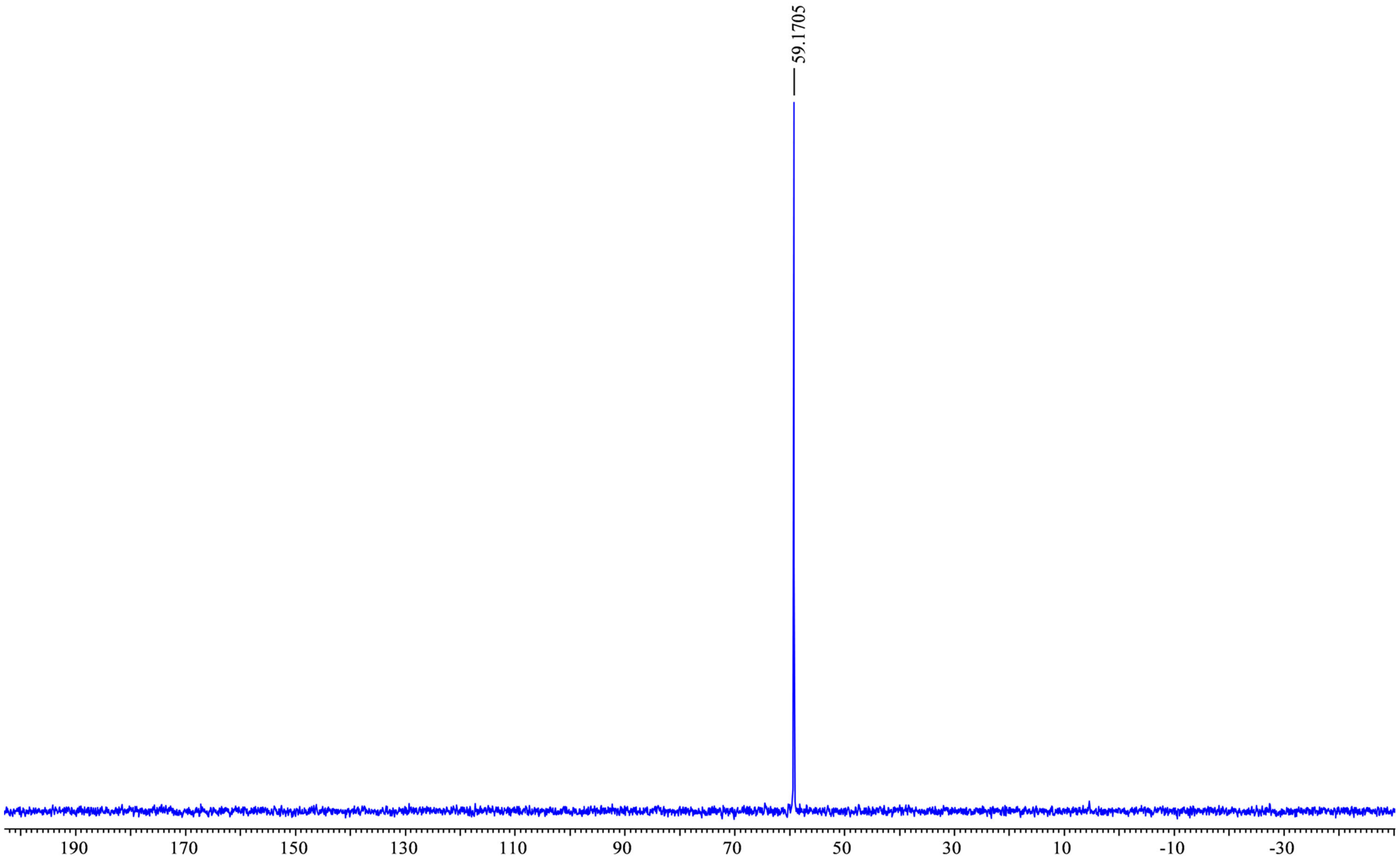

Figure 1S. ^{31}P-{^{1}H} NMR spectrum (162.0 MHz, $CDCl_3$) of $(Et_2N)_3P^+–C_6H_{13}\ I^–$ (**4a**).

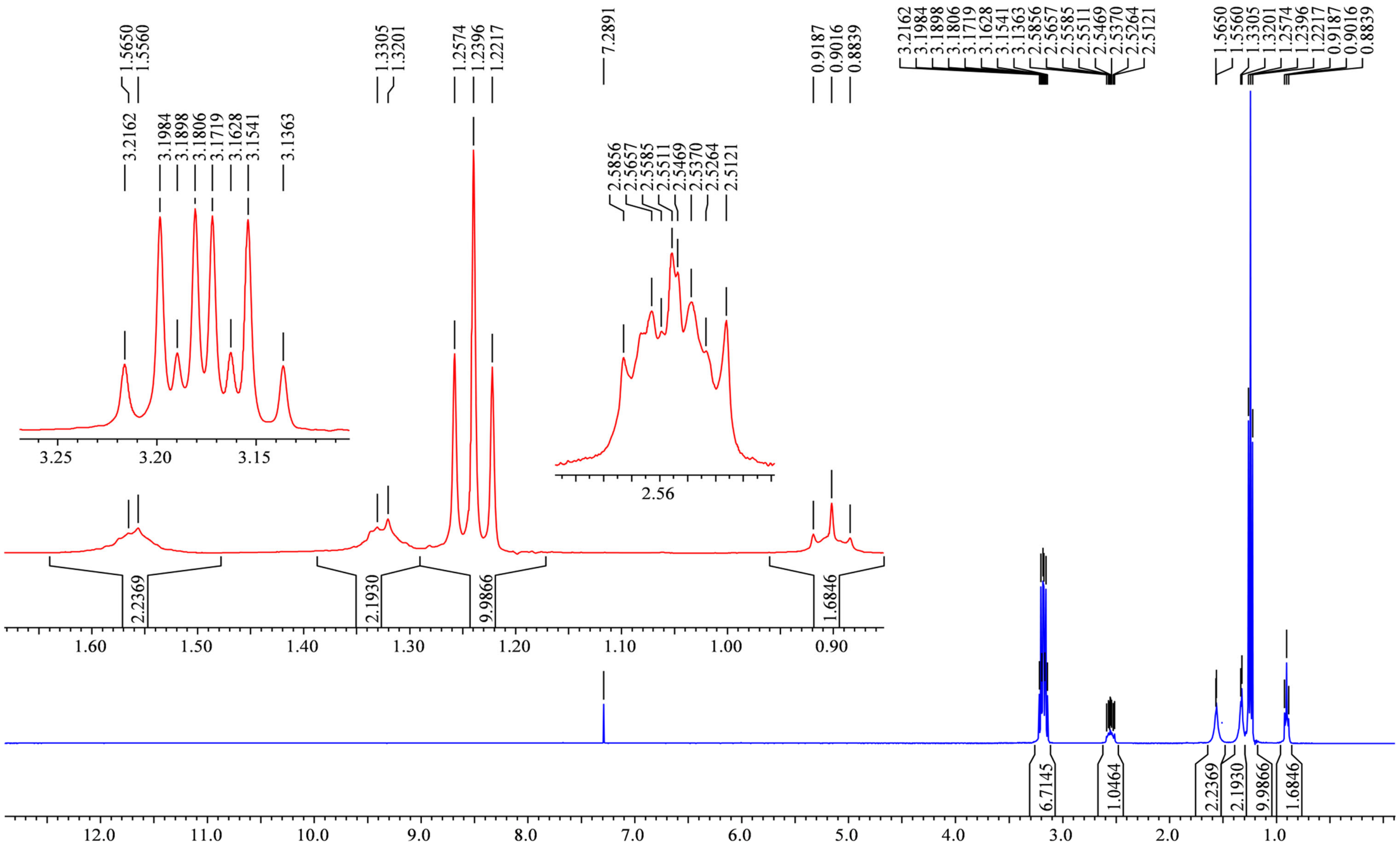

Figure 2S. ^{1}H NMR spectrum (400.0 MHz, $CDCl_3$) of $(Et_2N)_3P^+–C_6H_{13}\ I^-$ (**4a**).

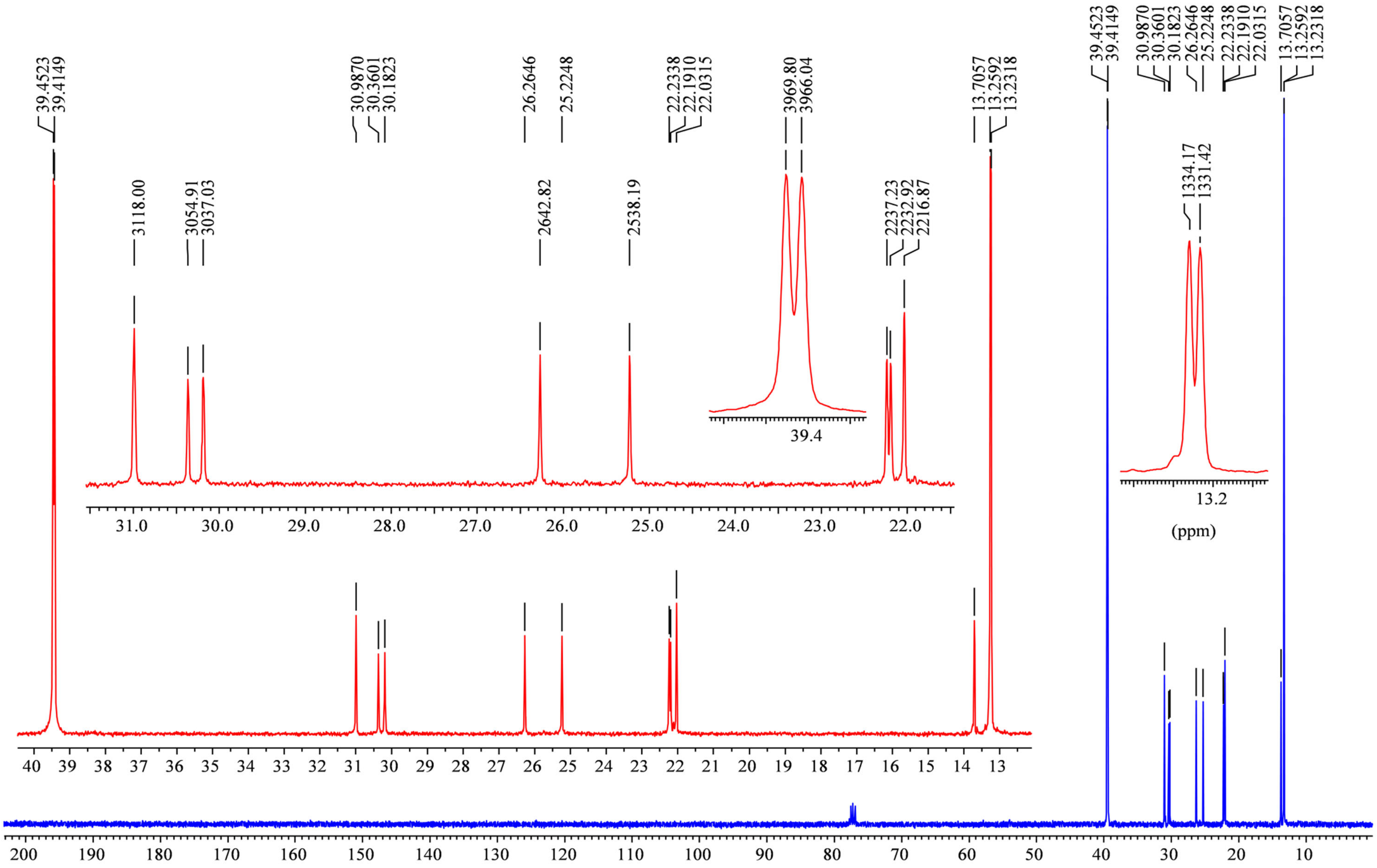

Figure 3S. ^{13}C-{^{1}H} NMR spectrum (100.6 MHz, $CDCl_3$) of $(Et_2N)_3P^+–C_6H_{13}\ I^-$ (**4a**).

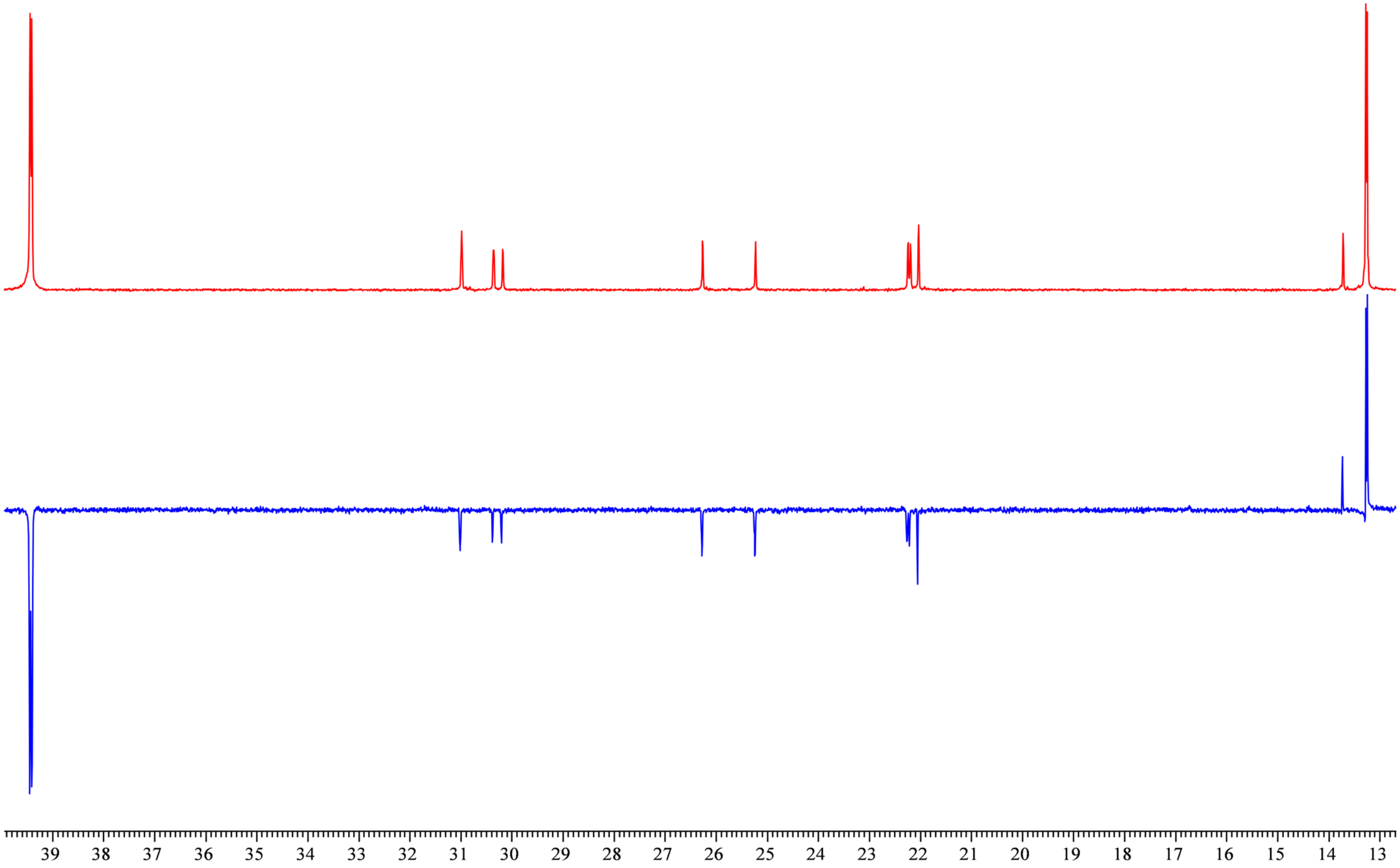

Figure 4S. ^{13}C-{^{1}H} and ^{13}C-{^{1}H}-dept NMR spectra (100.6 MHz, $CDCl_3$) of $(Et_2N)_3P^+$–C_6H_{13} I^- (**4a**).

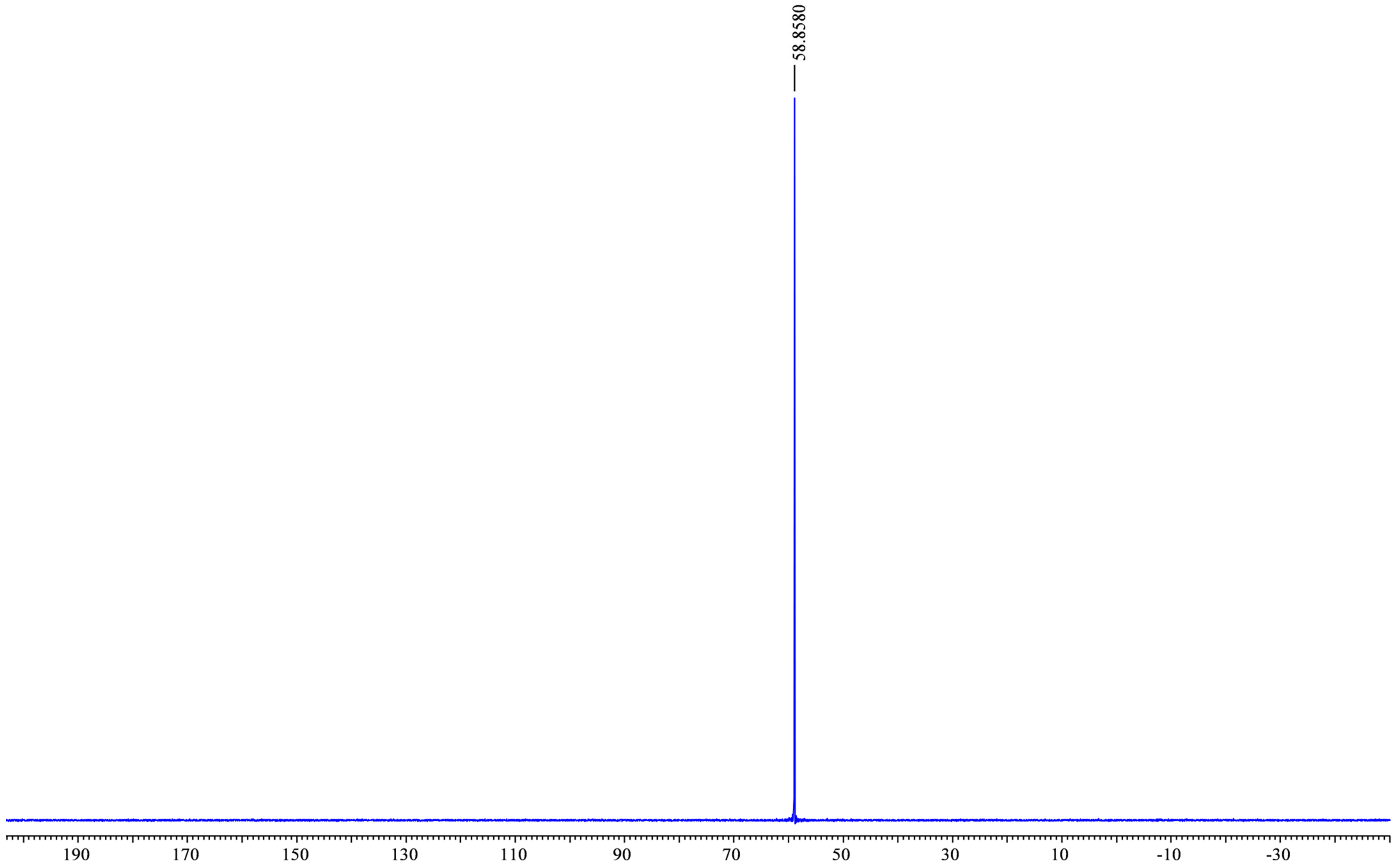

Figure 5S. ^{31}P-{^{1}H} NMR spectrum (400.0 MHz, $CDCl_3$) of $(Et_2N)_3P^+–C_8H_{17}\ I^−$ (**4b**).

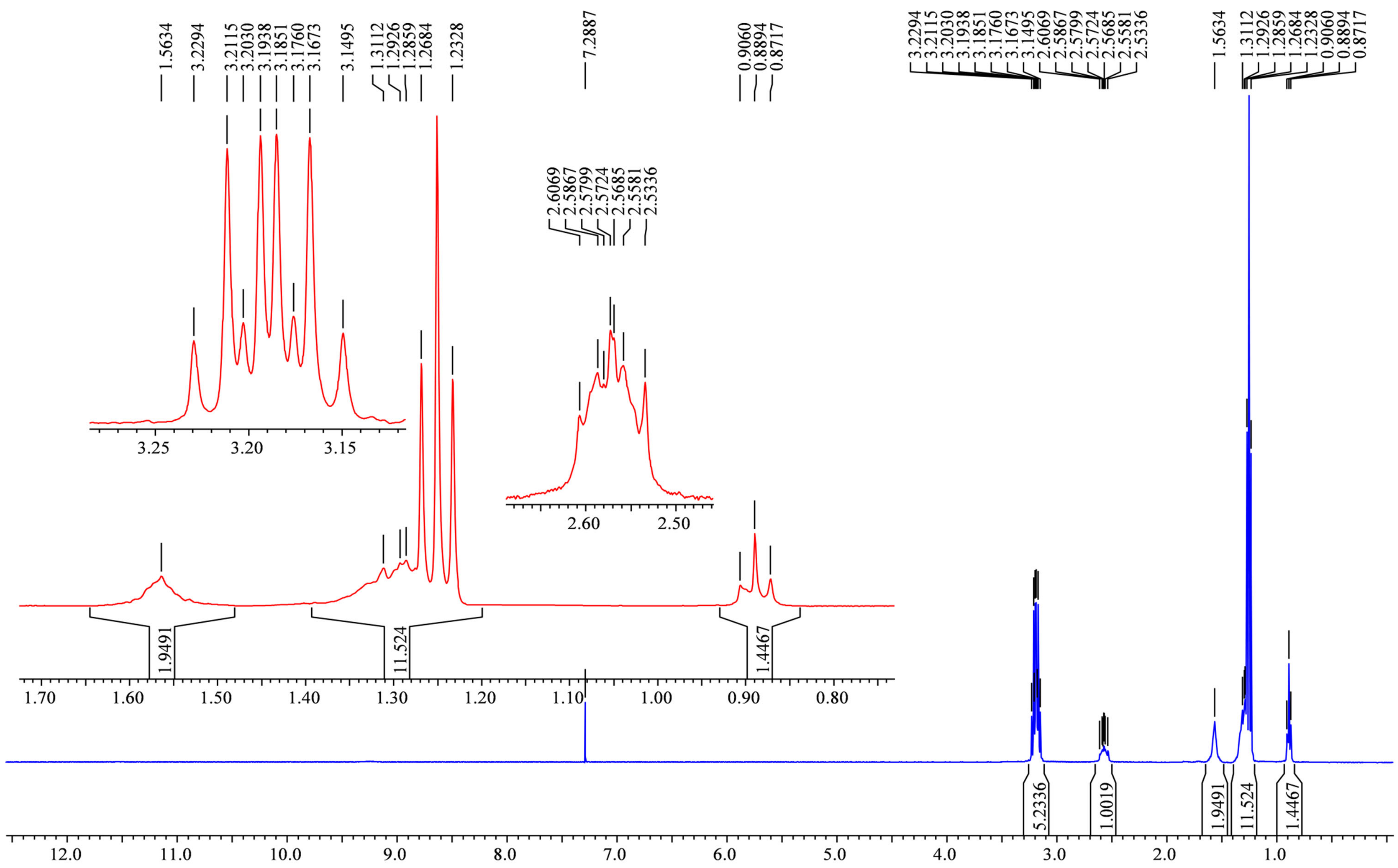

Figure 6S. ^{1}H NMR spectrum (400.0 MHz, $CDCl_3$) of $(Et_2N)_3P^+–C_8H_{17}\ I^–$ (**4b**).

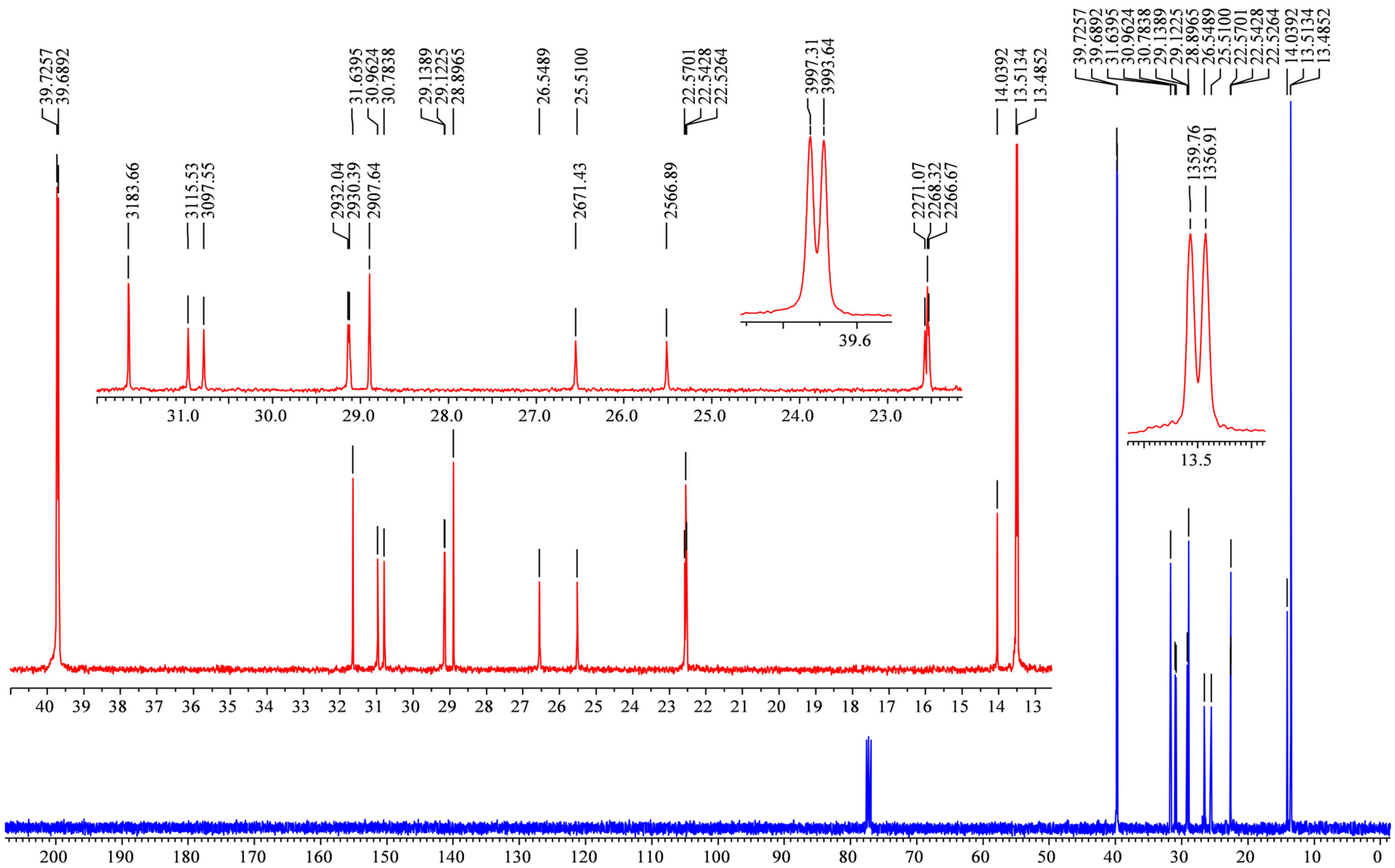

Figure 7S. ^{13}C-{^1H} NMR spectrum (100.6 MHz, $CDCl_3$) of $(Et_2N)_3P^+–C_8H_{17}$ $I^–$ (**4b**).

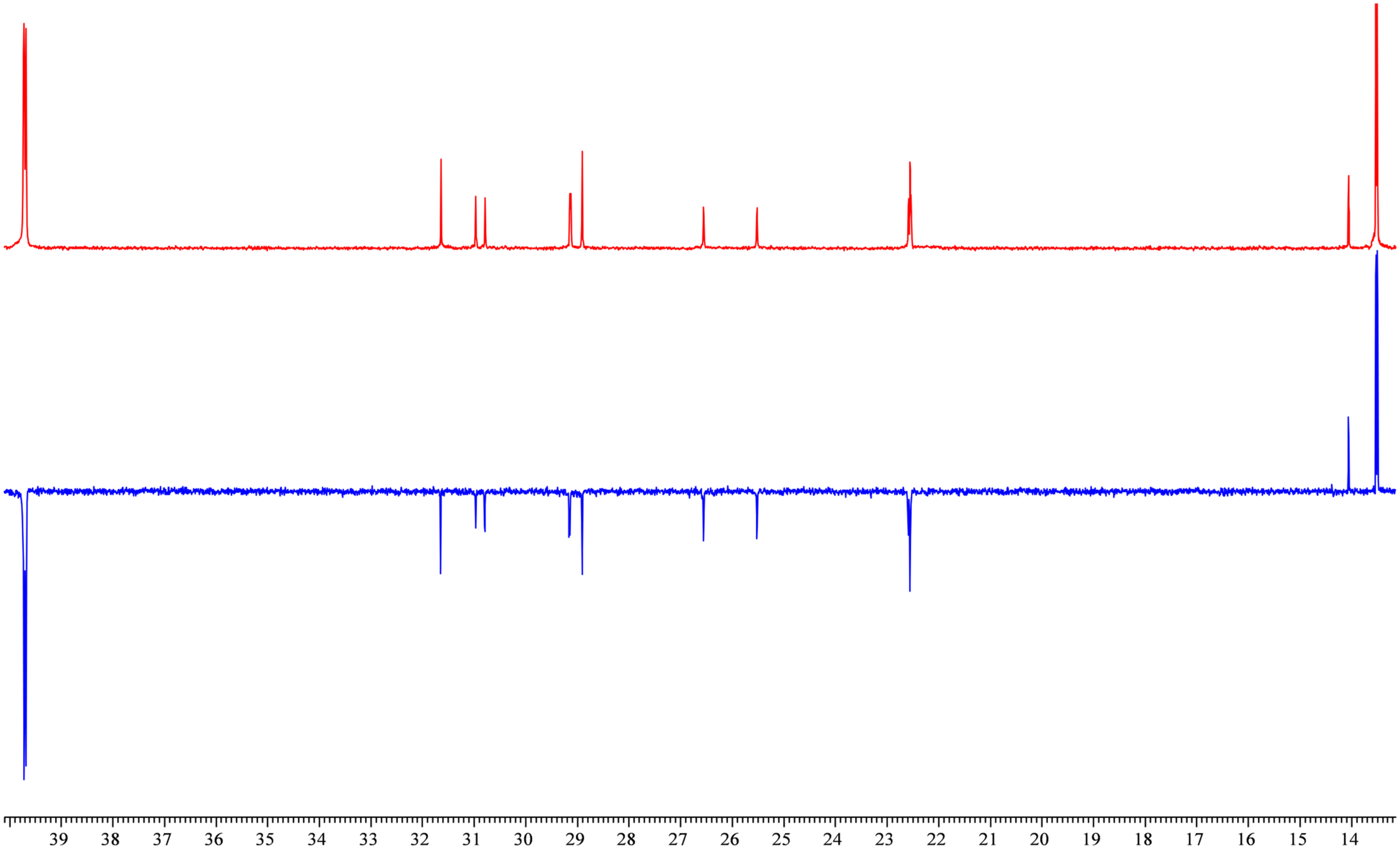

Figure 8S. ^{13}C-{^1H} and ^{13}C-{^1H}-dept NMR spectra (100.6 MHz, $CDCl_3$) of $(Et_2N)_3P^+–C_8H_{17}\ I^–$ (**4b**).

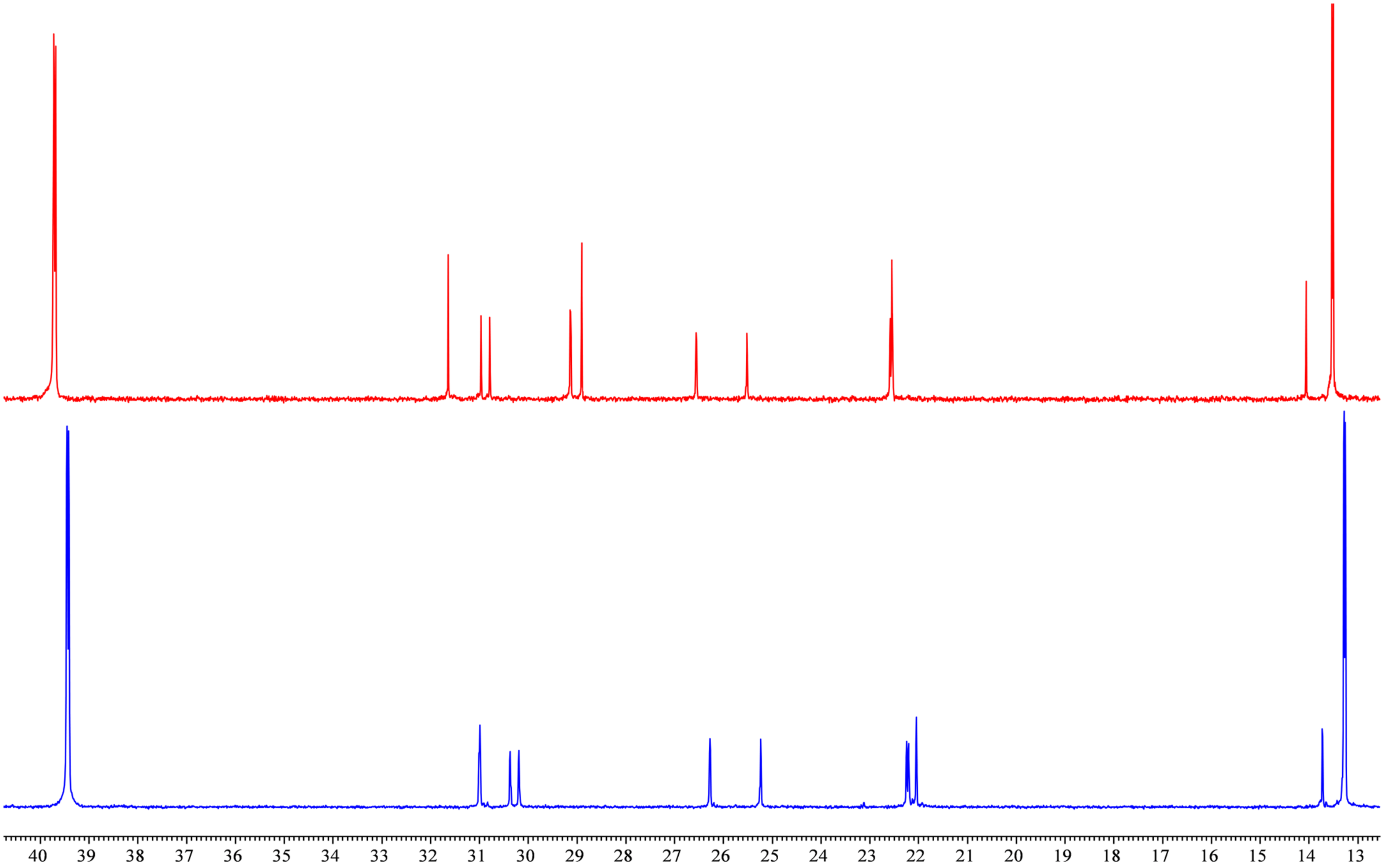

Figure 9S. ^{13}C-{^{1}H} NMR spectra (400.0 MHz, $CDCl_3$) of $(Et_2N)_3P^+–C_8H_{17}$ $I^–$ (**4b**, red) and $(Et_2N)_3P^+–C_6H_{13}$ $I^–$ (**4a**, blue).

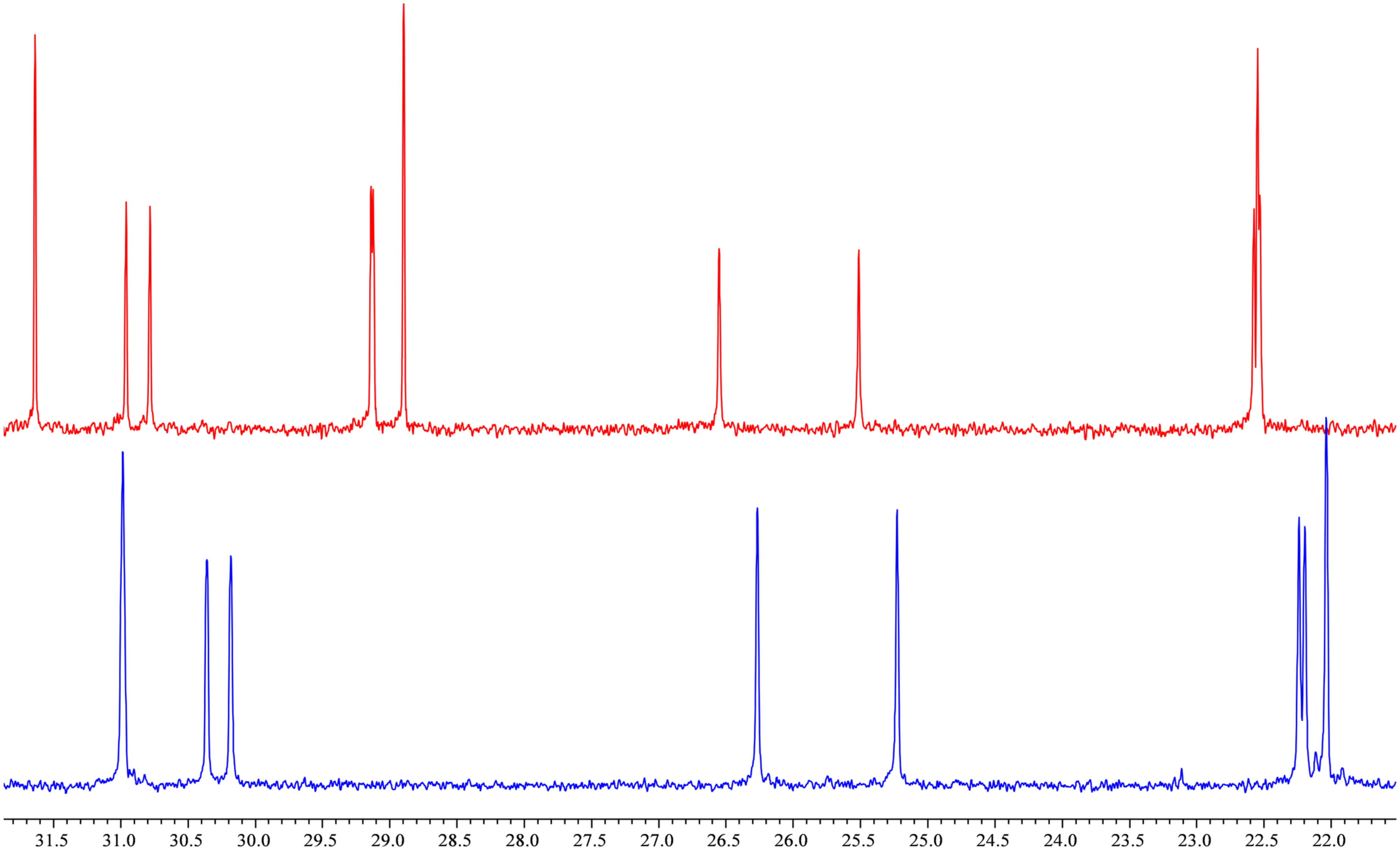

Figure 10S. ^{13}C-{^{1}H} NMR spectra (400.0 MHz, $CDCl_3$) of $(Et_2N)_3P^+–C_8H_{17}$ $I^−$ (**4b**, red) and $(Et_2N)_3P^+–C_6H_{13}$ $I^−$ (**4a**, blue).

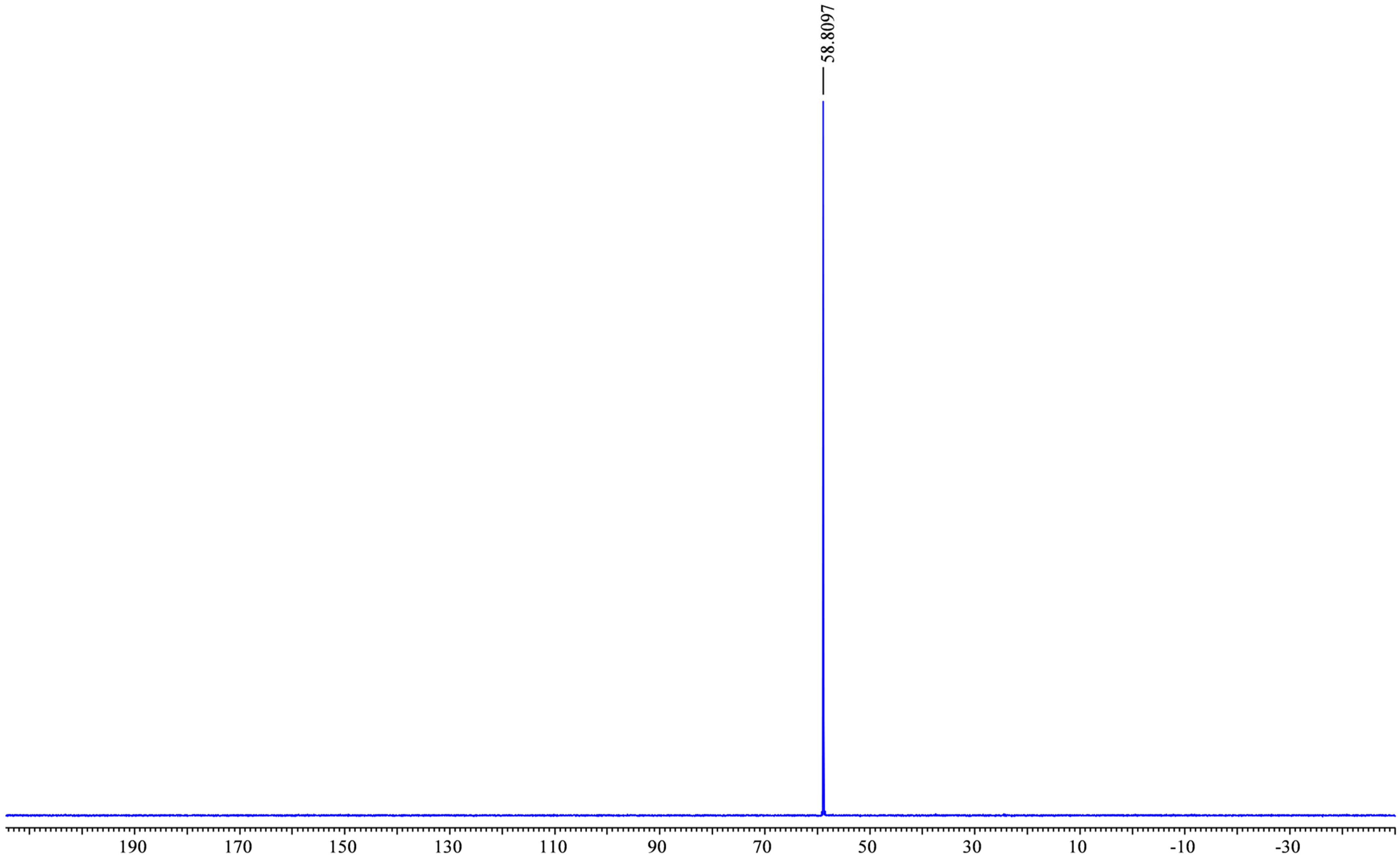

Figure 11S. ^{31}P-{^{1}H} NMR spectrum (400.0 MHz, $CDCl_3$) of $(Et_2N)_3P^+–C_9H_{19}\ I^–$ (**4c**).

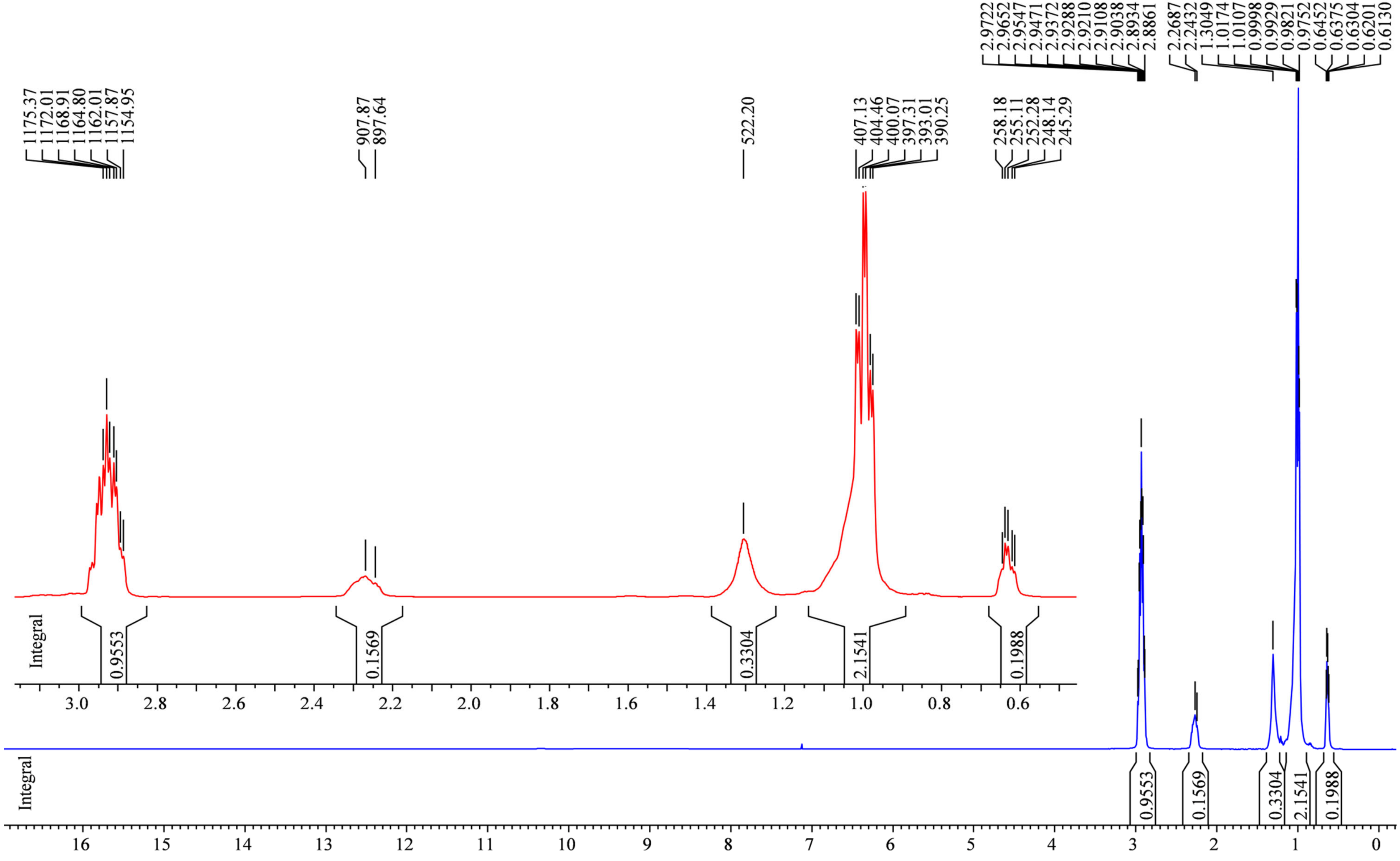

Figure 12S. ^{1}H NMR spectrum (400.0 MHz, $CDCl_3$) of $(Et_2N)_3P^+–C_9H_{19}$ $I^–$ (**4c**).

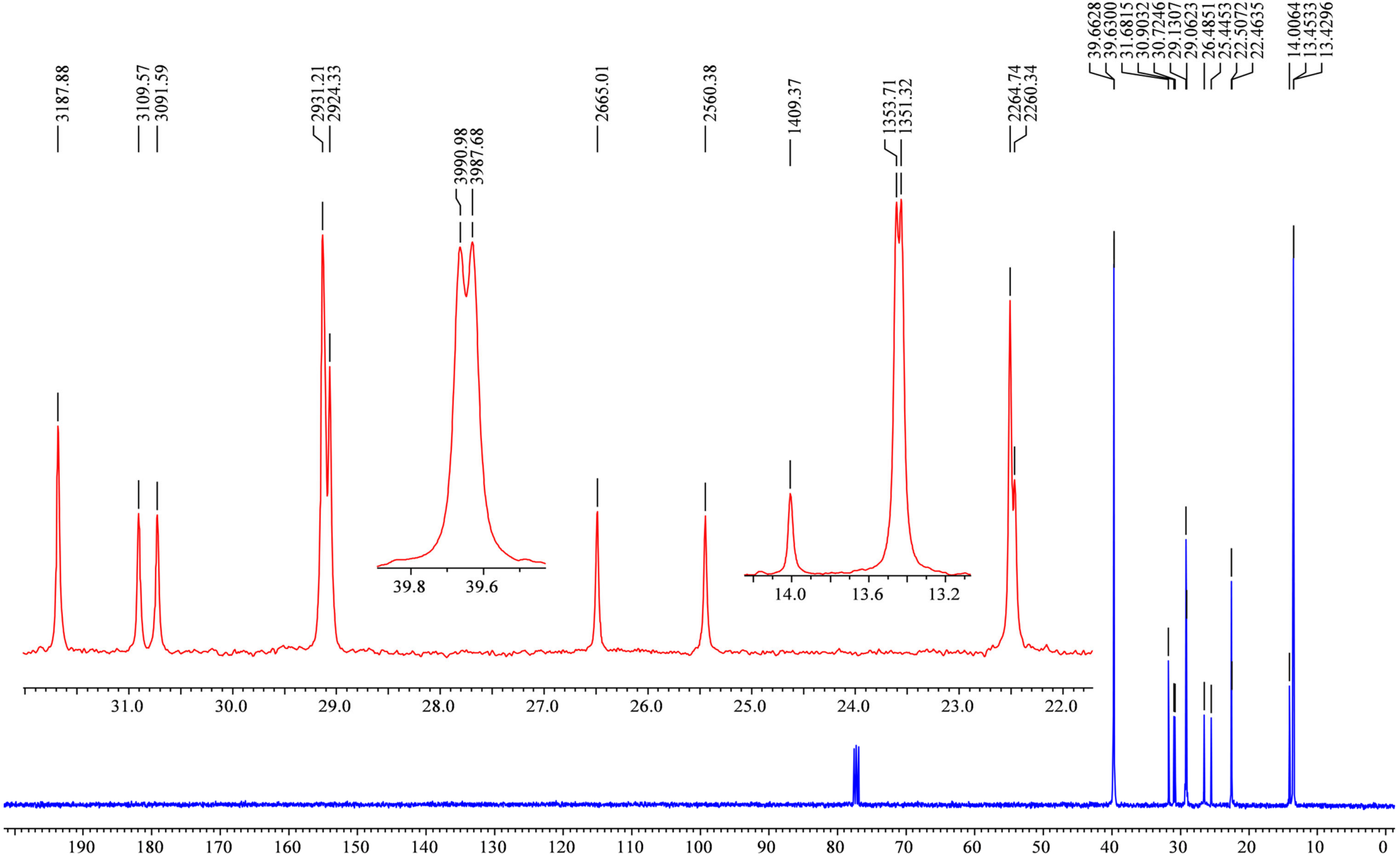

Figure 13S. ^{13}C-{^{1}H} NMR spectrum (100.6 MHz, $CDCl_3$) of $(Et_2N)_3P^+–C_9H_{19}\ I^–$ (**4c**). `

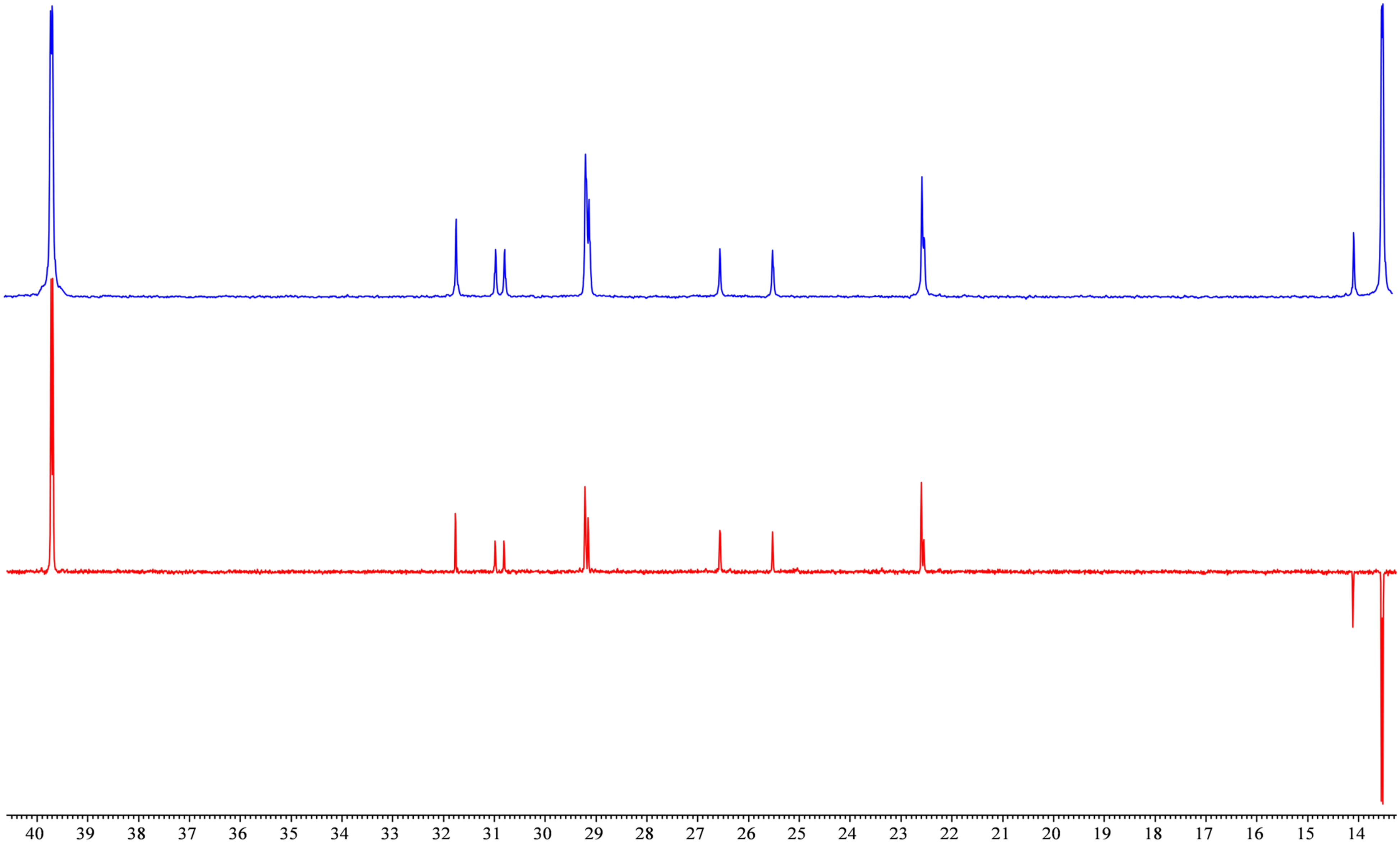

Figure 14S. ^{13}C-{^{1}H} and ^{13}C-{^{1}H}-dept NMR spectra (100.6 MHz, $CDCl_3$) of $(Et_2N)_3P^+–C_9H_{19}\ I^-$ (**4c**).

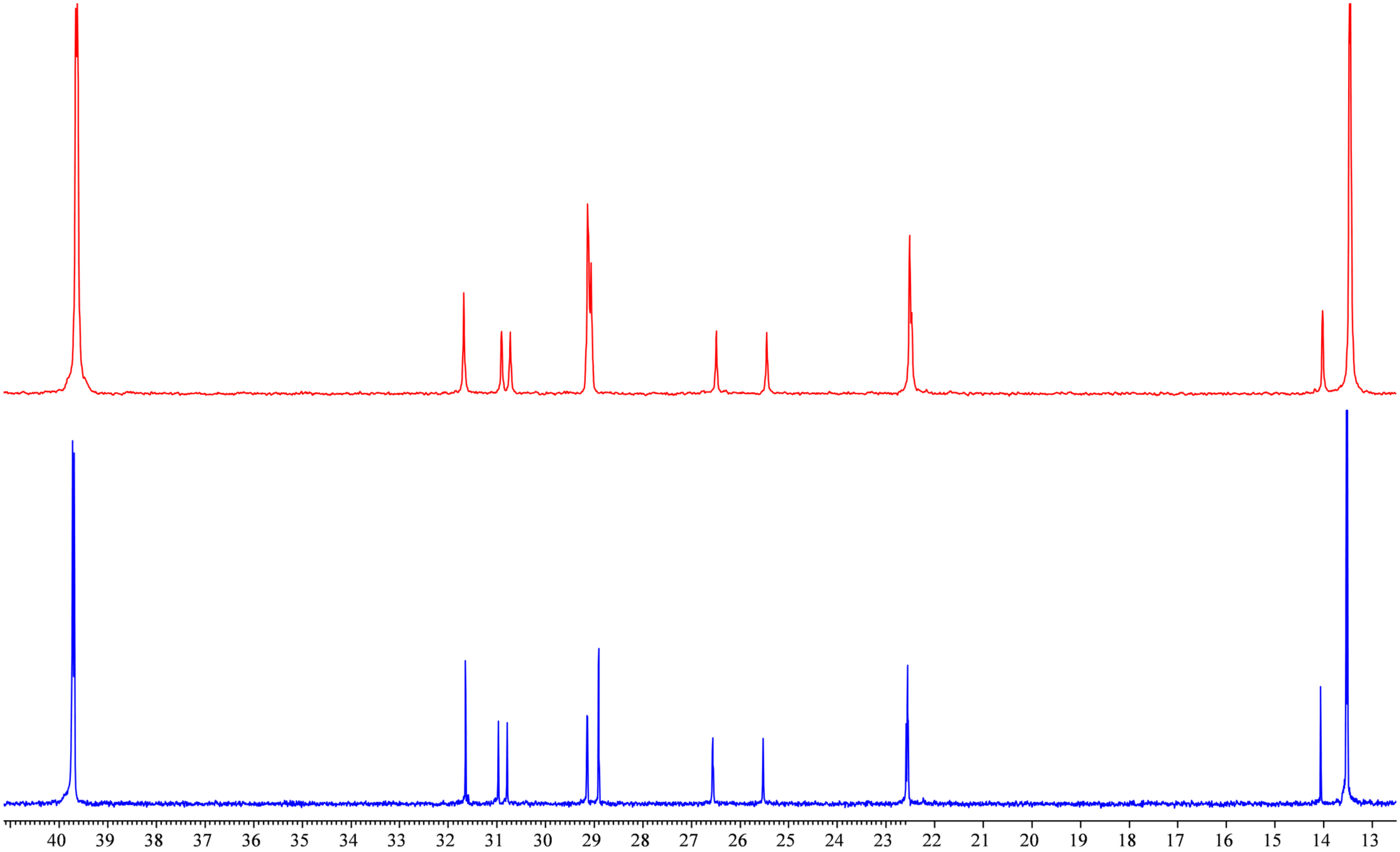

Figure 15S. ^{13}C-{^{1}H} NMR spectra (400.0 MHz, $CDCl_3$) of $(Et_2N)_3P^+$–C_9H_{19} I^- (**4c**, red) and $(Et_2N)_3P^+$–C_8H_{17} I^- (**4b**, blue).

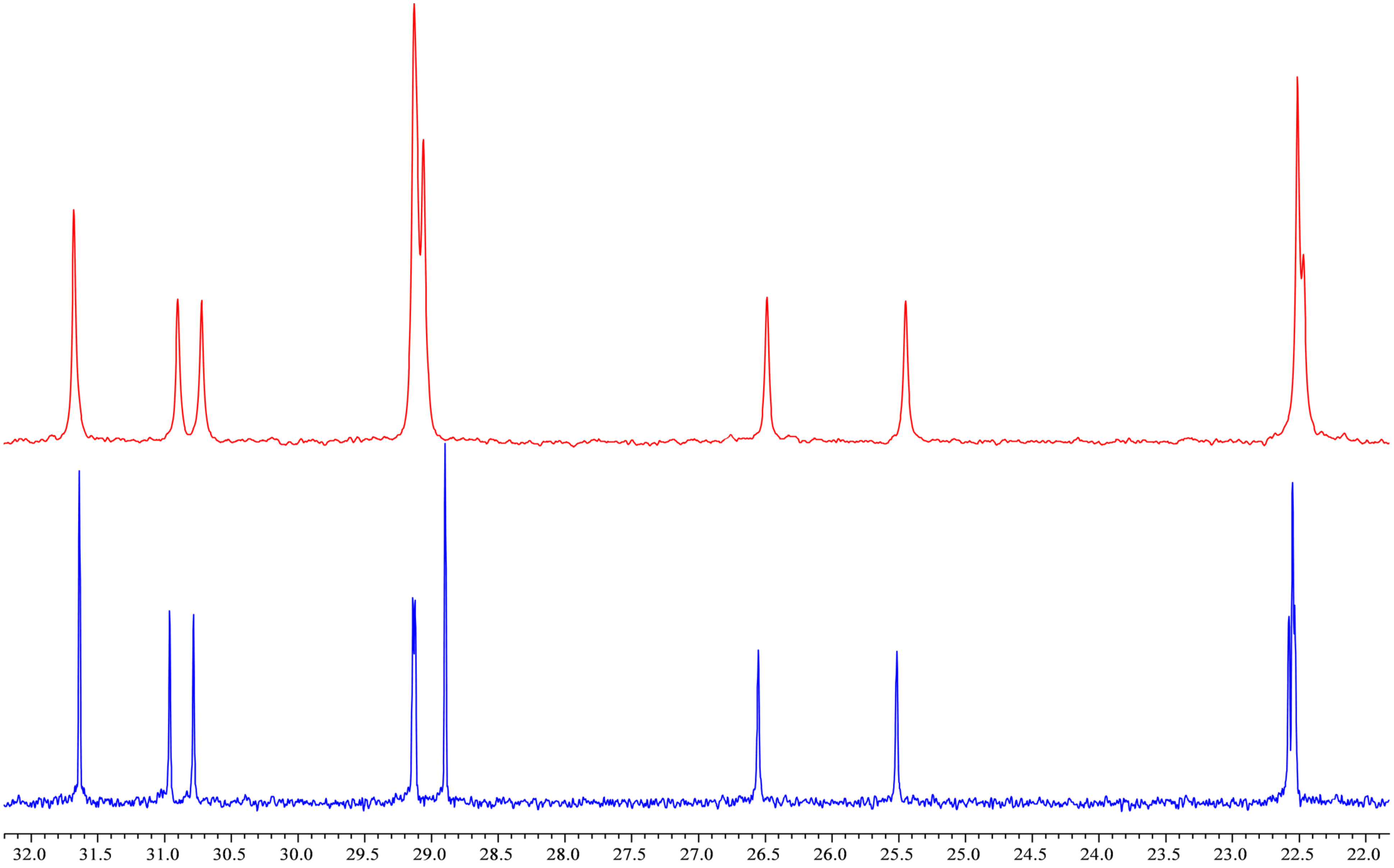

Figure 16S. ^{13}C-{^{1}H} NMR spectra (400.0 MHz, $CDCl_3$) of $(Et_2N)_3P^+–C_9H_{19}\ I^−$ (**4c**, red) and $(Et_2N)_3P^+–C_8H_{17}\ I^−$ (**4b**, blue).

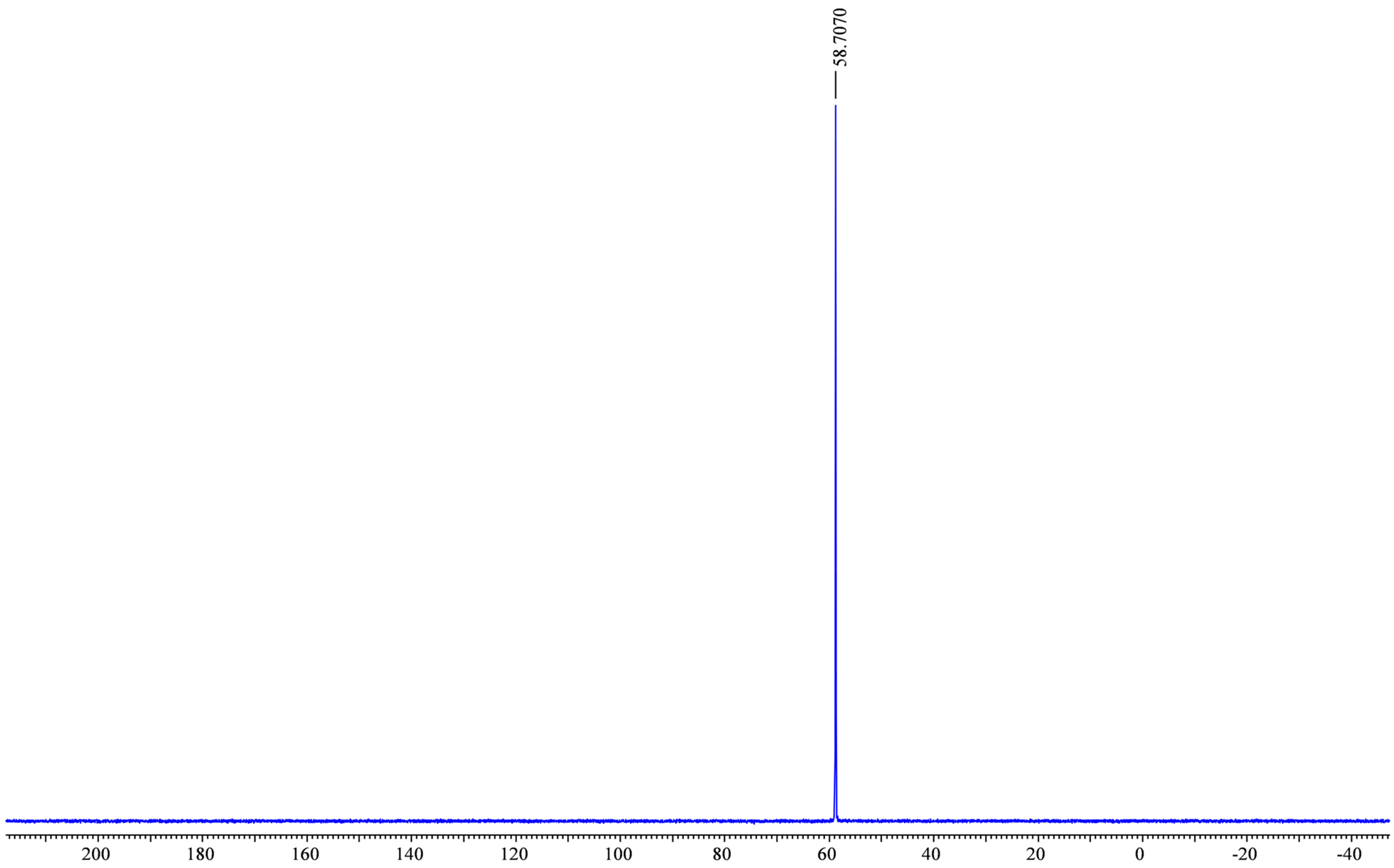

Figure 17S. ^{31}P-{^{1}H} NMR spectrum (400.0 MHz, $CDCl_3$) of $(Et_2N)_3P^+–C_{10}H_{21}\ I^−$ (**4d**).

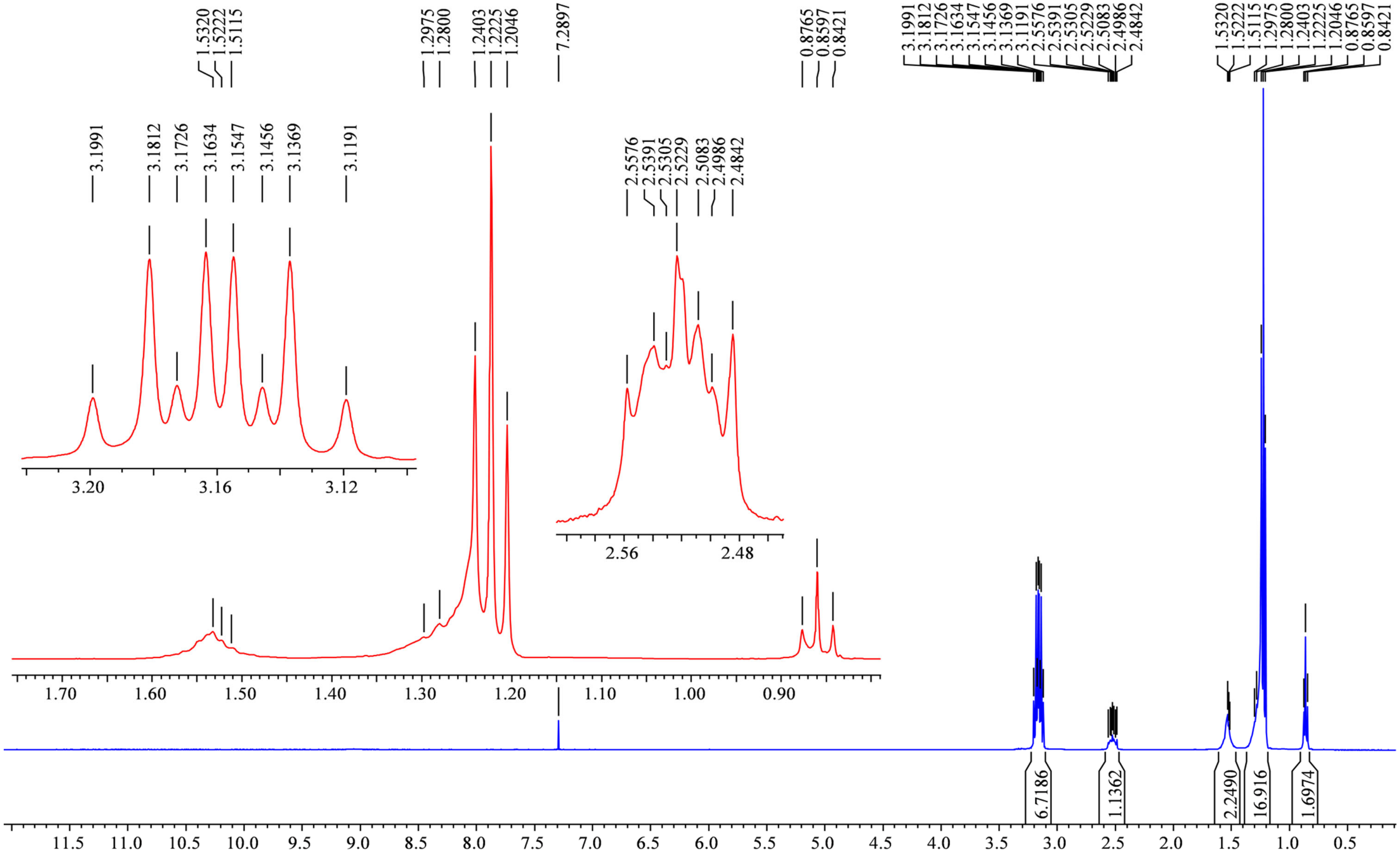

Figure 18S. ^{1}H NMR spectrum (400.0 MHz, $CDCl_3$) of $(Et_2N)_3P^+–C_{10}H_{21}$ $I^–$ (**4d**).

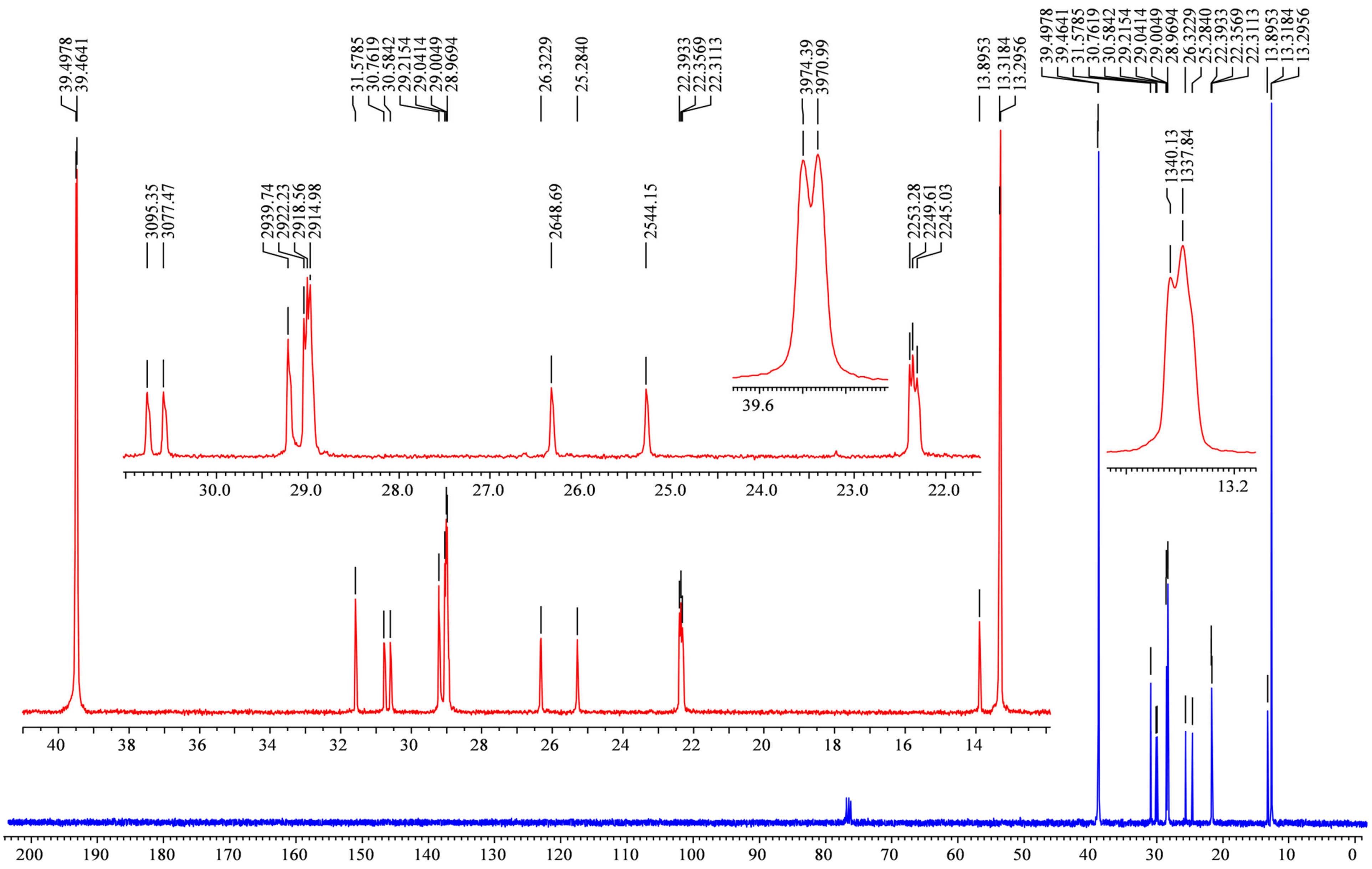

Figure 19S. ^{13}C-{^{1}H} NMR spectrum (100.6 MHz, $CDCl_3$) of $(Et_2N)_3P^{+}–C_{10}H_{21}\ I^{–}$ (**4d**).

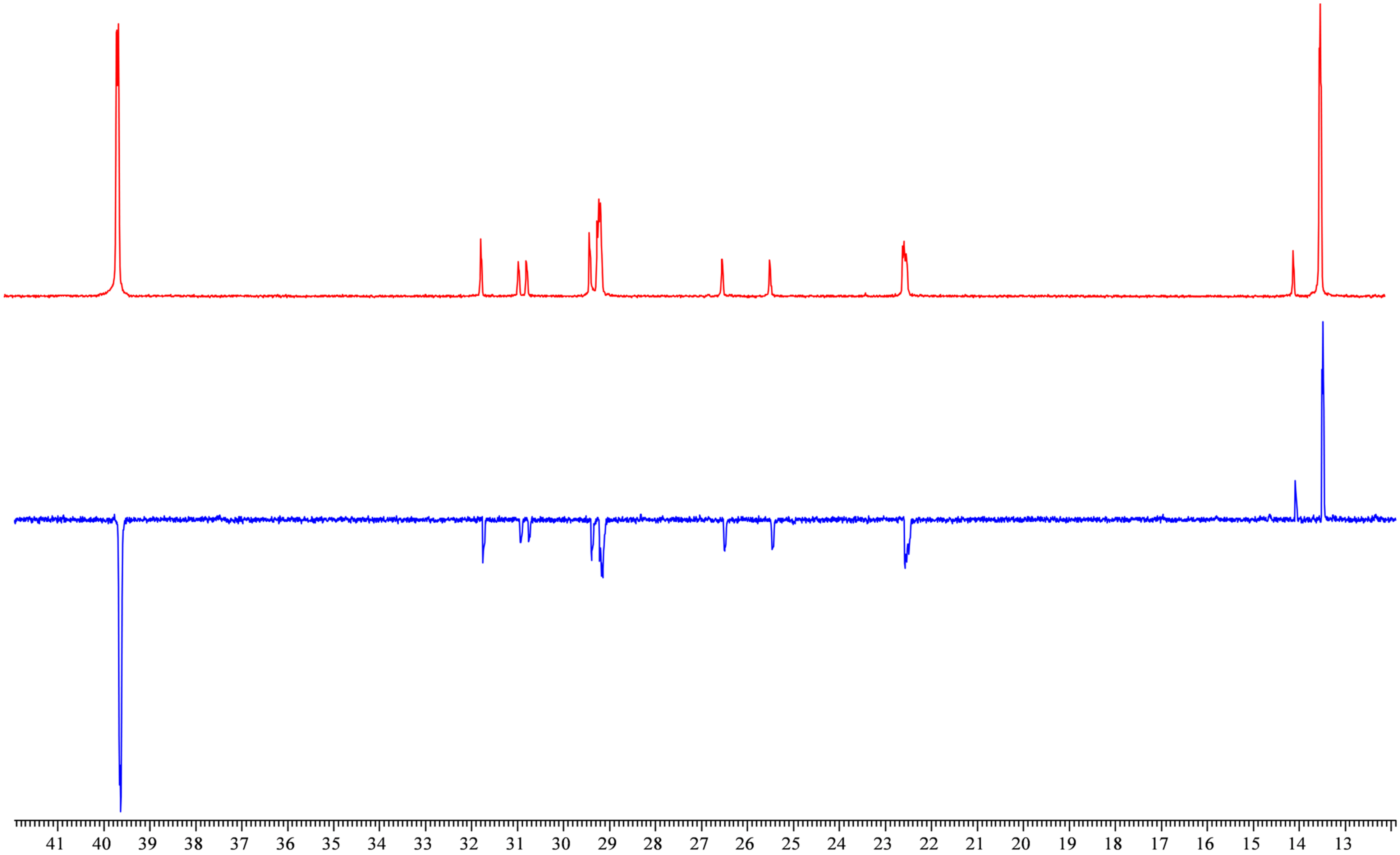

Figure 20S. ^{13}C-{^{1}H} and ^{13}C-{^{1}H}-dept NMR spectra (100.6 MHz, $CDCl_3$) of $(Et_2N)_3P^+–C_{10}H_{21}\ I^-$ (**4d**).

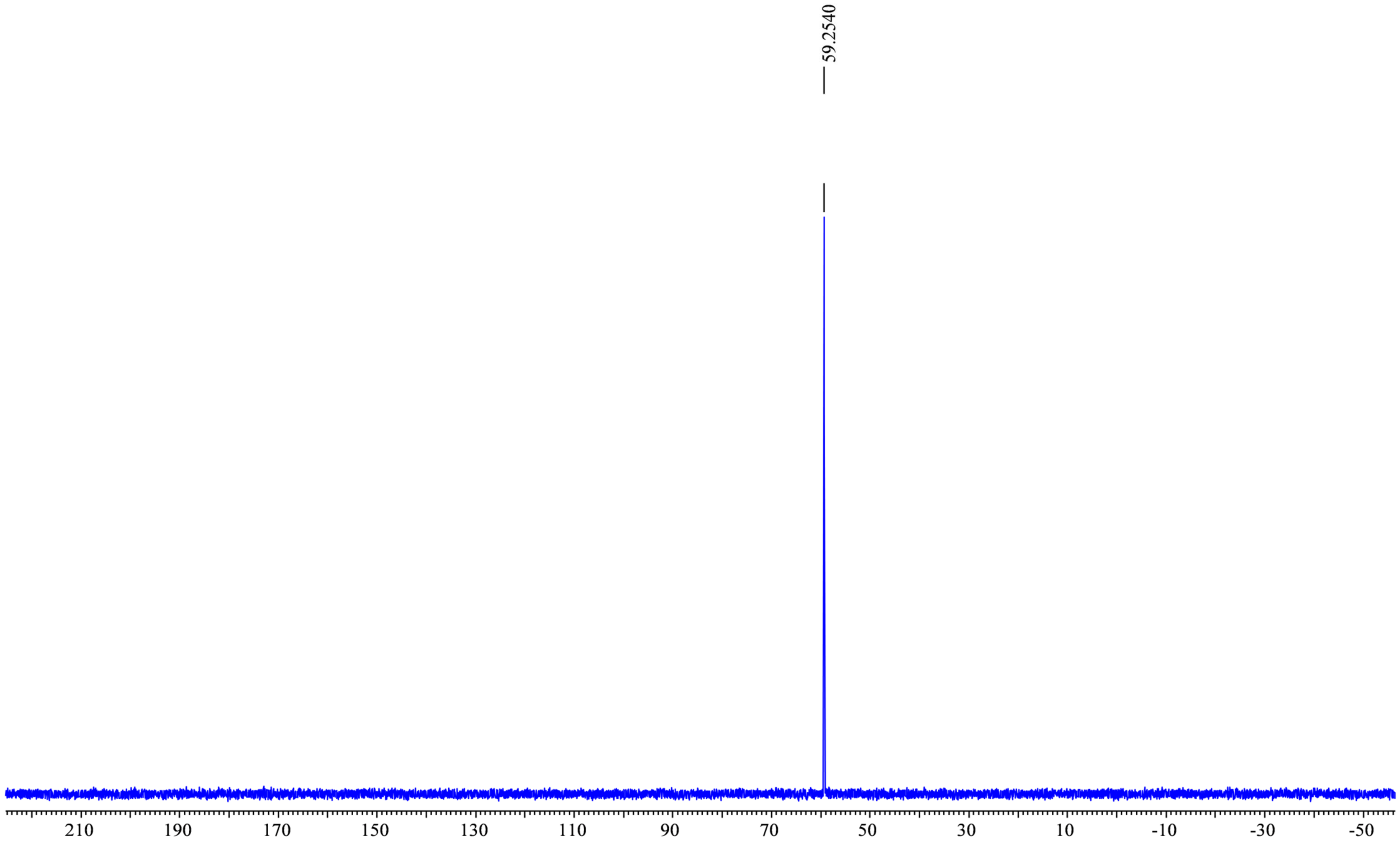

Figure 21S. ^{31}P-{^{1}H} NMR spectrum (162.0 MHz, $CDCl_3$) of $(Et_2N)_3P^+$–$C_{14}H_{29}$ Br^- (**4e**).

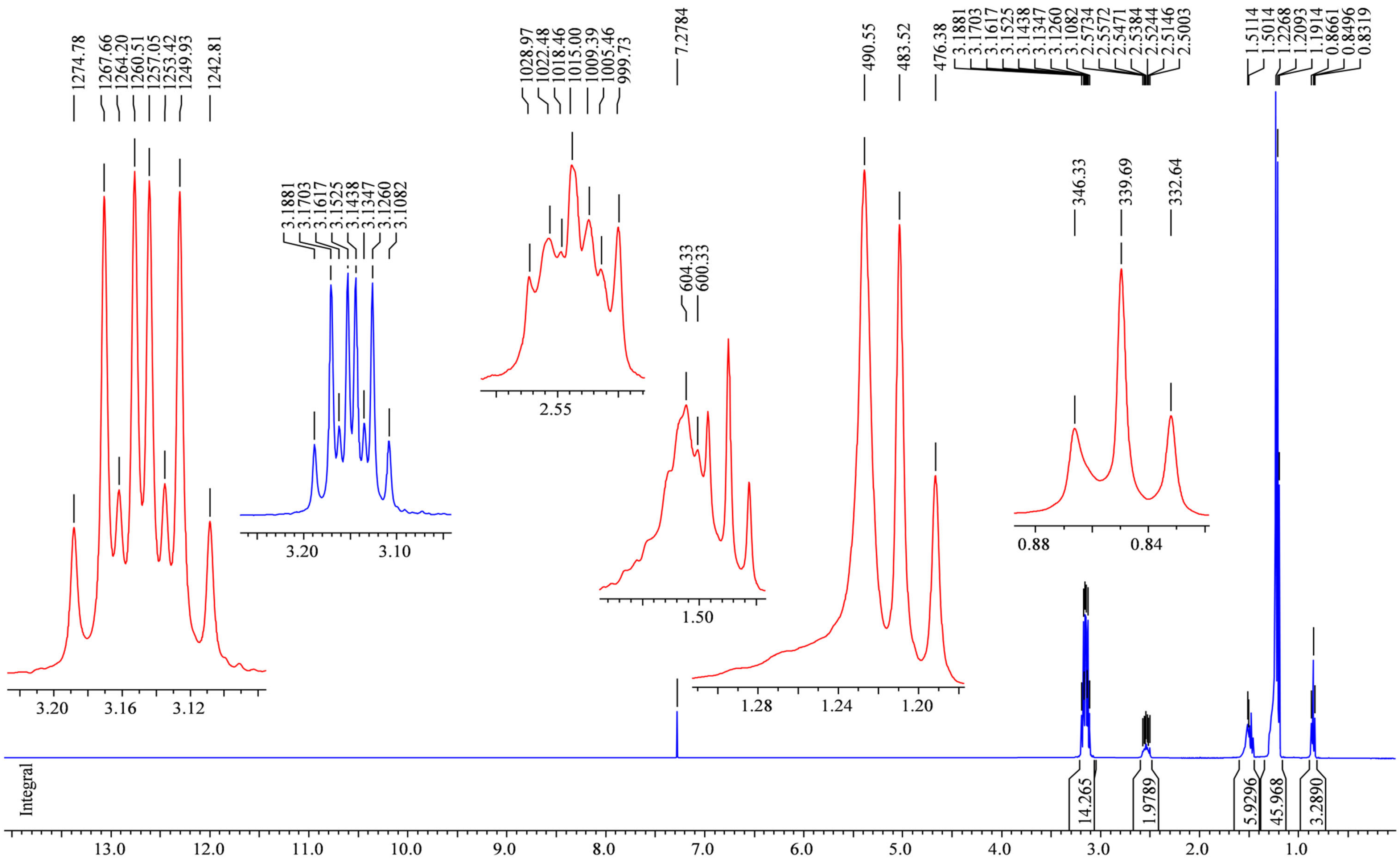

Figure 22S. ^{1}H NMR spectrum (400.0 MHz, $CDCl_3$) of $(Et_2N)_3P^+–C_{14}H_{29}\ Br^-$ (**4e**).

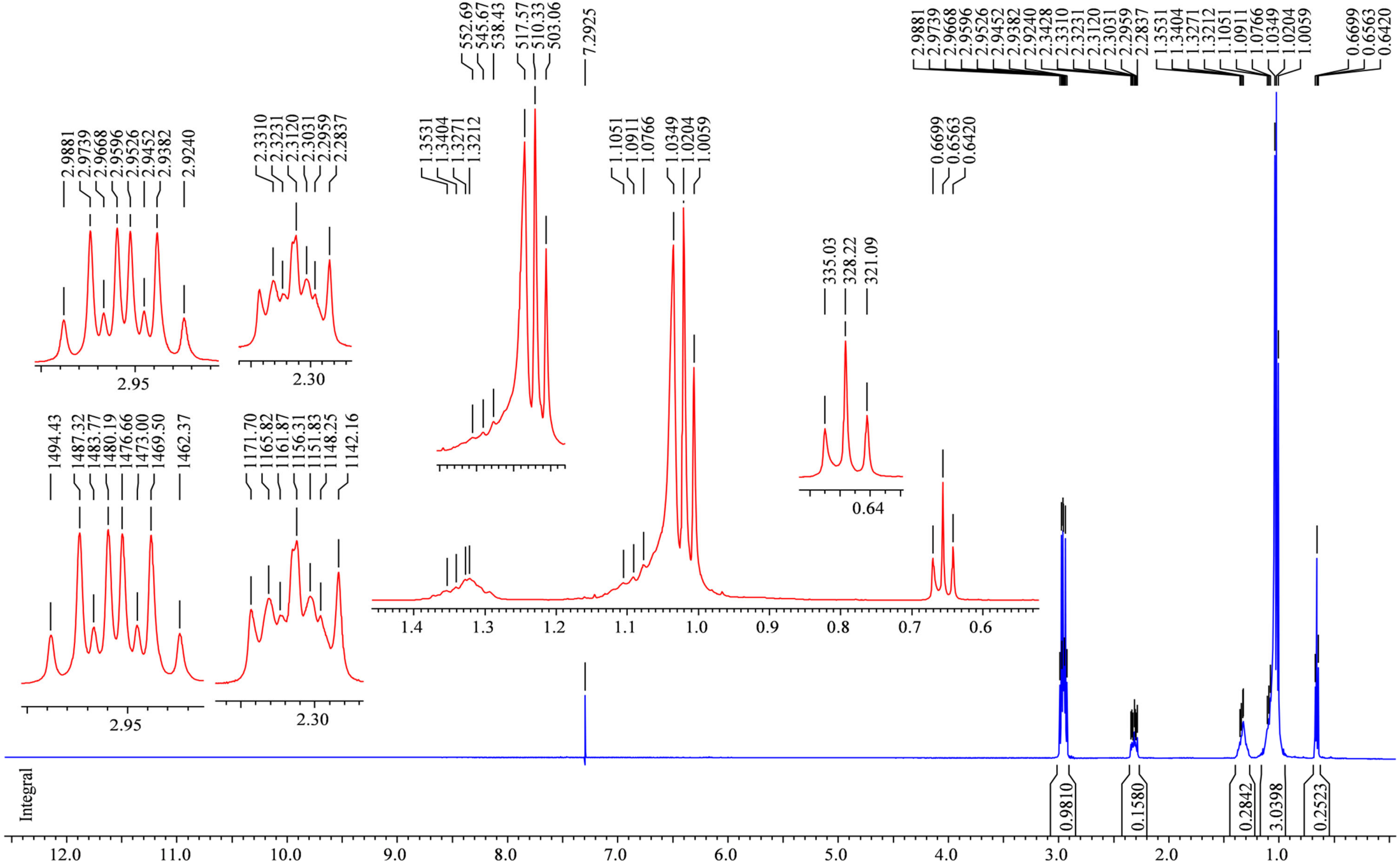

Figure 23S. ^{1}H NMR spectrum (500.0 MHz, $CDCl_3$) of $(Et_2N)_3P^+–C_{14}H_{29}$ Br^- (**4e**).

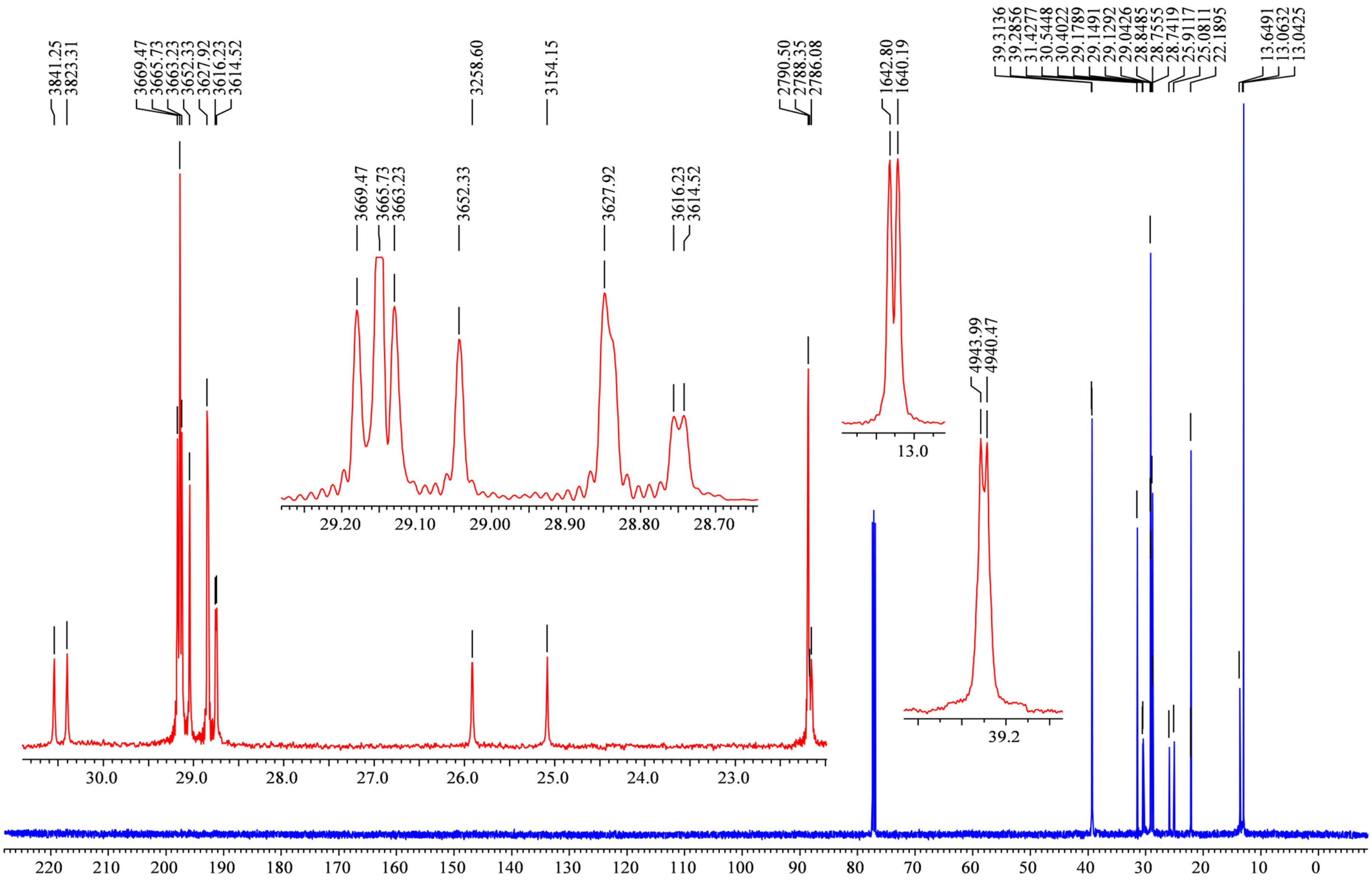

Figure 24S. ^{13}C-{^{1}H} NMR spectrum (125.0 MHz, $CDCl_3$) of $(Et_2N)_3P^+–C_{14}H_{29}\ Br^–$ (**4e**).

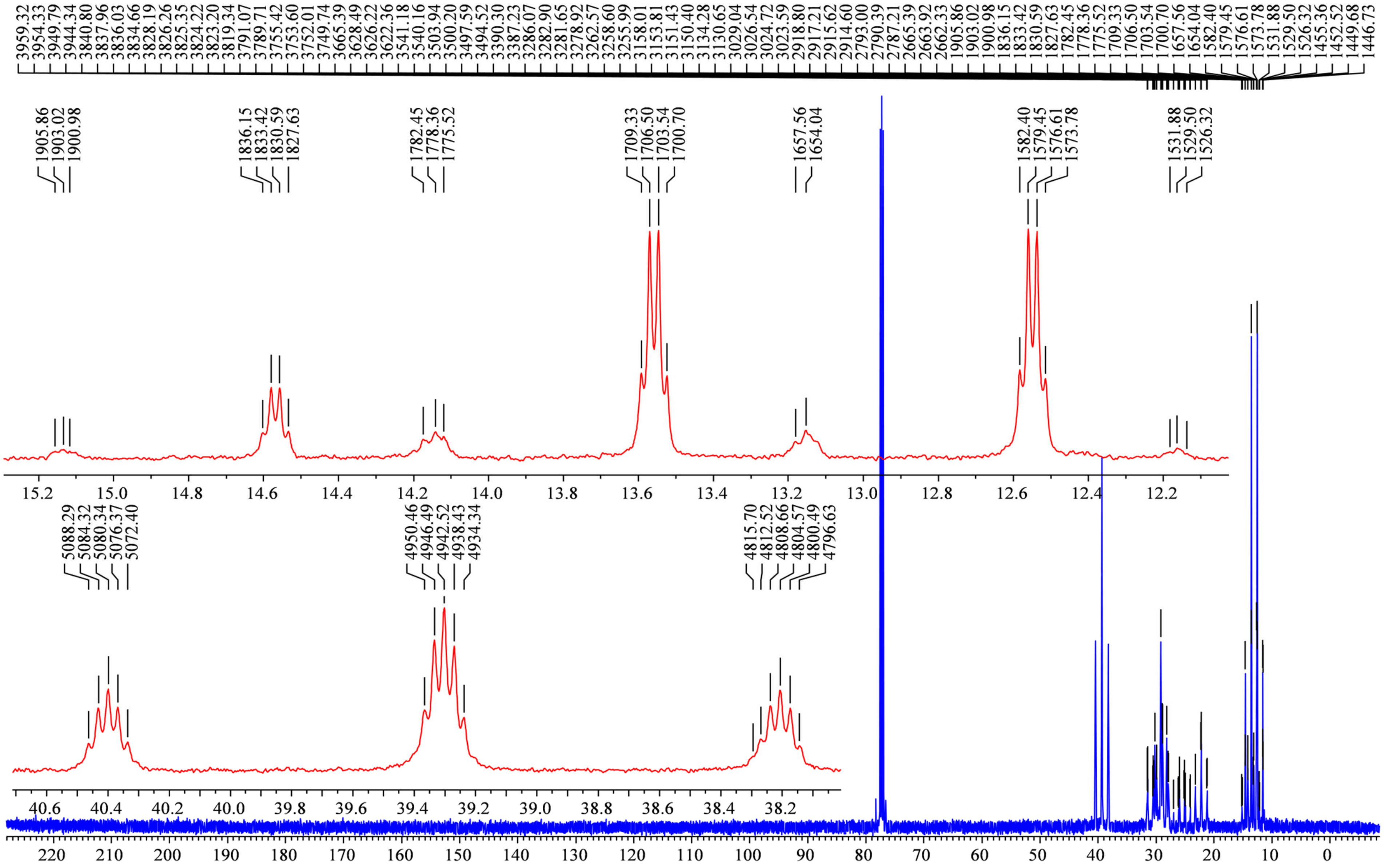

Figure 25S. ^{13}C NMR spectrum (125.0 MHz, $CDCl_3$) of $(Et_2N)_3P^+–C_{14}H_{29}$ $Br^–$ (**4e**).

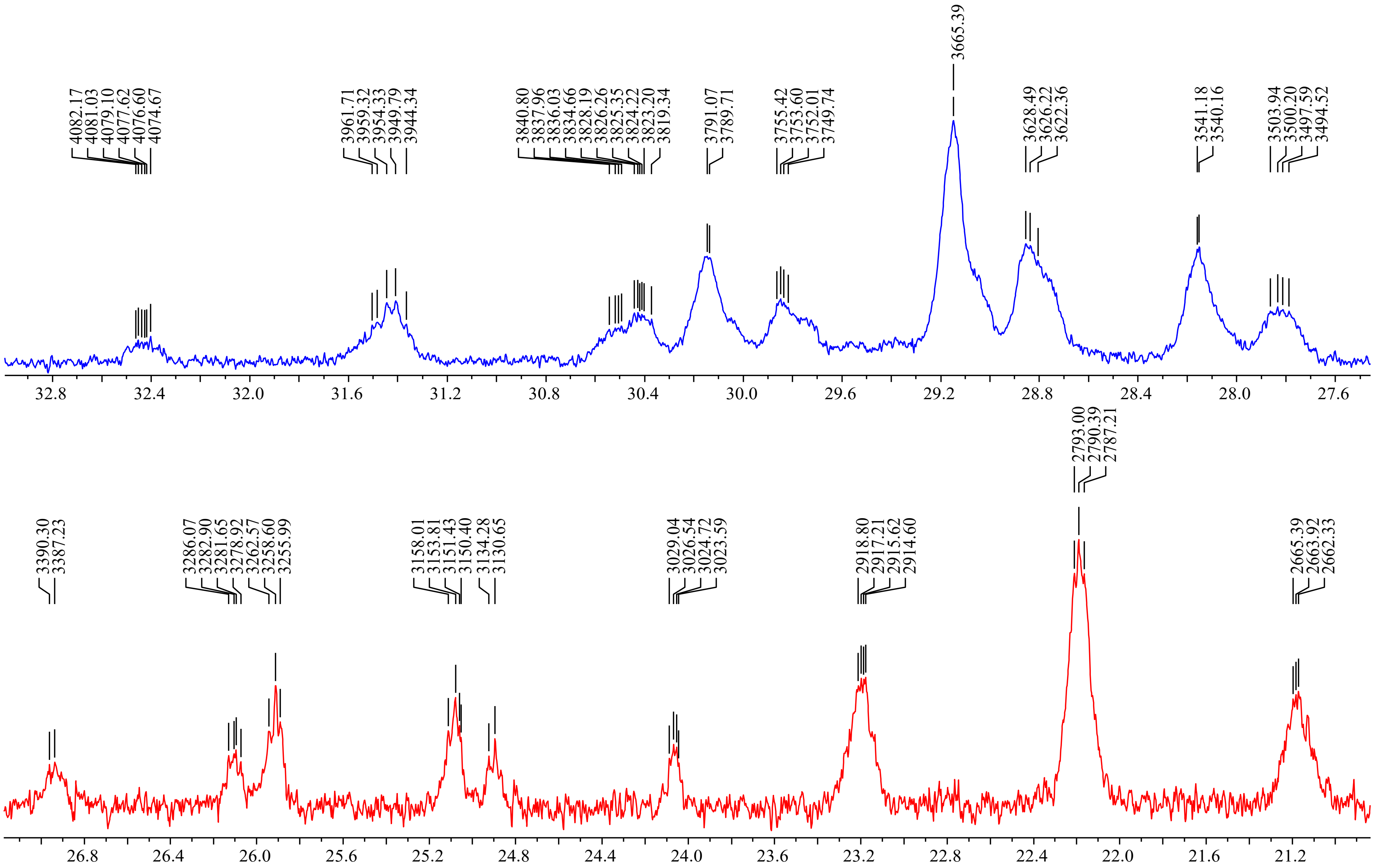

Figure 26S. High-field fragments of ^{13}C NMR spectrum (125.0 MHz, $CDCl_3$) of $(Et_2N)_3P^+–C_{14}H_{29}$ Br^- (**4e**).

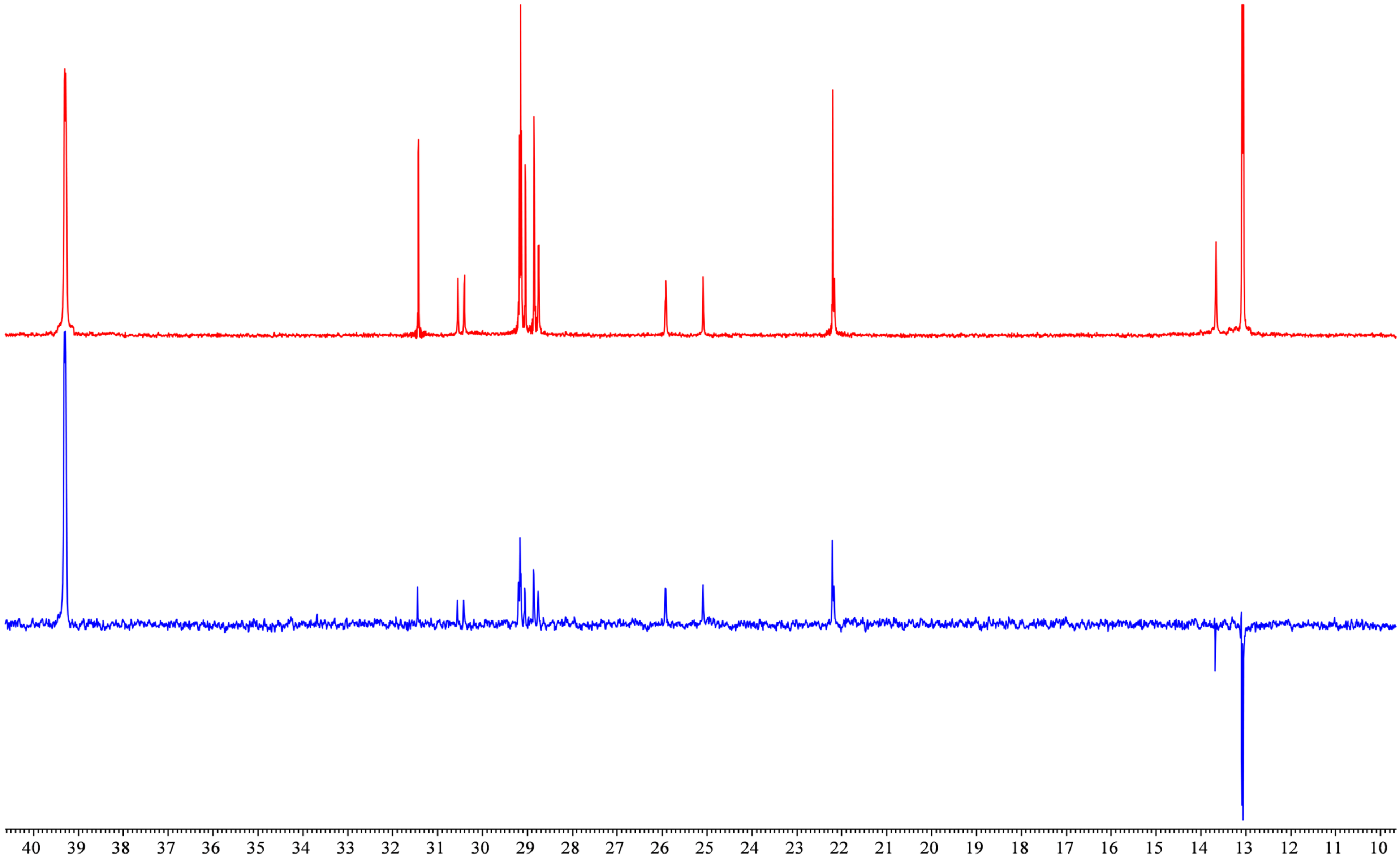

Figure 27S. ^{13}C-{^{1}H} and ^{13}C-{^{1}H}-dept NMR spectra (125.0 MHz, $CDCl_3$) of $(Et_2N)_3P^+–C_{14}H_{29}$ $Br^–$ (**4e**).

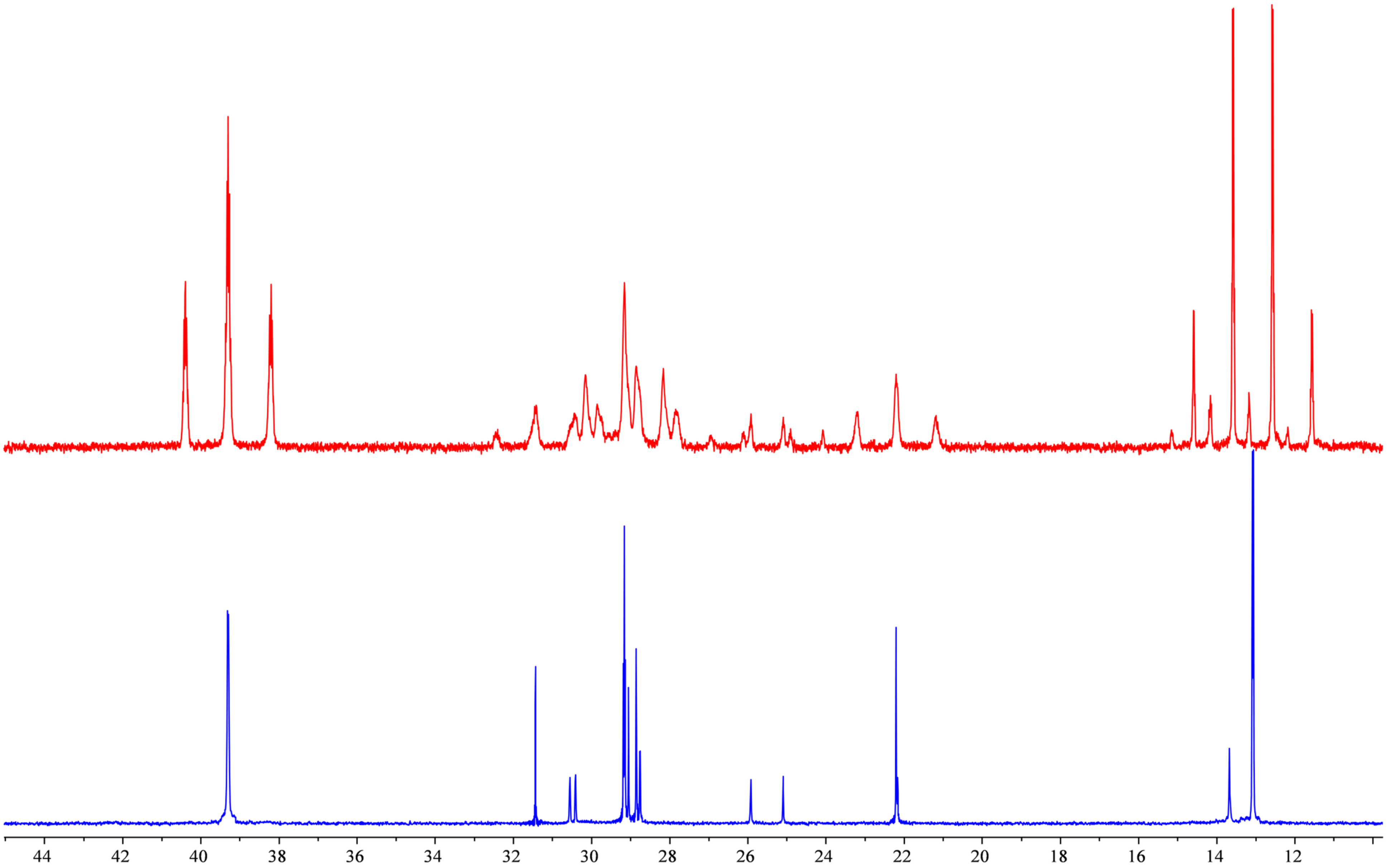

Figure 28S. ^{13}C-{^{1}H} and ^{13}C NMR spectra (125.0 MHz, $CDCl_3$) of $(Et_2N)_3P^+–C_{14}H_{29}$ $Br^–$ (**4e**).

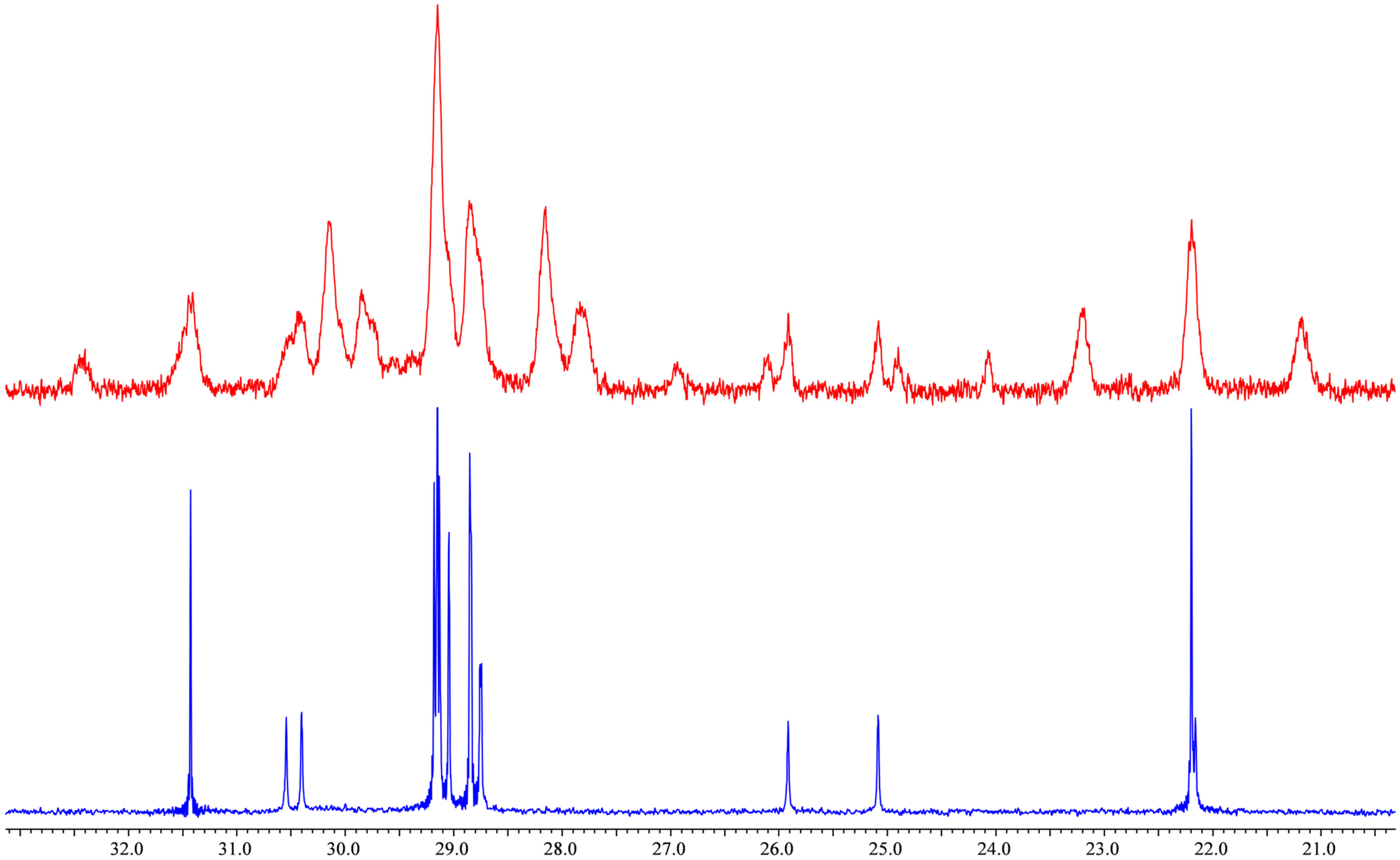

Figure 29S. High-field fragments of ^{13}C-{^{1}H} and ^{13}C NMR spectra (125.0 MHz, $CDCl_3$) of $(Et_2N)_3P^+–C_{14}H_{29}\ Br^-$ (**4e**).

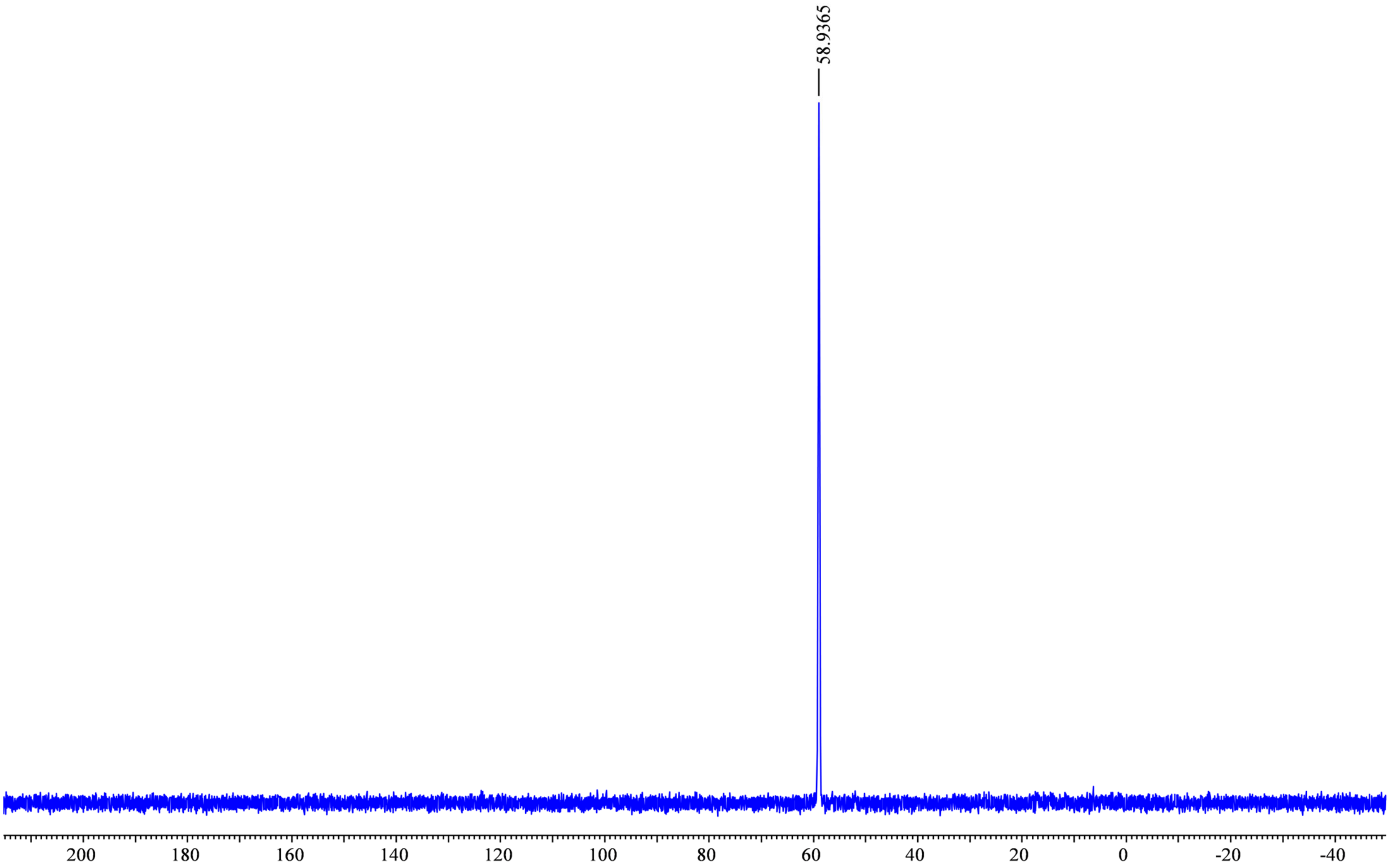

Figure 30S. ^{31}P-{^{1}H} NMR spectrum (162.0 MHz, $CDCl_3$) of $(Et_2N)_2(Ph)P^{+}$–C_6H_{13} I^{-} (**5a**).

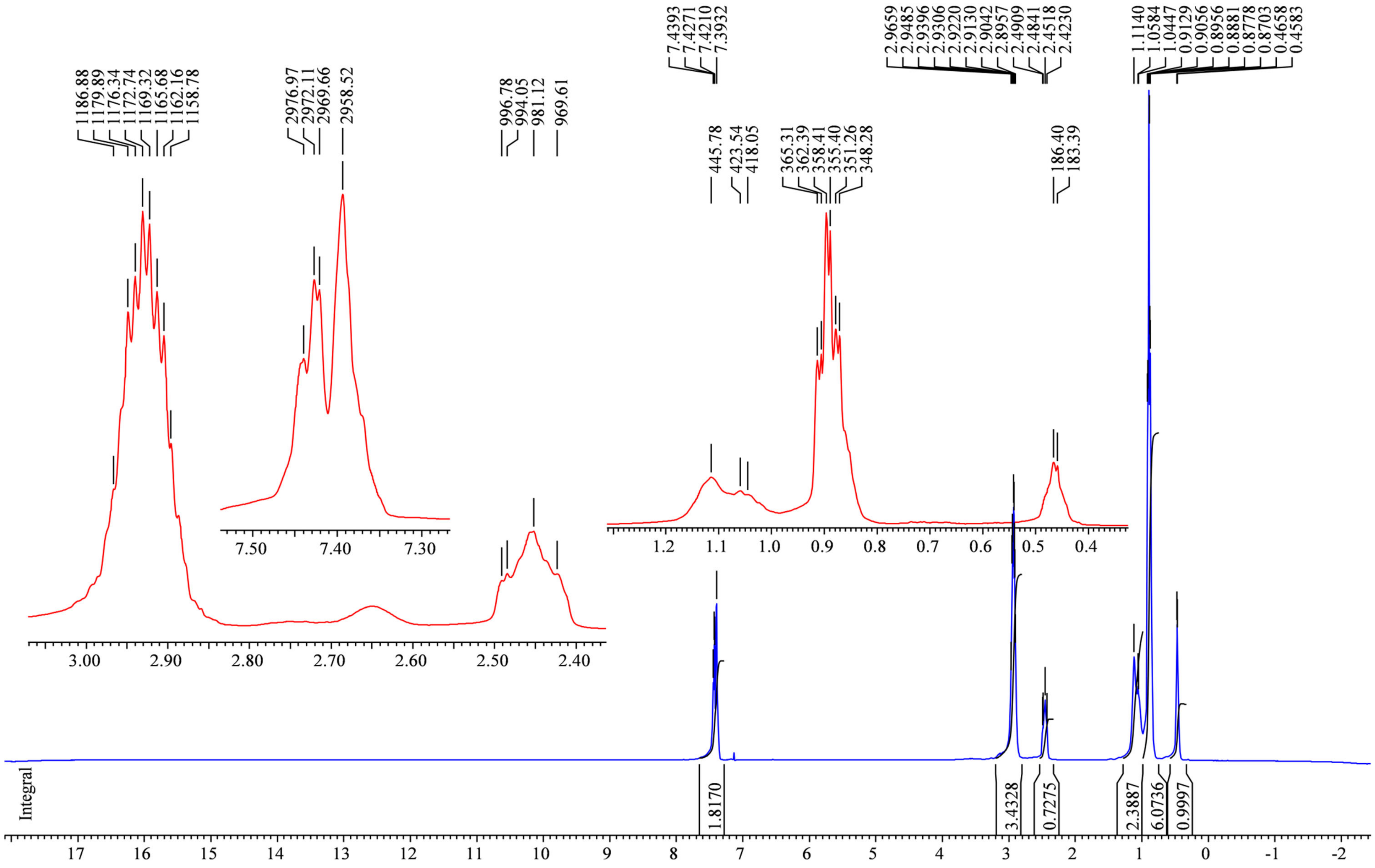

Figure 31S. ^{1}H NMR spectrum (400.0 MHz, $CDCl_3$) of $(Et_2N)_2(Ph)P^+–C_6H_{13}\ I^-$ (**5a**).

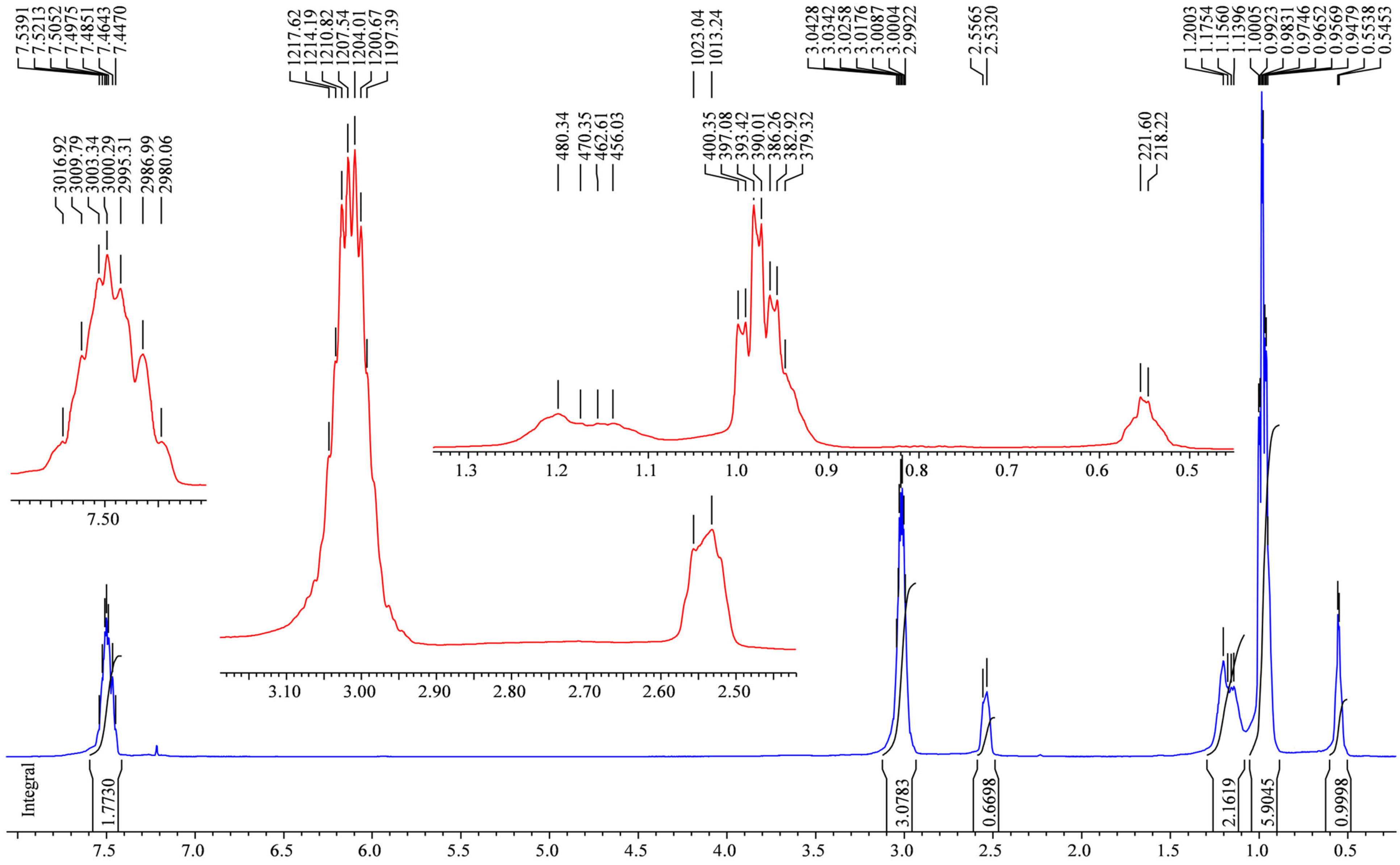

Figure 32S. High-field fragment of ^{1}H NMR spectrum (400.0 MHz, $CDCl_3$) of $(Et_2N)_2(Ph)P^{+}–C_6H_{13}$ $I^{–}$ (**5a**).

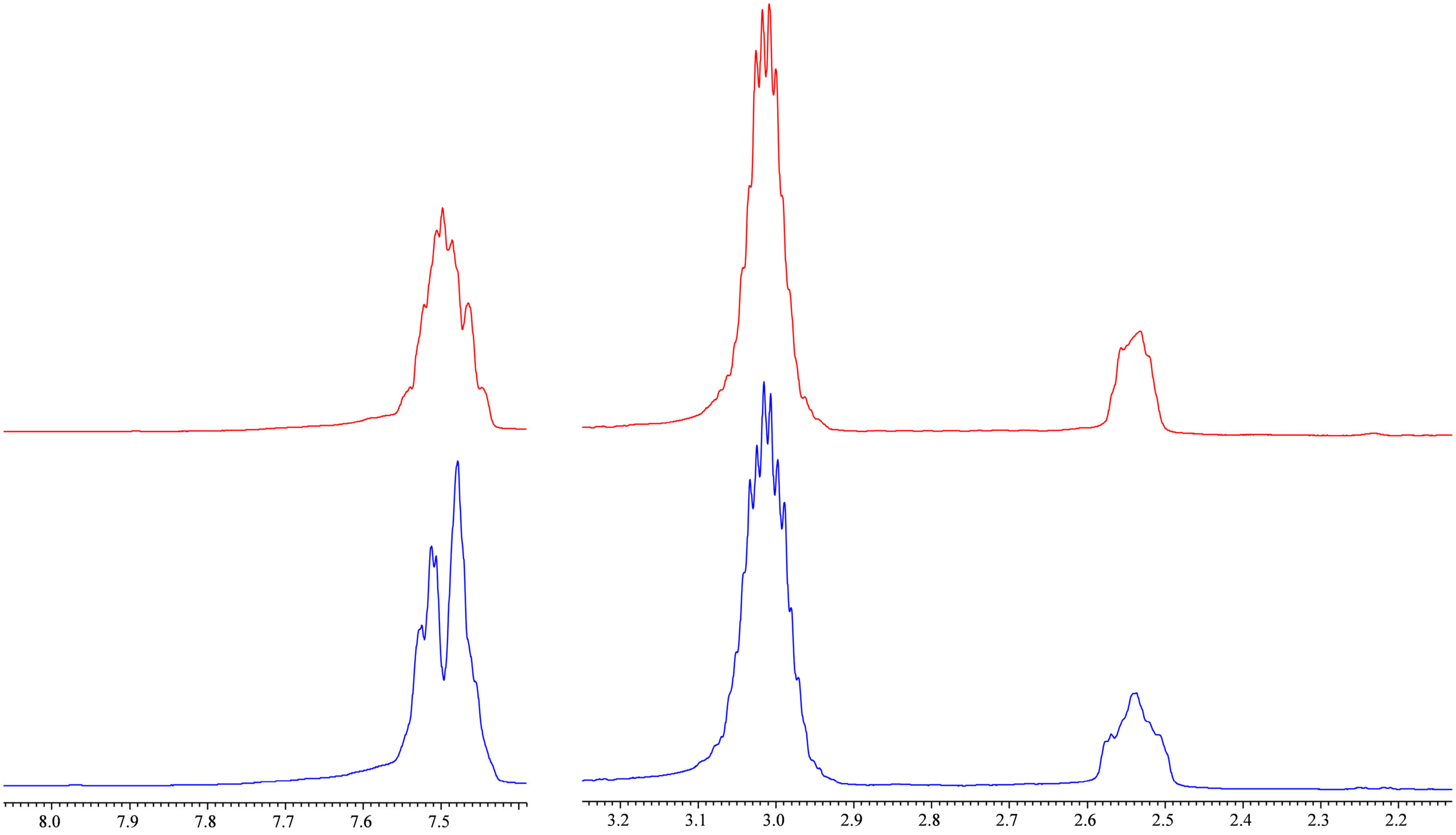

Figure 33S. ^1H and ^1H-{^{31}P} NMR spectra (400.0 MHz, $CDCl_3$) of $(Et_2N)_2(Ph)P^+–C_6H_{13}$ $I^–$ (**5a**).

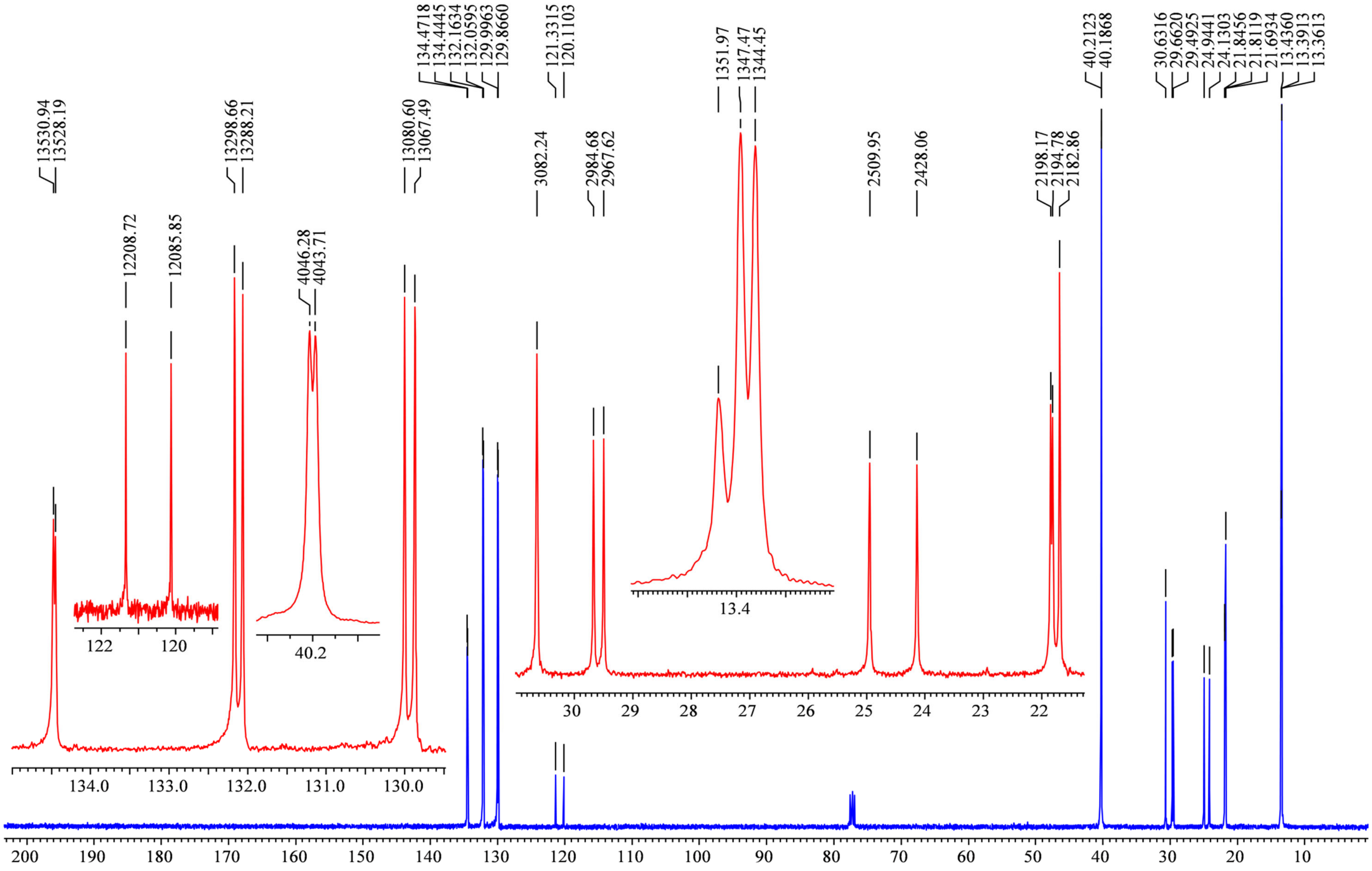

Figure 34S. ^{13}C-{^{1}H} NMR spectrum (100.6 MHz, $CDCl_3$) of $(Et_2N)_2(Ph)P^+–C_6H_{13}$ $I^–$ (**5a**).

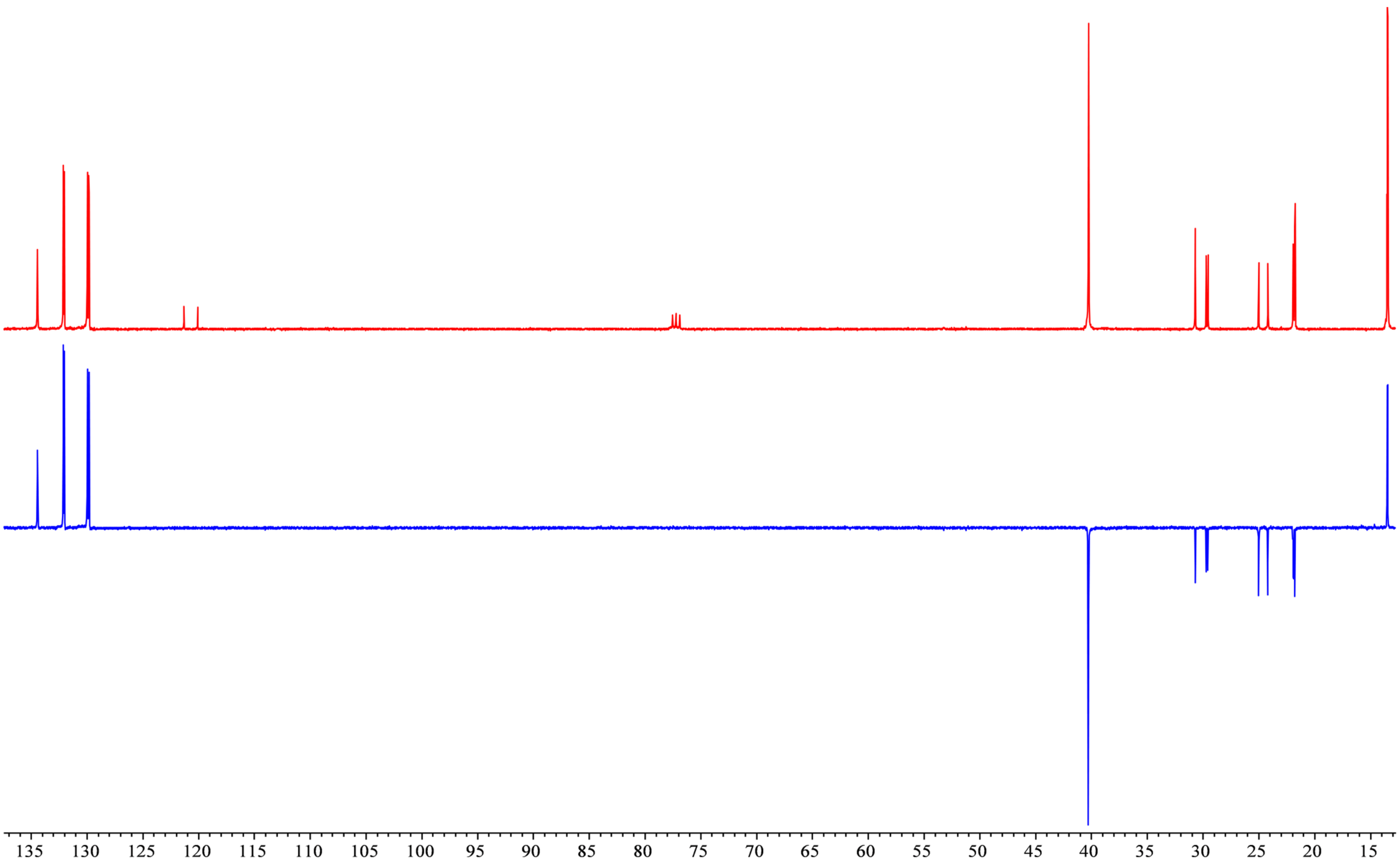

Figure 35S. ^{13}C-{^{1}H} and ^{13}C-{^{1}H}-dept NMR spectra (100.6 MHz, $CDCl_3$) of $(Et_2N)_2(Ph)P^+–C_6H_{13}\ I^−$ (**5a**).

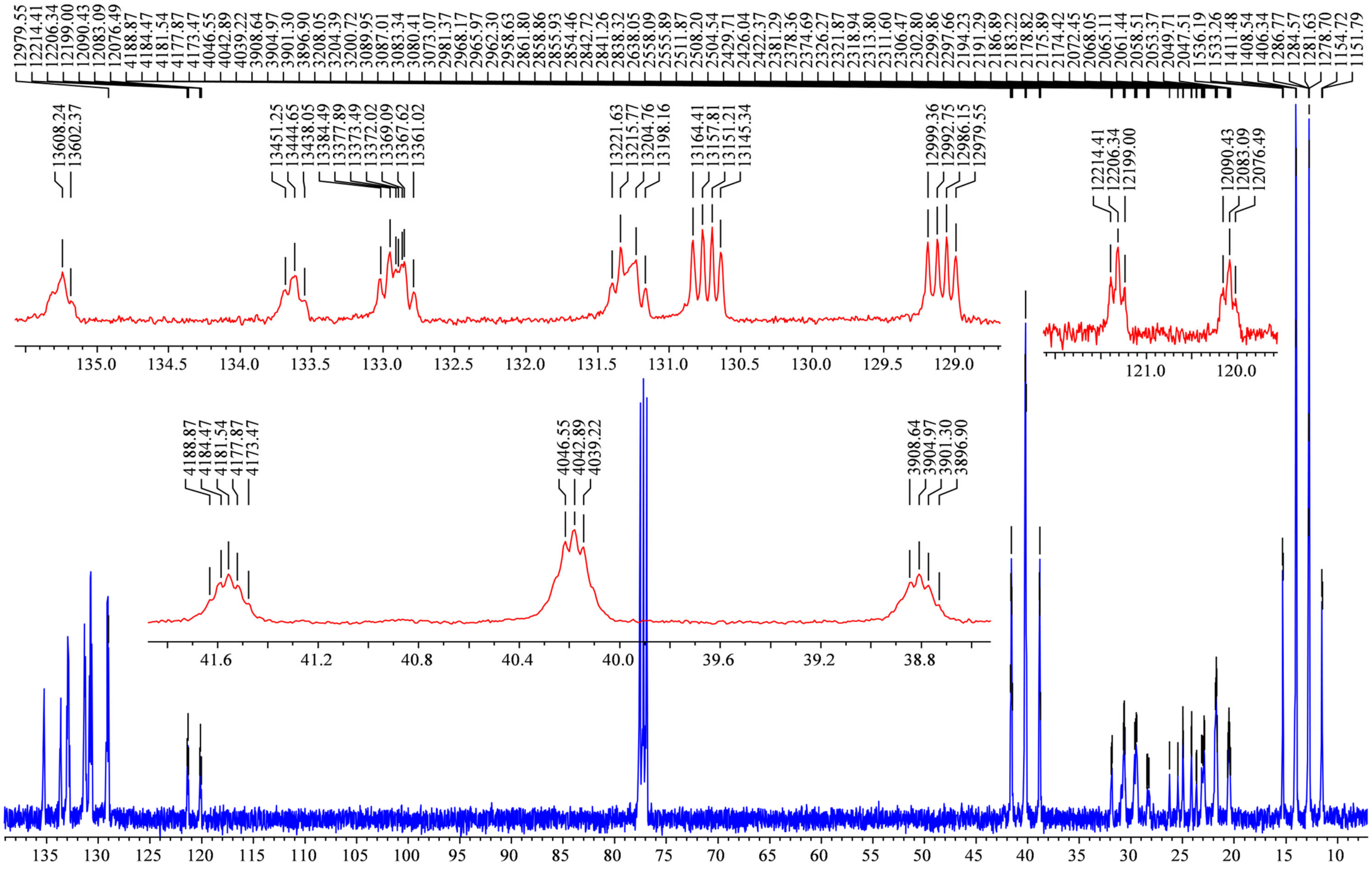

Figure 36S. ^{13}C NMR spectrum (100.6 MHz, $CDCl_3$) of $(Et_2N)_2(Ph)P^+–C_6H_{13}\ I^-$ (**5a**).

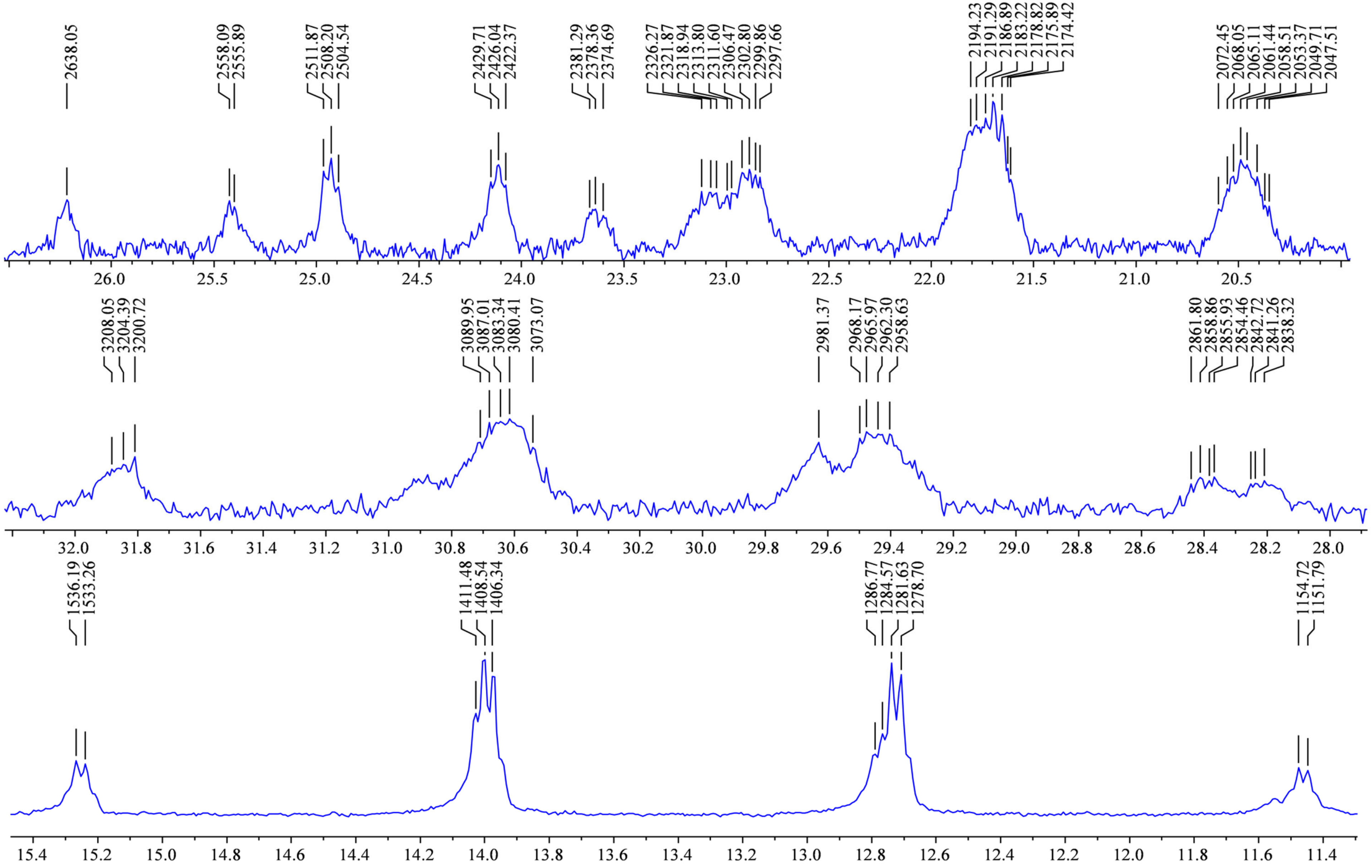

Figure 37S. High-field fragments of ^{13}C NMR spectrum (100.6 MHz, $CDCl_3$) of $(Et_2N)_2(Ph)P^+–C_6H_{13}$ $I^–$ (**5a**).

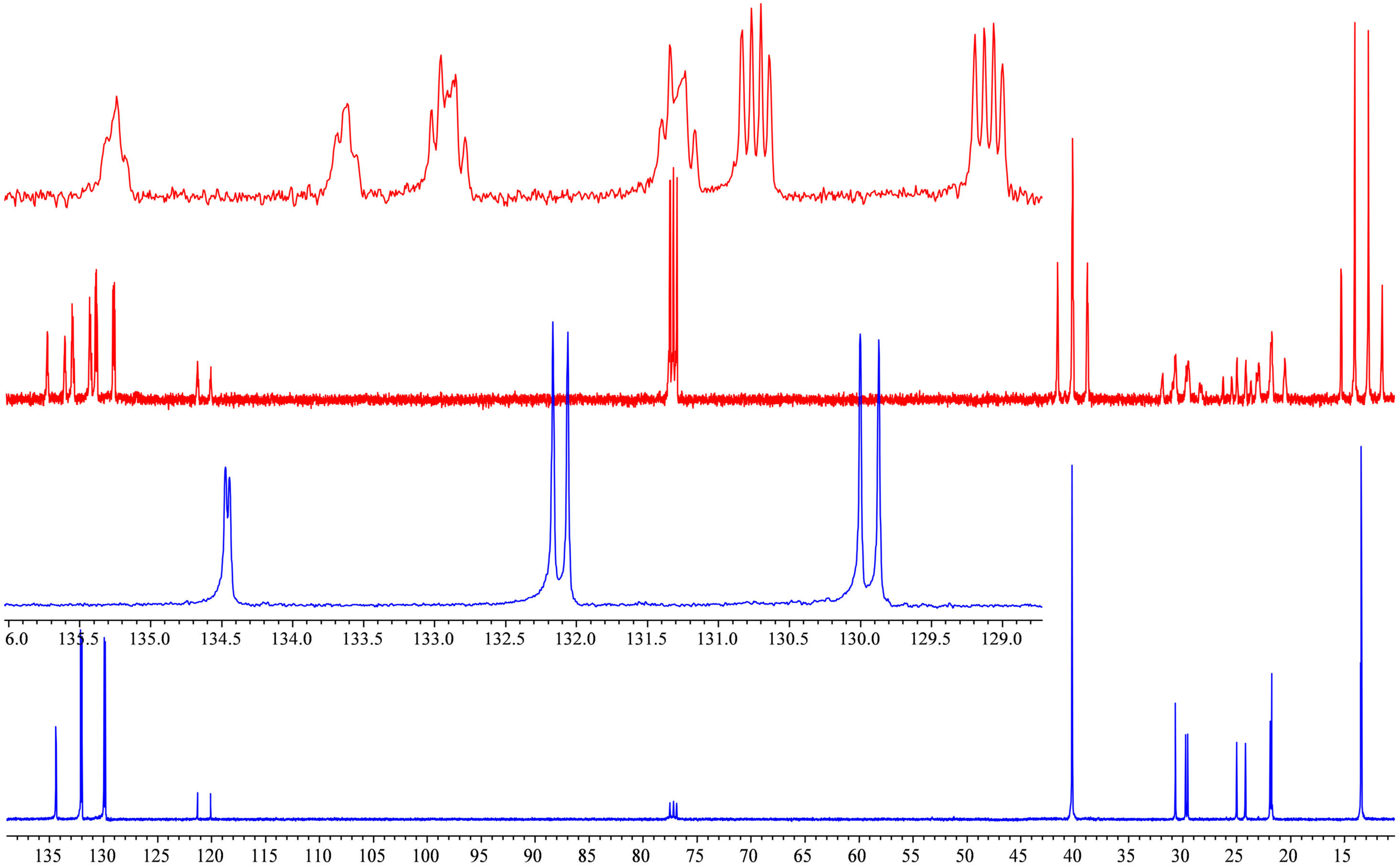

Figure 38S. ^{13}C-{^{1}H} and ^{13}C NMR spectra (100.6 MHz, $CDCl_3$) of $(Et_2N)_2(Ph)P^{+}–C_6H_{13}$ I^{-} (**5a**).

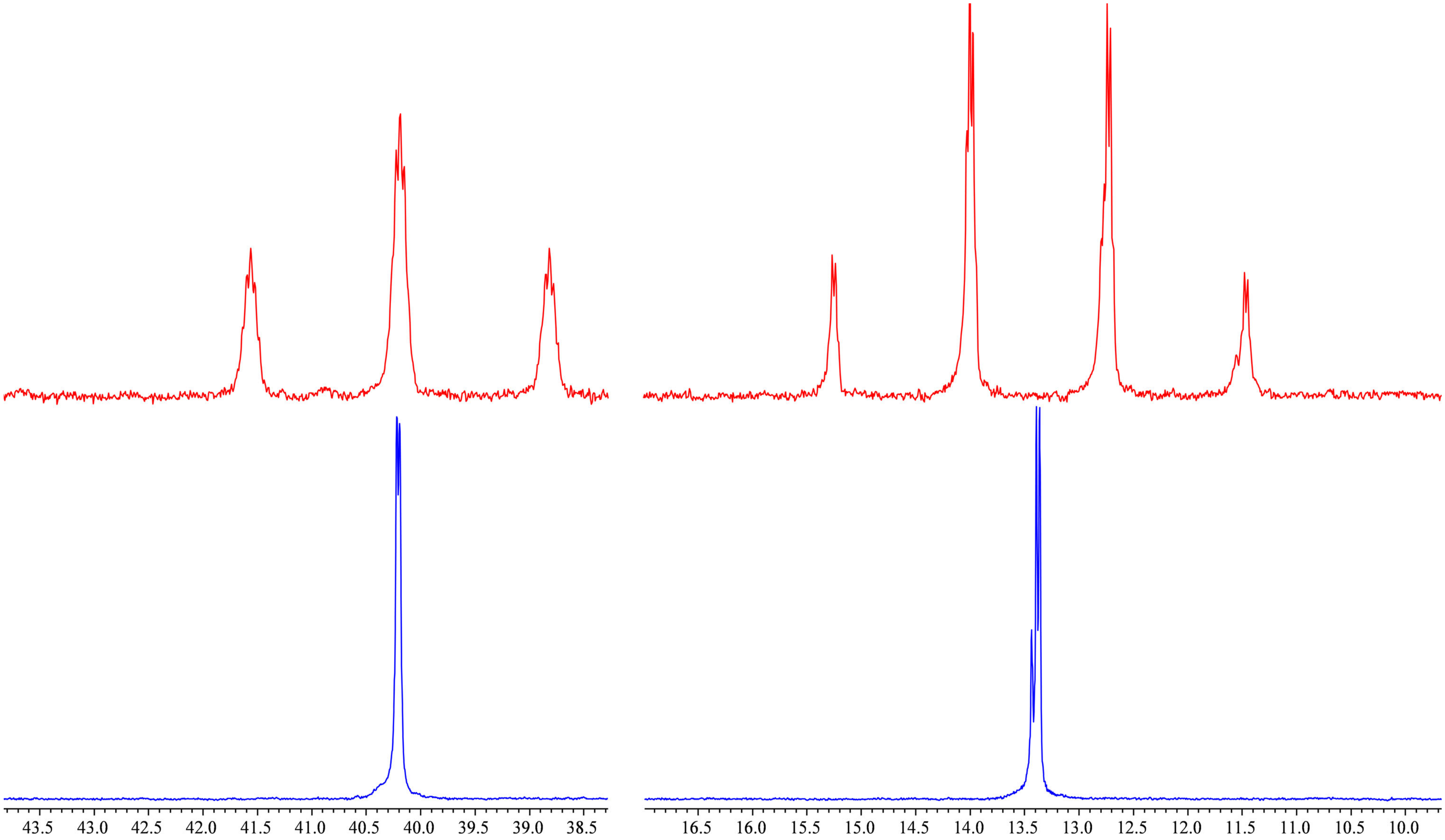

Figure 39S. High-field region of ^{13}C-{^{1}H} and ^{13}C NMR spectra (100.6 MHz, $CDCl_3$) of $(Et_2N)_2(Ph)P^+–C_6H_{13}$ $I^–$ (**5a**).

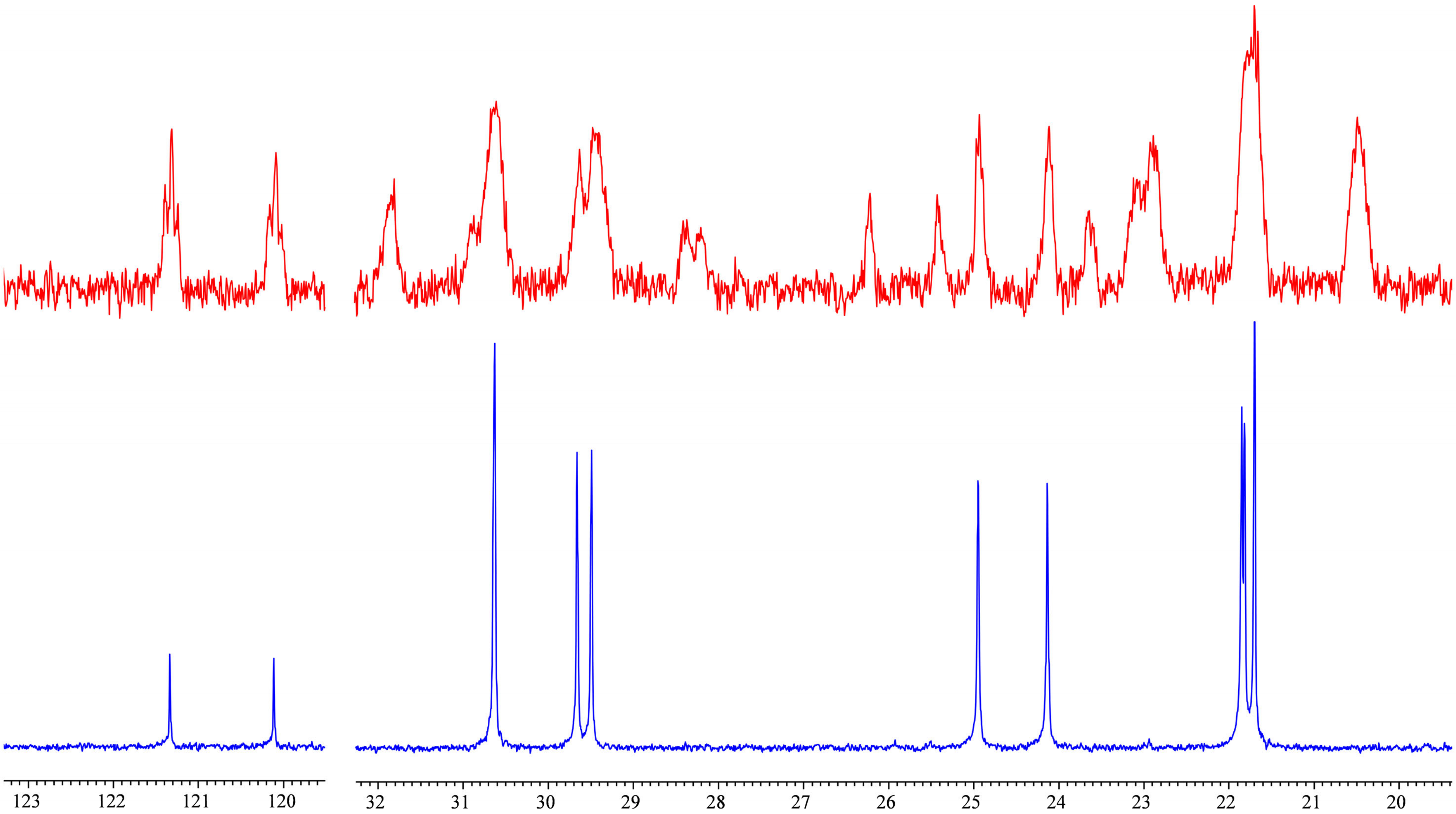

Figure 40S. The 121-123 and 20-32 ppm regions of ^{13}C-{^{1}H} and ^{13}C NMR spectra (100.6 MHz, $CDCl_3$) of $(Et_2N)_2(Ph)P^{+}–C_6H_{13}$ $I^{–}$ (**5a**).

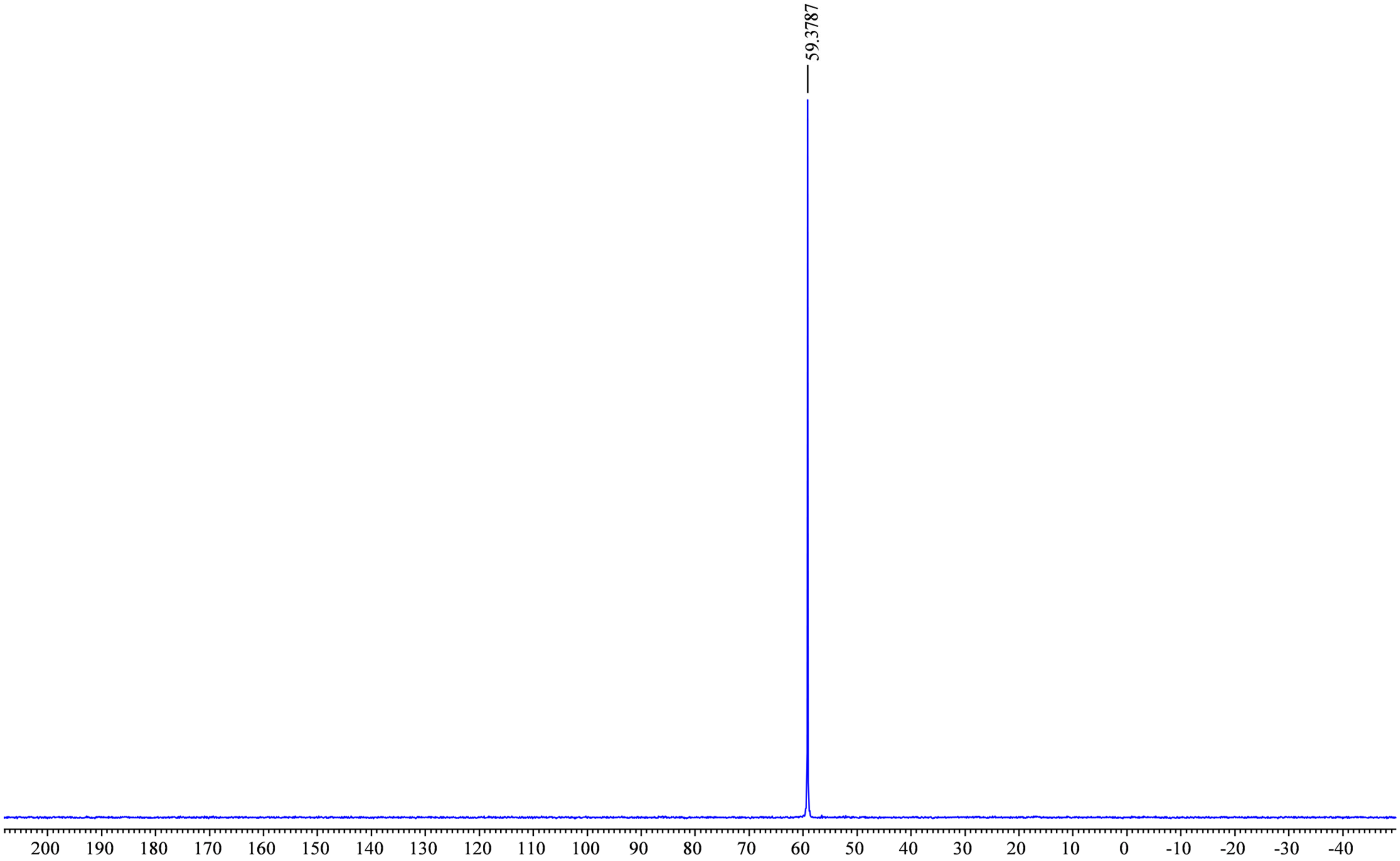

Figure 41S. ^{31}P-{^{1}H} NMR spectrum (162.0 MHz, $CDCl_3$) of $(Et_2N)_2(Ph)P^+–C_8H_{17}\ I^−$ (**5b**).

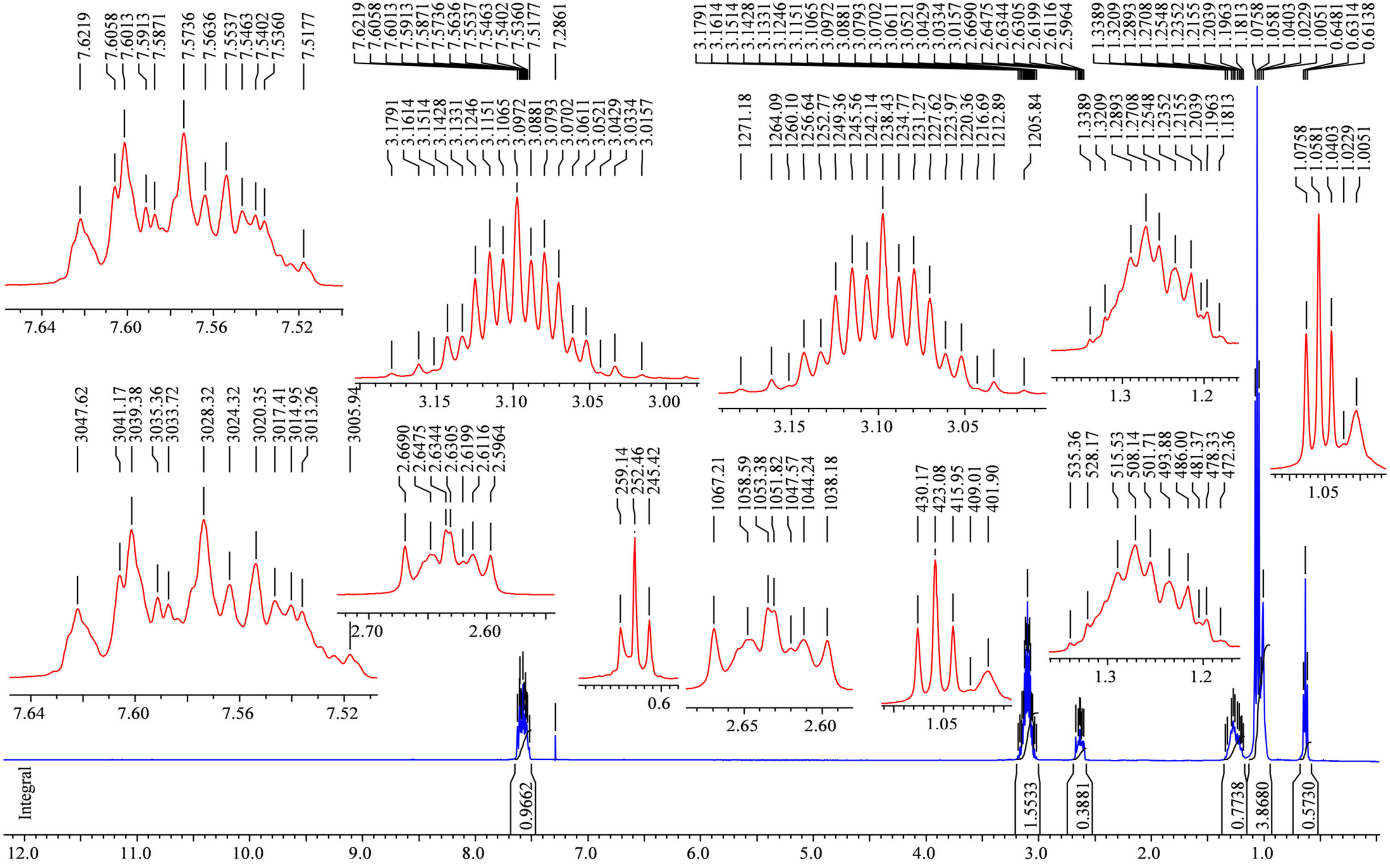

Figure 42S. ^{1}H NMR spectrum (400.0 MHz, $CDCl_3$) of $(Et_2N)_2(Ph)P^+–C_8H_{17}$ I^- (**5b**).

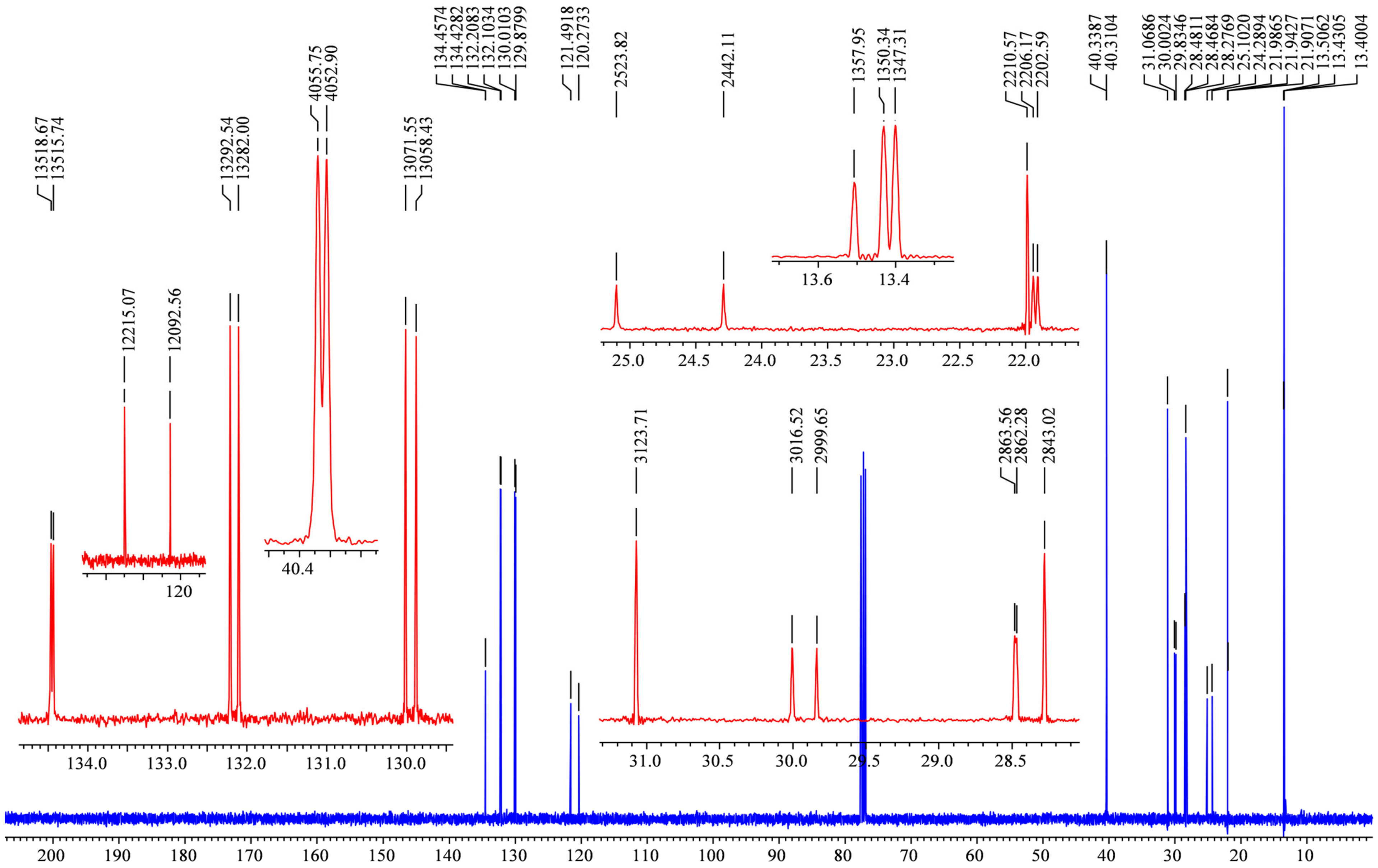

Figure 43S. $^{13}C-\{^1H\}$ NMR spectrum (100.6 MHz, $CDCl_3$) of $(Et_2N)_2(Ph)P^+–C_8H_{17}\ I^-$ (**5b**).

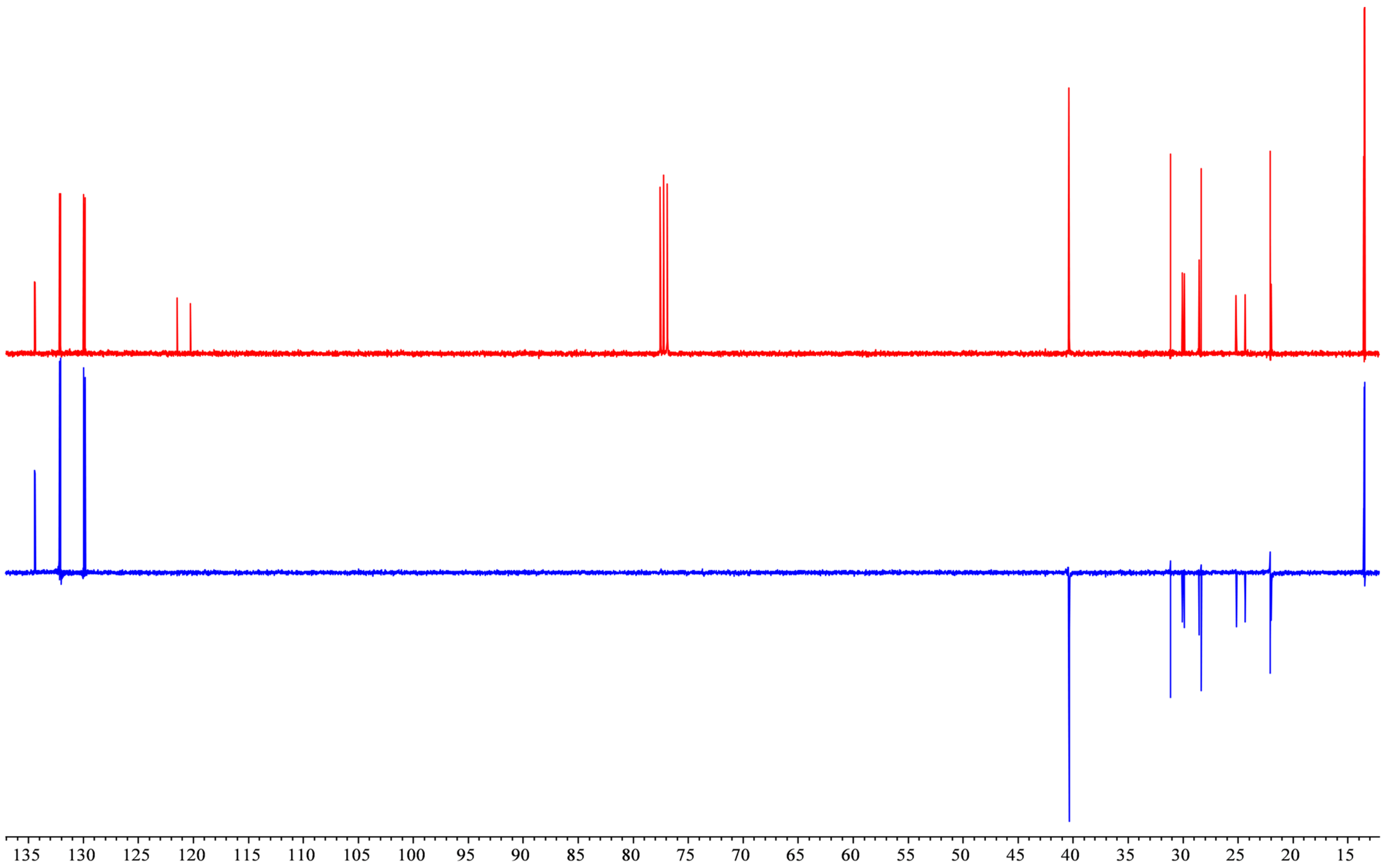

Figure 44S. ^{13}C-{^{1}H} and ^{13}C-{^{1}H}-dept NMR spectra (100.6 MHz, $CDCl_3$) of $(Et_2N)_2(Ph)P^+–C_8H_{17}\ I^-$ (**5b**).

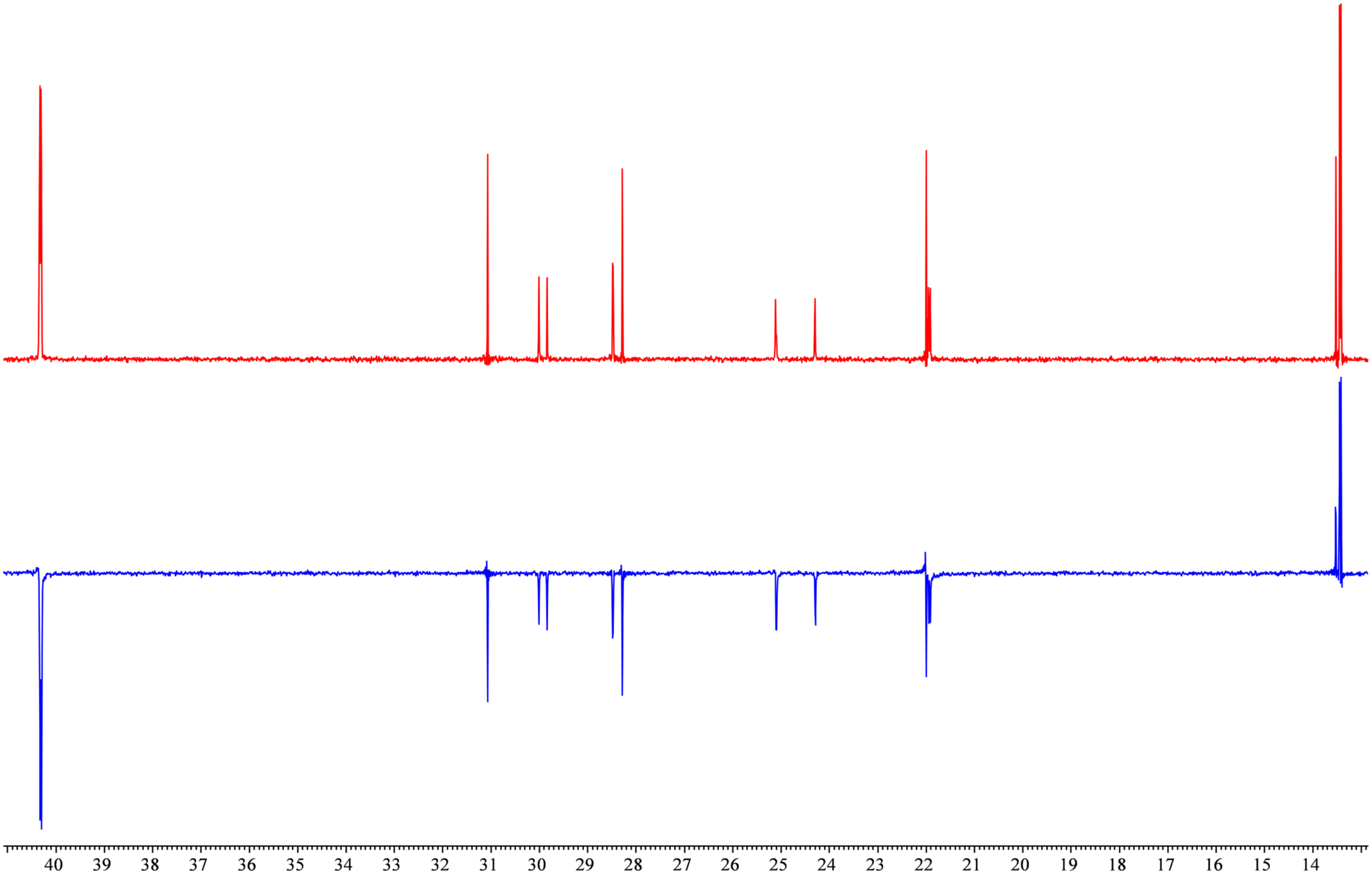

Figure 45S. High-field region of ^{13}C-{^{1}H} and ^{13}C-{^{1}H}-dept NMR spectra (100.6 MHz, $CDCl_3$) of $(Et_2N)_2(Ph)P^{+}$–C_8H_{17} I^{-} (**5b**).

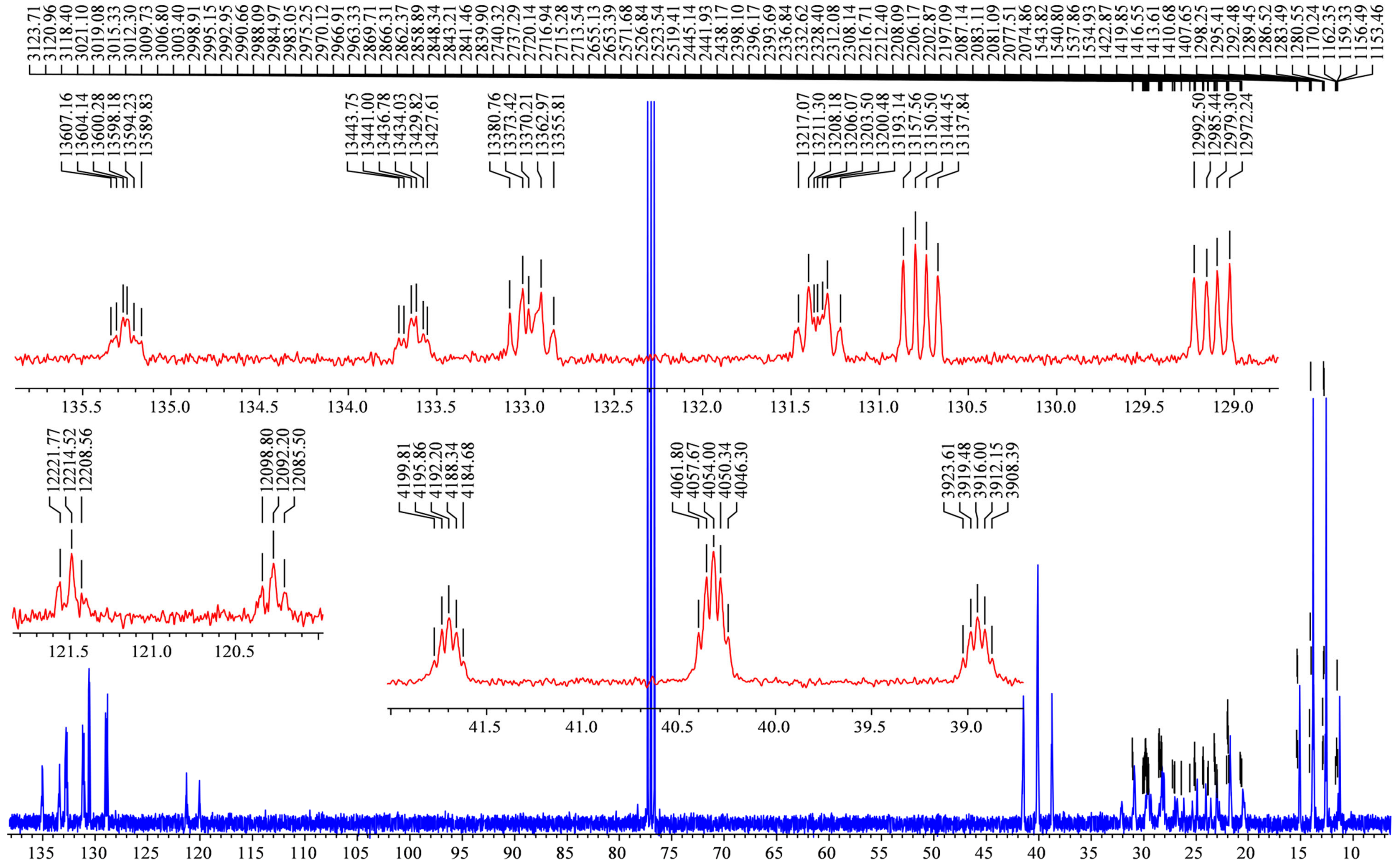

Figure 46S. ^{13}C NMR spectrum (100.6 MHz, $CDCl_3$) of $(Et_2N)_2(Ph)P^+–C_8H_{17}\ I^-$ (**5b**).

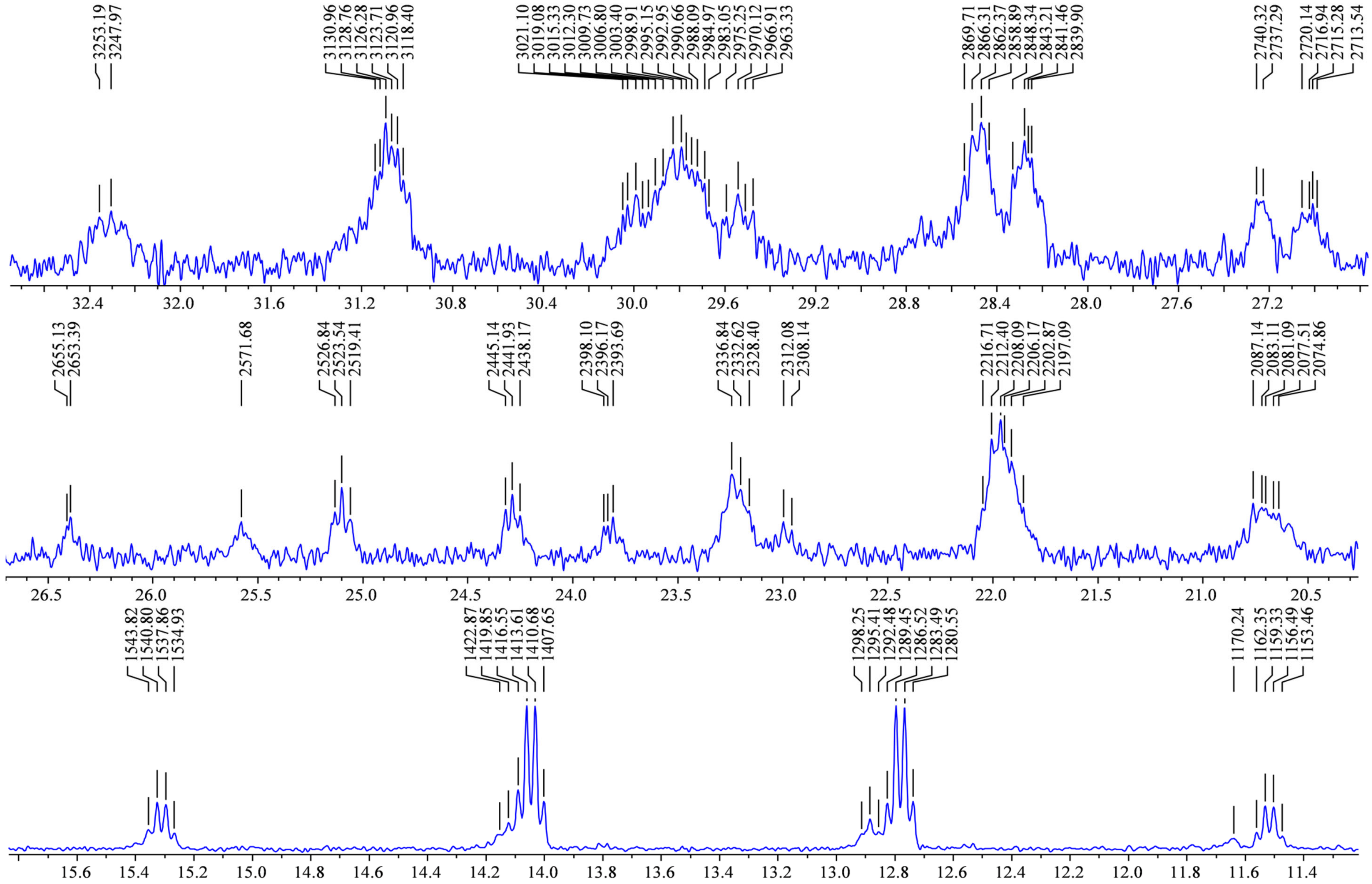

Figure 47S. High-field fragments of ^{13}C NMR spectrum (100.6 MHz, $CDCl_3$) of $(Et_2N)_2(Ph)P^+–C_8H_{17}\ I^-$ (**5b**).

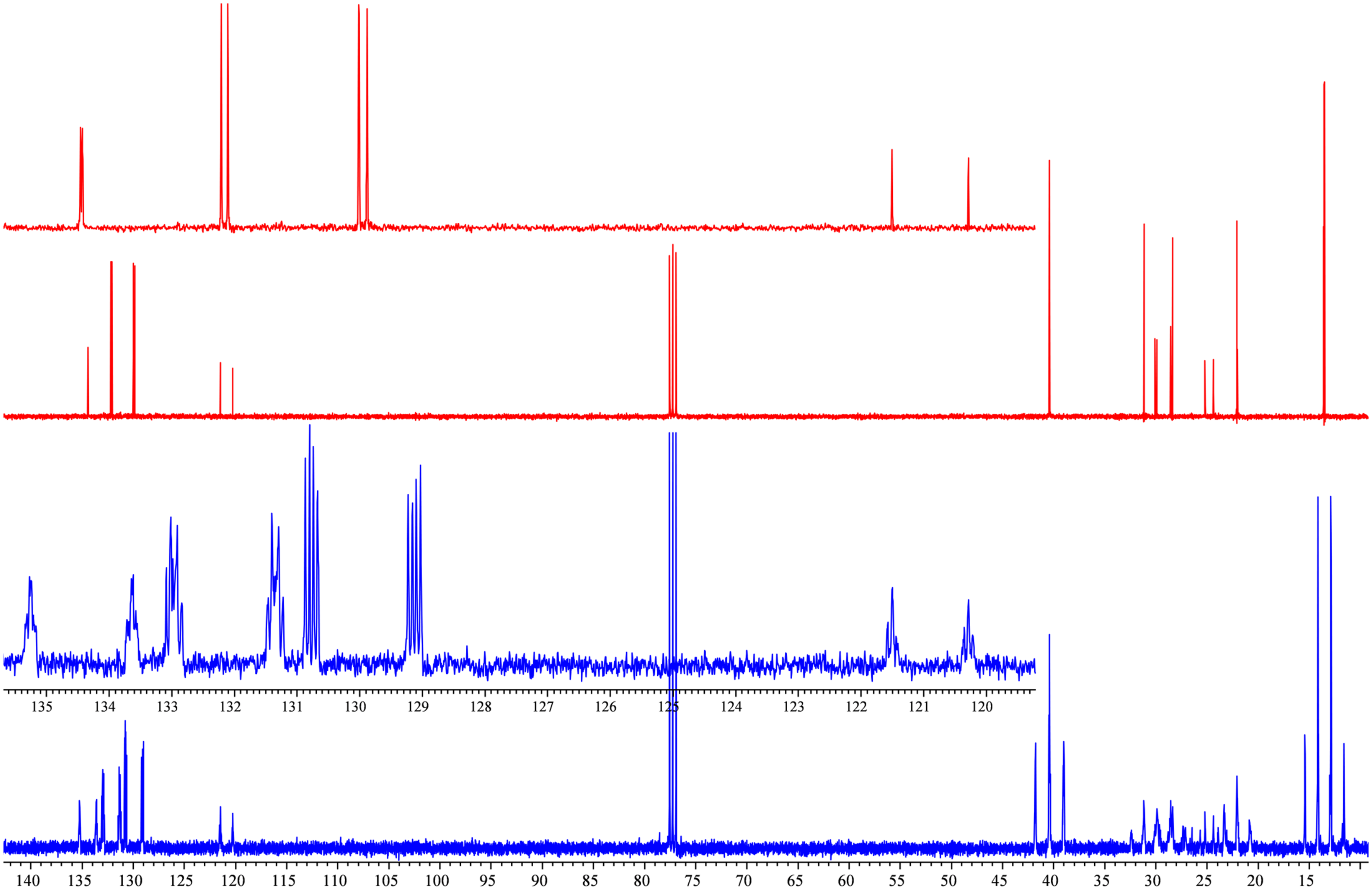

Figure 48S. ^{13}C-{^{1}H} and ^{13}C NMR spectra (100.6 MHz, $CDCl_3$) of $(Et_2N)_2(Ph)P^{+}–C_8H_{17}$ $I^{–}$ (**5b**).

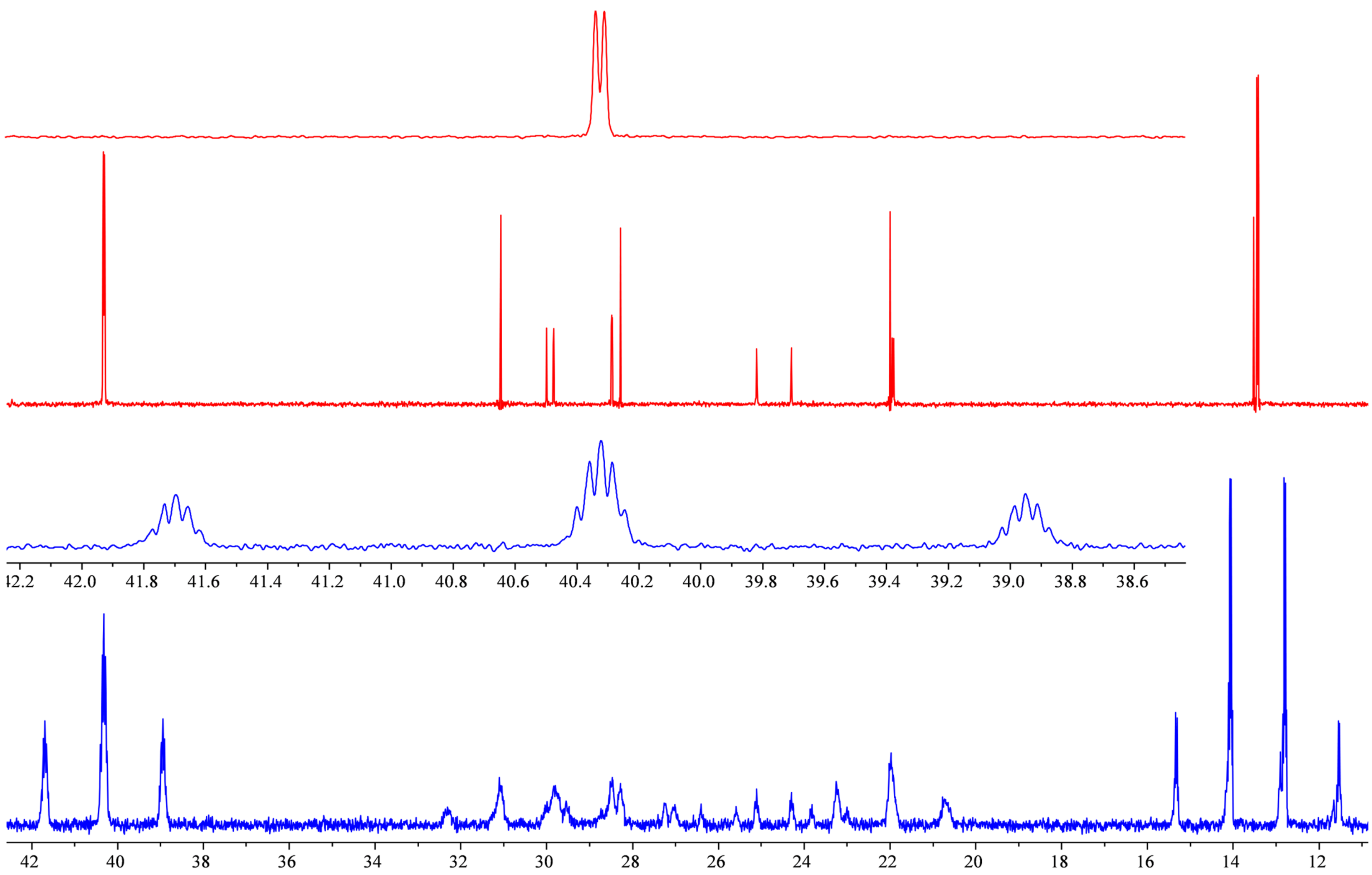

Figure 49S. High-field fragments of ^{13}C-{^{1}H} and ^{13}C NMR spectra (100.6 MHz, $CDCl_3$) of $(Et_2N)_2(Ph)P^{+}–C_8H_{17}$ $I^{–}$ (**5b**).

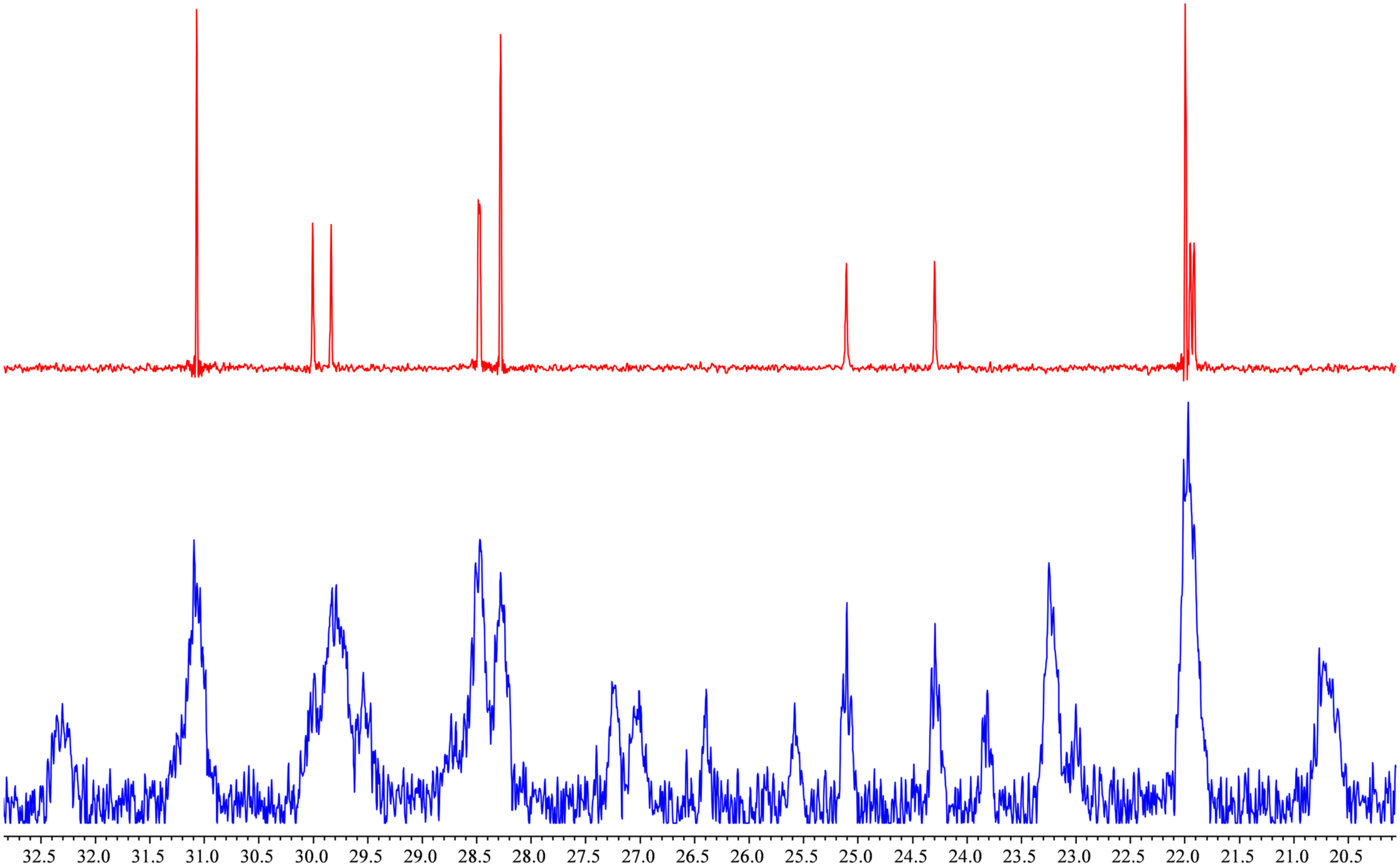

Figure 50S. The 20-32 ppm region of ^{13}C-{^{1}H} and ^{13}C NMR spectra (100.6 MHz, $CDCl_3$) of $(Et_2N)_2(Ph)P^+–C_8H_{17}\ I^–$ (**5b**).

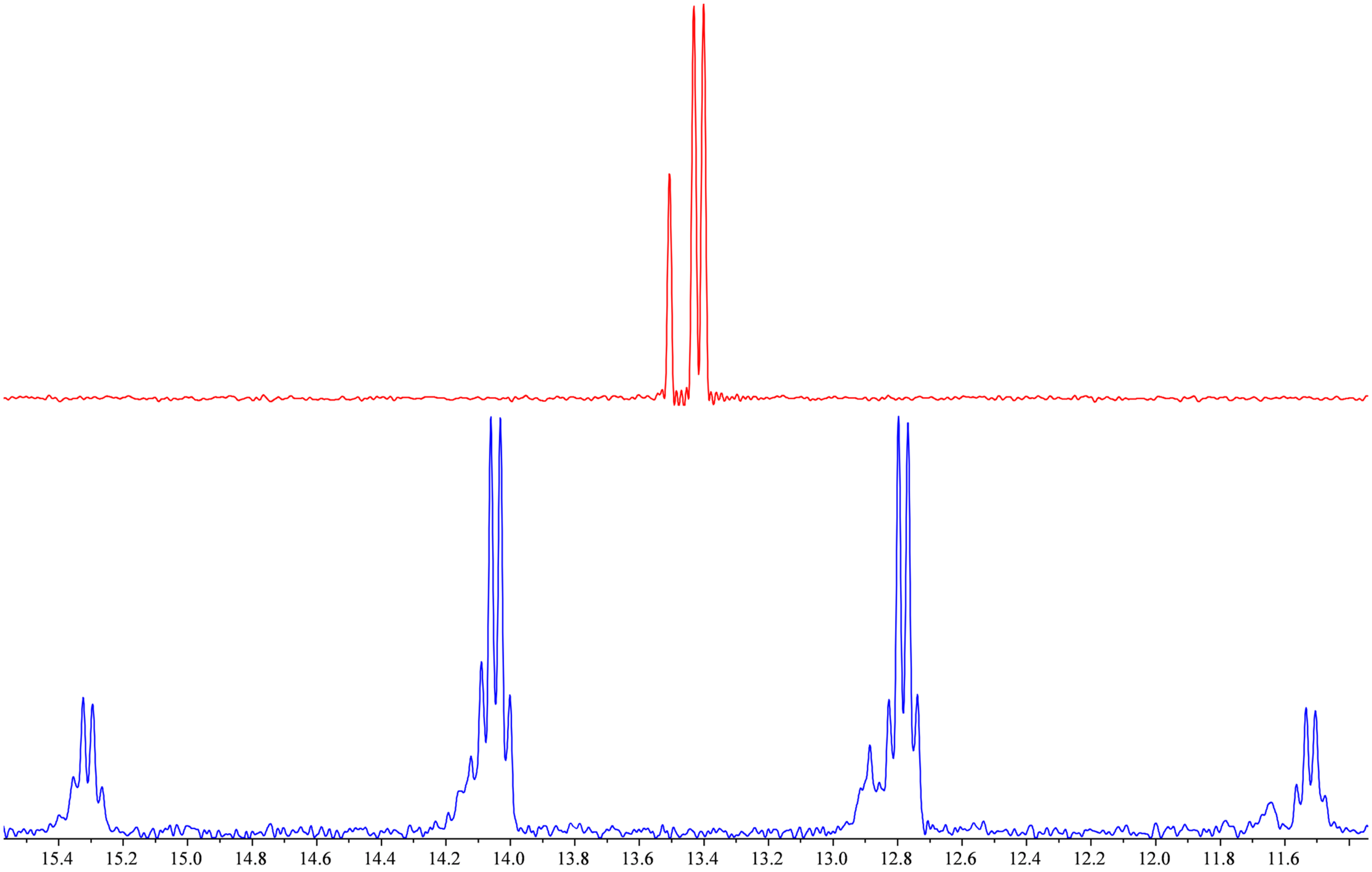

Figure 51S. The 11-16 ppm region of ^{13}C-{^1H} and ^{13}C NMR spectra (100.6 MHz, $CDCl_3$) of $(Et_2N)_2(Ph)P^+–C_8H_{17}\ I^-$ (**5b**).

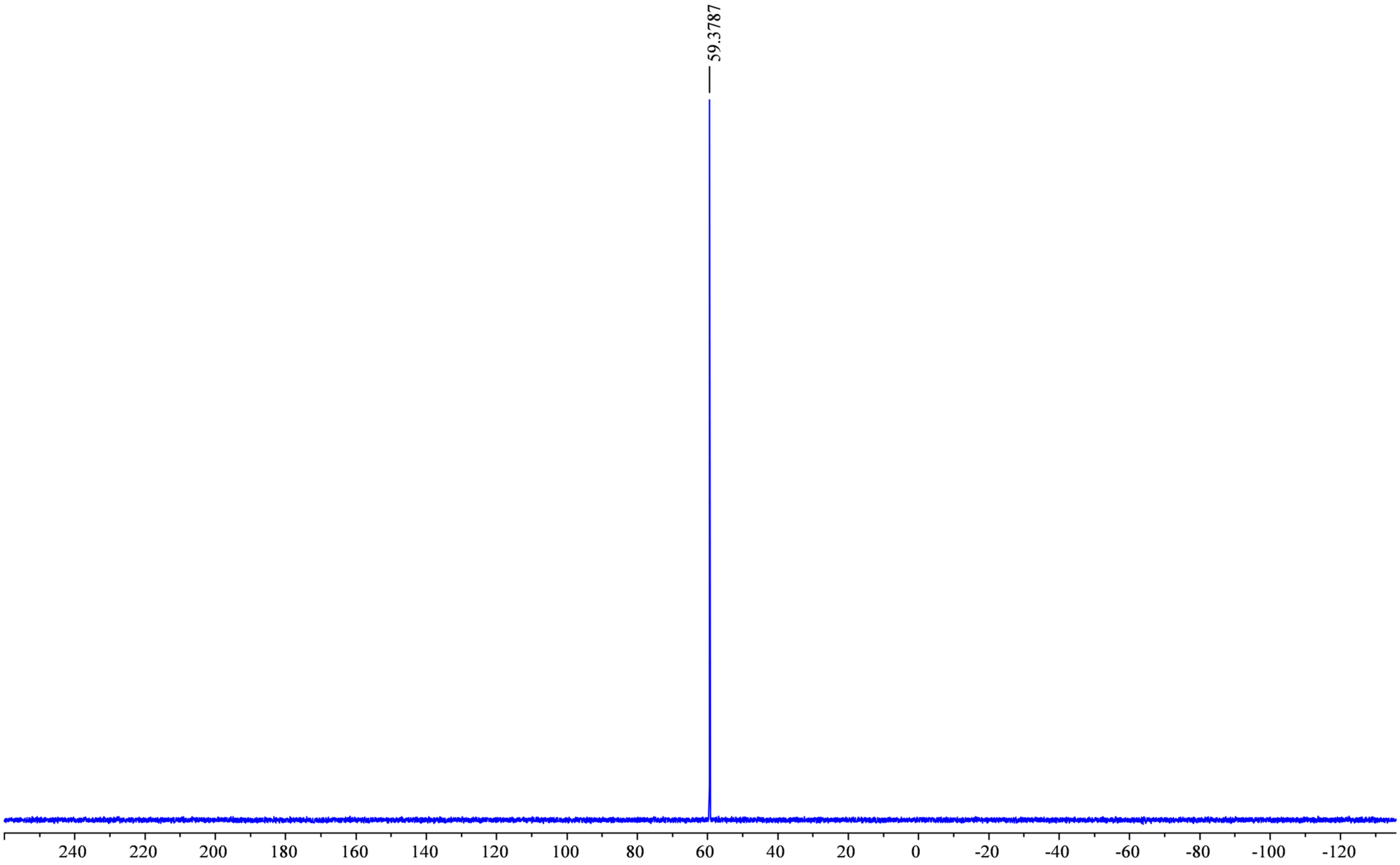

Figure 52S. ^{31}P-{^{1}H} NMR spectrum (162.0 MHz, $CDCl_3$) of $(Et_2N)_2(Ph)P^+–C_9H_{19}$ $I^−$ (**5c**).

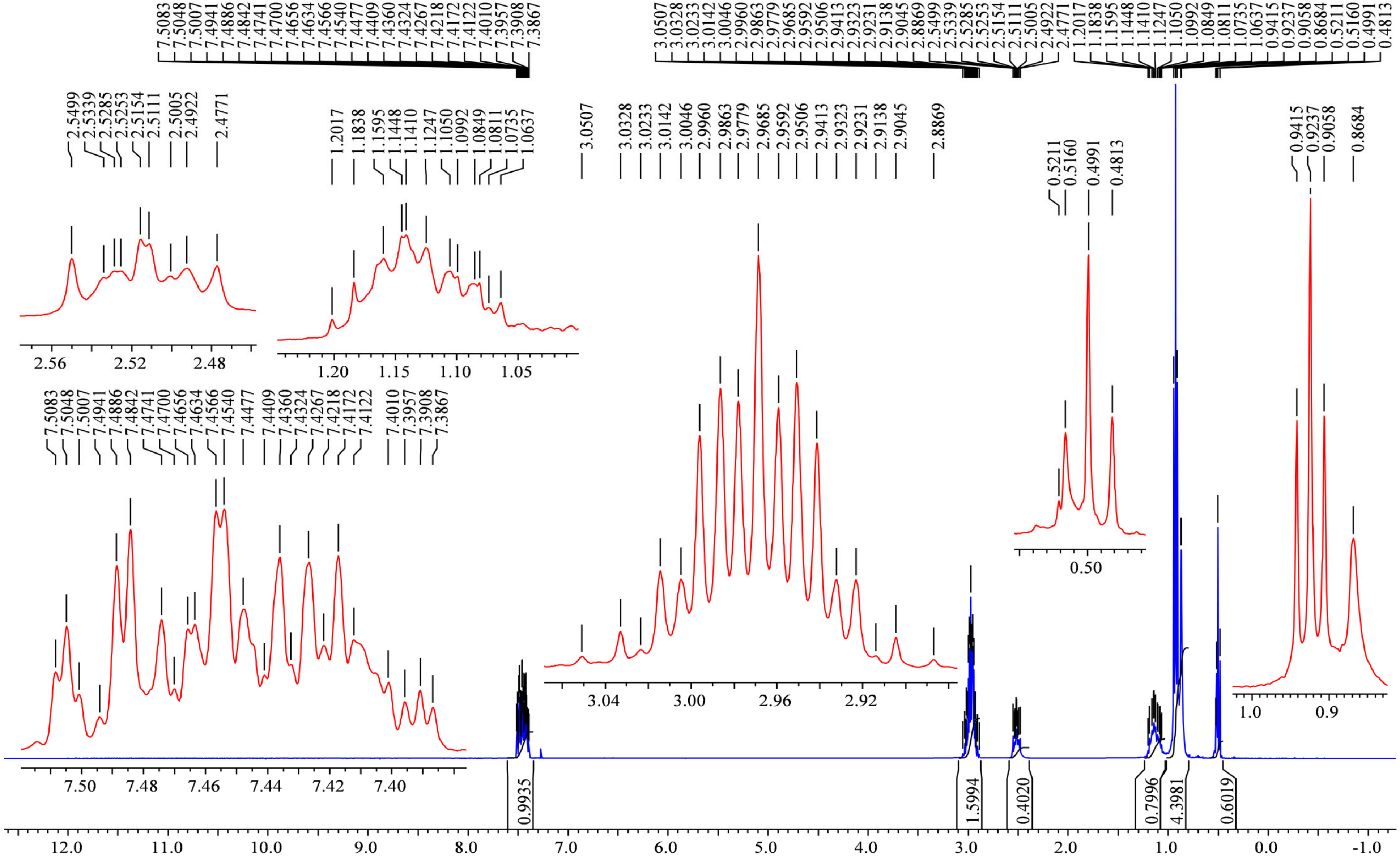

Figure 53S. ^{1}H NMR spectrum (400.0 MHz, $CDCl_3$) of $(Et_2N)_2(Ph)P^+–C_9H_{19}\ I^-$ (**5c**).

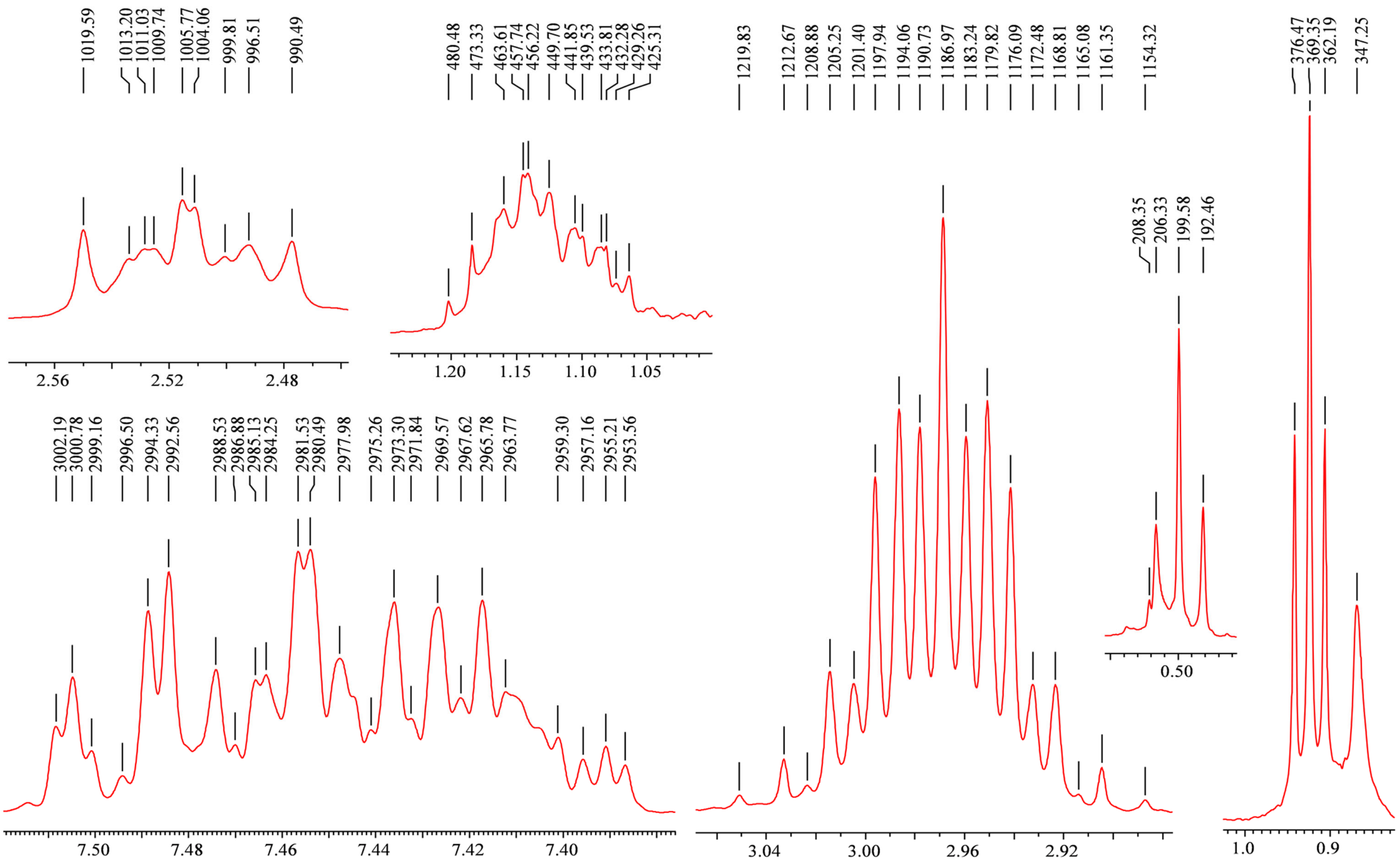

Figure 54S. Fragments of ^{1}H NMR spectrum (400.0 MHz, $CDCl_3$) of $(Et_2N)_2(Ph)P^{+}–C_9H_{19}$ $I^{–}$ (**5c**).

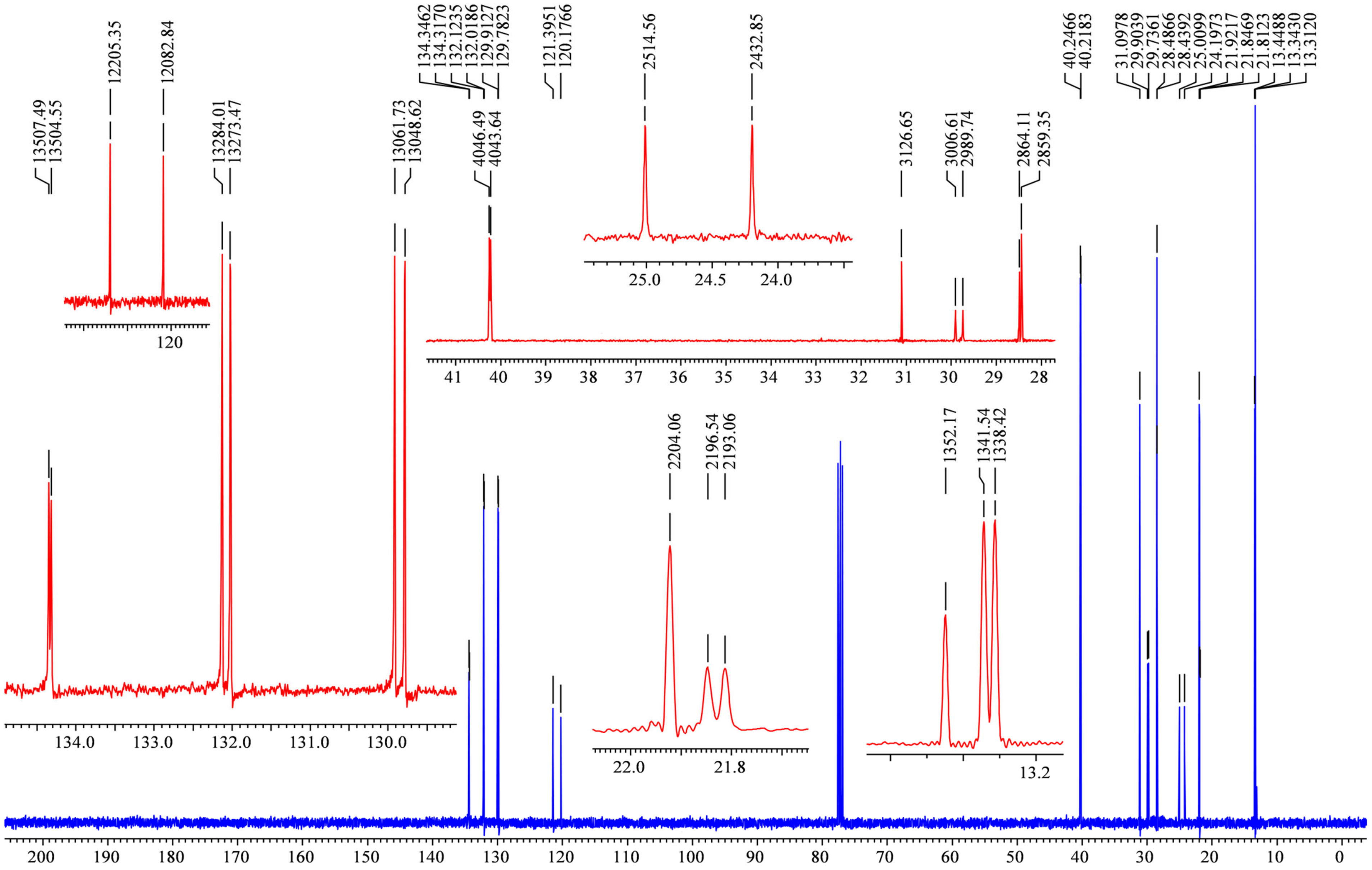

Figure 55S. $^{13}C-\{^1H\}$ NMR spectrum (100.6 MHz, $CDCl_3$) of $(Et_2N)_2(Ph)P^+–C_9H_{19}\ I^-$ (**5c**).

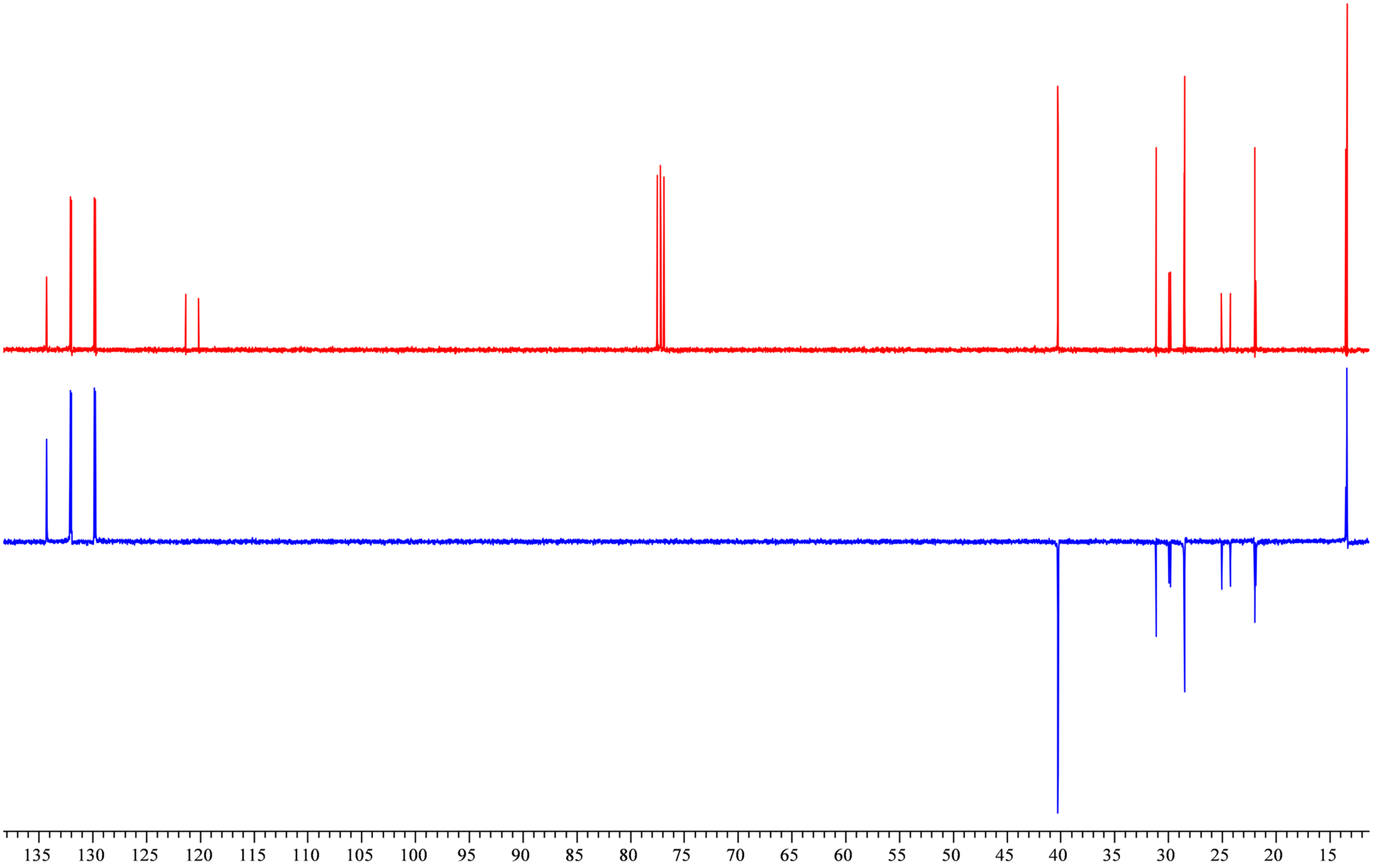

Figure 56S. ^{13}C-{^{1}H} and ^{13}C-{^{1}H}-dept NMR spectra (100.6 MHz, $CDCl_3$) of $(Et_2N)_2(Ph)P^+–C_9H_{19}\ I^-$ (**5c**).

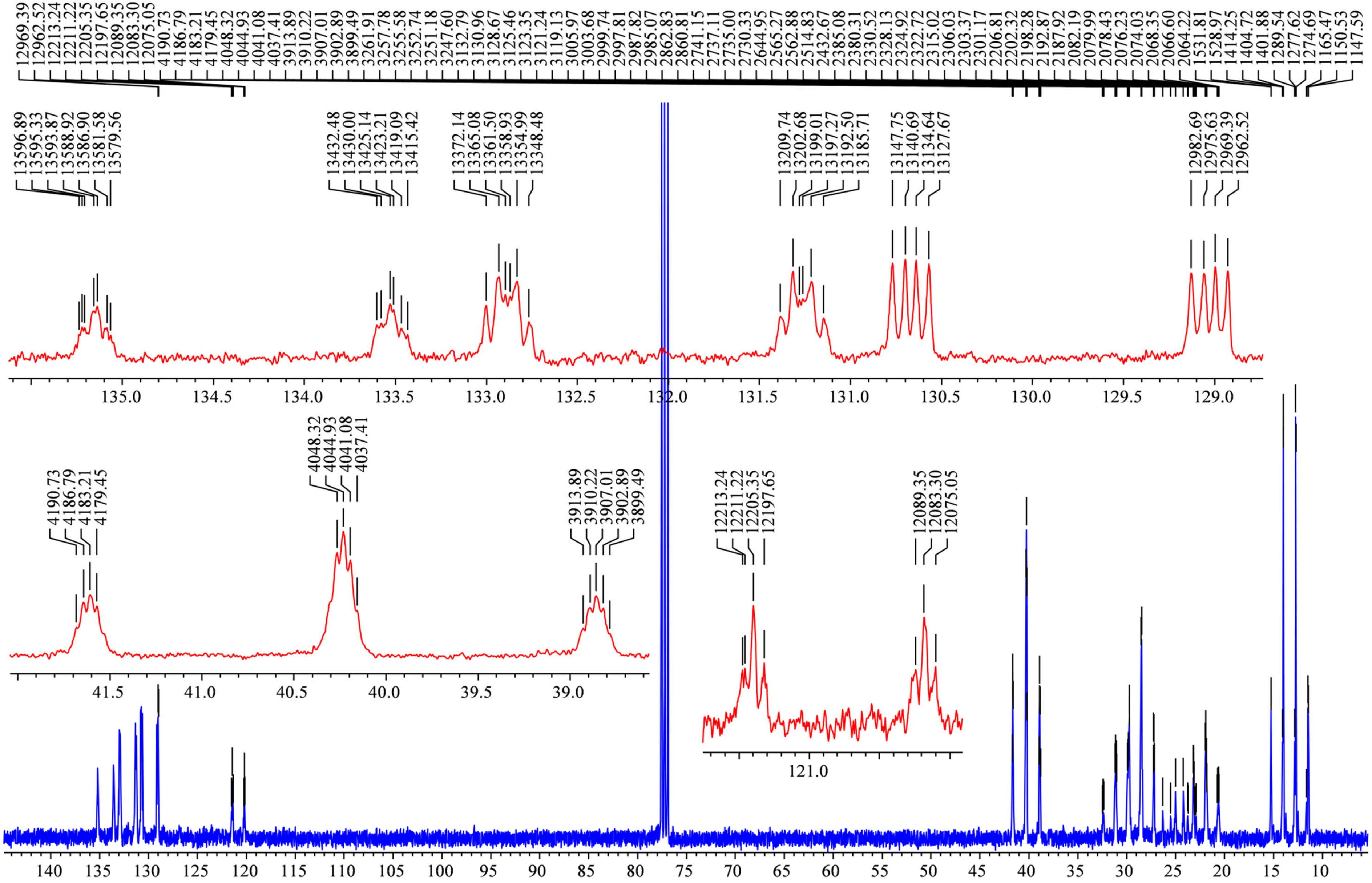

Figure 57S. ^{13}C NMR spectrum (100.6 MHz, $CDCl_3$) of $(Et_2N)_2(Ph)P^+–C_9H_{19}\ I^-$ (**5c**).

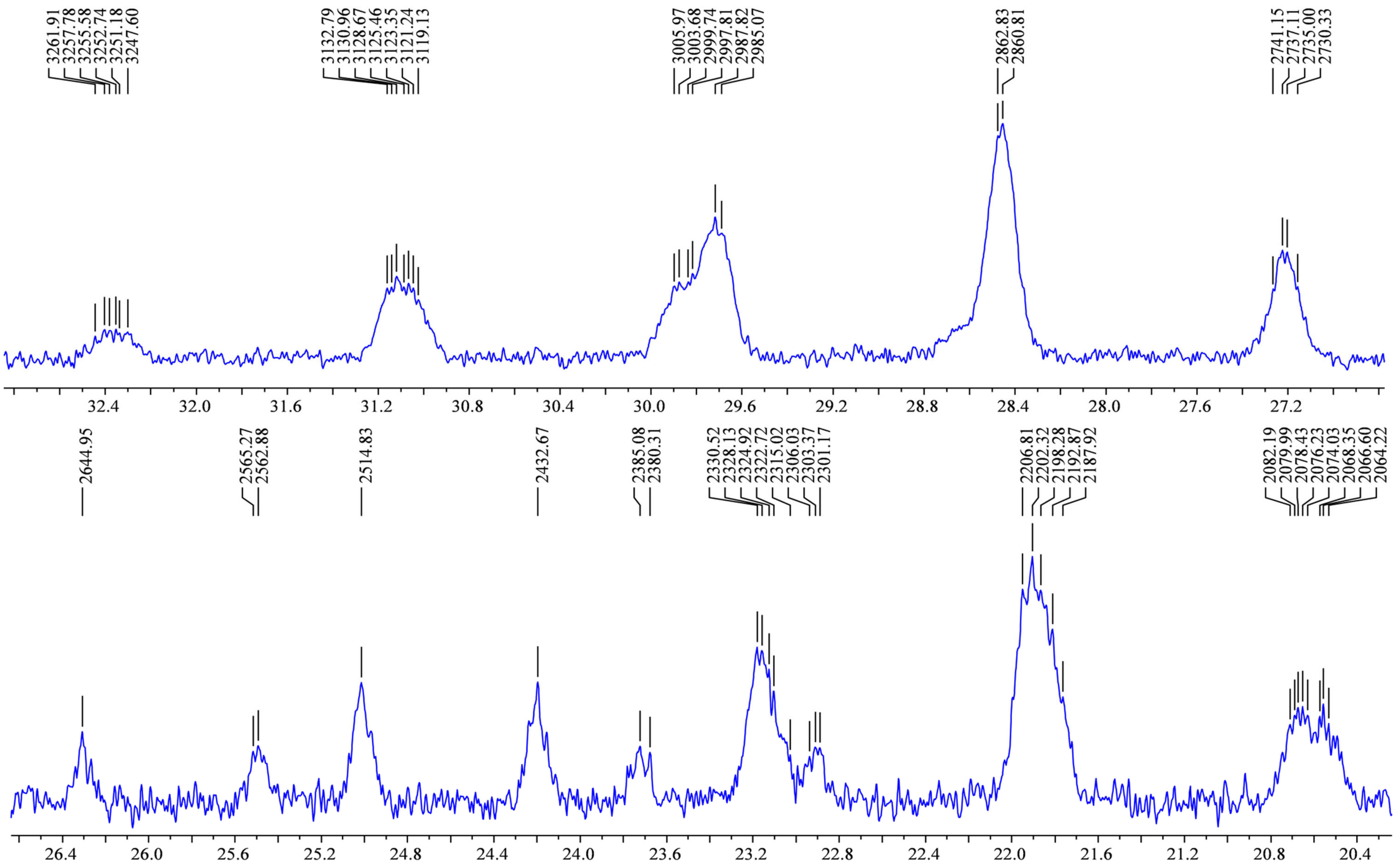

Figure 58S. The 20-27 and 27-32 ppm regions of ^{13}C NMR spectrum (100.6 MHz, $CDCl_3$) of $(Et_2N)_2(Ph)P^+–C_9H_{19}\ I^-$ (**5c**).

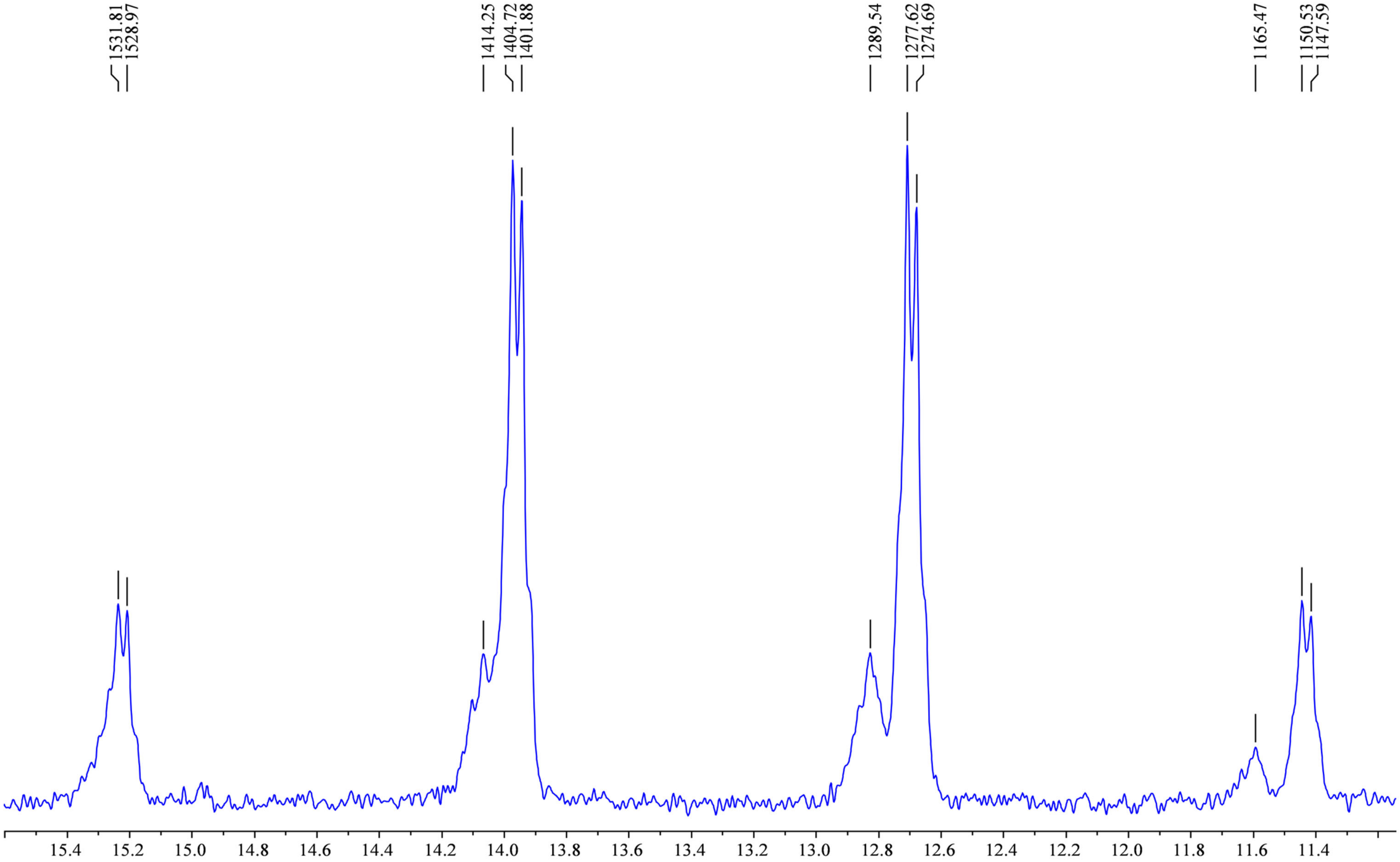

Figure 59S. High-field fragment of ^{13}C NMR spectrum (100.6 MHz, $CDCl_3$) of $(Et_2N)_2(Ph)P^+–C_9H_{19}$ $I^–$ (**5c**).

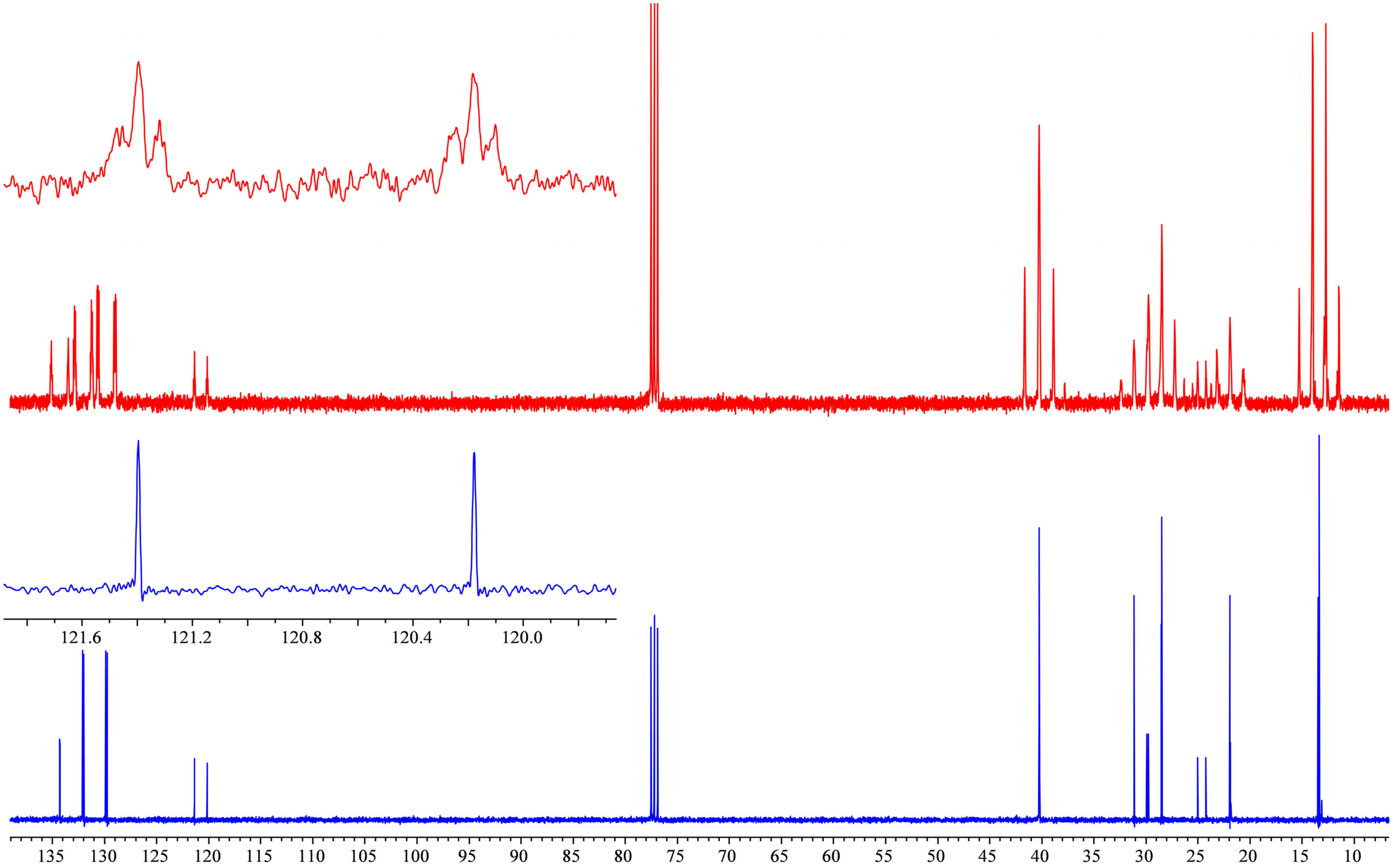

Figure 60S. ^{13}C and ^{13}C-{^{1}H} NMR spectra (100.6 MHz, $CDCl_3$) of $(Et_2N)_2(Ph)P^+–C_9H_{19}$ $I^–$ (**5c**).

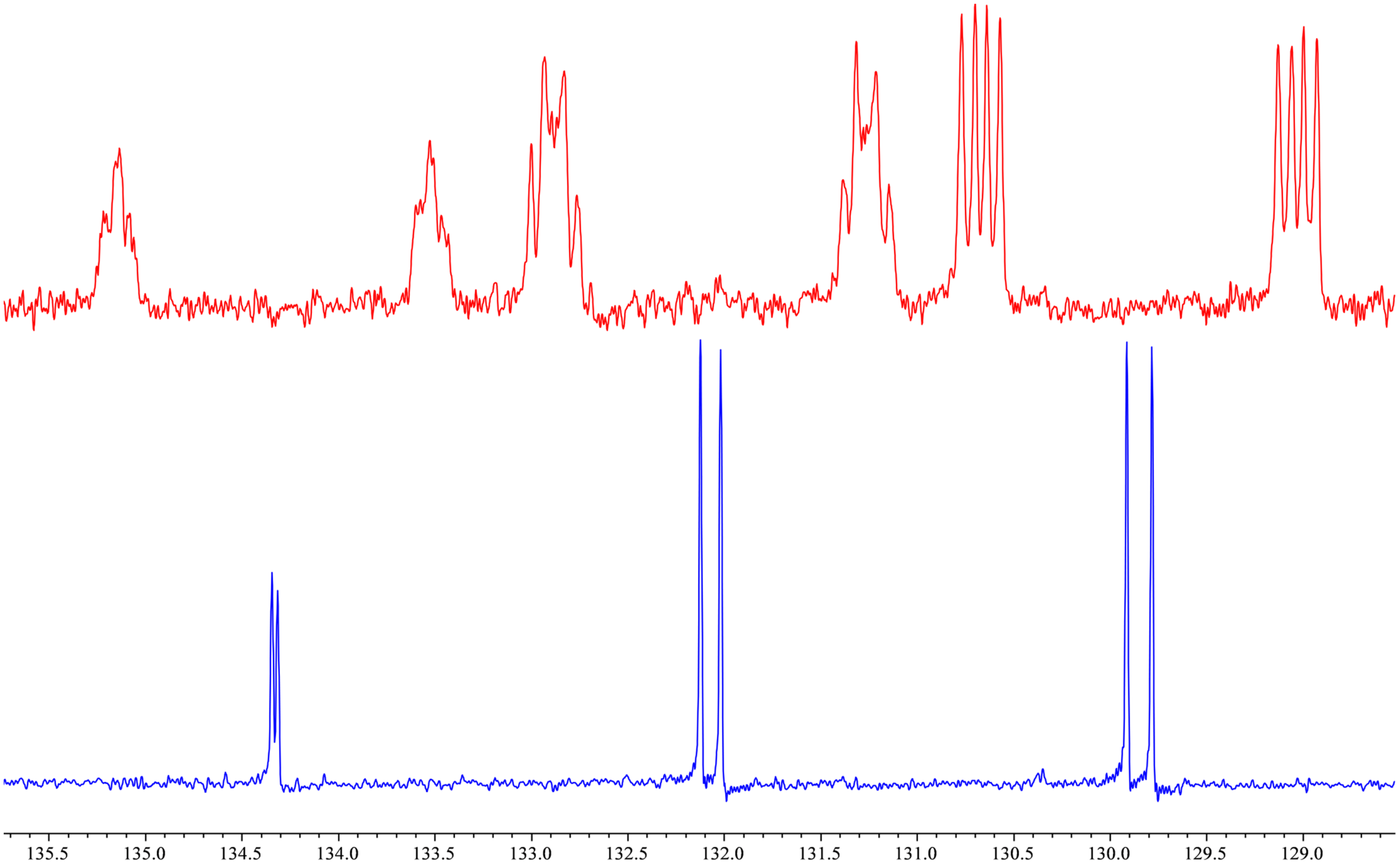

Figure 61S. Low-field fragment of ^{13}C and $^{13}C-\{^{1}H\}$ NMR spectra (100.6 MHz, $CDCl_3$) of $(Et_2N)_2(Ph)P^{+}–C_9H_{19}$ $I^{–}$ (**5c**).

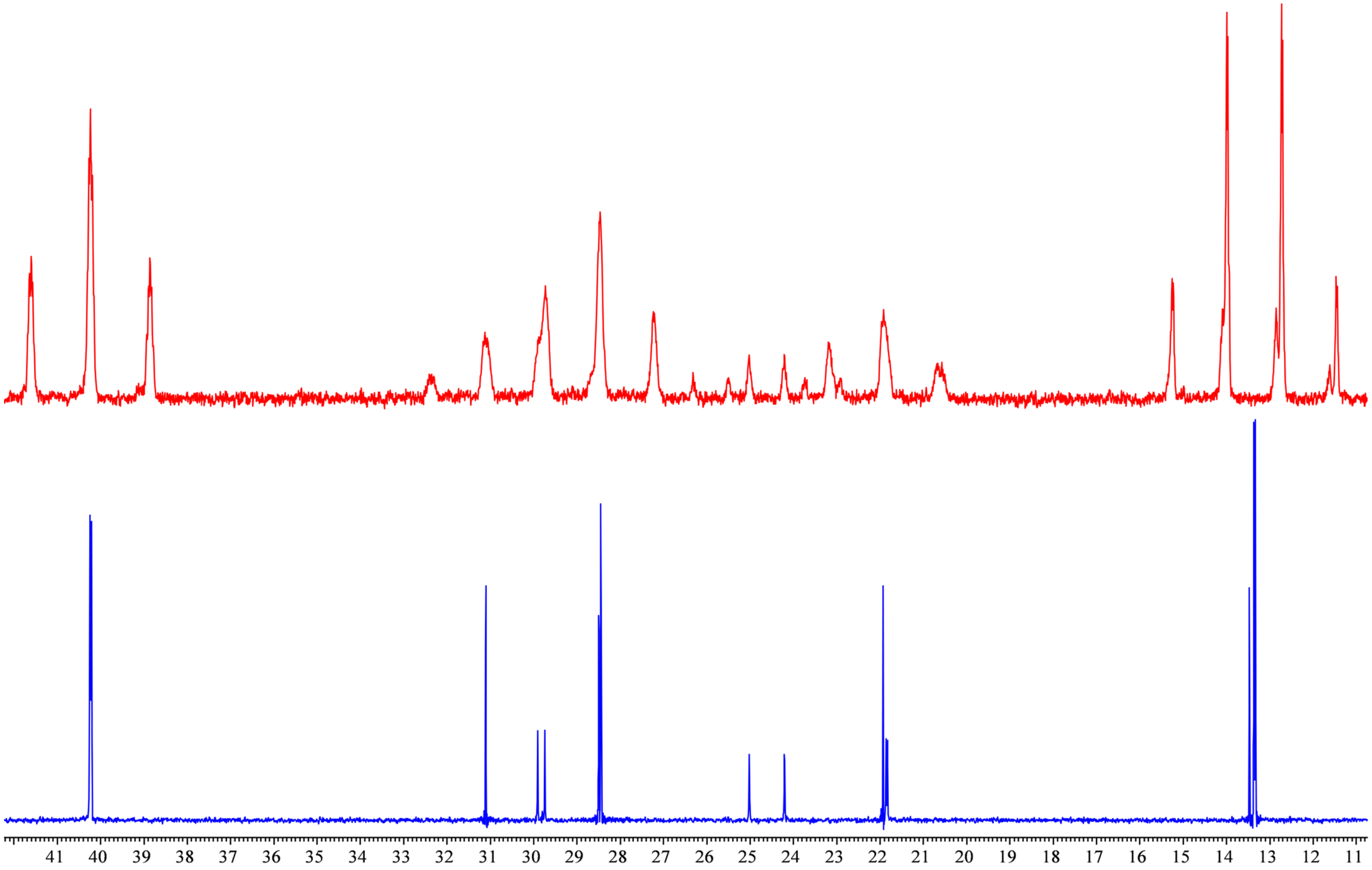

Figure 62S. High-field fragment of ^{13}C and ^{13}C-{^{1}H} NMR spectra (100.6 MHz, $CDCl_3$) of $(Et_2N)_2(Ph)P^{+}–C_9H_{19}$ $I^{−}$ (**5c**).

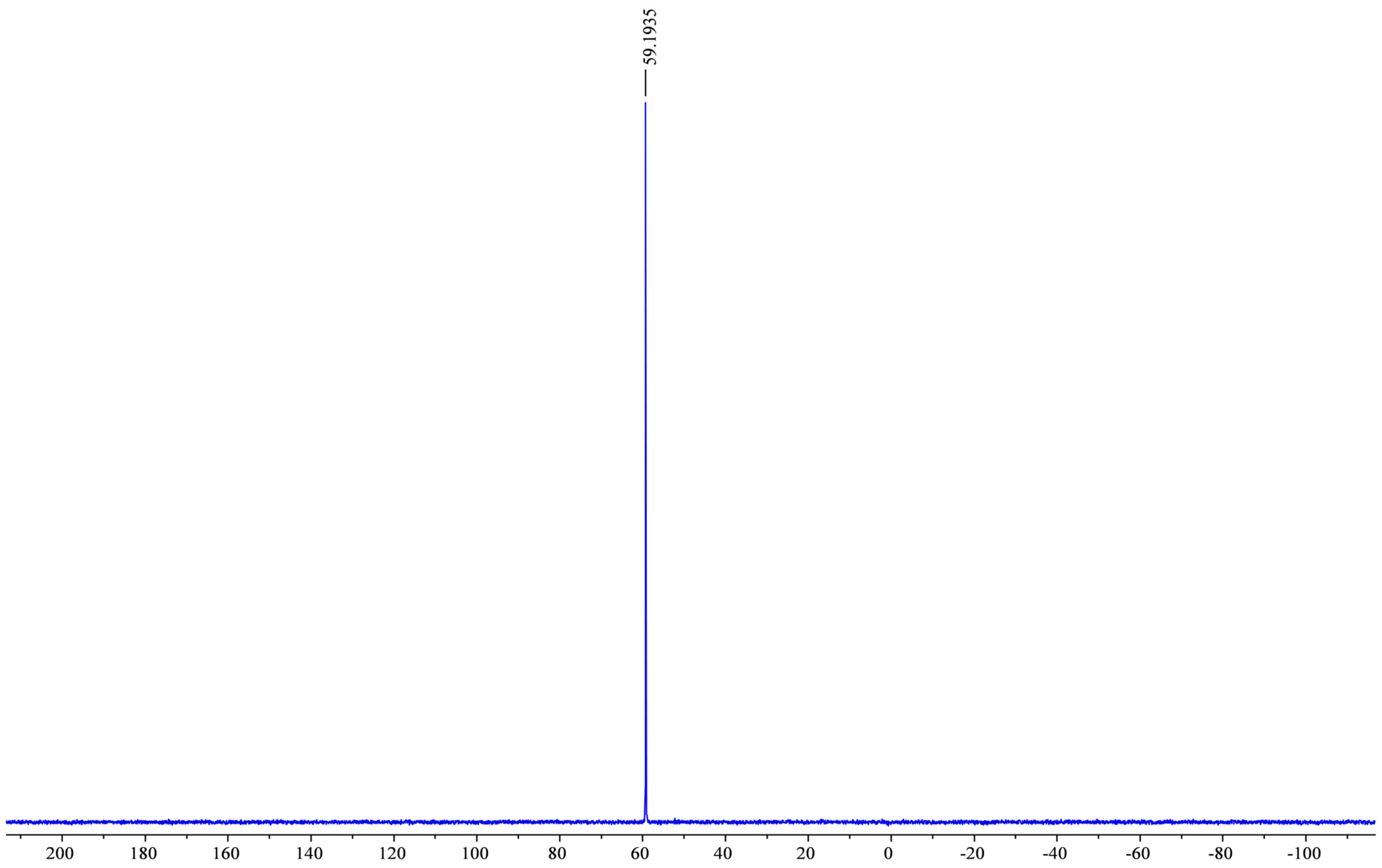

Figure 63S. ^{31}P-{^{1}H} NMR spectrum (400.0 MHz, $CDCl_3$) of $(Et_2N)_2(Ph)P^{+}–C_{10}H_{21}$ I^{-} (**5d**).

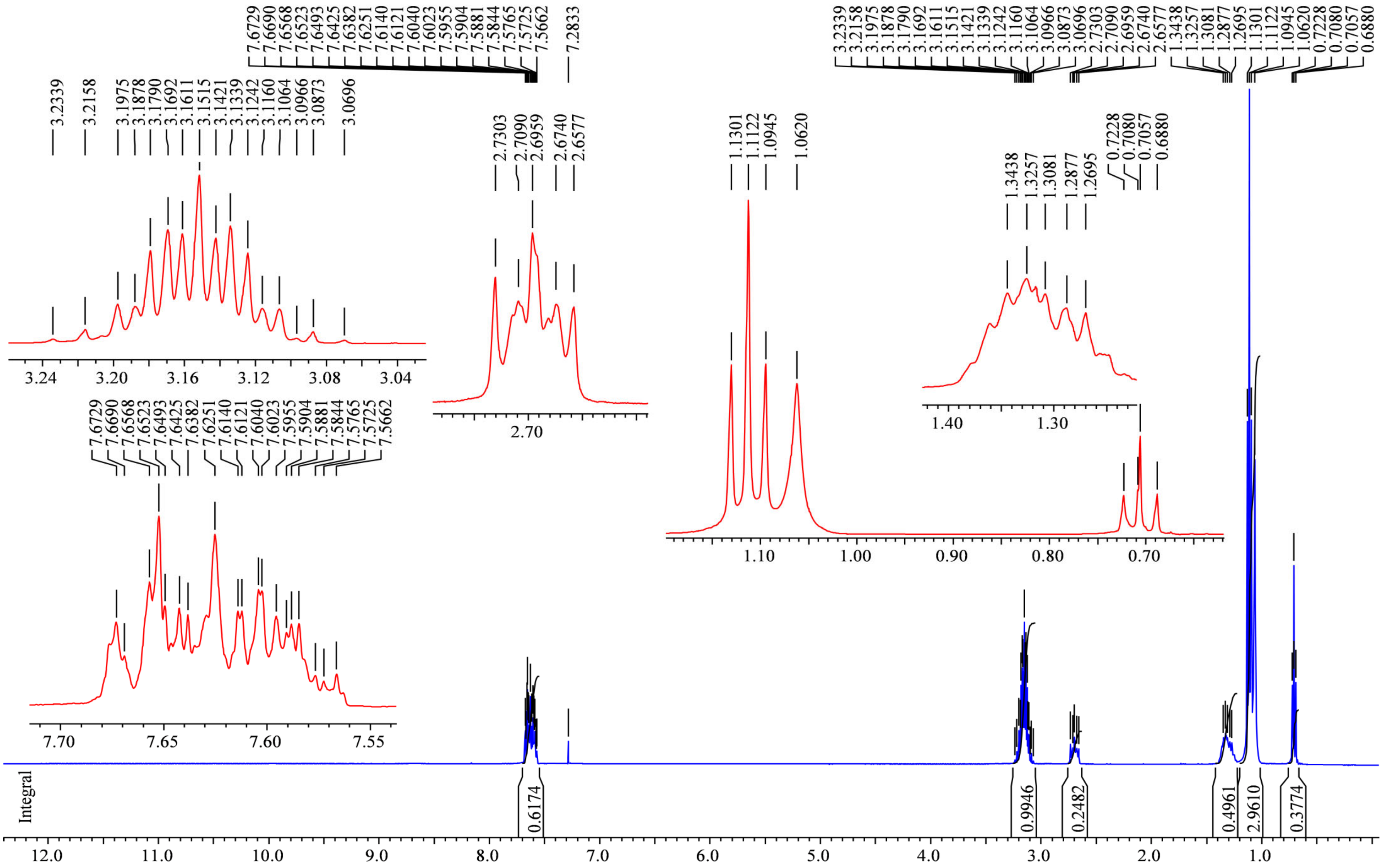

Figure 64S. ^{1}H NMR spectrum (400.0 MHz, $CDCl_3$) of $(Et_2N)_2(Ph)P^+–C_{10}H_{21}$ $I^–$ (**5d**).

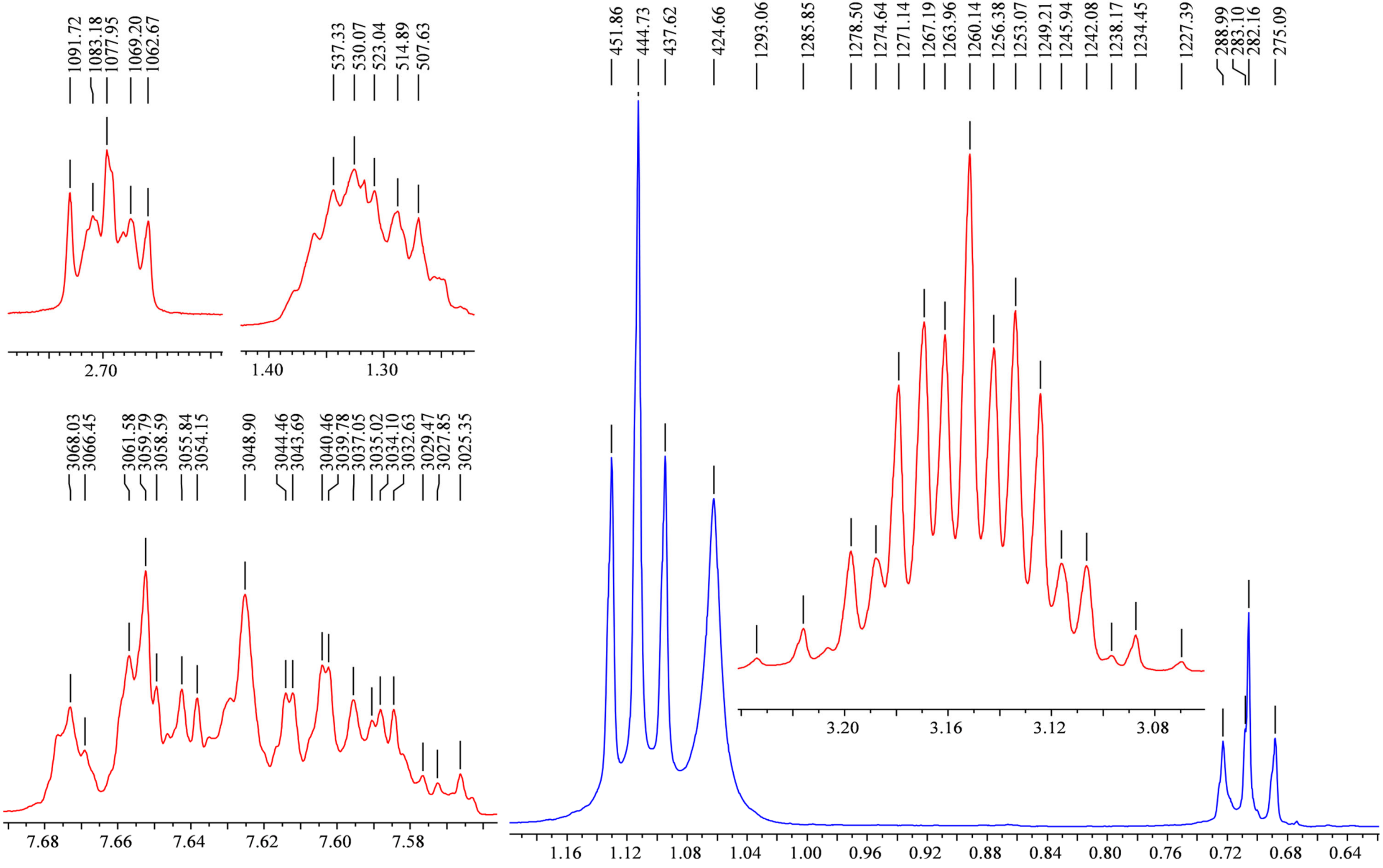

Figure 65S. Fragments of ^{1}H NMR spectrum (400.0 MHz, $CDCl_3$) of $(Et_2N)_2(Ph)P^{+}–C_{10}H_{21}\ I^{–}$ (**5d**).

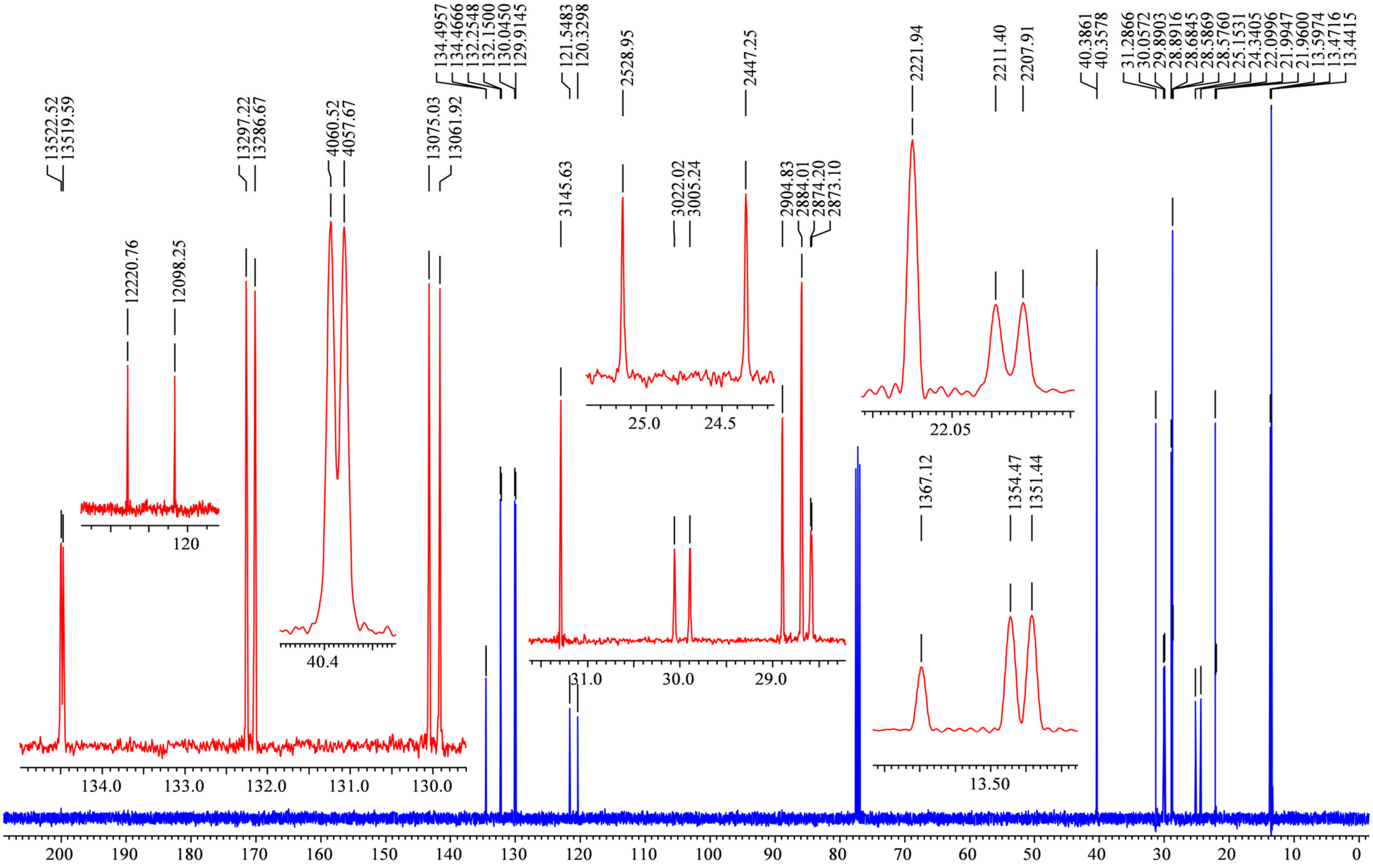

Figure 66S. ^{13}C-{^{1}H} NMR spectrum (100.6 MHz, $CDCl_3$) of $(Et_2N)_2(Ph)P^+$–$C_{10}H_{21}$ I^- (**5d**).

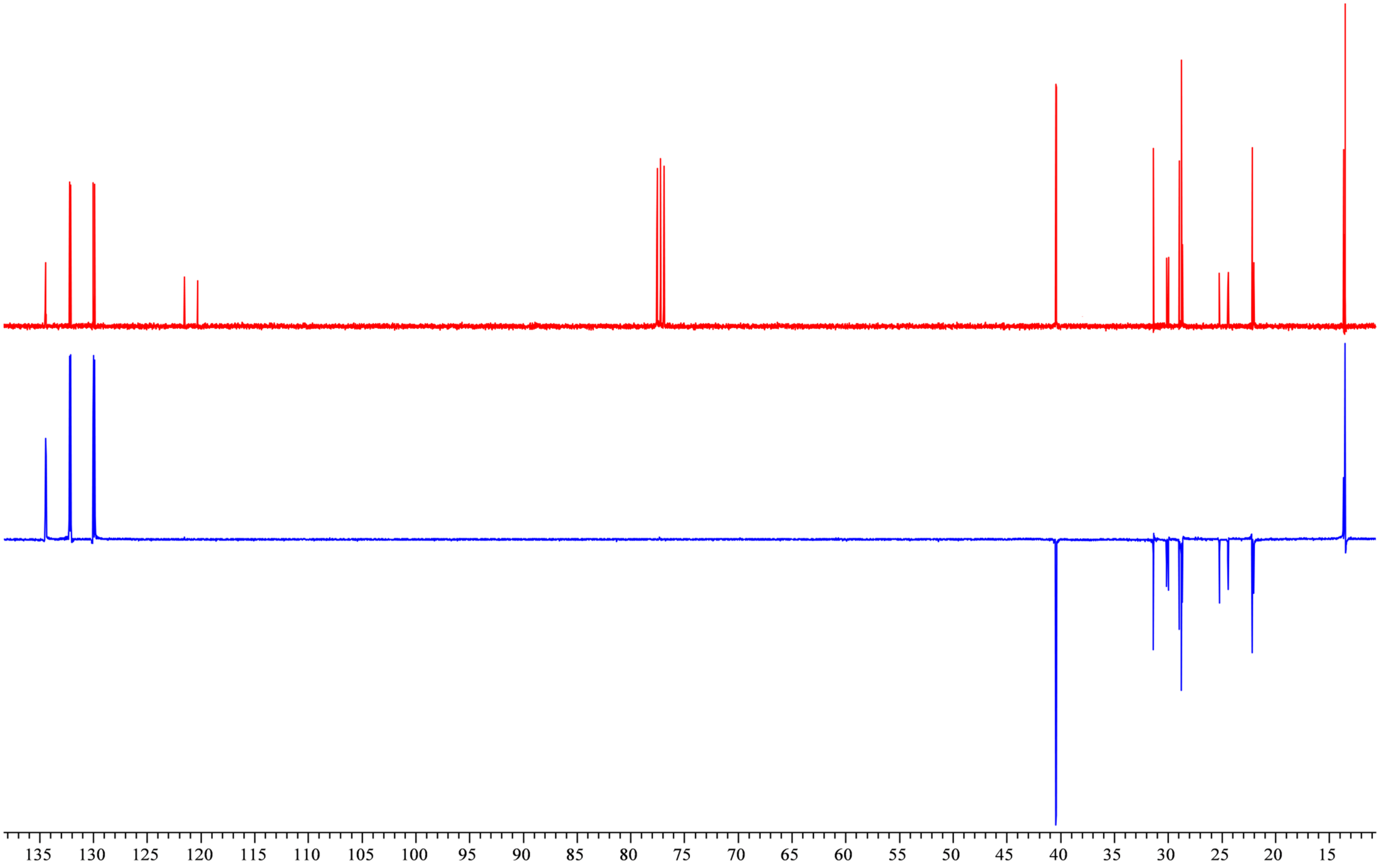

Figure 67S. ^{13}C-{^{1}H} and ^{13}C-{^{1}H}-dept NMR spectra (100.6 MHz, $CDCl_3$) of $(Et_2N)_2(Ph)P^{+}–C_{10}H_{21}$ I^{-} (**5d**).

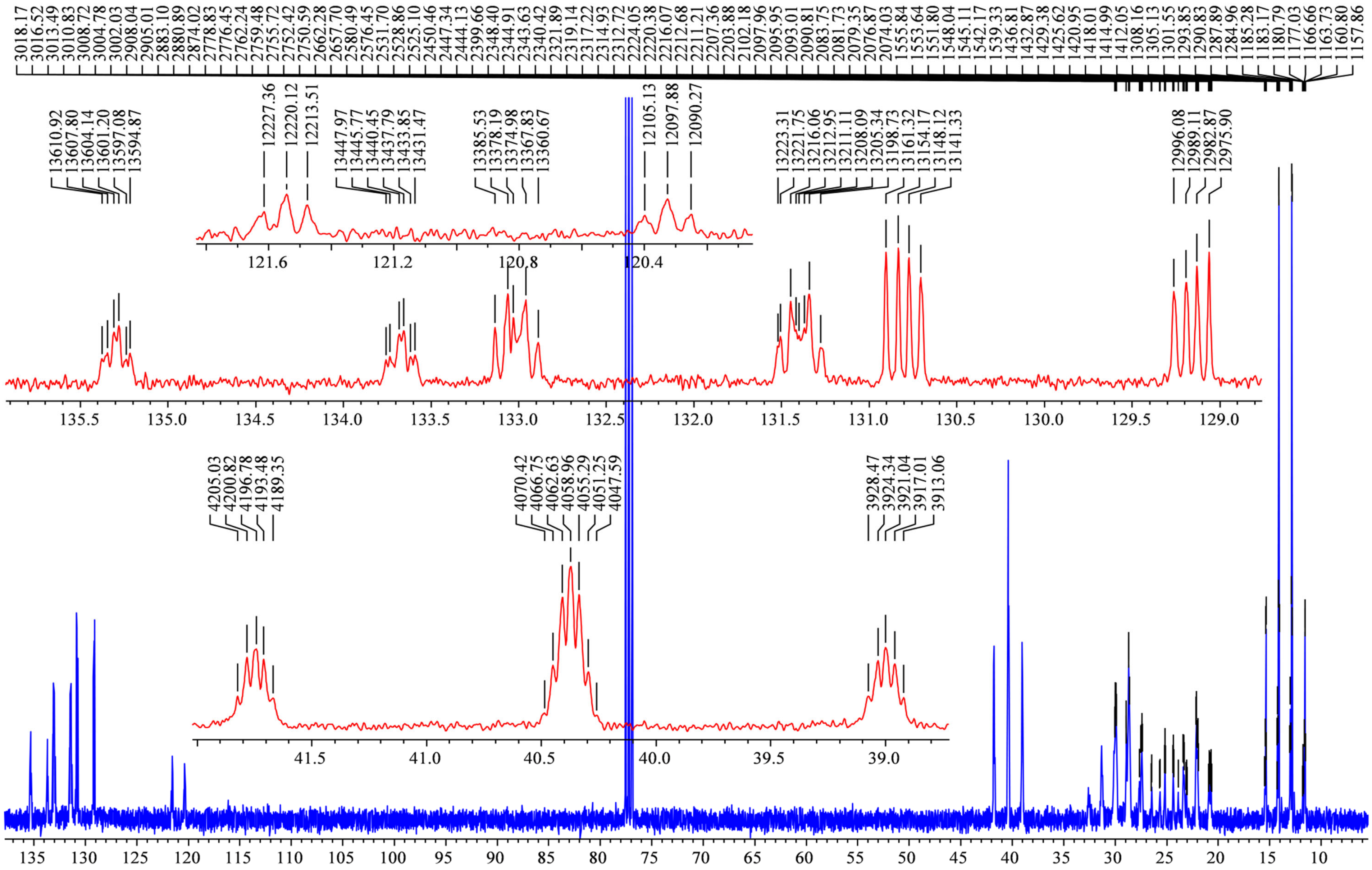

Figure 68S. ^{13}C NMR spectrum (100.6 MHz, $CDCl_3$) of $(Et_2N)_2(Ph)P^+–C_{10}H_{21}$ $I^–$ (**5d**).

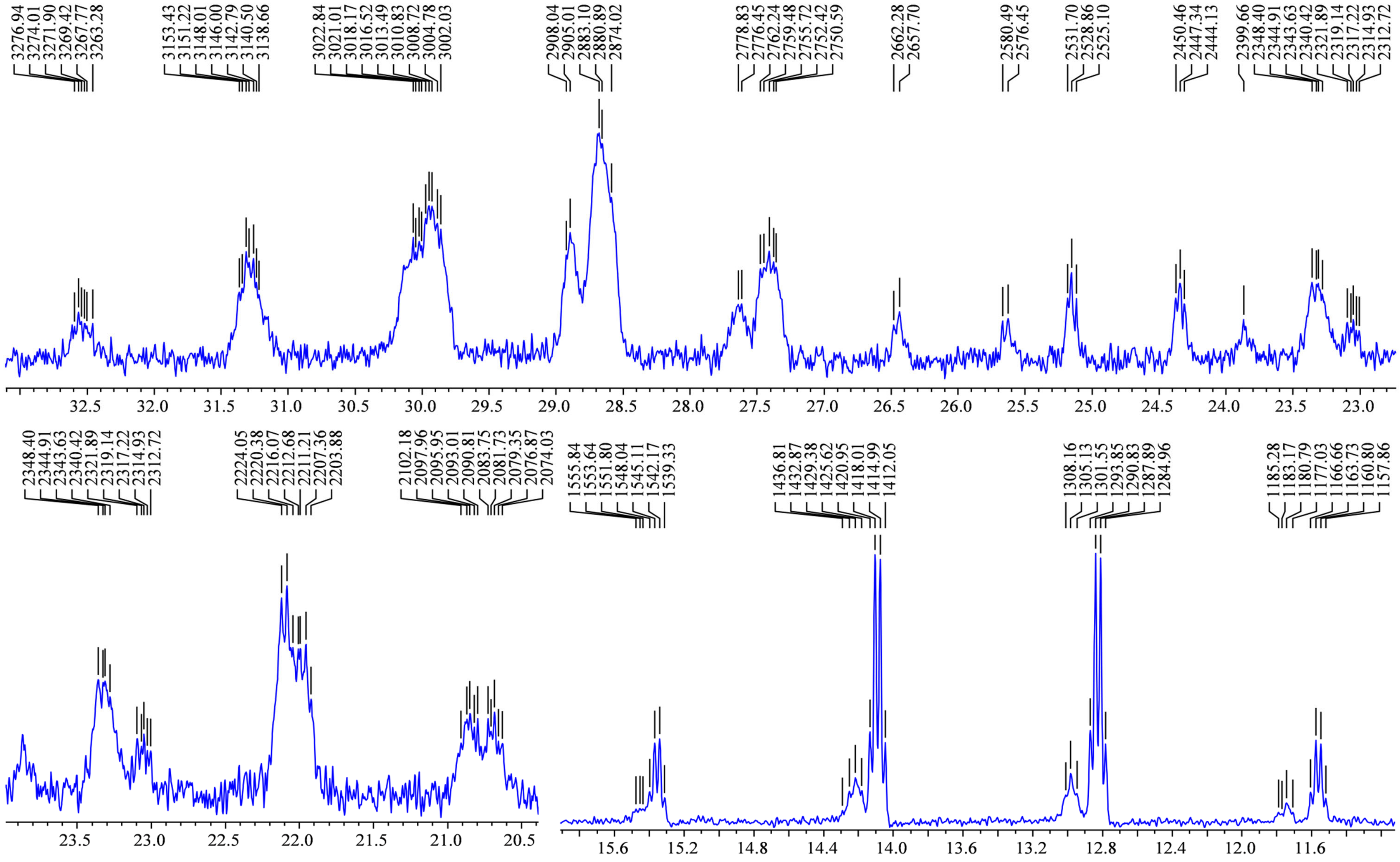

Figure 69S. High-field fragments of ^{13}C NMR spectrum (100.6 MHz, $CDCl_3$) of $(Et_2N)_2(Ph)P^+–C_{10}H_{21}$ $I^–$ (**5d**).

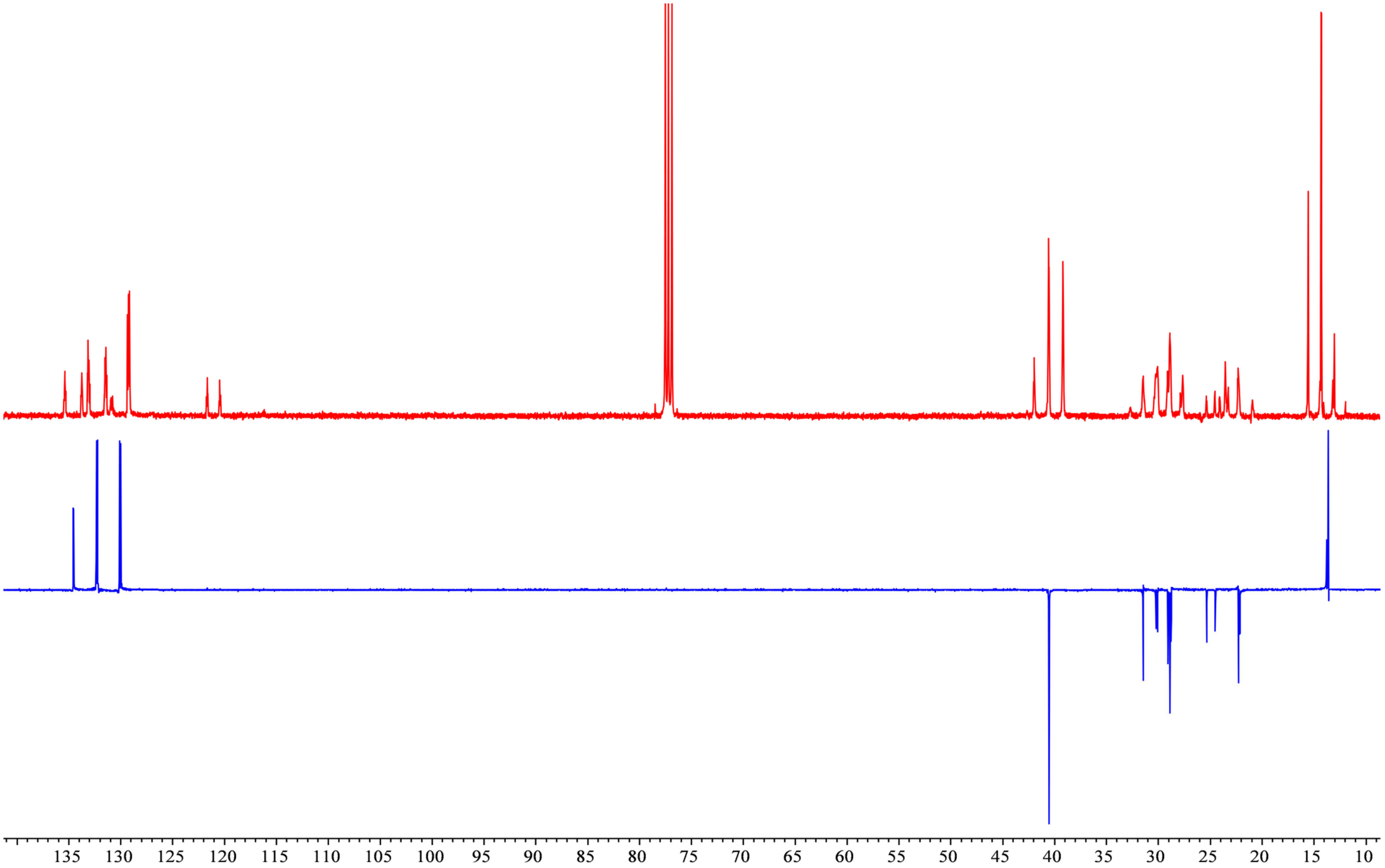

Figure 70S. ^{13}C and ^{13}C-$\{^1H\}$-dept NMR spectra (100.6 MHz, $CDCl_3$) of $(Et_2N)_2(Ph)P^+–C_{10}H_{21}\ I^-$ (**5d**).

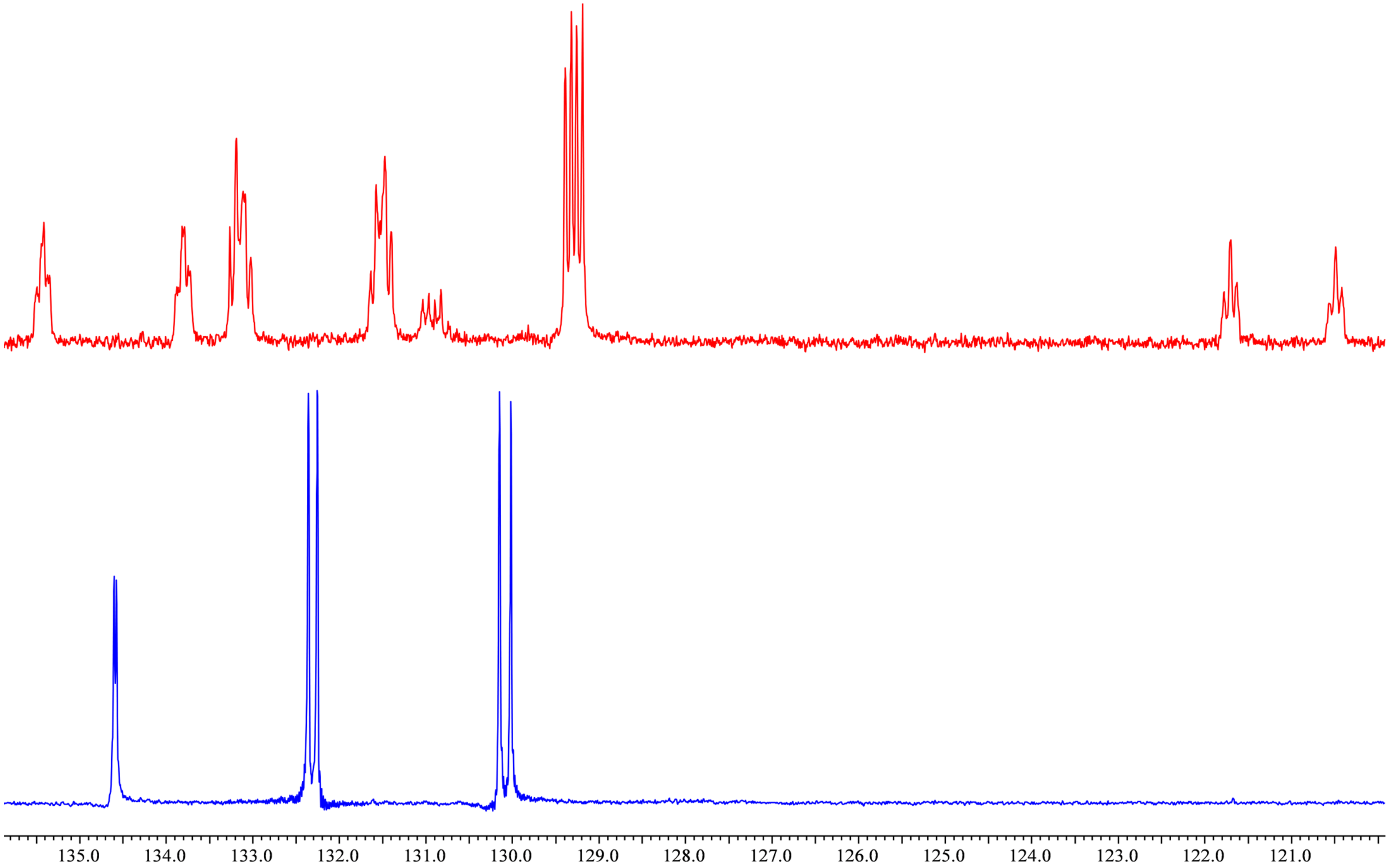

Figure 71S. Low-field region of ^{13}C and ^{13}C-{^{1}H}-dept NMR spectra (100.6 MHz, $CDCl_3$) of $(Et_2N)_2(Ph)P^{+}–C_{10}H_{21}$ $I^{–}$ (**5d**).

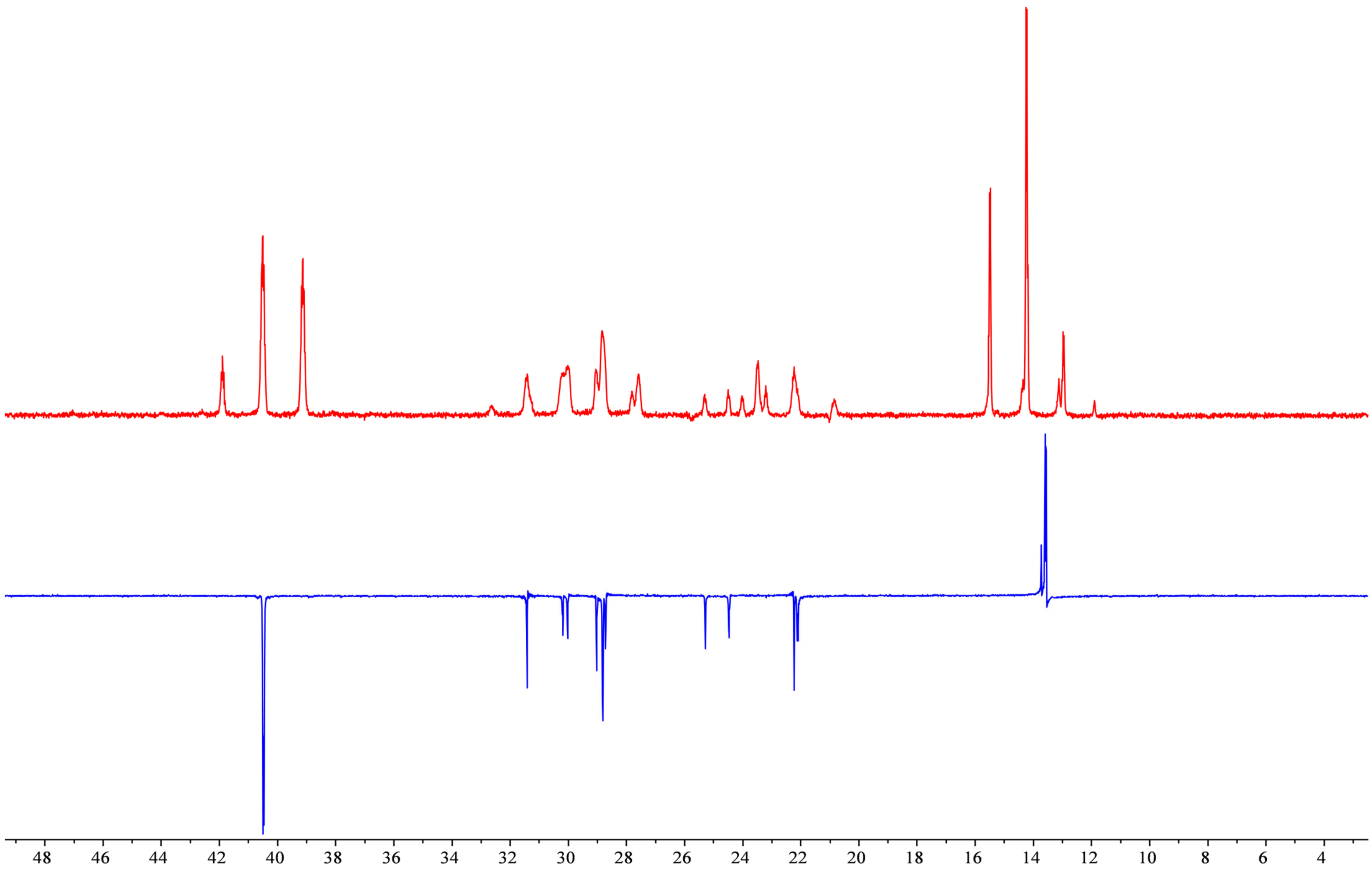

Figure 72S. High-field region of ^{13}C and ^{13}C-{^{1}H}-dept NMR spectra (100.6 MHz, $CDCl_3$) of $(Et_2N)_2(Ph)P^+–C_{10}H_{21}$ $I^–$ (**5d**).

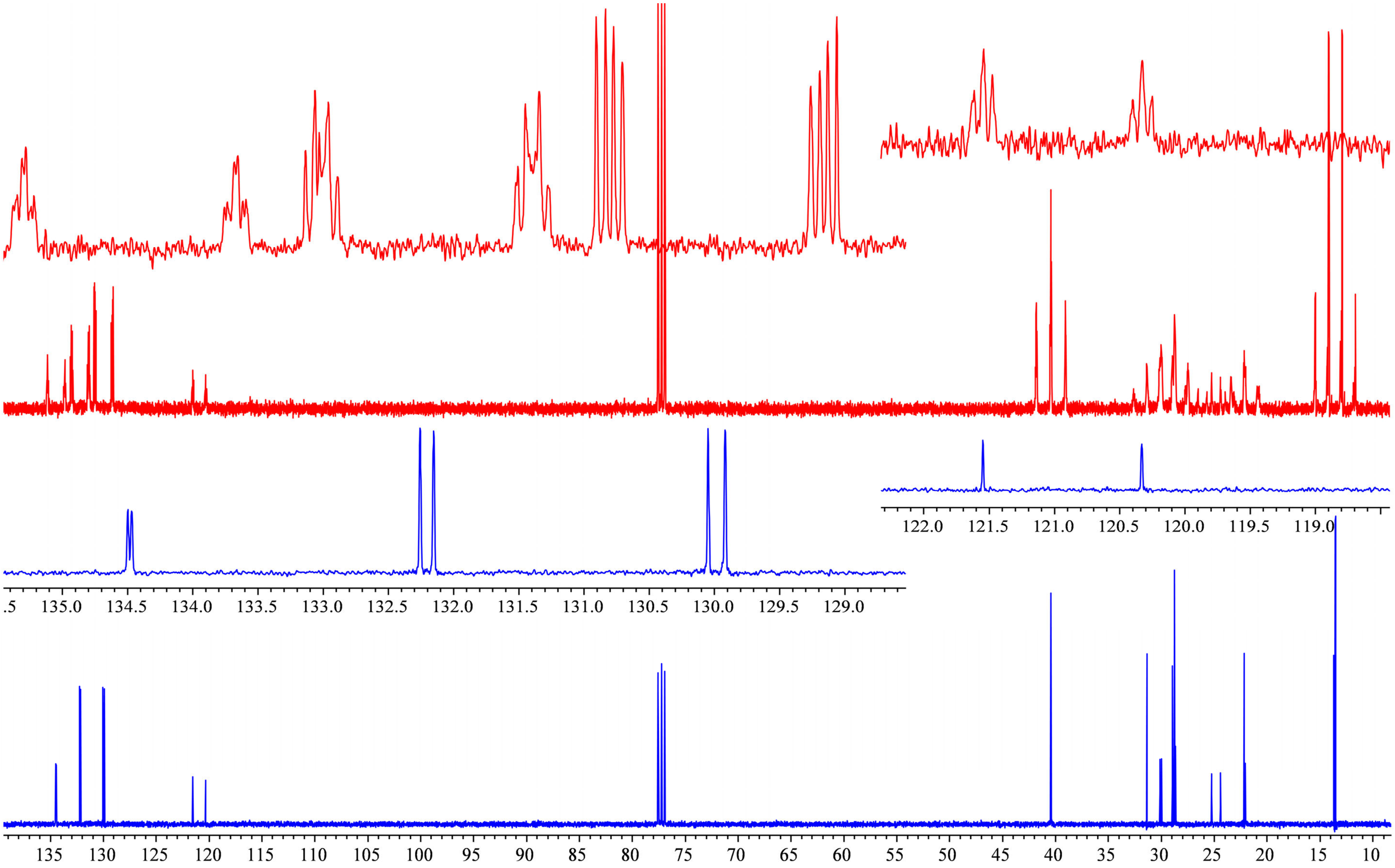

Figure 73S. ^{13}C and ^{13}C-{^{1}H} NMR spectra (100.6 MHz, $CDCl_3$) of $(Et_2N)_2(Ph)P^{+}–C_{10}H_{21}$ $I^{–}$ (**5d**).

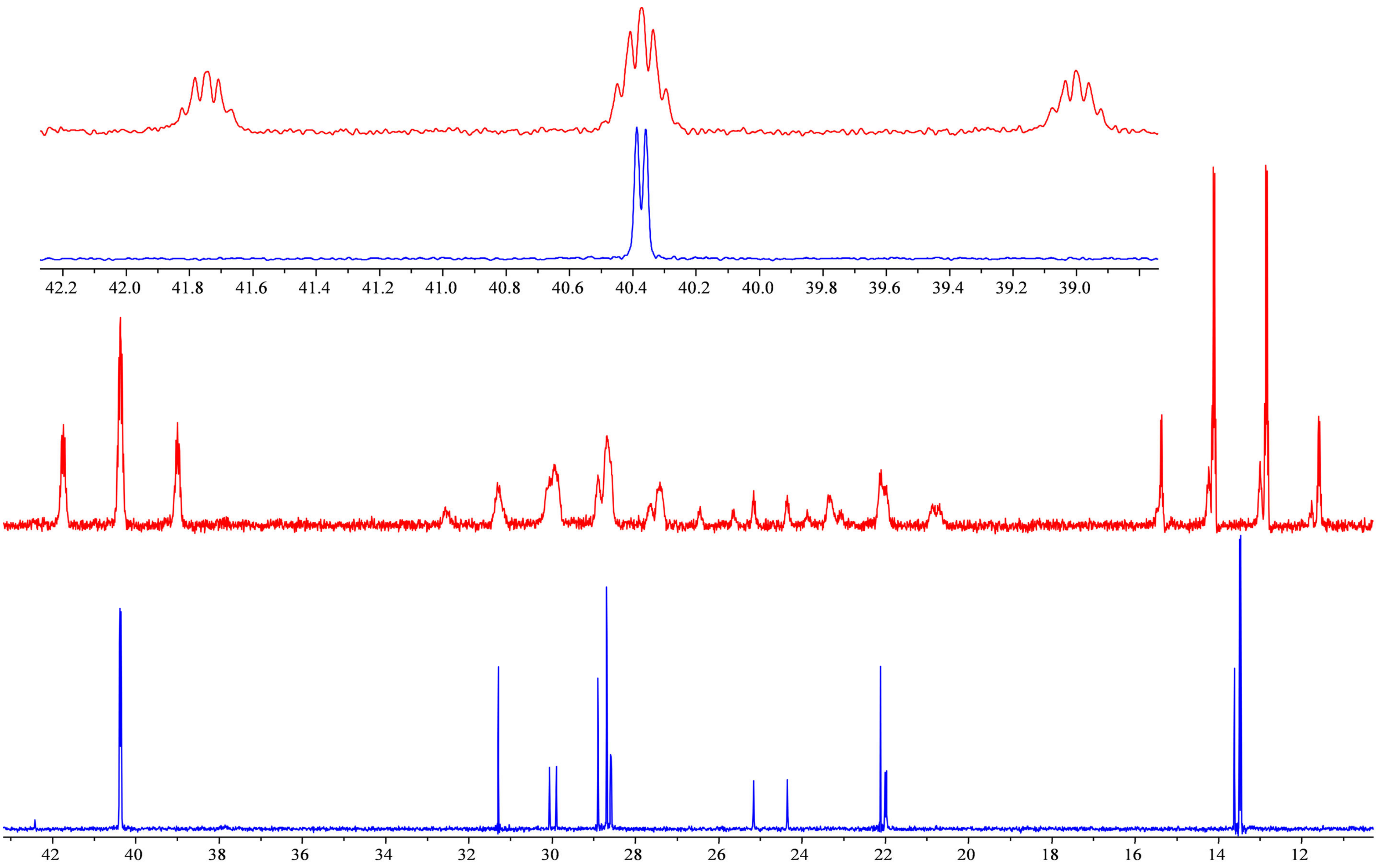

Figure 74S. High-field region of ^{13}C and ^{13}C-{^{1}H} NMR spectra (100.6 MHz, $CDCl_3$) of $(Et_2N)_2(Ph)P^+–C_{10}H_{21}\ I^–$ (**5d**).

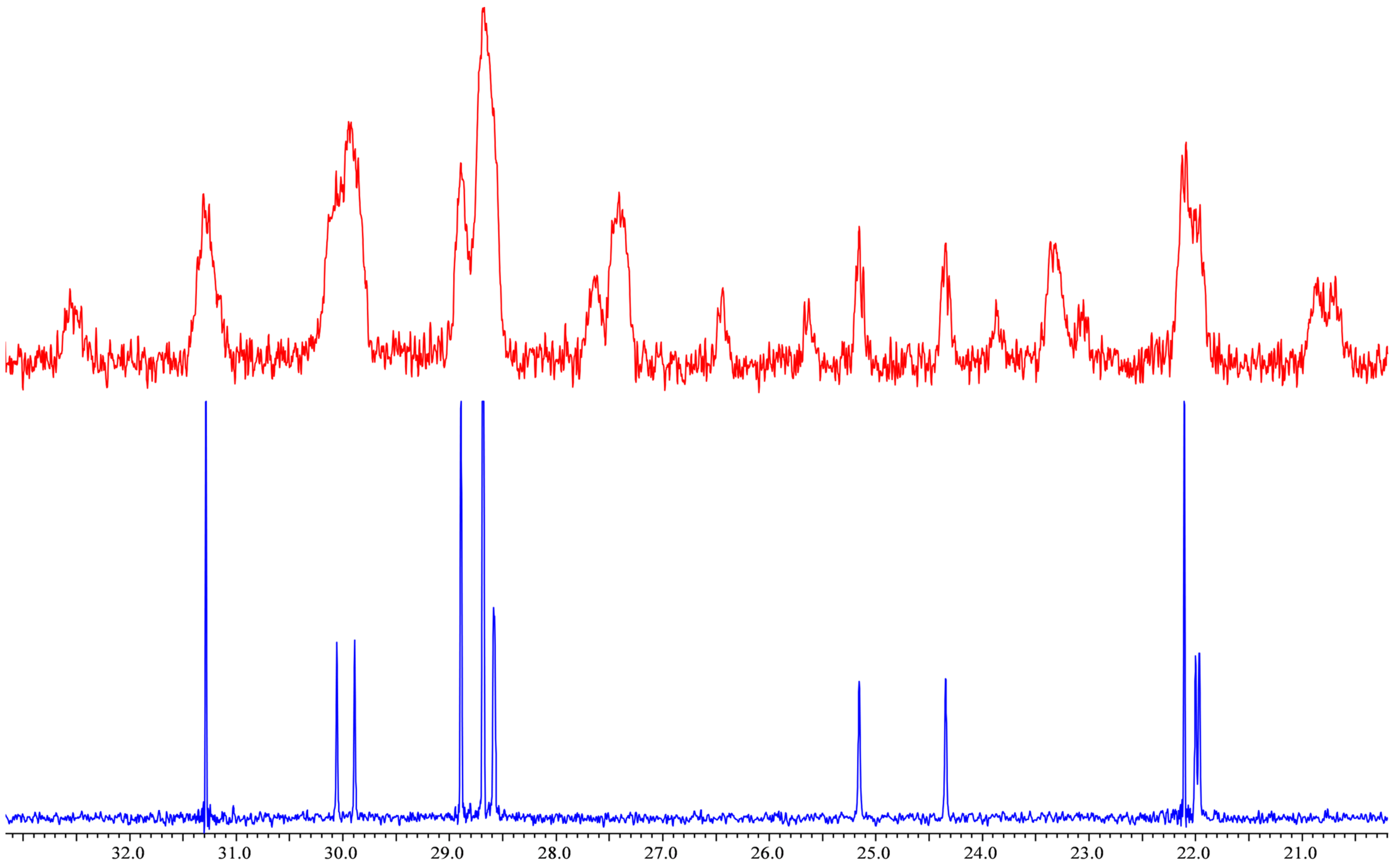

Figure 75S. The 20-33 ppm region of ^{13}C and ^{13}C-{^{1}H} NMR spectra (100.6 MHz, $CDCl_3$) of $(Et_2N)_2(Ph)P^+–C_{10}H_{21}$ $I^–$ (**5d**).

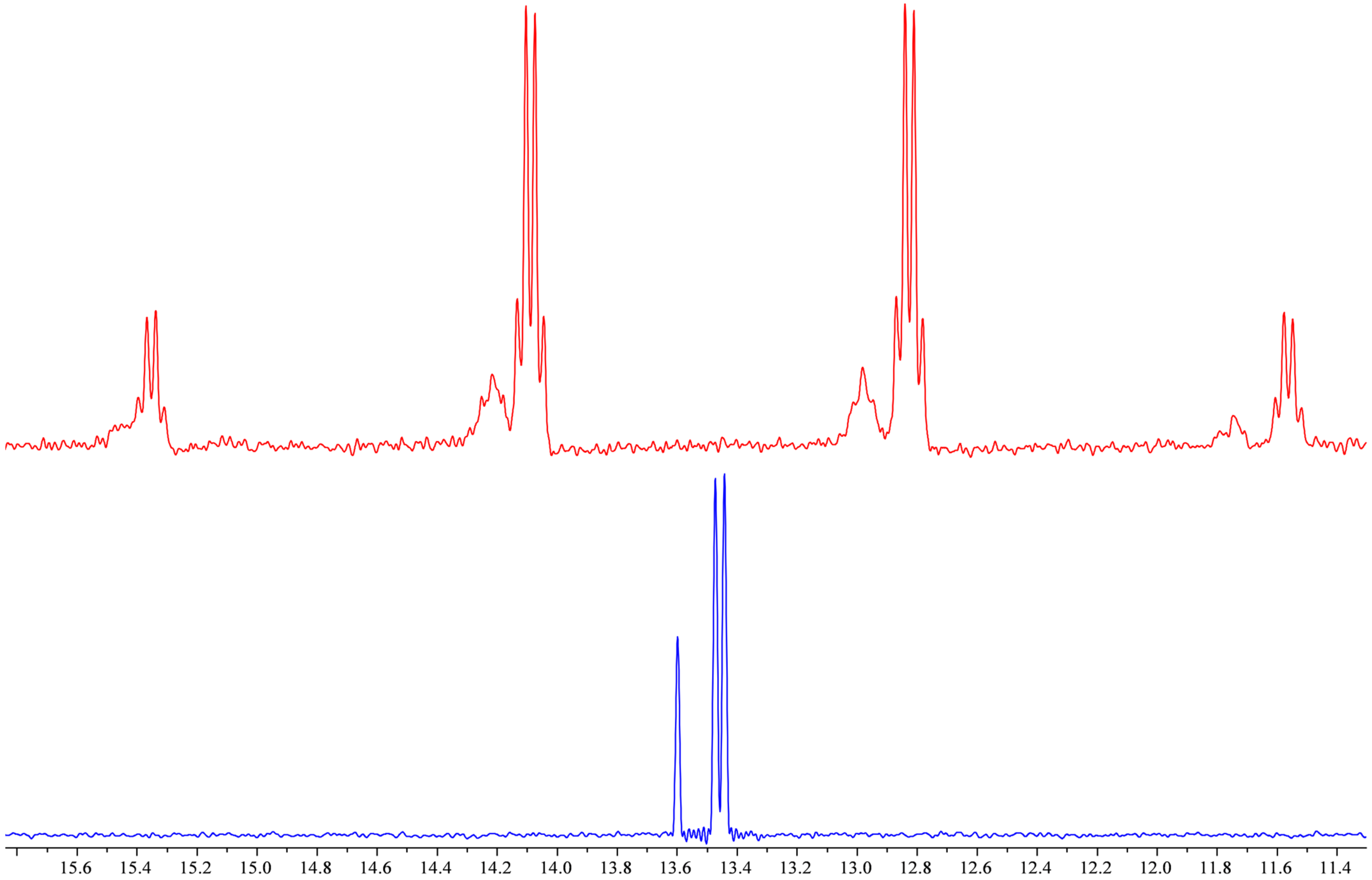

Figure 76S. The 11-16 ppm region of ^{13}C and ^{13}C-{^{1}H} NMR spectra (100.6 MHz, $CDCl_3$) of $(Et_2N)_2(Ph)P^+–C_{10}H_{21}$ $I^−$ (**5d**).

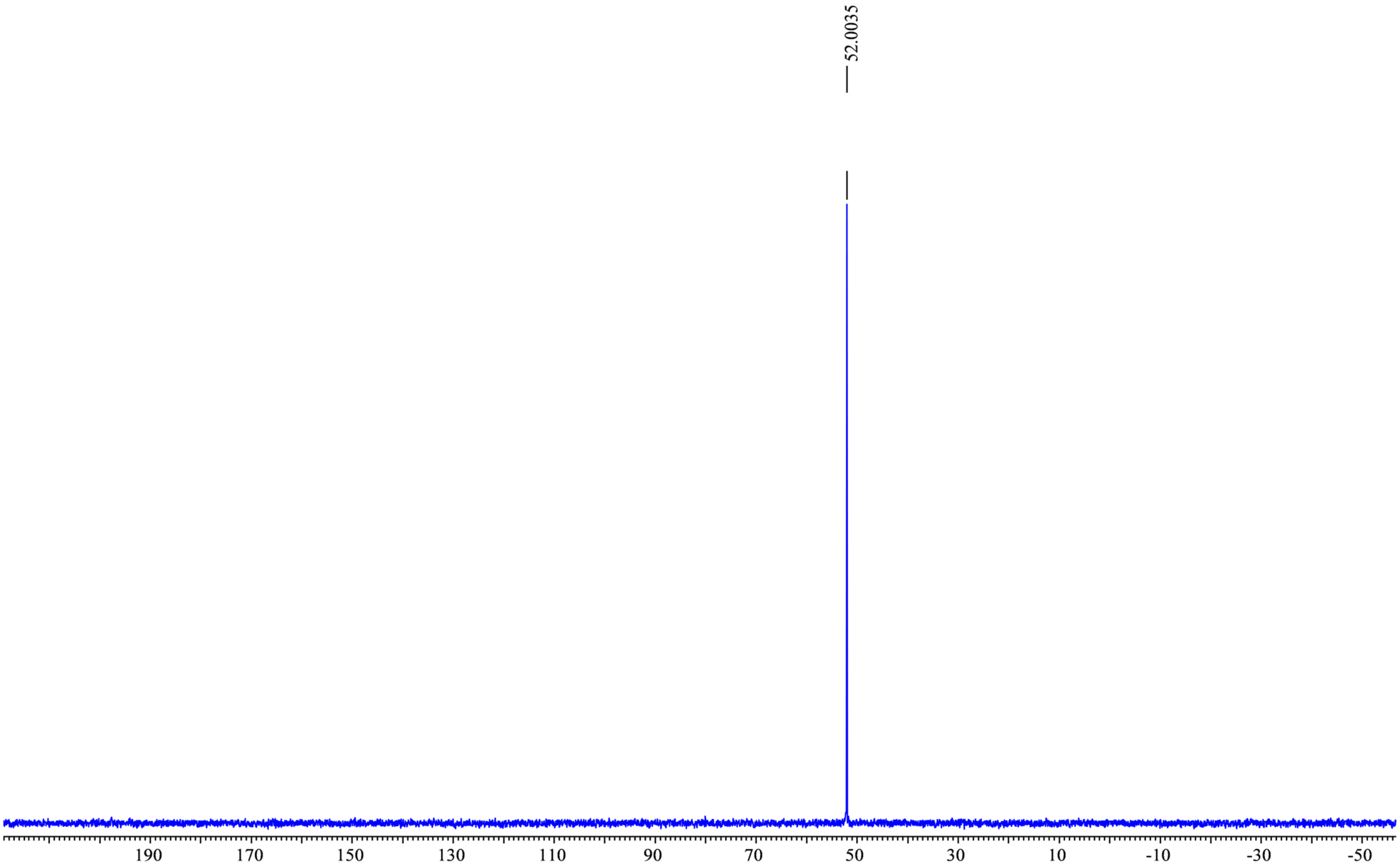

Figure 77S. ^{31}P-{^{1}H} NMR spectrum (162.0 MHz, $CDCl_3$) of $(Et_2N)Ph_2P^{+}–C_6H_{13}$ I^{-} (**6a**).

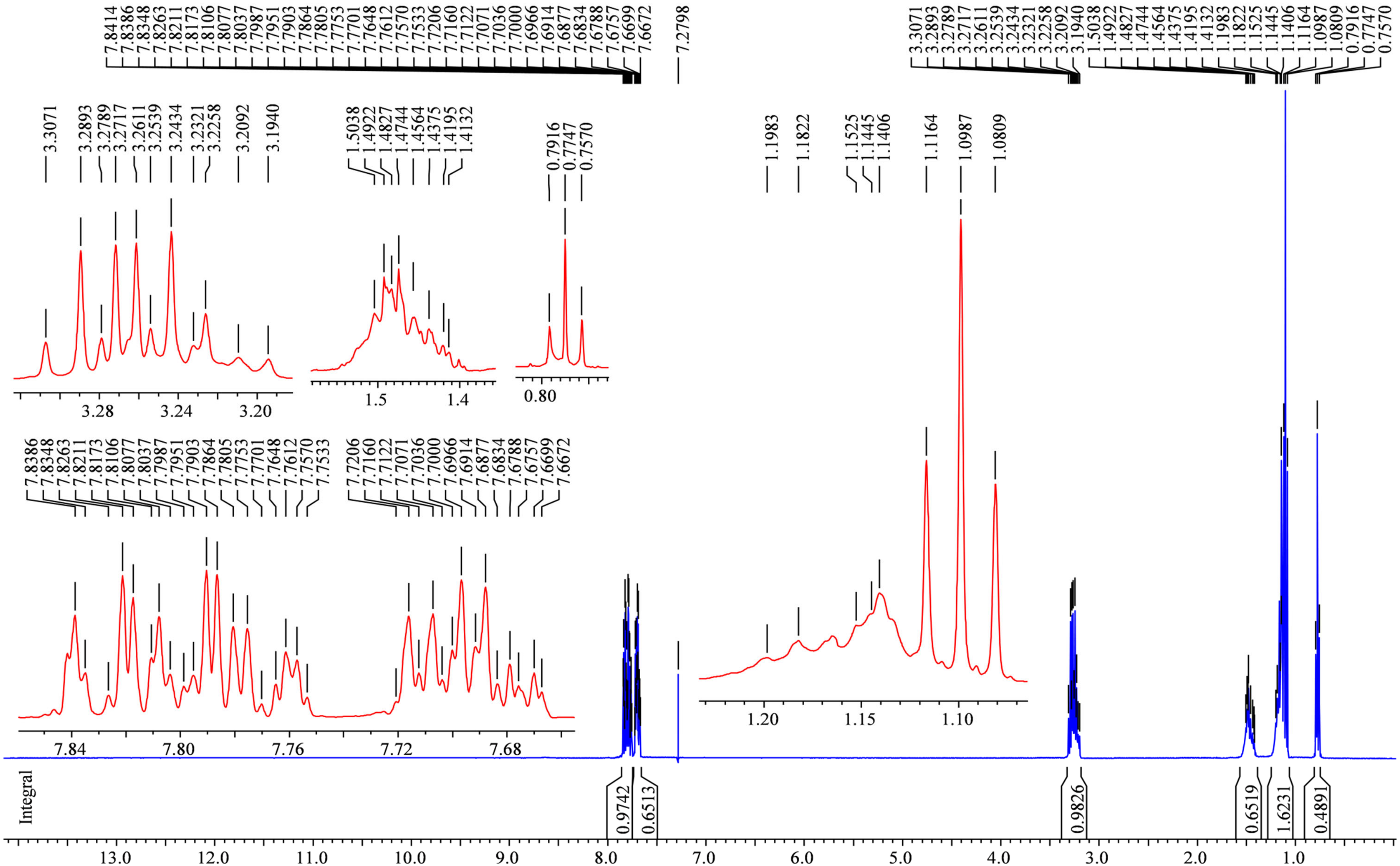

Figure 78S. ^{1}H NMR spectrum (400.0 MHz, $CDCl_3$) of $(Et_2N)Ph_2P^{+}–C_6H_{13}$ $I^{–}$ (**6a**).

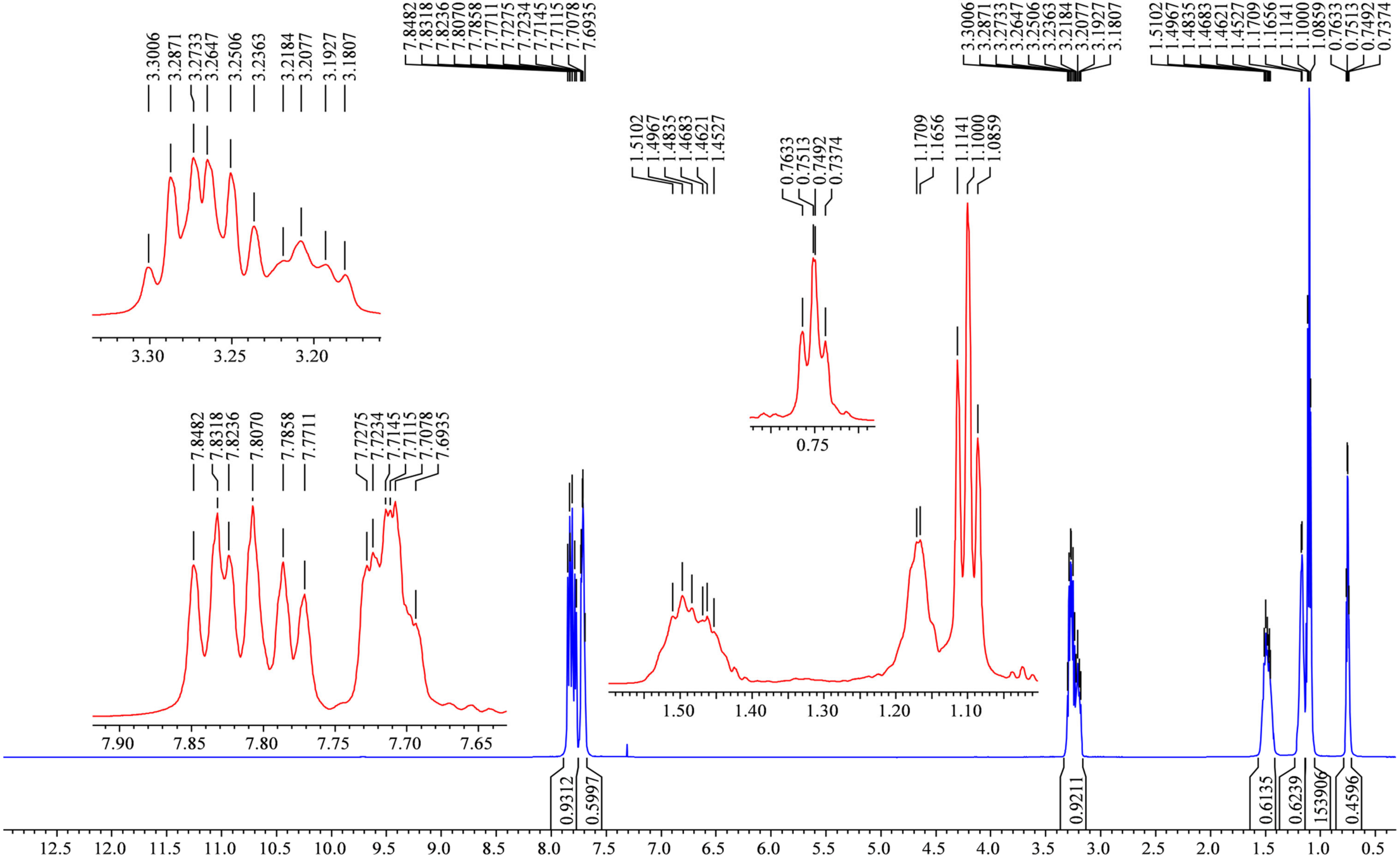

Figure 79S. ^{1}H NMR spectrum (500.0 MHz, $CDCl_3$) of $(Et_2N)Ph_2P^+–C_6H_{13}\ I^−$ (**6a**).

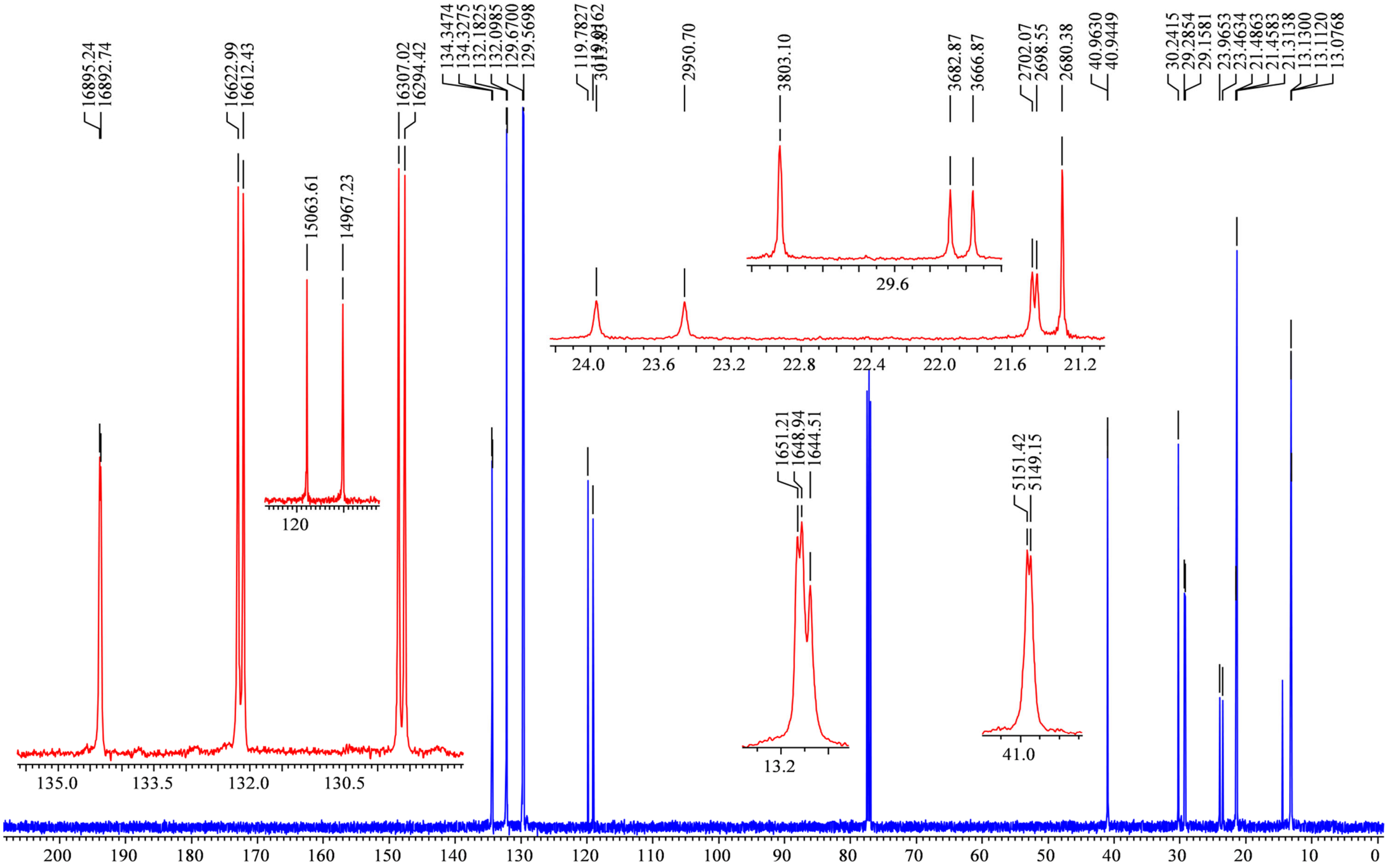

Figure 80S. ^{13}C-{^{1}H} NMR spectrum (125.0 MHz, $CDCl_3$) of $(Et_2N)Ph_2P^+–C_6H_{13}\ I^–$ (**6a**).

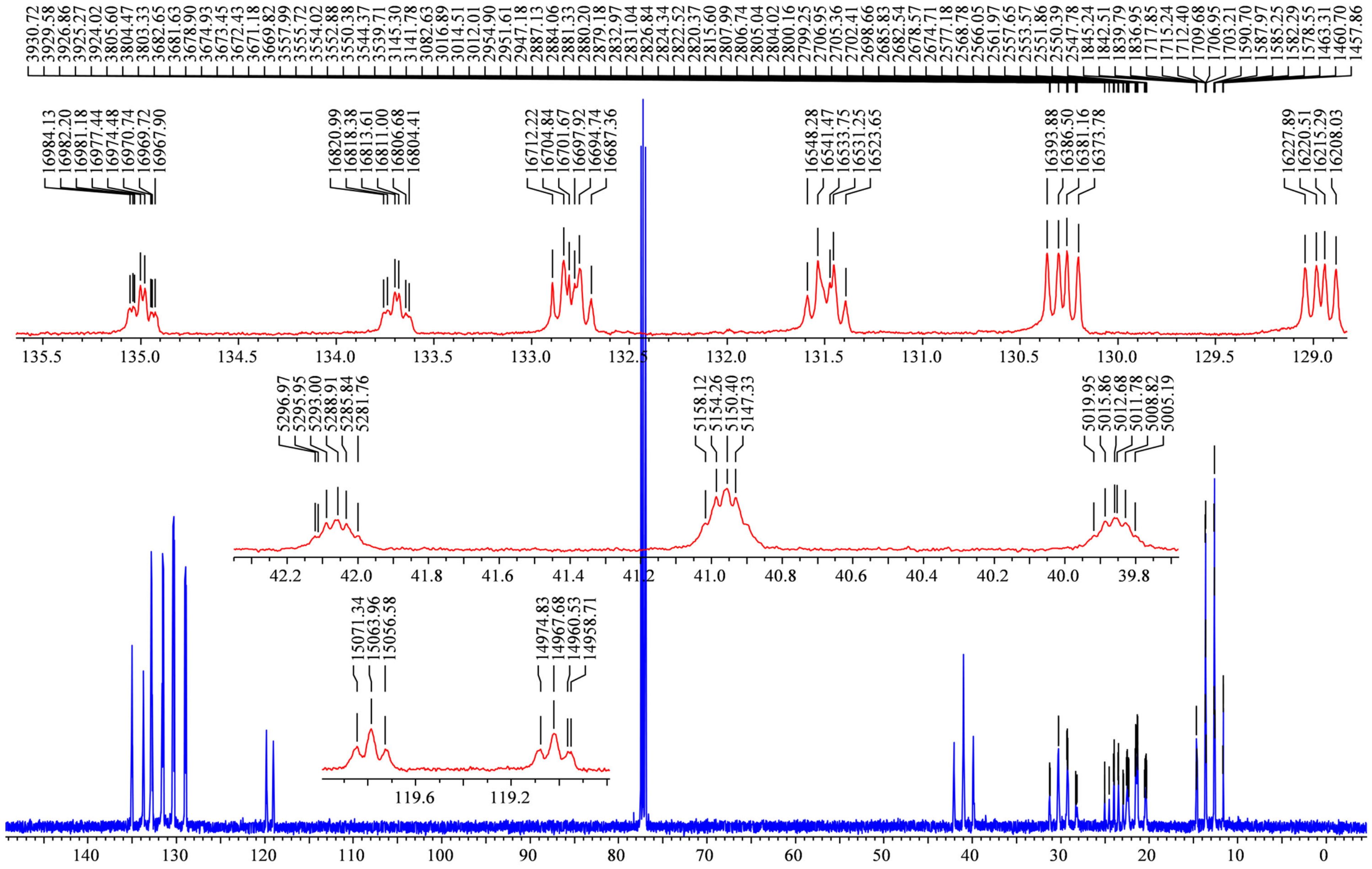

Figure 81S. ^{13}C NMR spectrum (125.8 MHz, $CDCl_3$) of $(Et_2N)Ph_2P^+–C_6H_{13}\ I^-$ (**6a**).

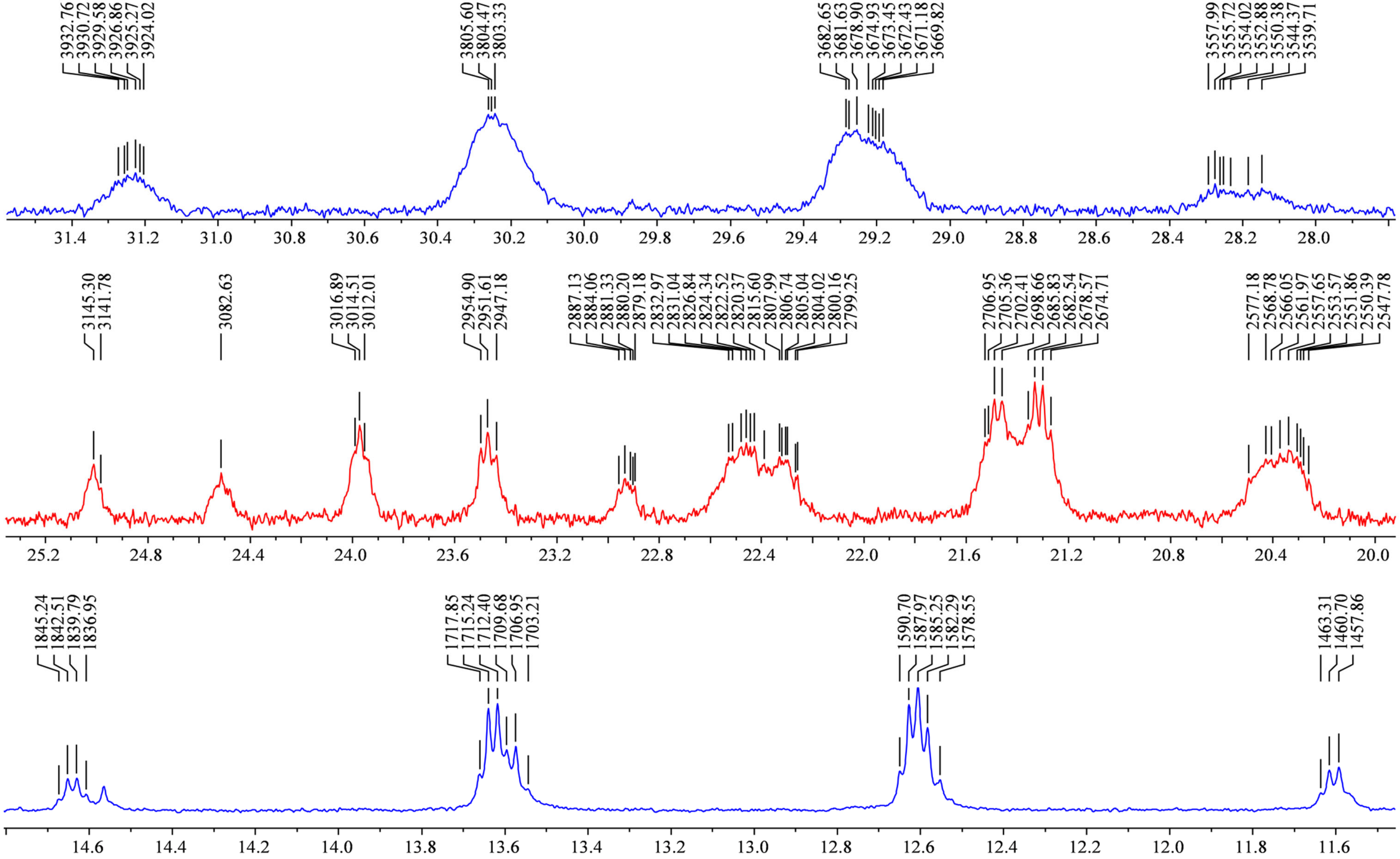

Figure 82S. High-field fragments of ^{13}C NMR spectrum (125.8 MHz, $CDCl_3$) of $(Et_2N)Ph_2P^+–C_6H_{13}$ $I^−$ (**6a**).

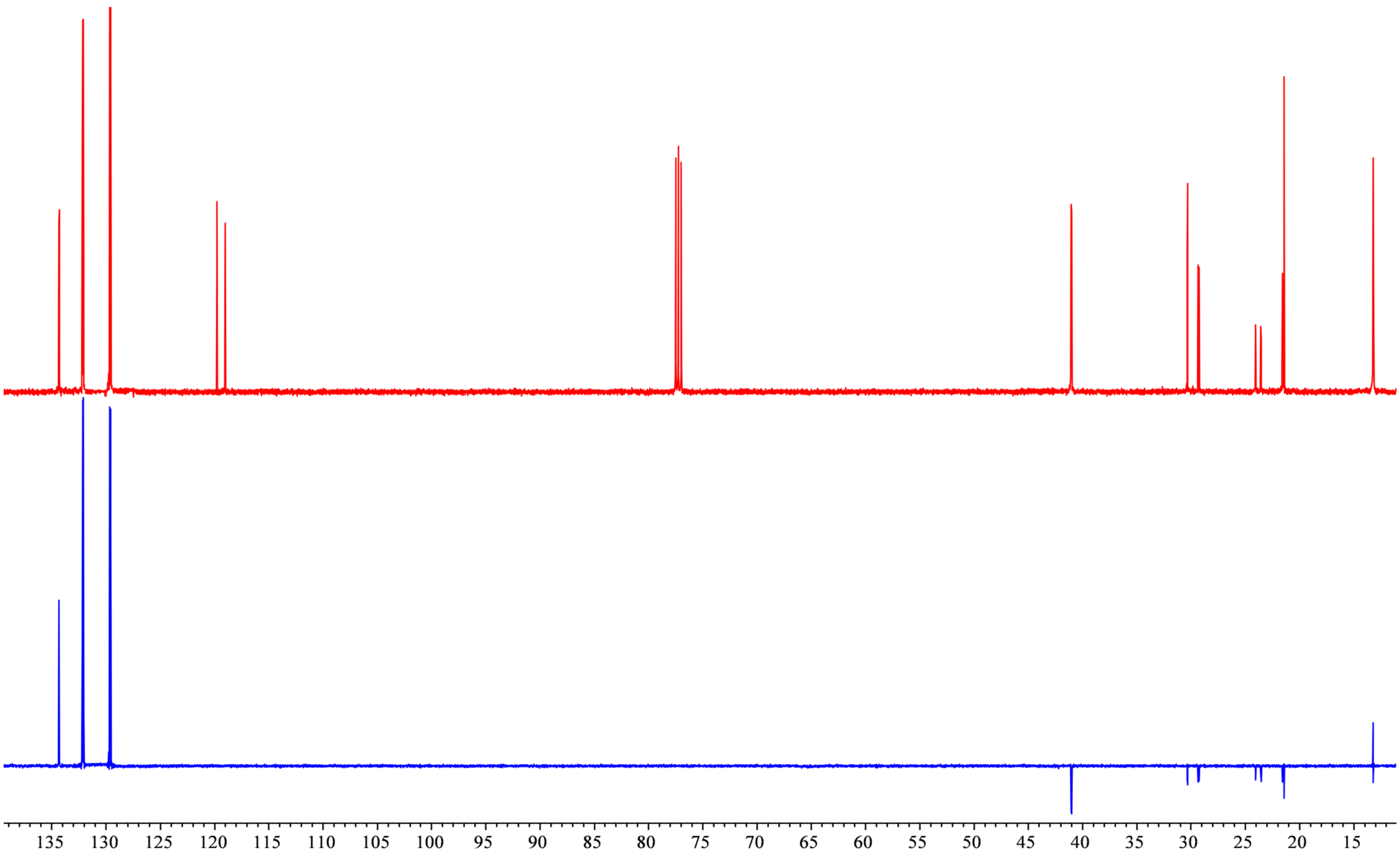

Figure 83S. ^{13}C-{^{1}H} and ^{13}C-{^{1}H}-dept NMR spectra (125.8 MHz, $CDCl_3$) of $(Et_2N)Ph_2P^+–C_6H_{13}\ I^-$ (**6a**).

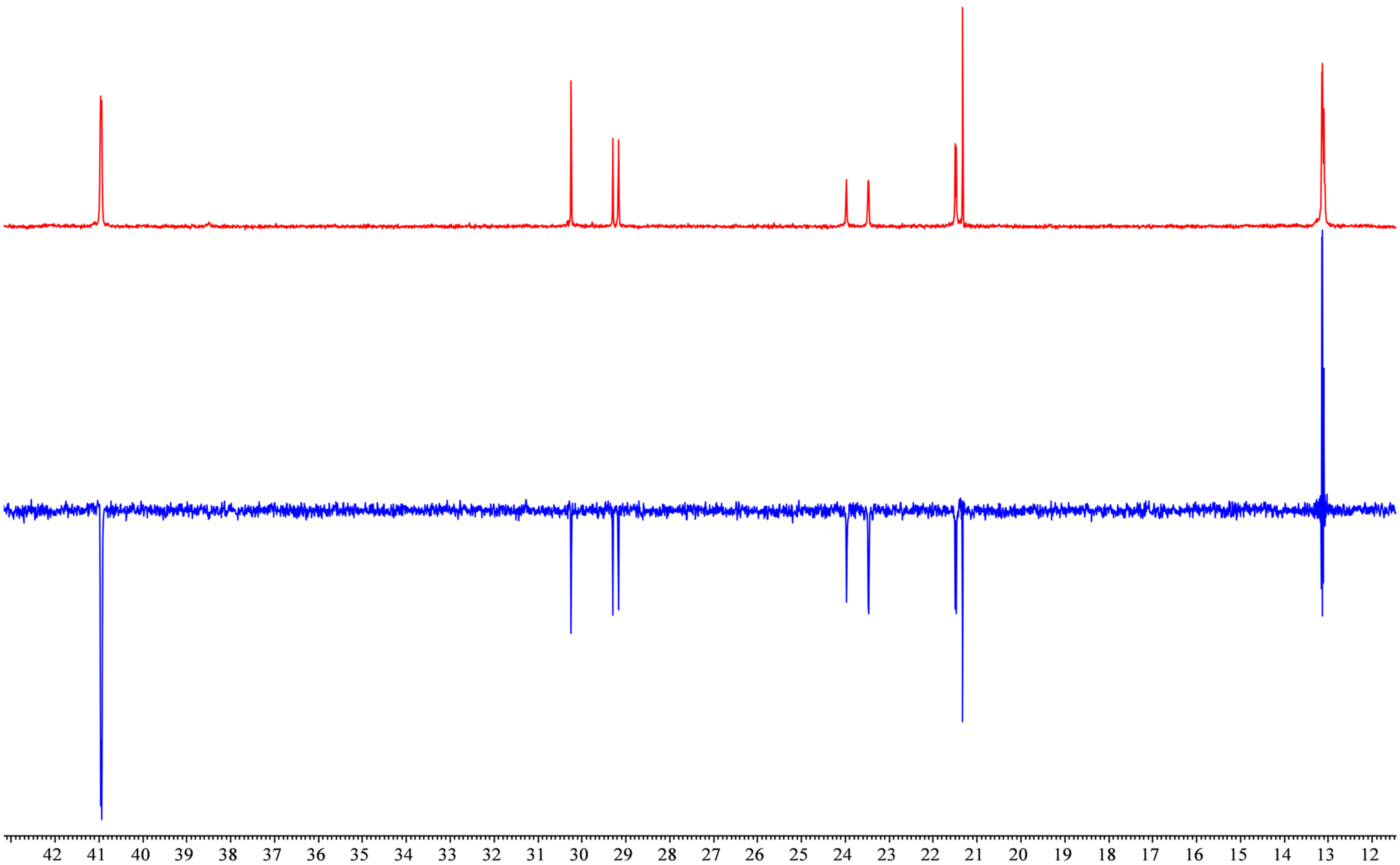

Figure 84S. High-field fragment of ^{13}C-{^{1}H} and ^{13}C-{^{1}H}-dept NMR spectra (125.8 MHz, $CDCl_3$) of $(Et_2N)Ph_2P^+–C_6H_{13}\ I^–$ (**6a**).

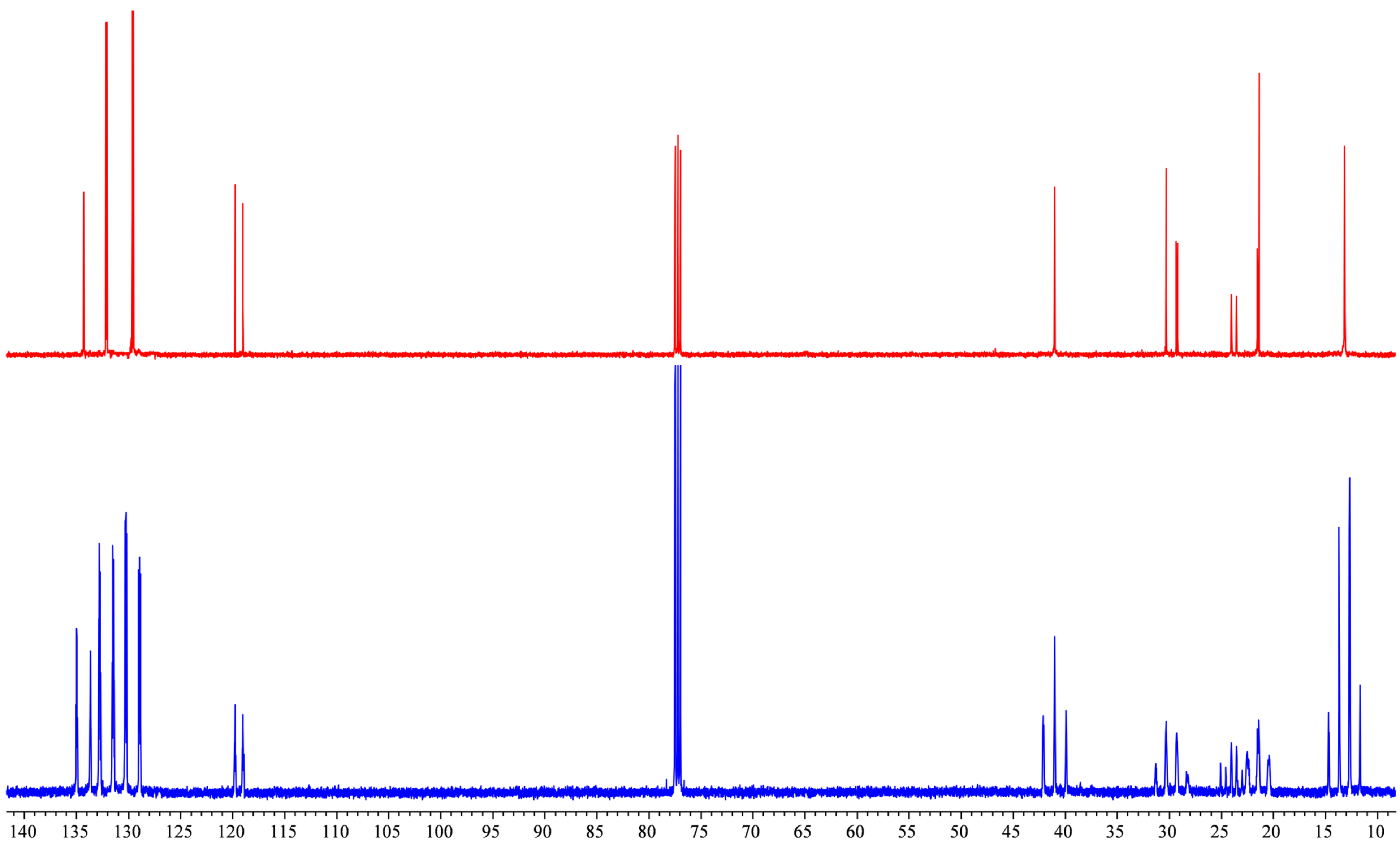

Figure 85S. ^{13}C-{^{1}H} and ^{13}C NMR spectra (125.8 MHz, $CDCl_3$) of $(Et_2N)Ph_2P^{+}–C_6H_{13}\ I^{–}$ (**6a**).

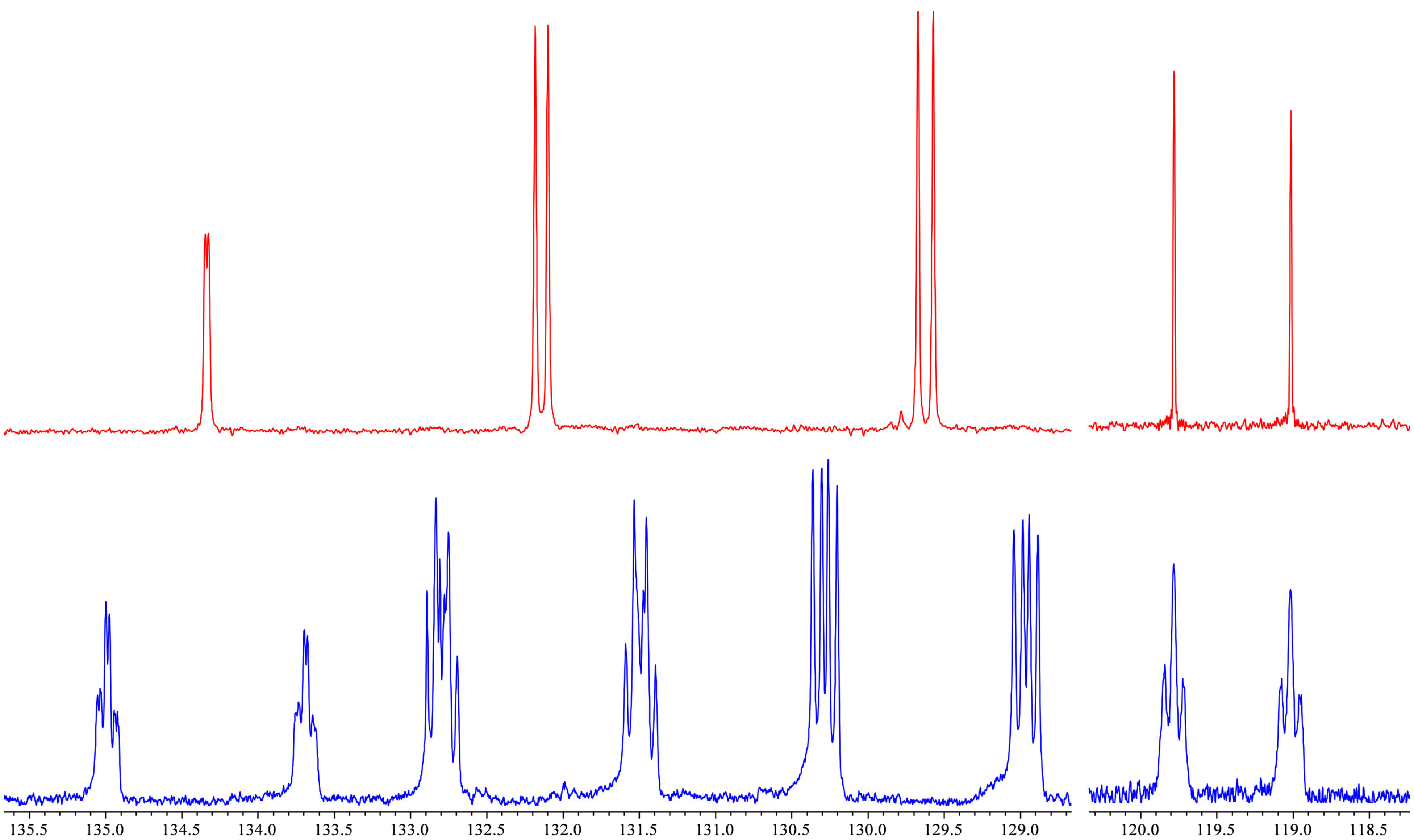

Figure 86S. Low-field fragment of ^{13}C-{^{1}H} and ^{13}C NMR spectra (125.8 MHz, $CDCl_3$) of $(Et_2N)Ph_2P^{+}–C_6H_{13}$ I^{-} (**6a**).

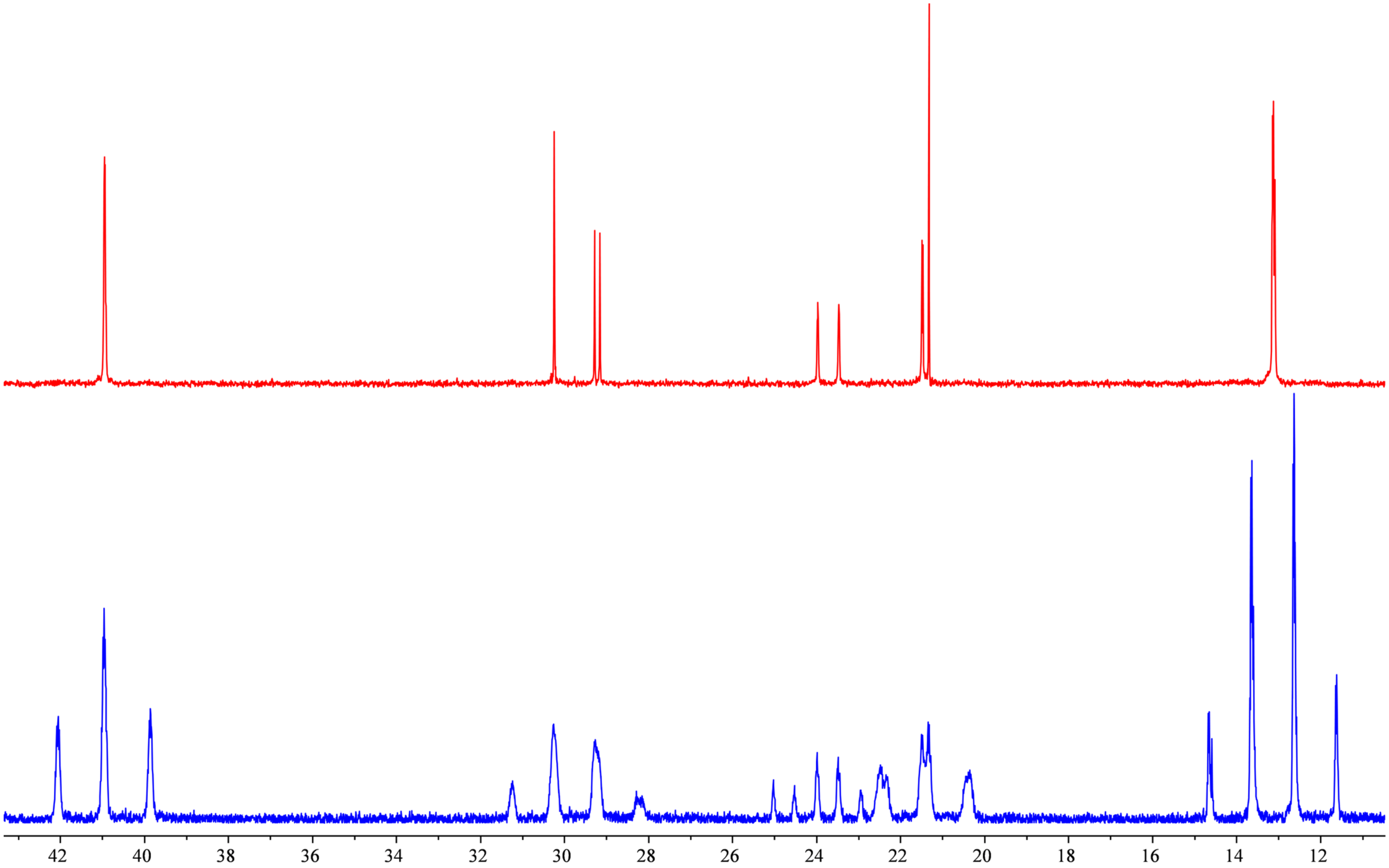

Figure 87S. High-field fragment of ^{13}C-{^{1}H} and ^{13}C NMR spectra (125.8 MHz, $CDCl_3$) of $(Et_2N)Ph_2P^{+}–C_6H_{13}$ $I^{–}$ (**6a**).

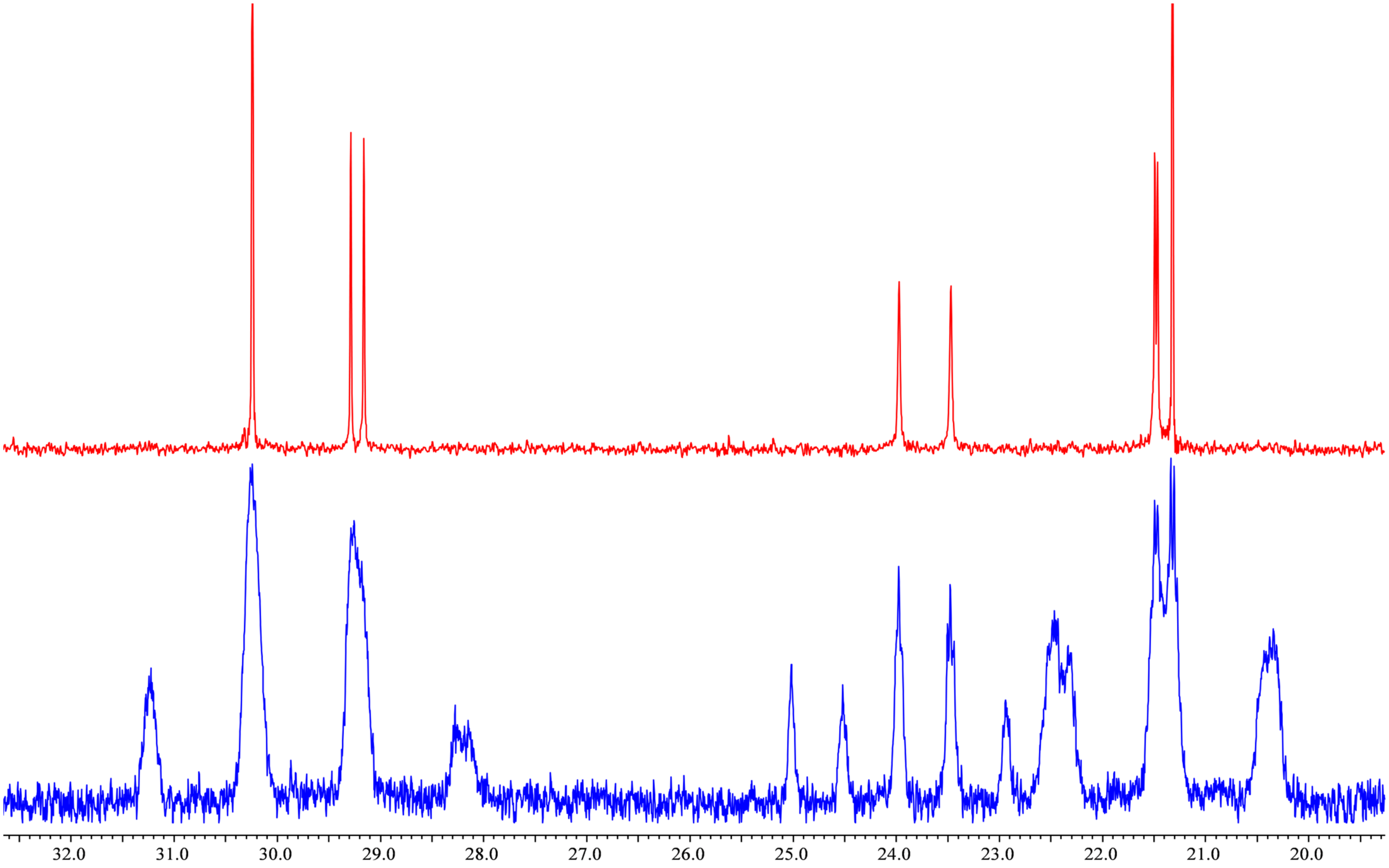

Figure 88S. The 20-32 ppm region of $^{13}C-\{^{1}H\}$ and ^{13}C NMR spectra (125.8 MHz, $CDCl_3$) of $(Et_2N)Ph_2P^{+}–C_6H_{13}\ I^{-}$ (**6a**).

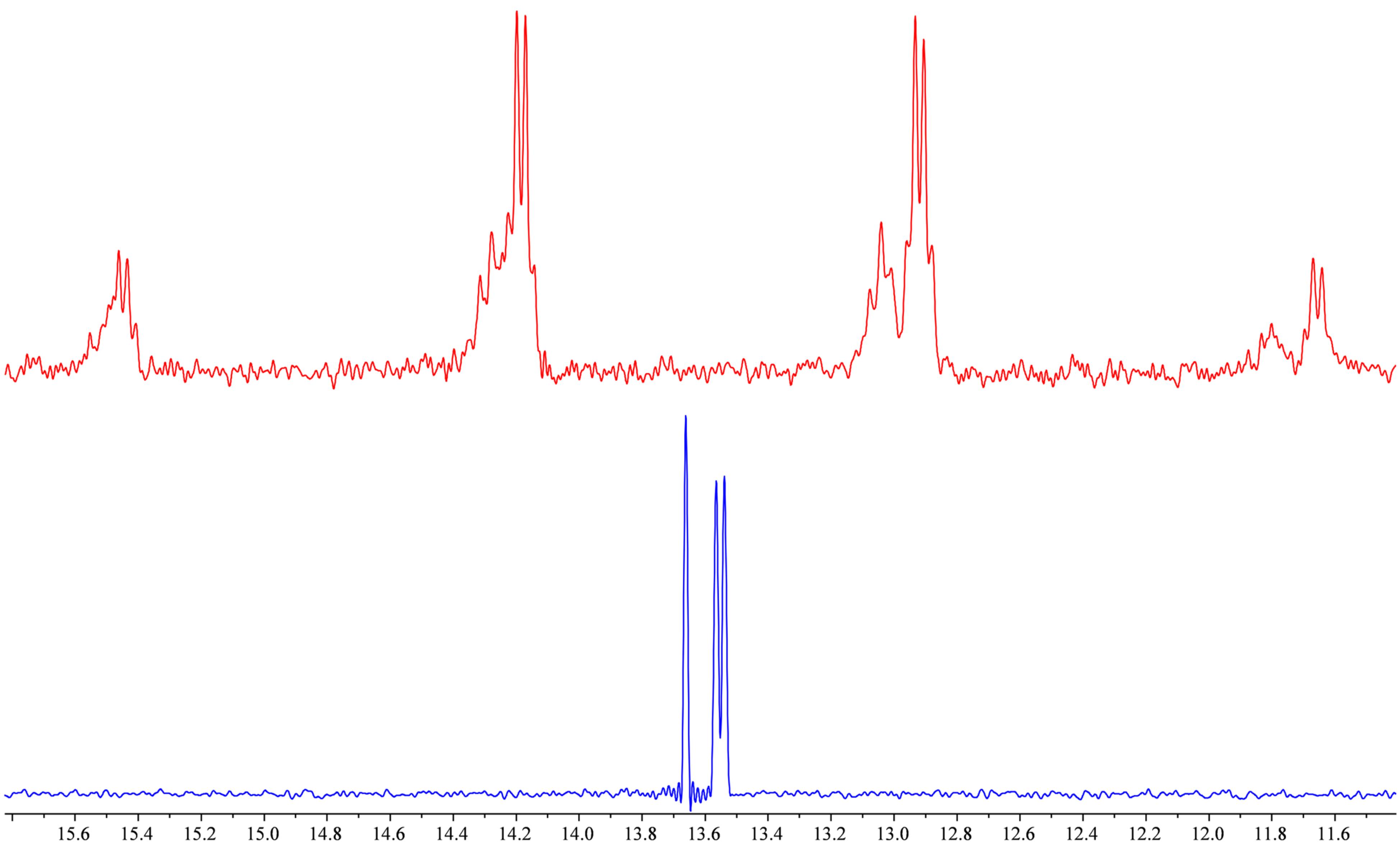

Figure 89S. The 11-16 ppm region of ^{13}C-{^{1}H} and ^{13}C NMR spectra (125.8 MHz, $CDCl_3$) of $(Et_2N)Ph_2P^+$–C_6H_{13} I^- (**6a**).

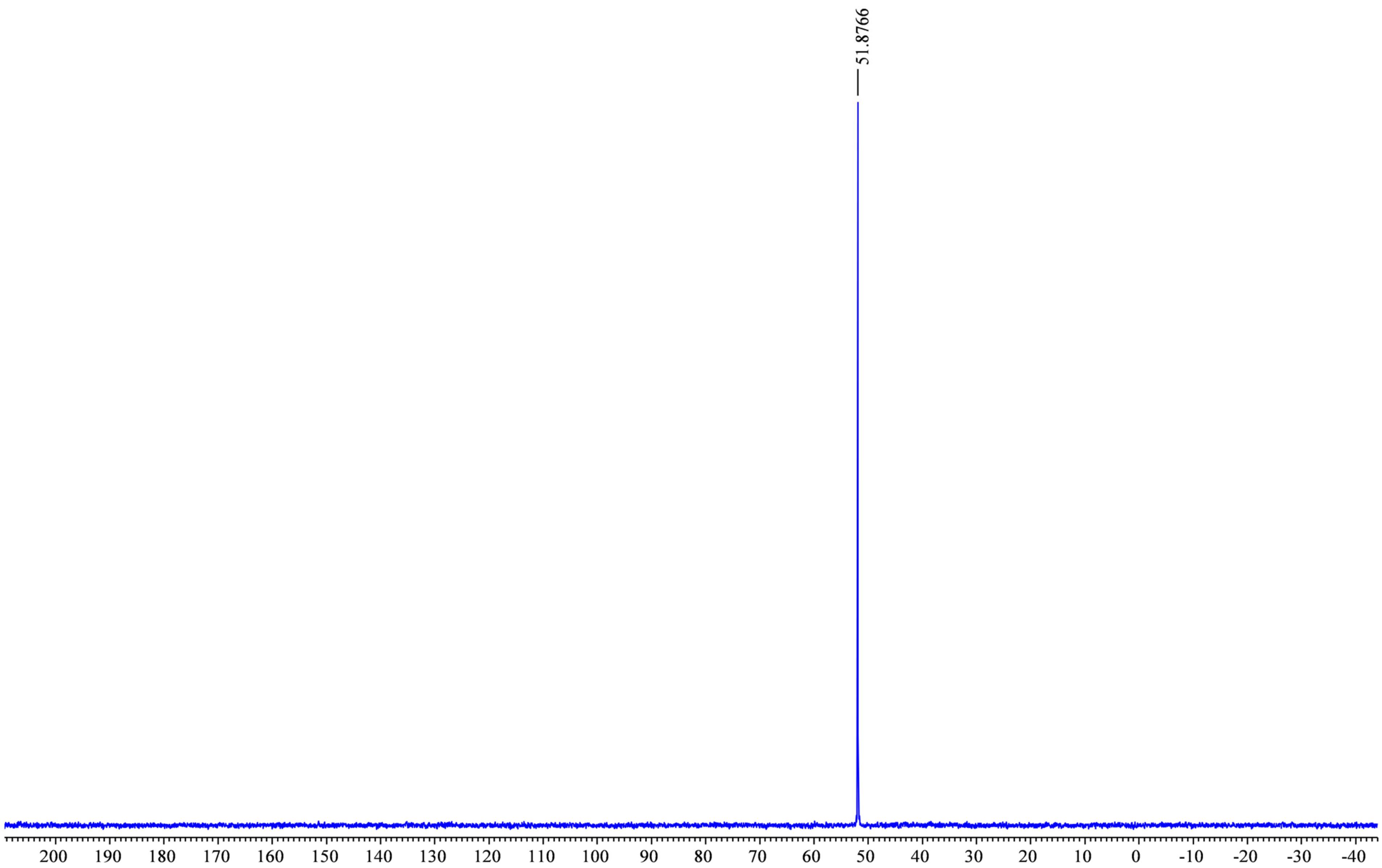

Figure 90S. ^{31}P-{^{1}H} NMR spectrum (162.0 MHz, $CDCl_3$) of $(Et_2N)Ph_2P^+–C_8H_{17}$ $I^–$ (**6b**).

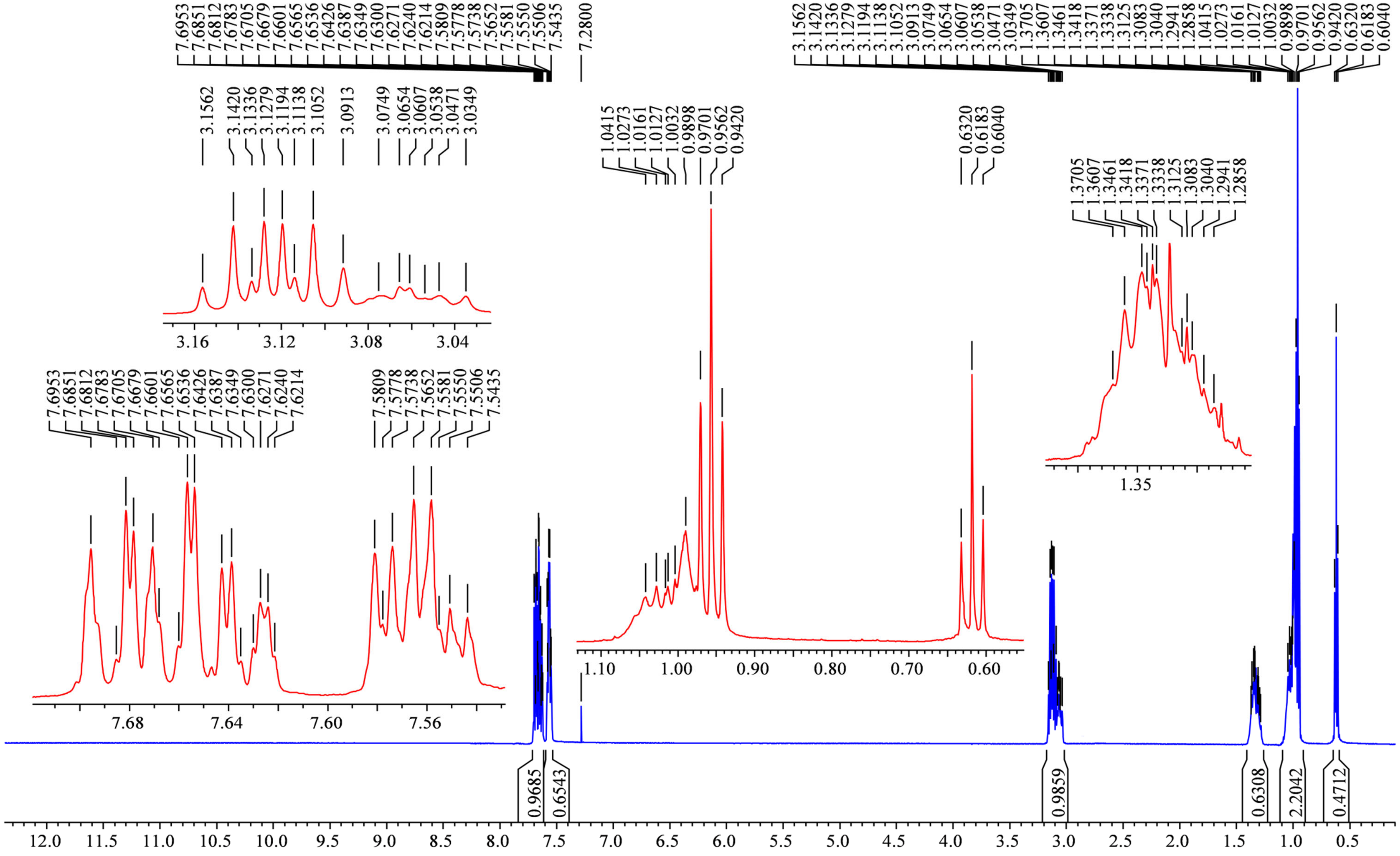

Figure 91S. ^{1}H NMR spectrum (500.0 MHz, $CDCl_3$) of $(Et_2N)Ph_2P^+–C_8H_{17}$ $I^–$ (**6b**).

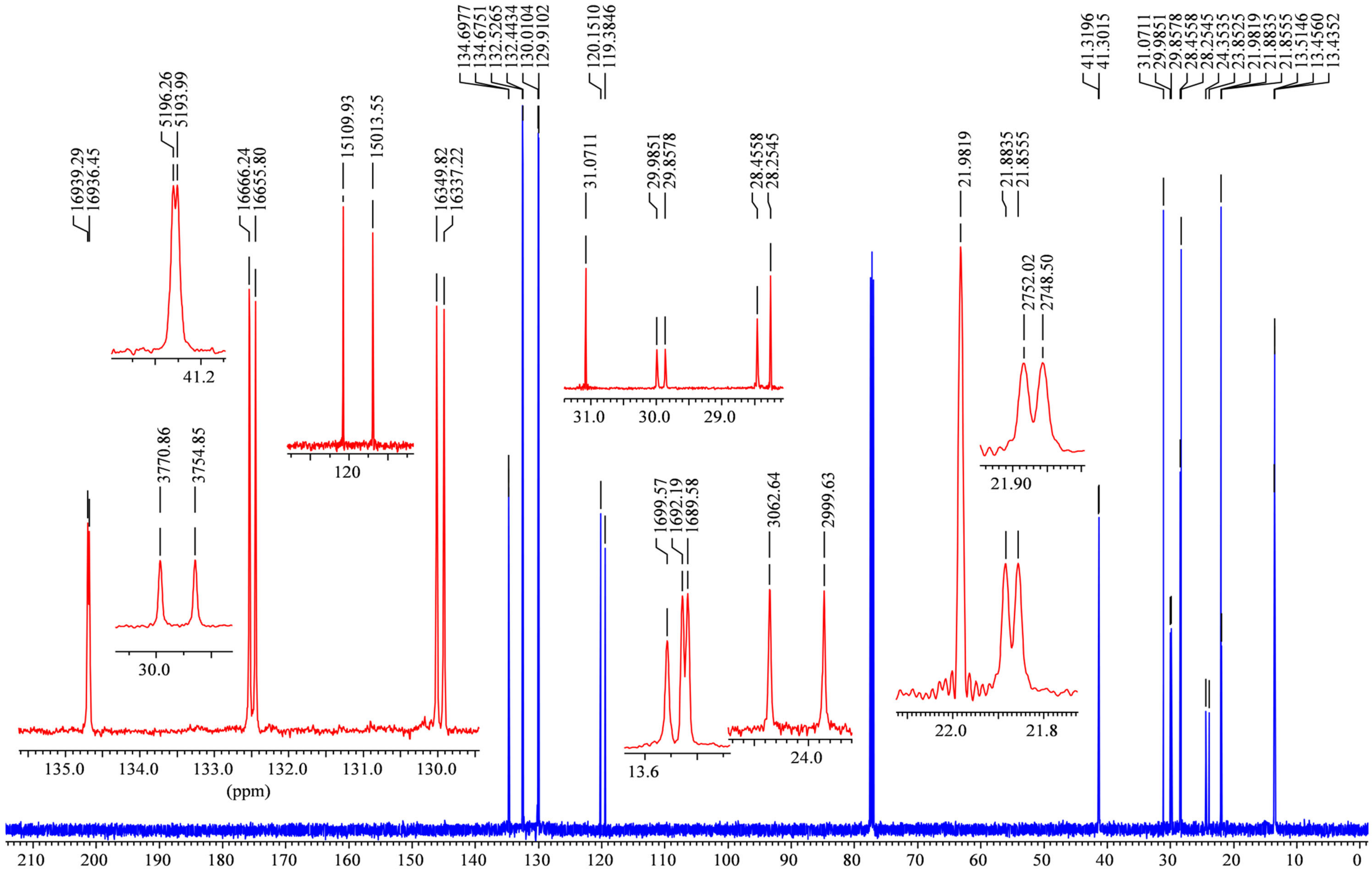

Figure 92S. $^{13}C-\{^{1}H\}$ NMR spectrum (125.8 MHz, $CDCl_3$) of $(Et_2N)Ph_2P^{+}–C_8H_{17}\ I^{-}$ (**6b**).

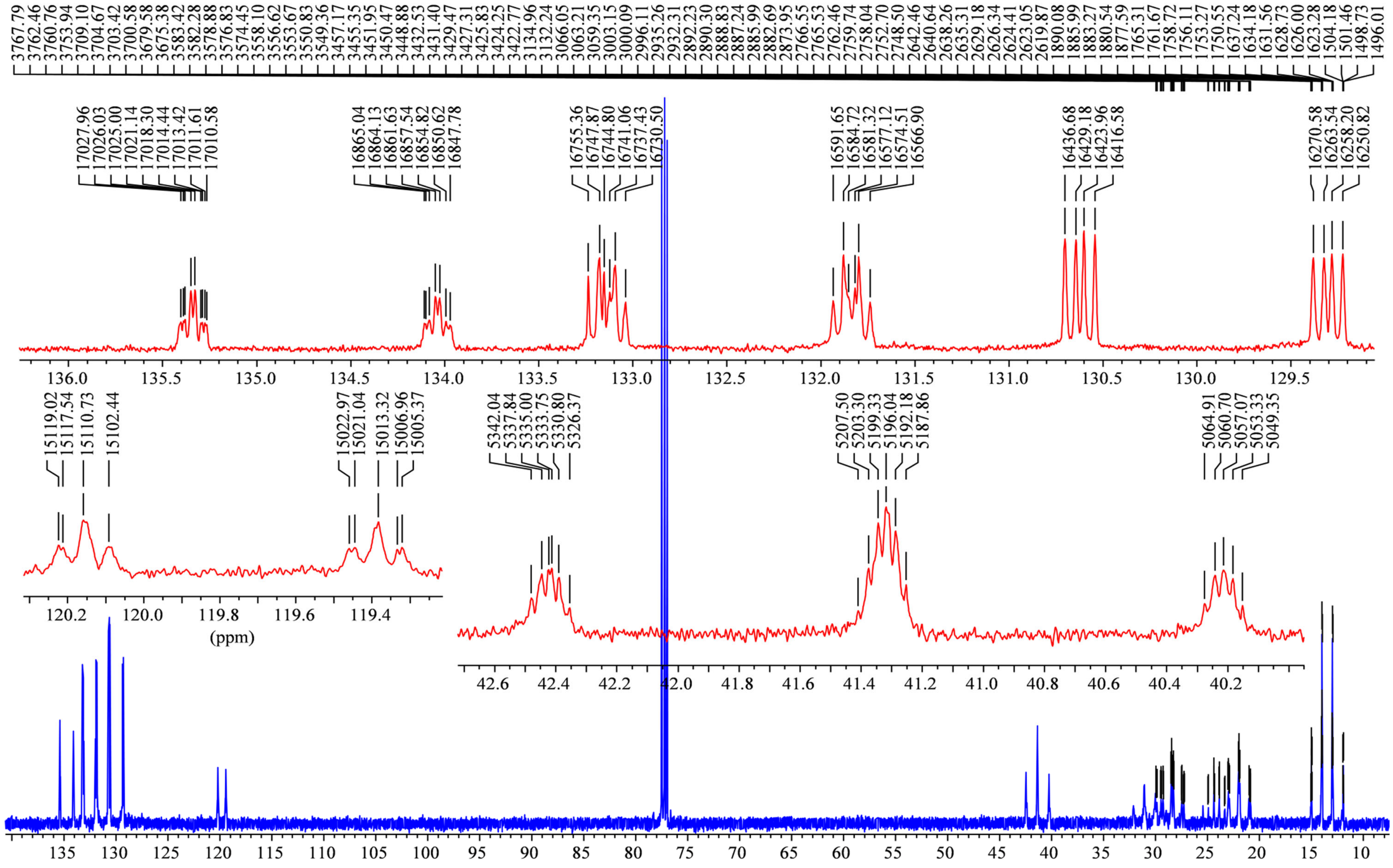

Figure 93S. ^{13}C NMR spectrum (125.8 MHz, $CDCl_3$) of $(Et_2N)Ph_2P^+–C_8H_{17}$ $I^–$ (**6b**).

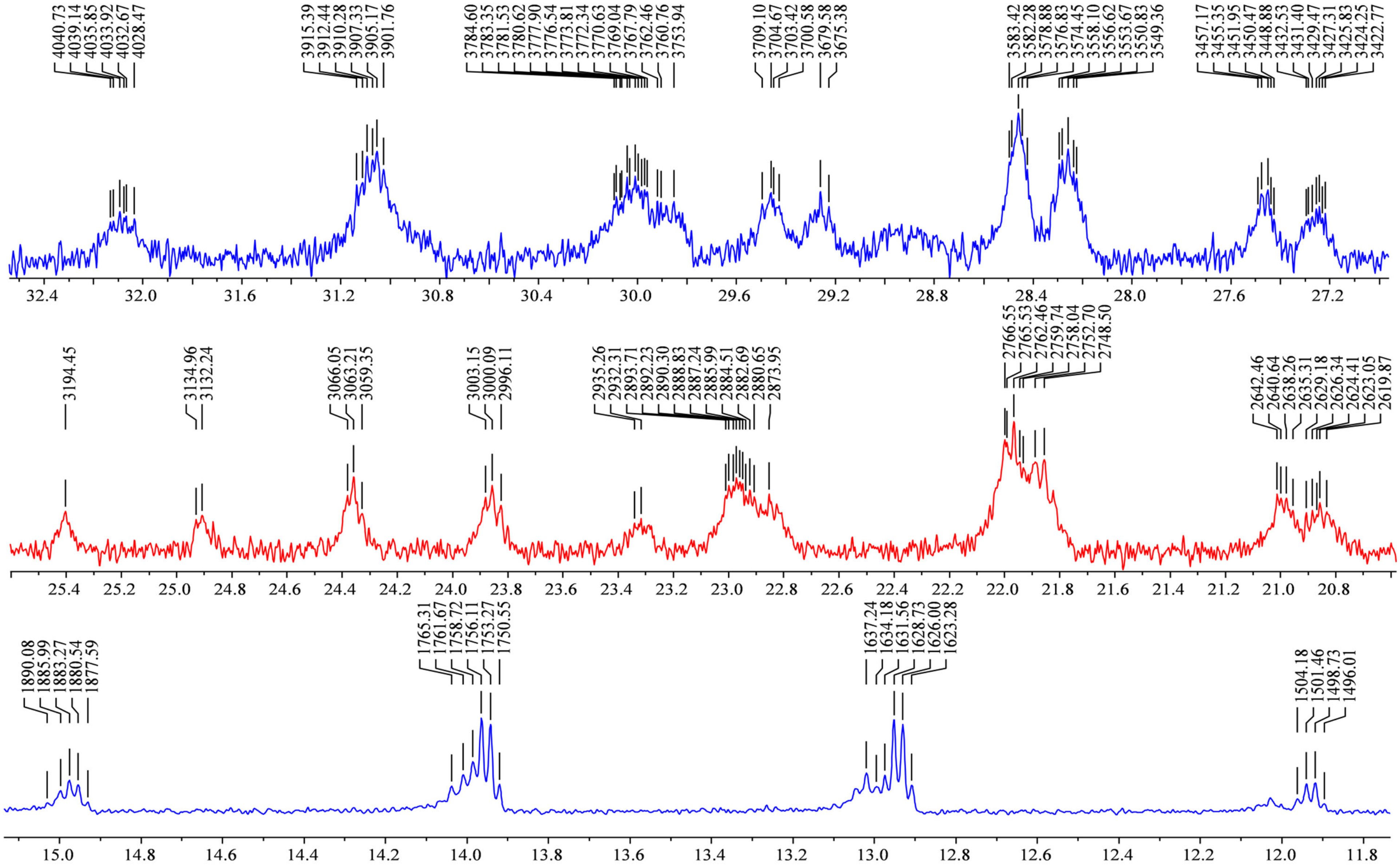

Figure 94S. High-field fragments of ^{13}C NMR spectrum (125.8 MHz, $CDCl_3$) of $(Et_2N)Ph_2P^+–C_8H_{17}$ I^- (**6b**).

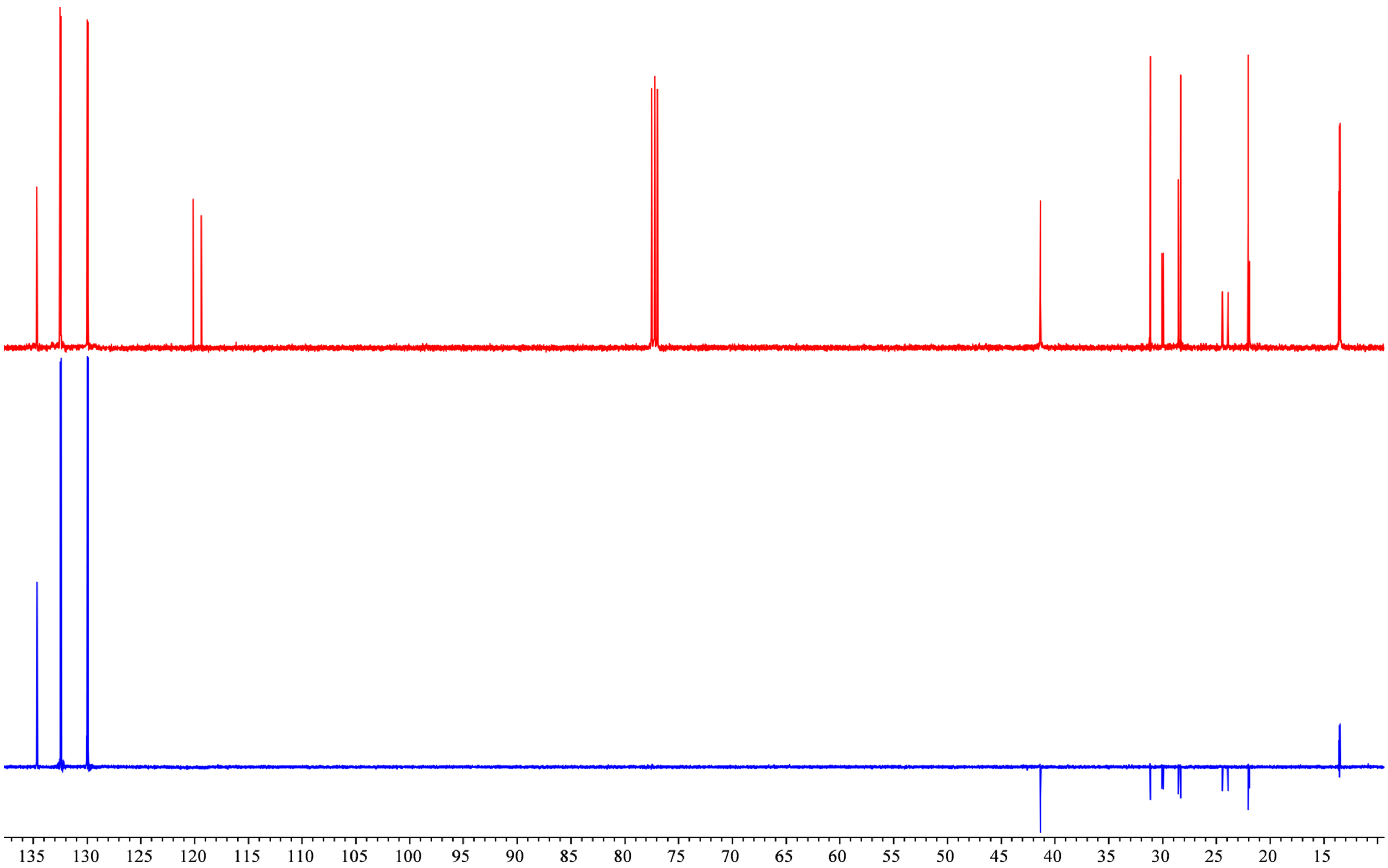

Figure 95S. ^{13}C-{^{1}H} and ^{13}C-{^{1}H}-dept NMR spectra (125.8 MHz, $CDCl_3$) of $(Et_2N)Ph_2P^{+}–C_8H_{17}\ I^{-}$ (**6b**).

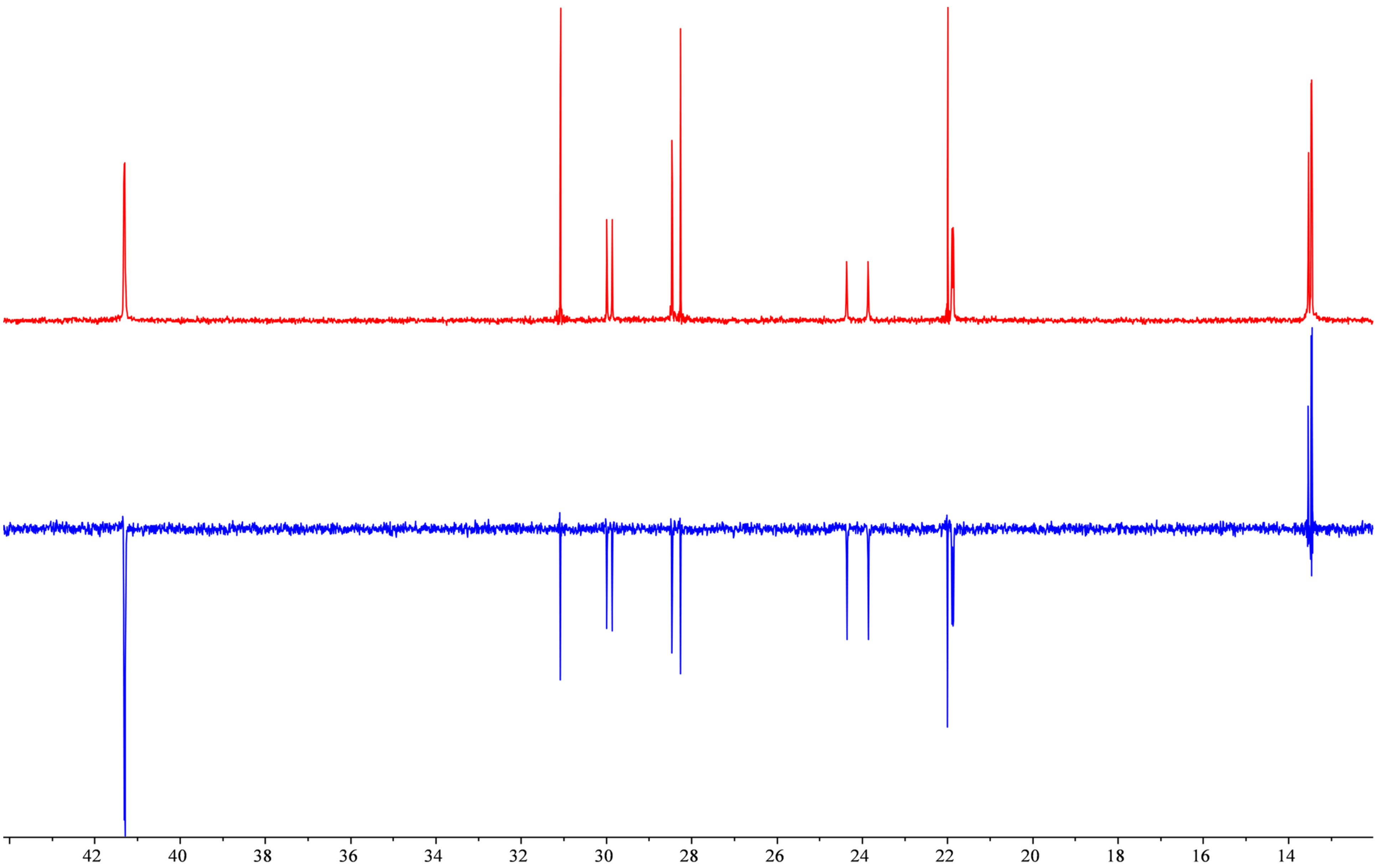

Figure 96S. High-field fragment of ^{13}C-{^{1}H} and ^{13}C-{^{1}H}-dept NMR spectra (125.8 MHz, $CDCl_3$) of $(Et_2N)Ph_2P^+–C_8H_{17}$ $I^–$ (**6b**).

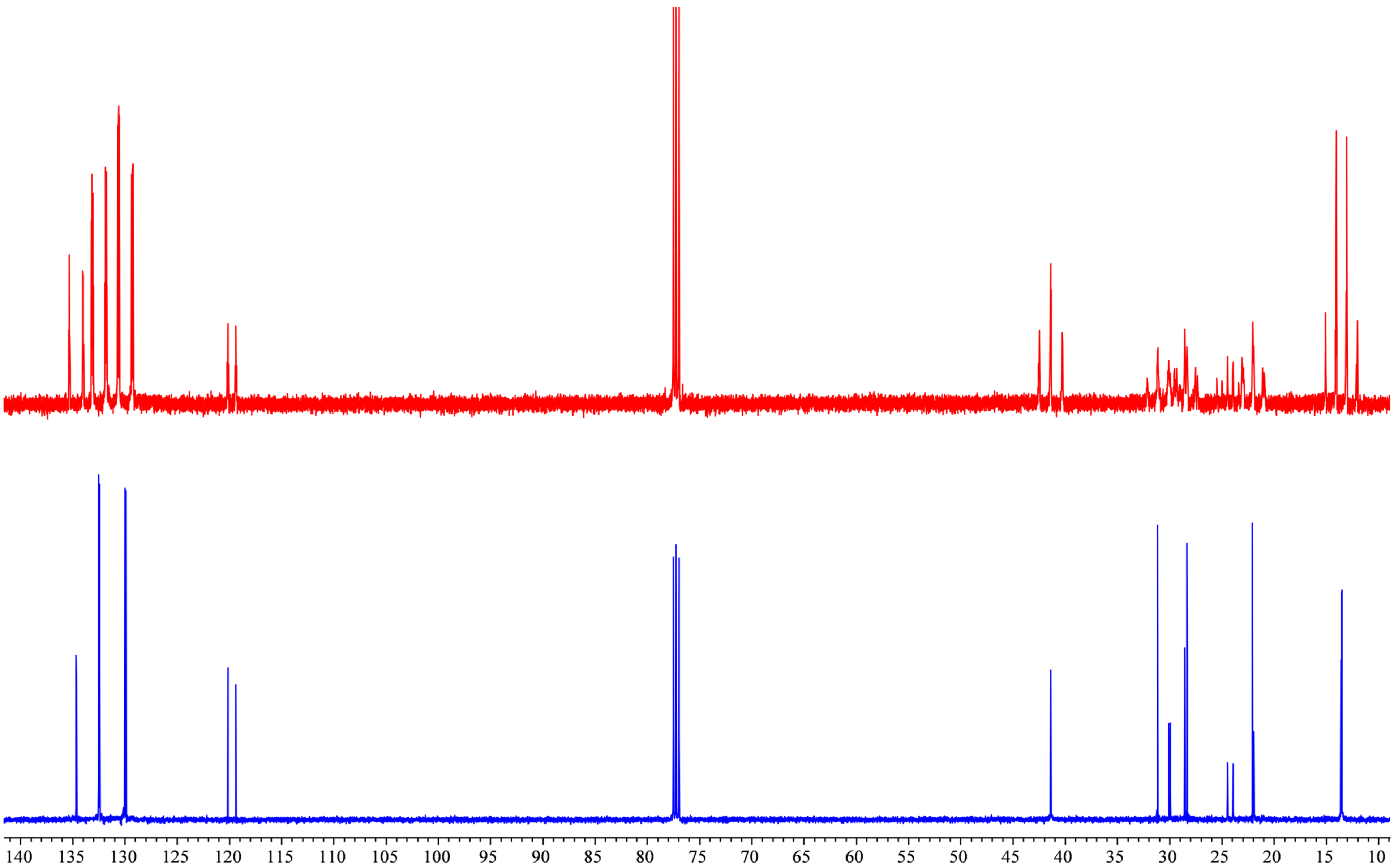

Figure 97S. $^{13}C-\{^{1}H\}$ and ^{13}C NMR spectra (125.8 MHz, $CDCl_3$) of $(Et_2N)Ph_2P^{+}–C_8H_{17}$ $I^{–}$ (**6b**).

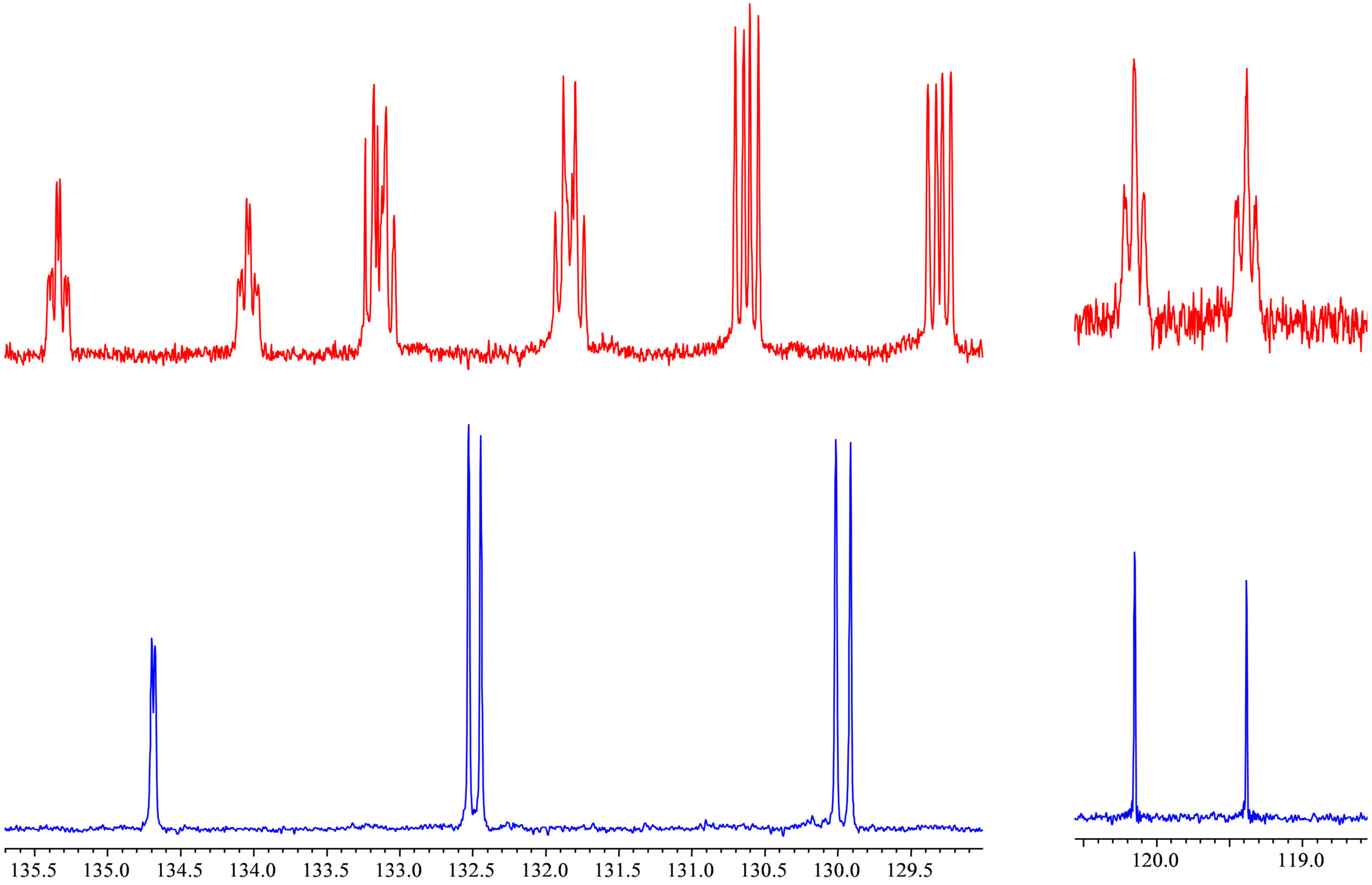

Figure 98S. Low-field fragments of ^{13}C-{^{1}H} and ^{13}C NMR spectra (125.8 MHz, $CDCl_3$) of $(Et_2N)Ph_2P^+–C_8H_{17}\ I^-$ (**6b**).

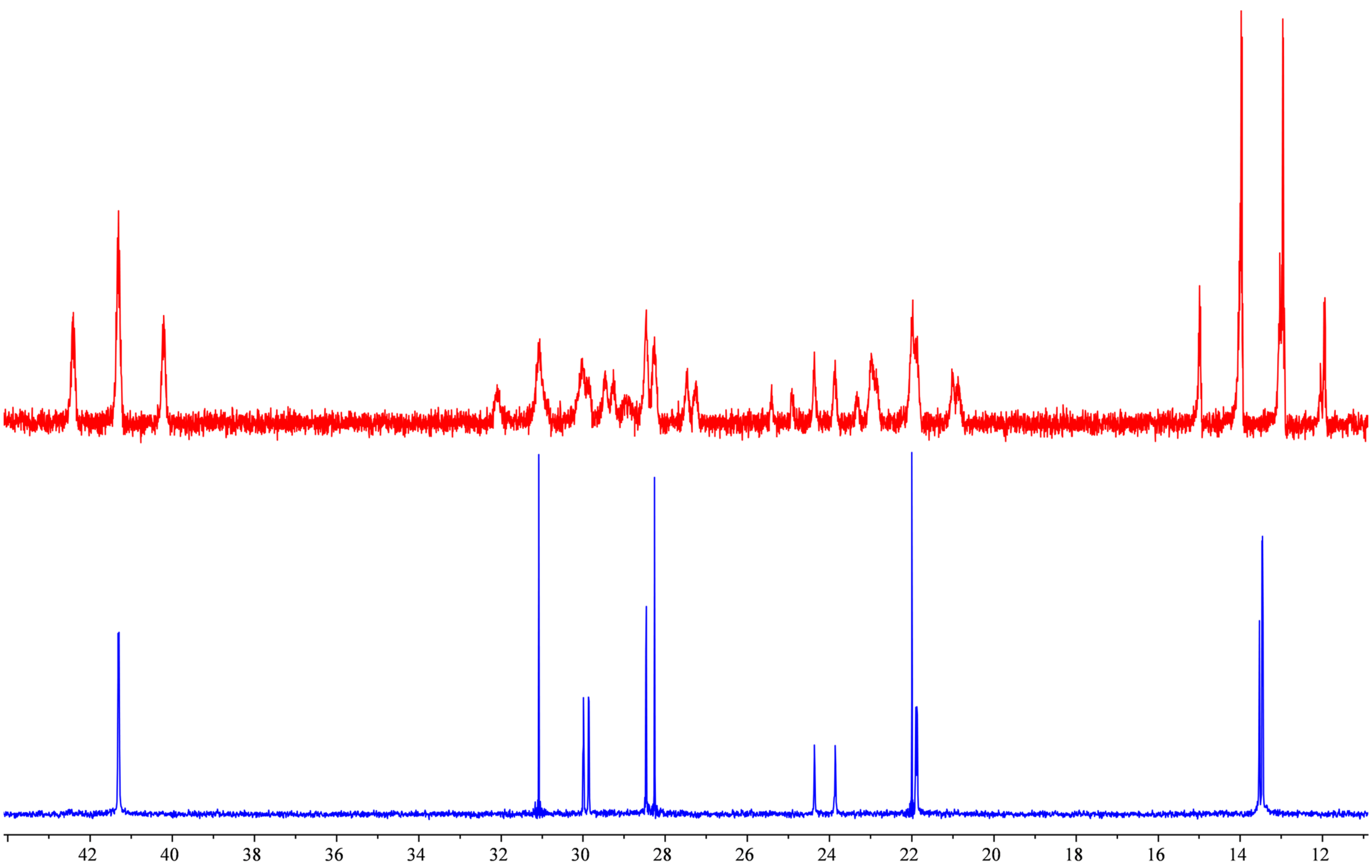

Figure 99S. High-field fragment of ^{13}C-{^{1}H} and ^{13}C NMR spectra (125.8 MHz, $CDCl_3$) of $(Et_2N)Ph_2P^{+}–C_8H_{17}$ $I^{–}$ (**6b**).

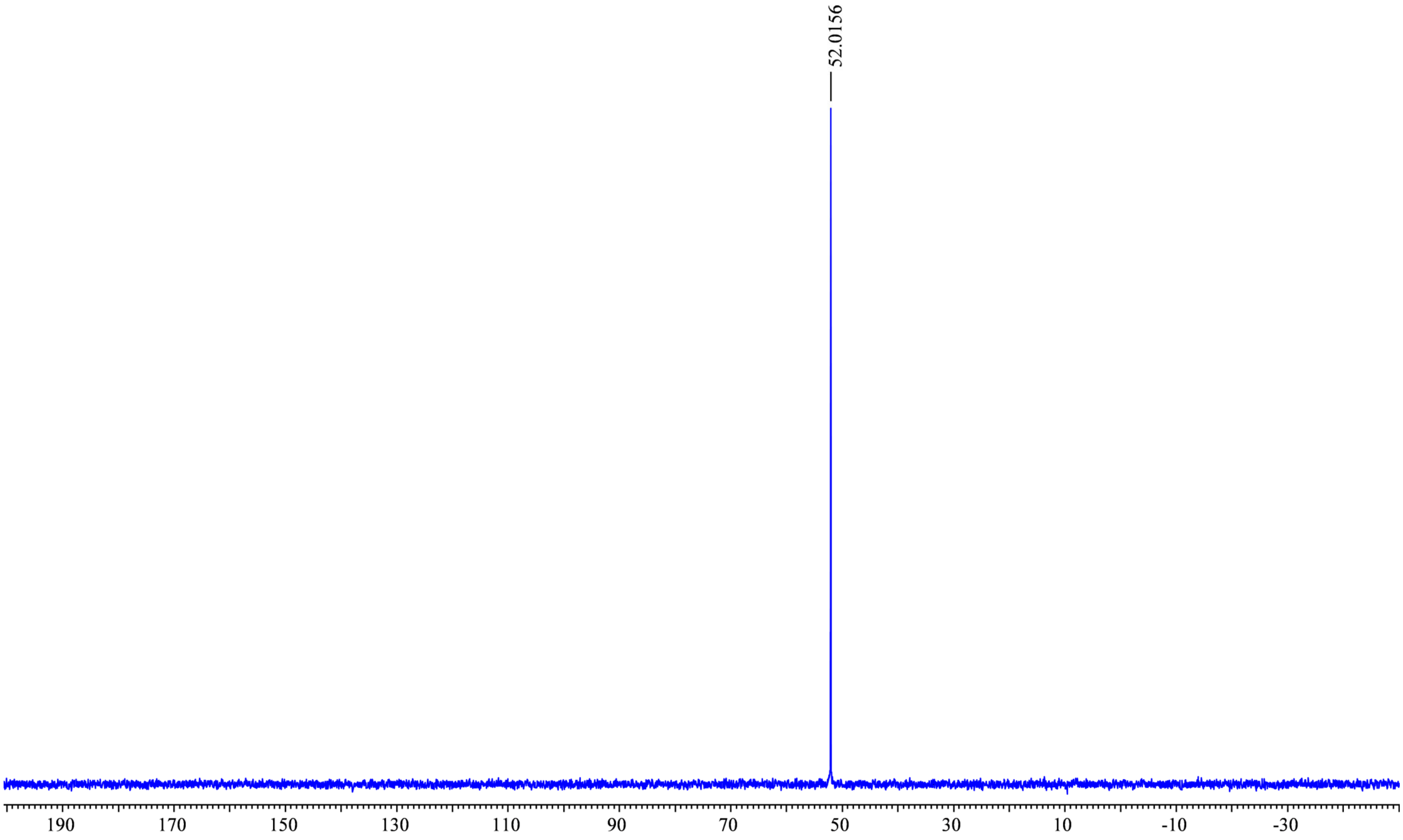

Figure 100S. ^{31}P-{^{1}H} NMR spectrum (162.0 MHz, $CDCl_3$) of $(Et_2N)Ph_2P^{+}$–C_9H_{19} I^{-} (**6c**).

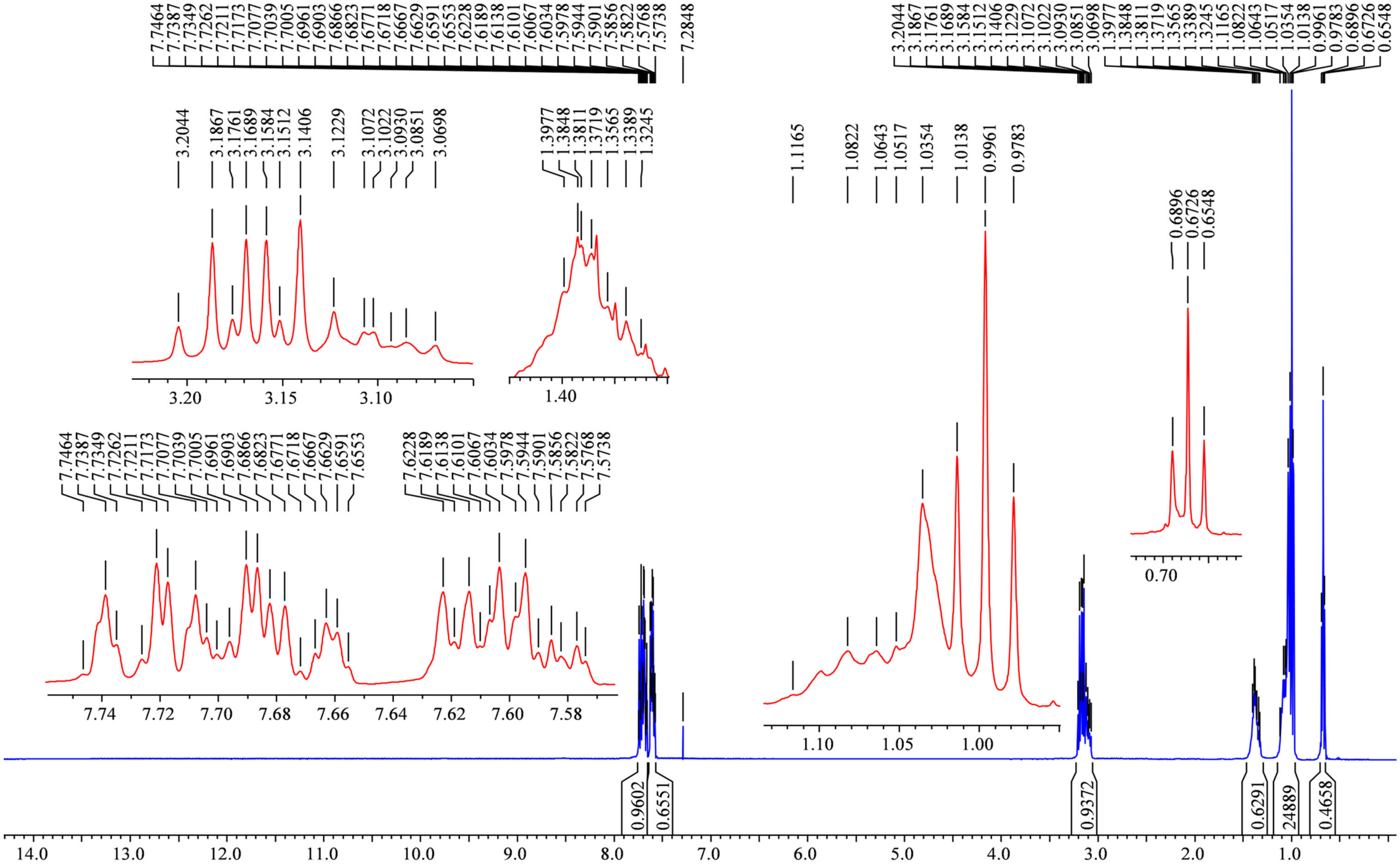

Figure 101S. ^{1}H NMR spectrum (400.0 MHz, $CDCl_3$) of $(Et_2N)Ph_2P^+–C_9H_{19}$ $I^–$ (**6c**).

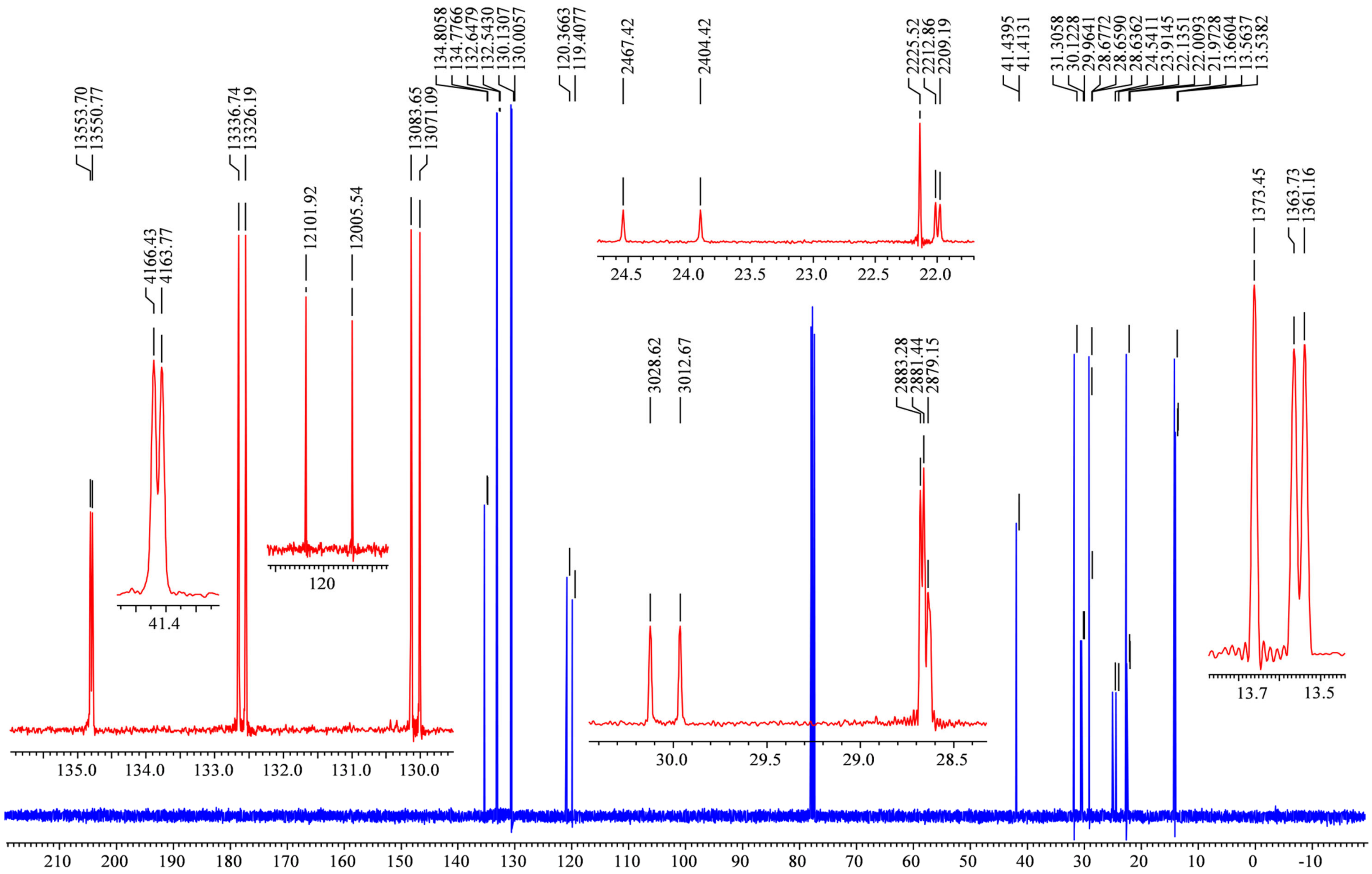

Figure 102S. $^{13}C-\{^{1}H\}$ NMR spectrum (100.6 MHz, $CDCl_3$) of $(Et_2N)Ph_2P^{+}–C_9H_{19}$ I^{-} (**6c**).

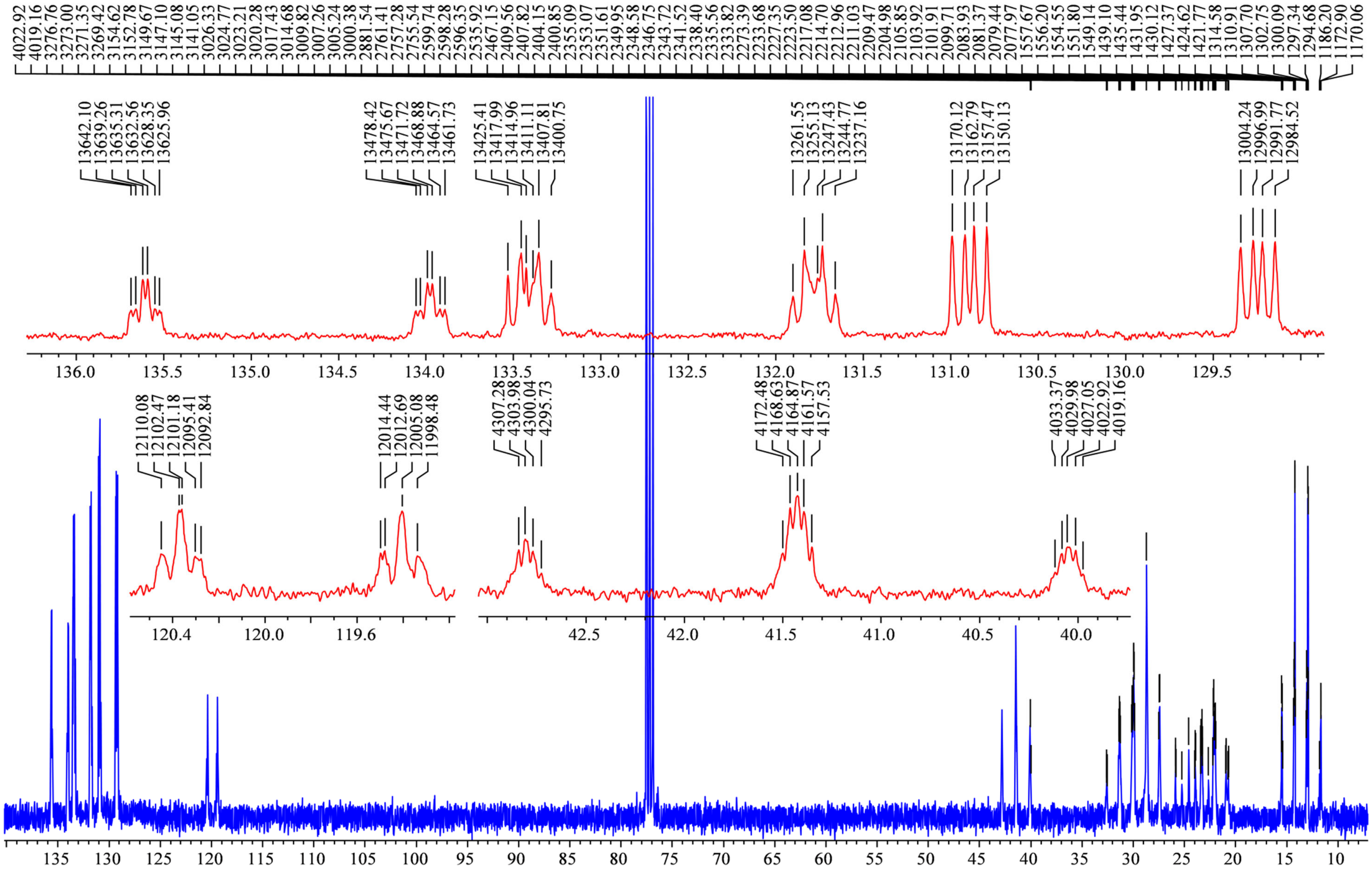

Figure 103S. ^{13}C NMR spectrum (100.6 MHz, $CDCl_3$) of $(Et_2N)Ph_2P^+–C_9H_{19}$ $I^–$ (**6c**).

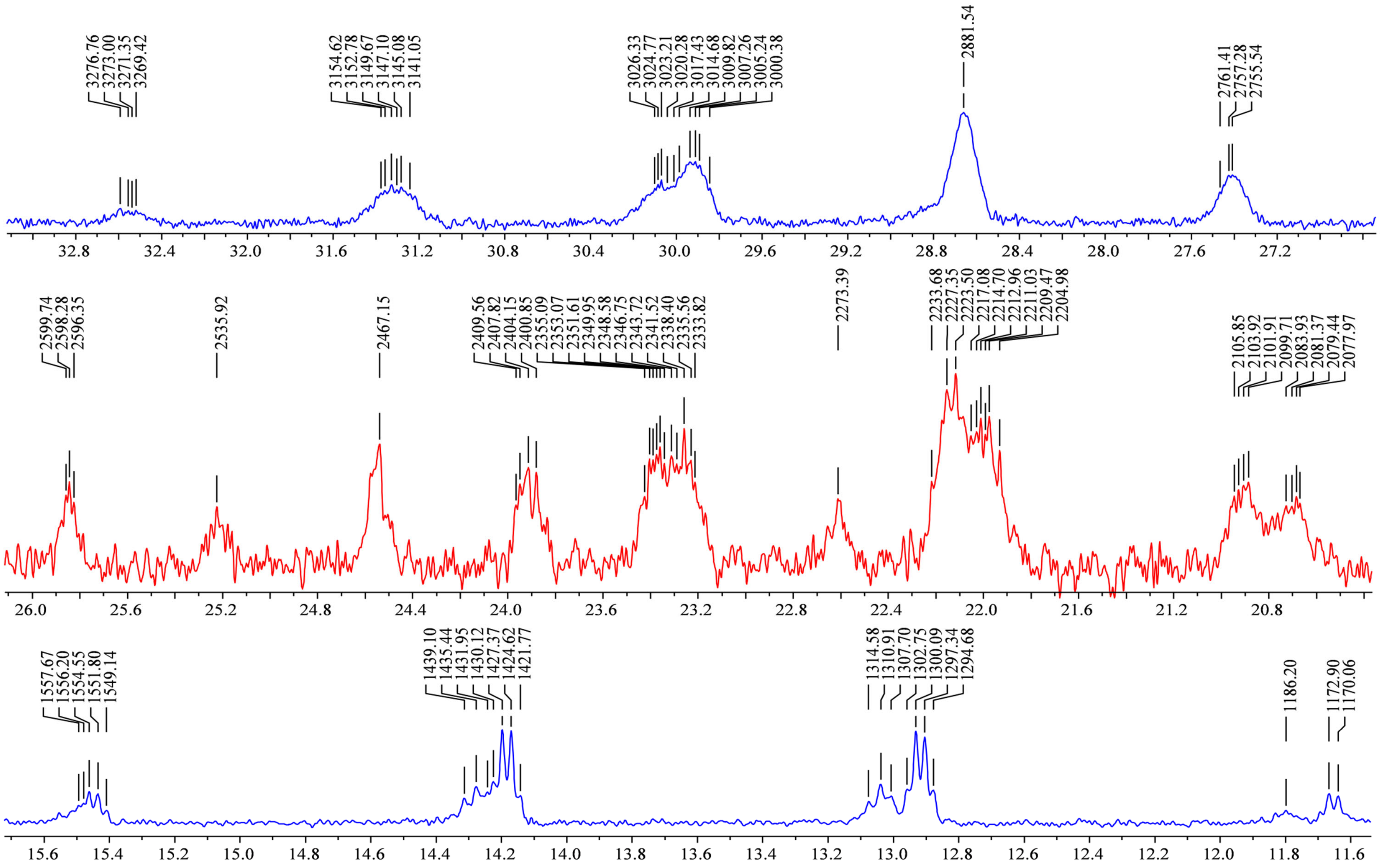

Figure 104S. High-field fragments of ^{13}C NMR spectrum (100.6 MHz, $CDCl_3$) of $(Et_2N)Ph_2P^+–C_9H_{19}$ $I^–$ (**6c**).

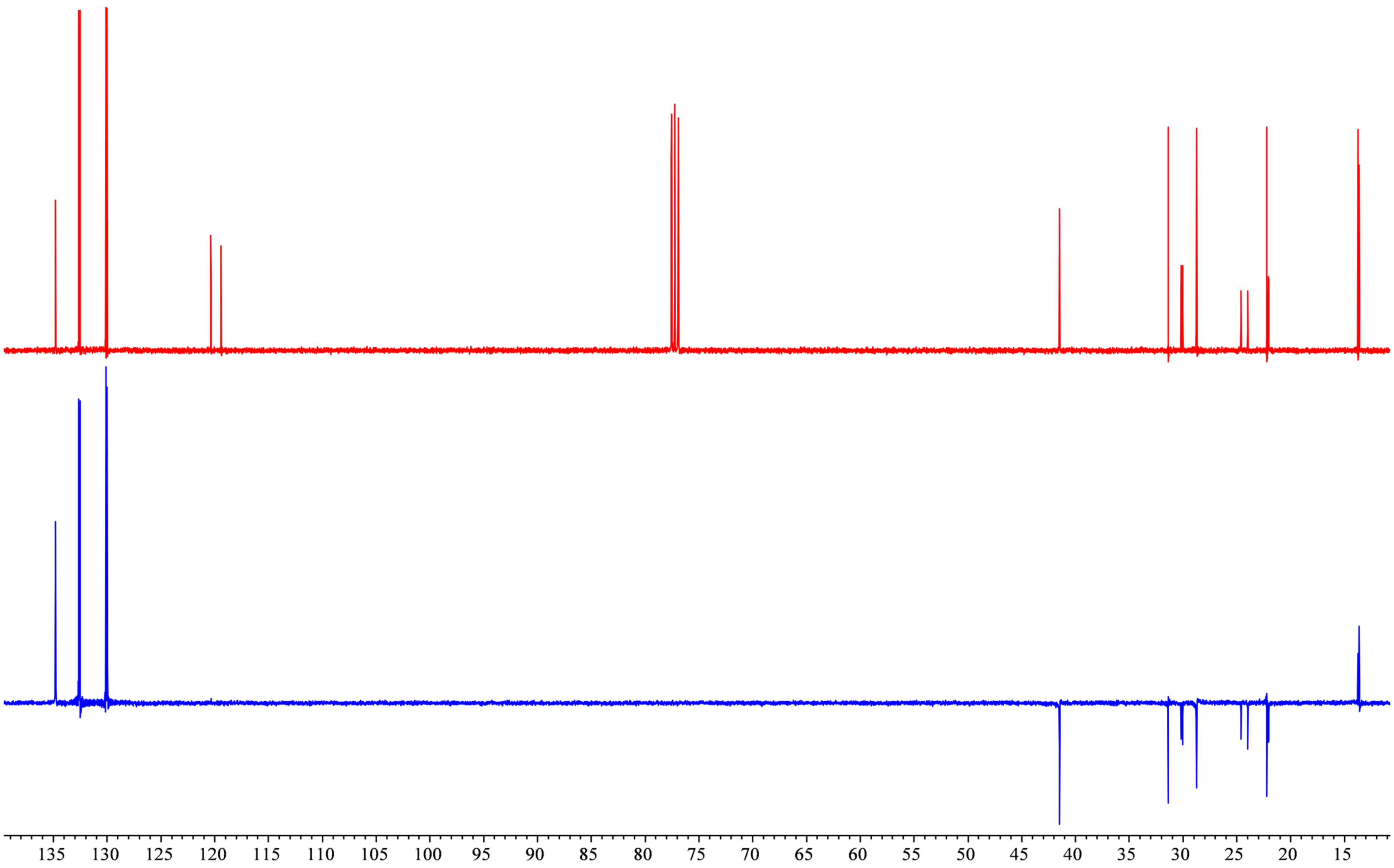

Figure 105S. ^{13}C-{^{1}H} and ^{13}C-{^{1}H}-dept NMR spectra (100.6 MHz, $CDCl_3$) of $(Et_2N)Ph_2P^+–C_9H_{19}\ I^-$ (**6c**).

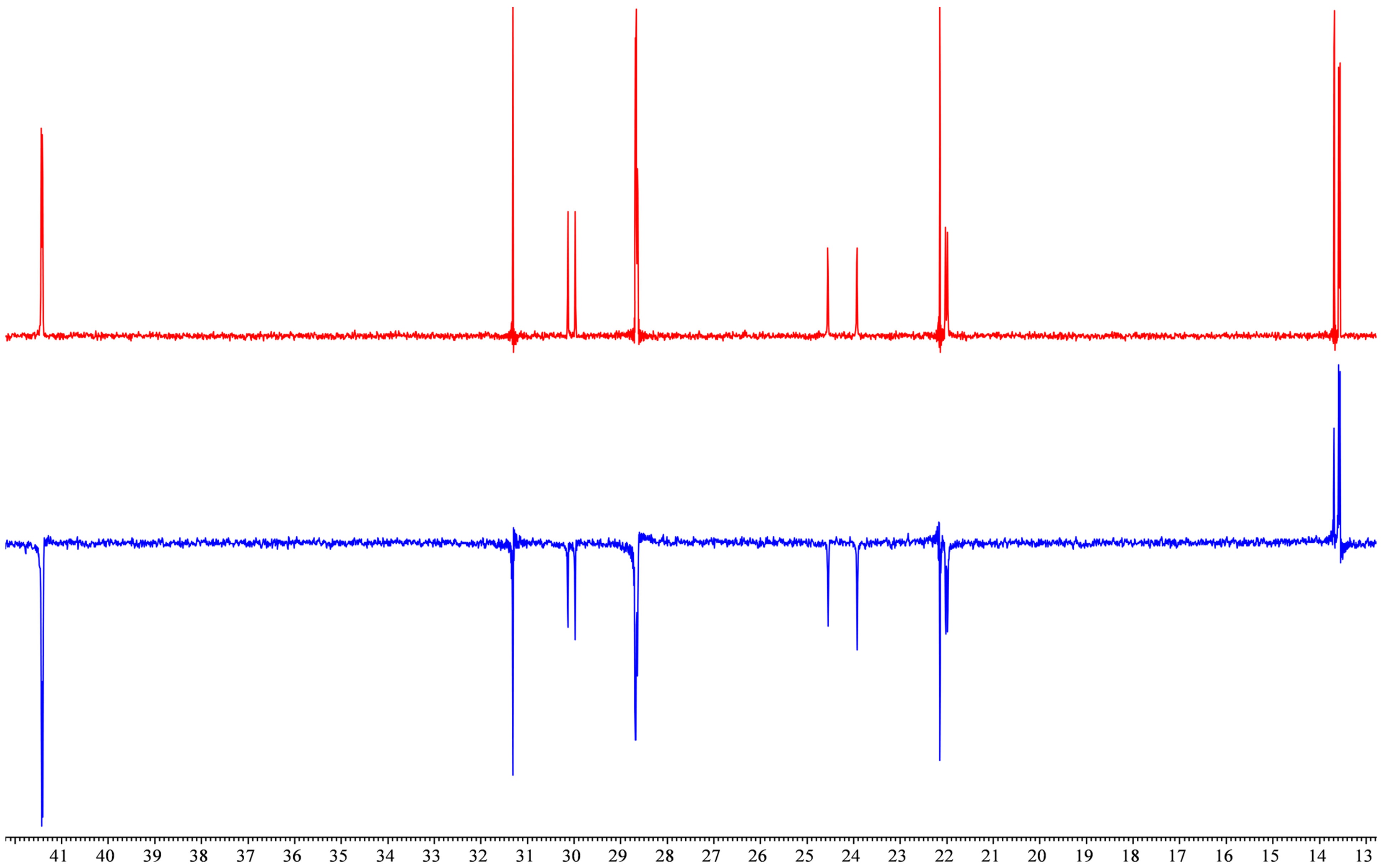

Figure 106S. High-field fragment of ^{13}C-{^{1}H} and ^{13}C-{^{1}H}-dept NMR spectra (100.6 MHz, $CDCl_3$) of $(Et_2N)Ph_2P^{+}–C_9H_{19}$ $I^{–}$ (**6c**).

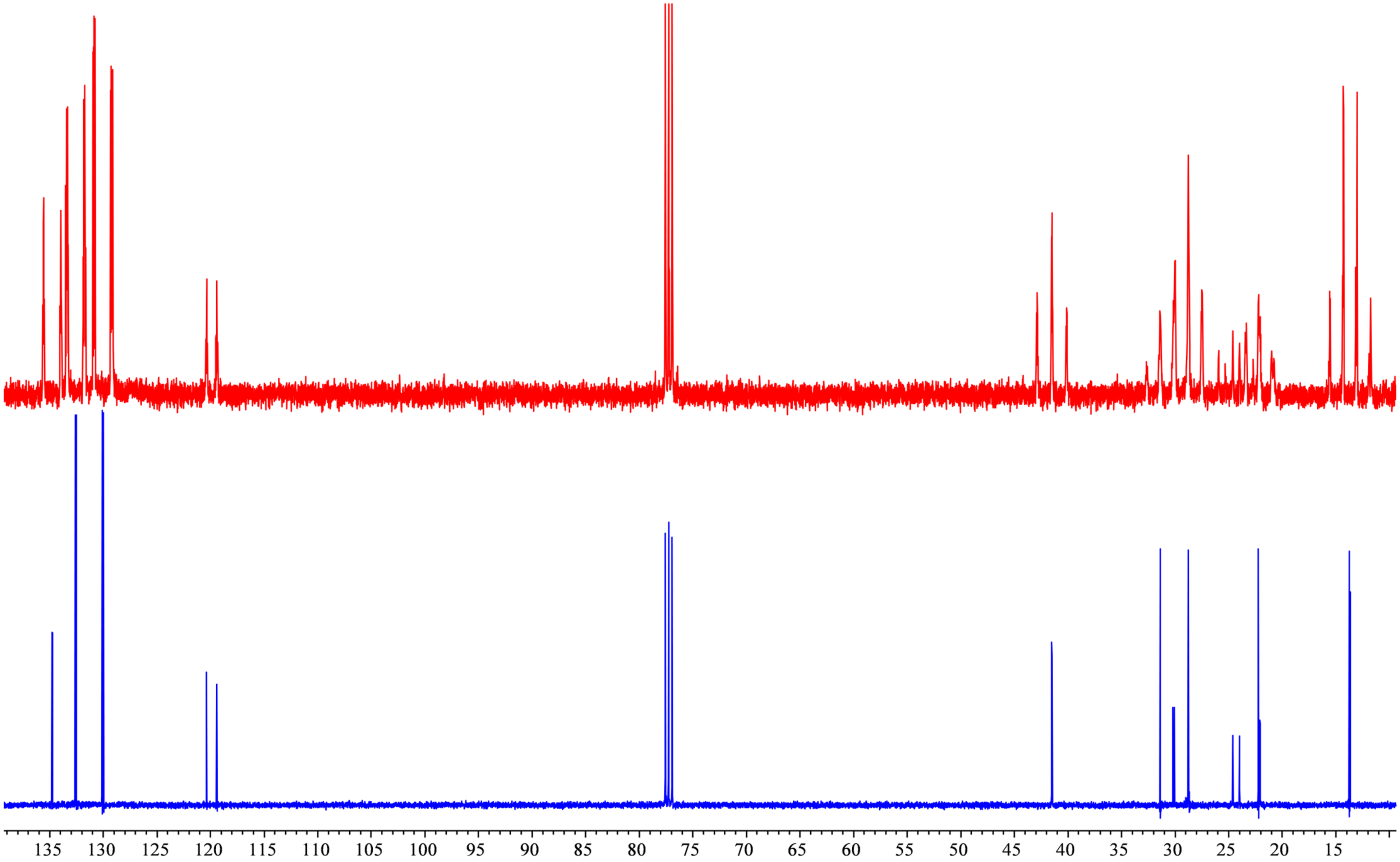

Figure 107S. ^{13}C-{^{1}H} and ^{13}C NMR spectra (100.6 MHz, $CDCl_3$) of $(Et_2N)Ph_2P^{+}–C_9H_{19}\ I^{-}$ (**6c**).

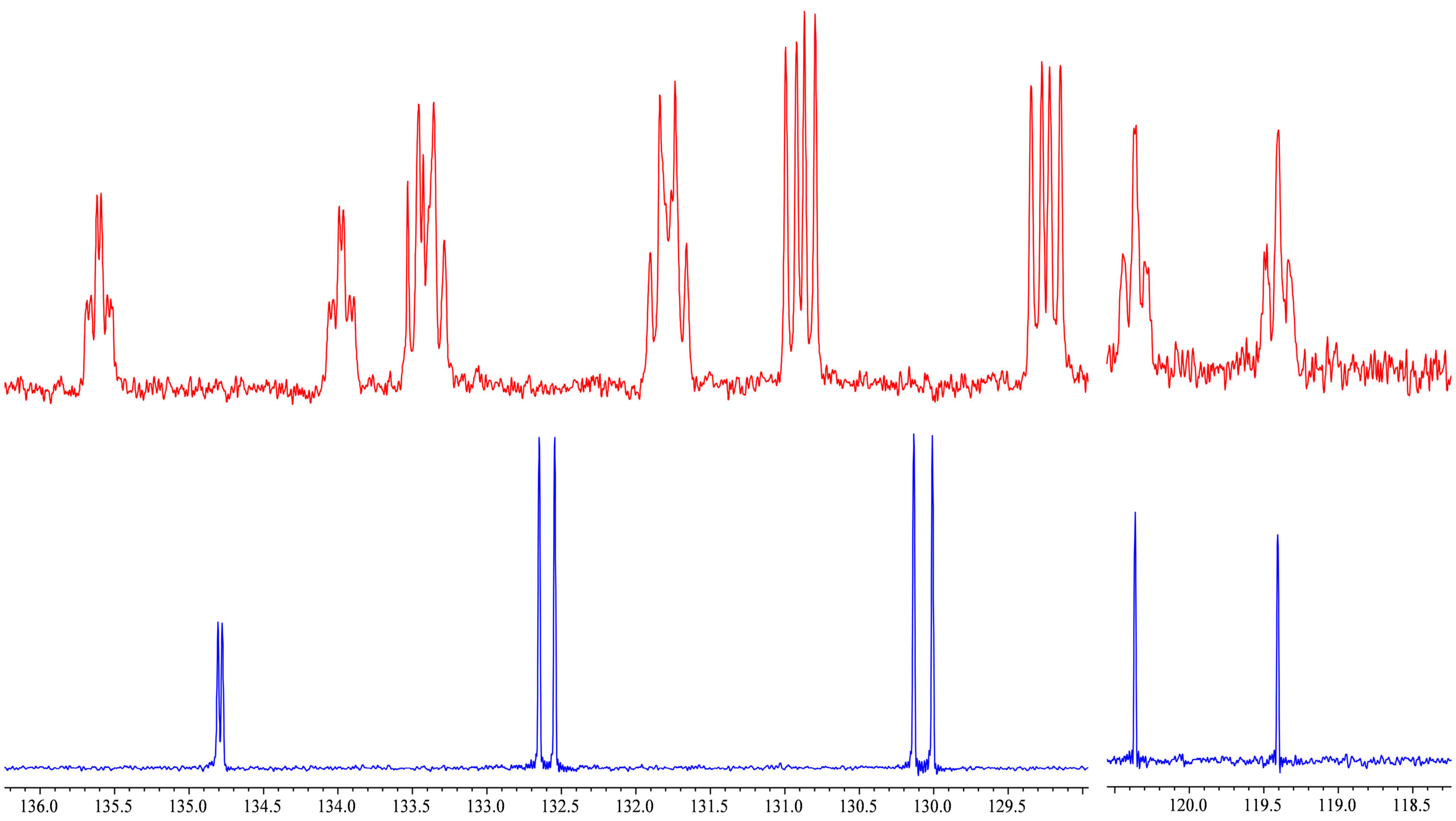

Figure 108S. Low-field fragments of ^{13}C-{^{1}H} and ^{13}C NMR spectra (100.6 MHz, $CDCl_3$) of $(Et_2N)Ph_2P^{+}–C_9H_{19}$ $I^{–}$ (**6c**).

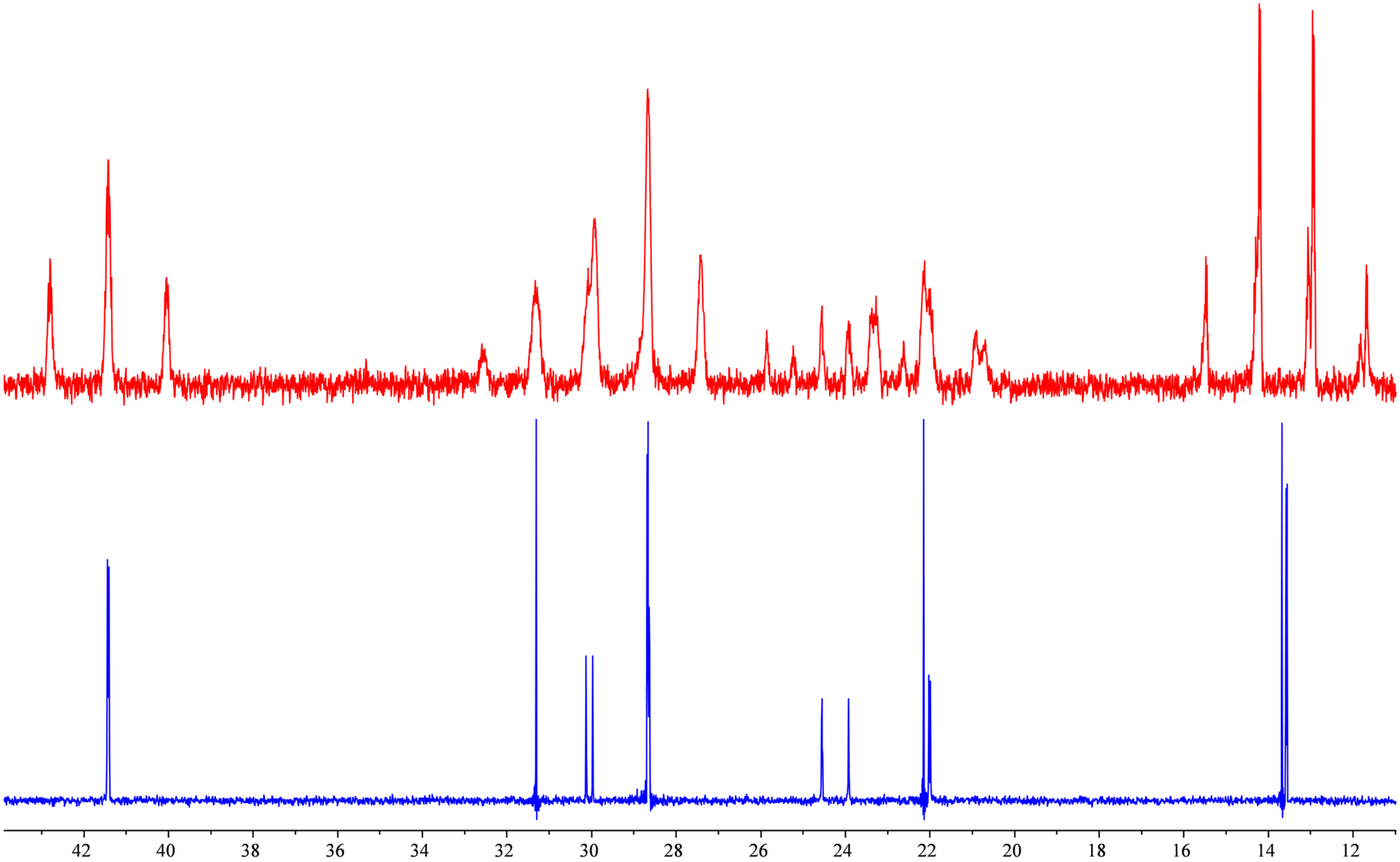

Figure 109S. High-field fragment of ^{13}C-{^{1}H} and ^{13}C NMR spectra (100.6 MHz, $CDCl_3$) of $(Et_2N)Ph_2P^{+}$–C_9H_{19} I^{-} (**6c**).

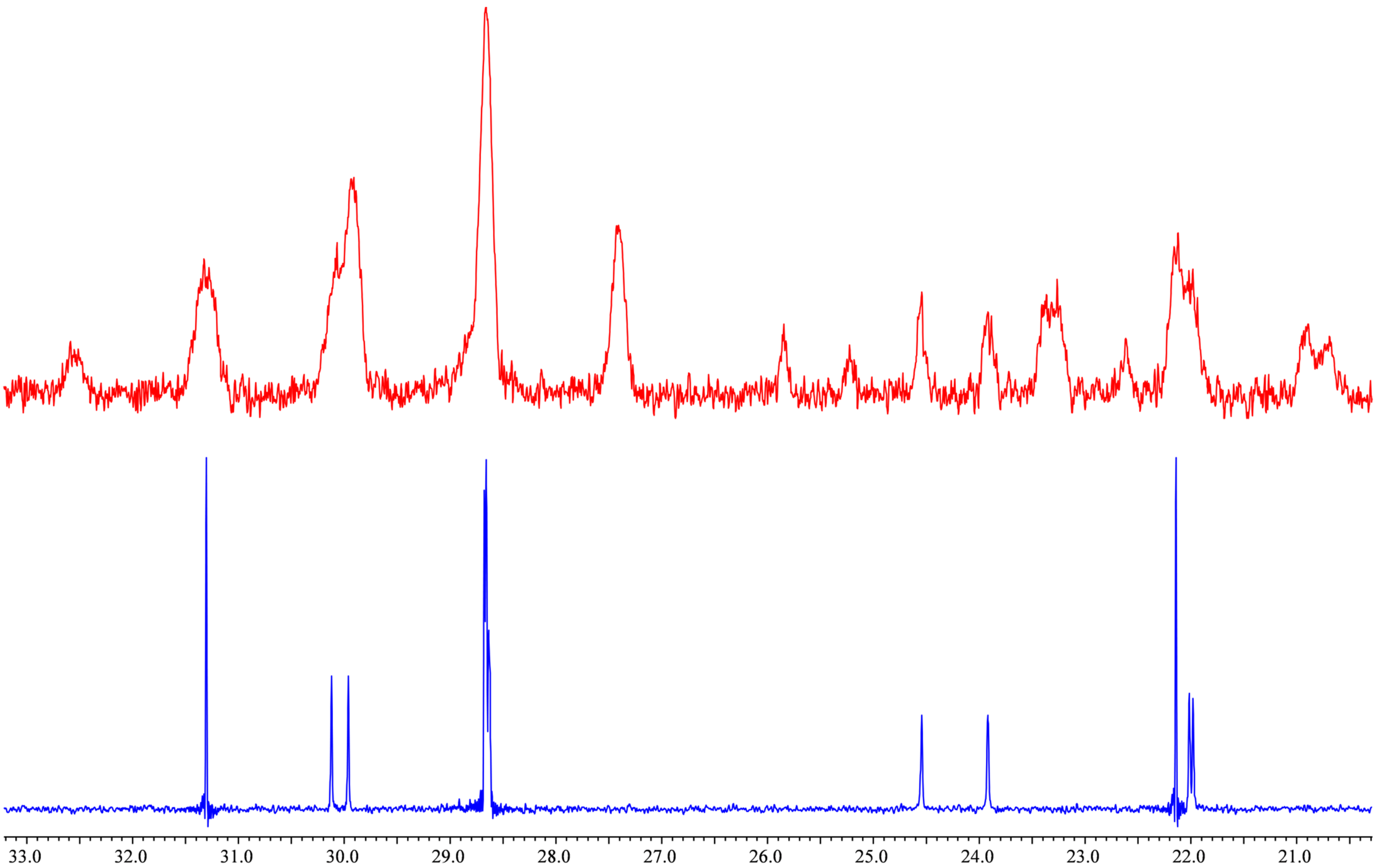

Figure 110S. The 21-33 ppm region of ^{13}C-{^{1}H} and ^{13}C NMR spectra (100.6 MHz, $CDCl_3$) of $(Et_2N)Ph_2P^+–C_9H_{19}$ $I^−$ (**6c**).

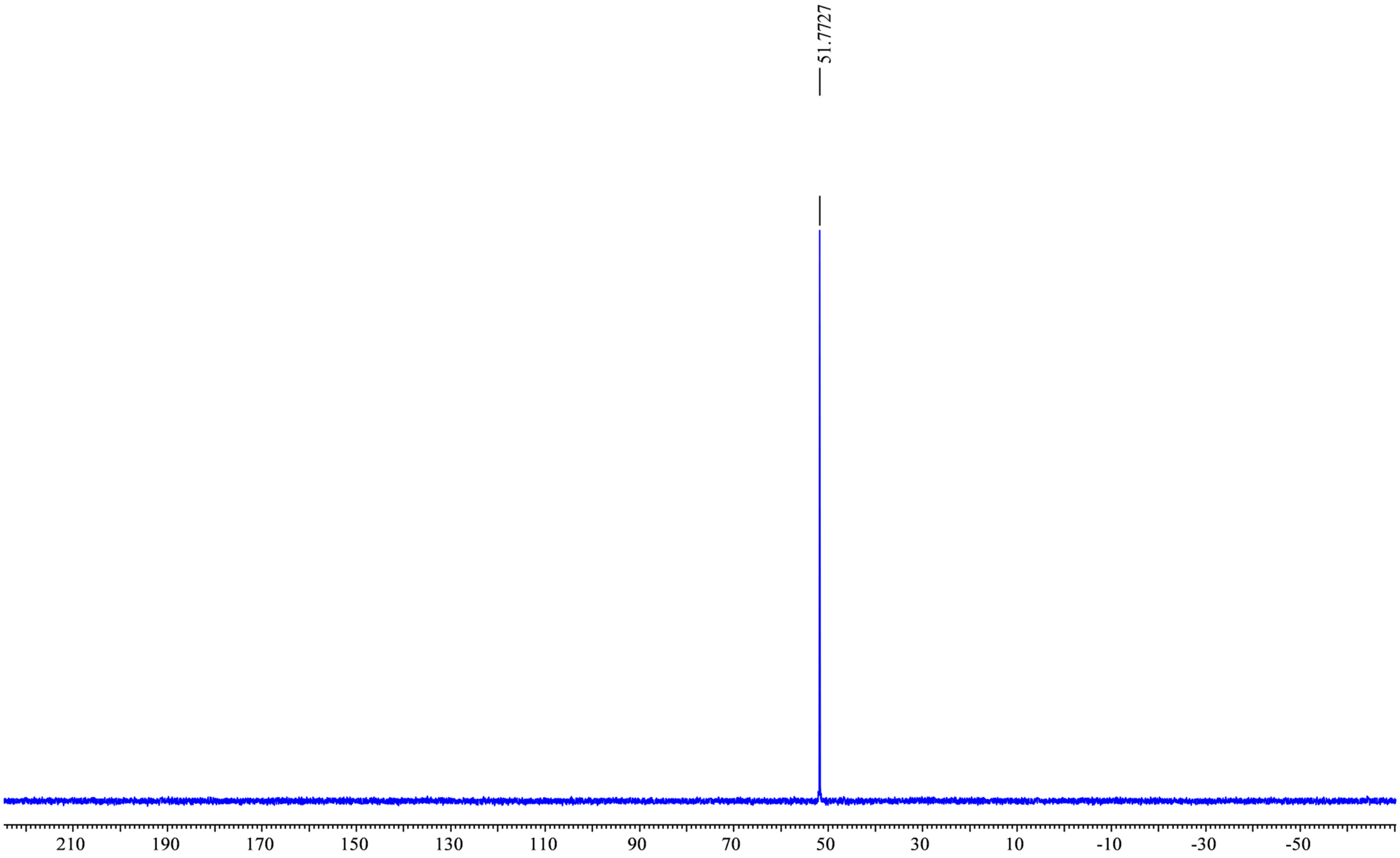

Figure 111S. ^{31}P-{^{1}H} NMR spectrum (162.0 MHz, $CDCl_3$) of $(Et_2N)Ph_2P^+–C_{10}H_{21}$ $I^−$ (**6d**).

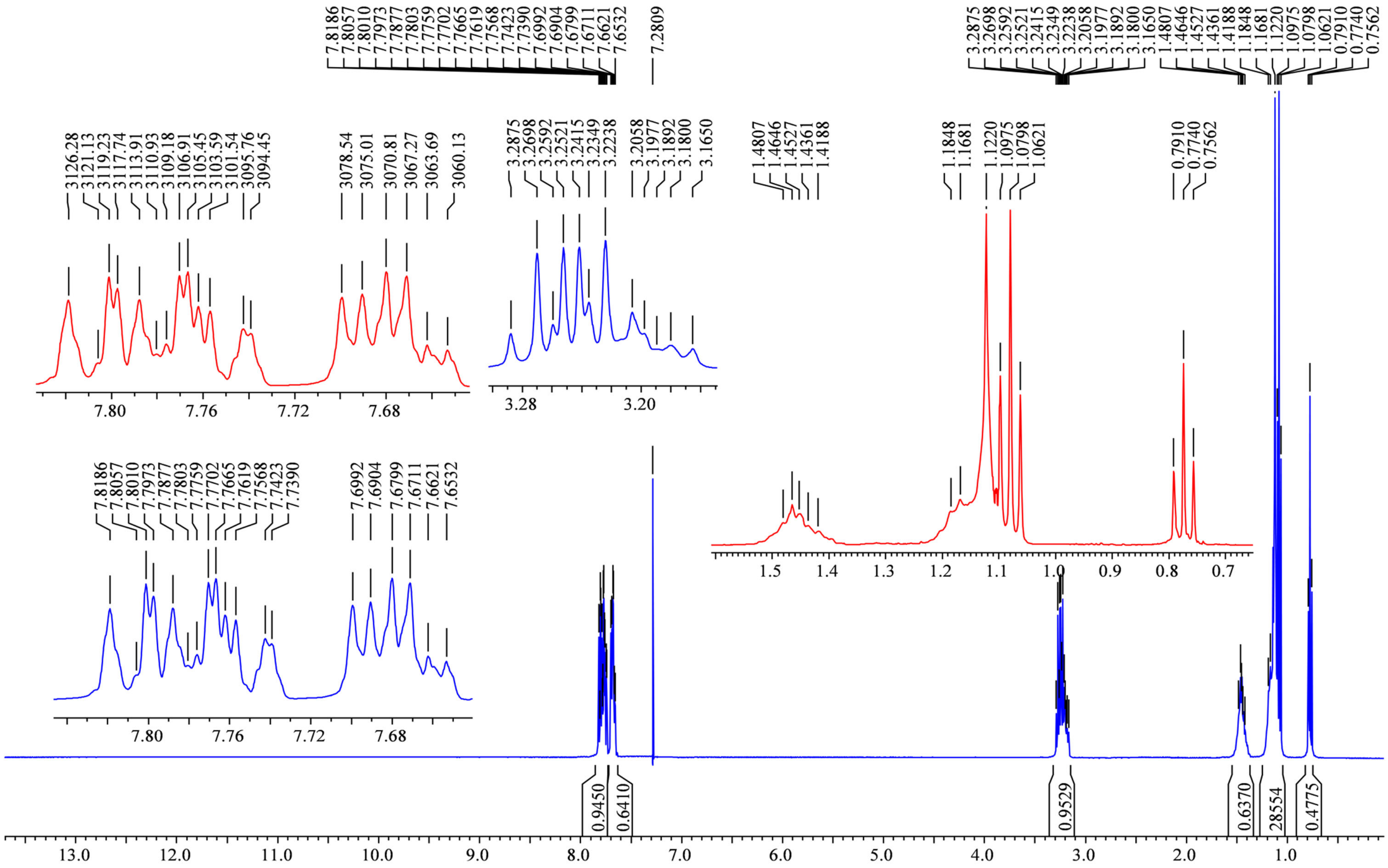

Figure 112S. ^{1}H NMR spectrum (400.0 MHz, $CDCl_3$) of $(Et_2N)Ph_2P^{+}–C_{10}H_{21}$ $I^{–}$ (**6d**).

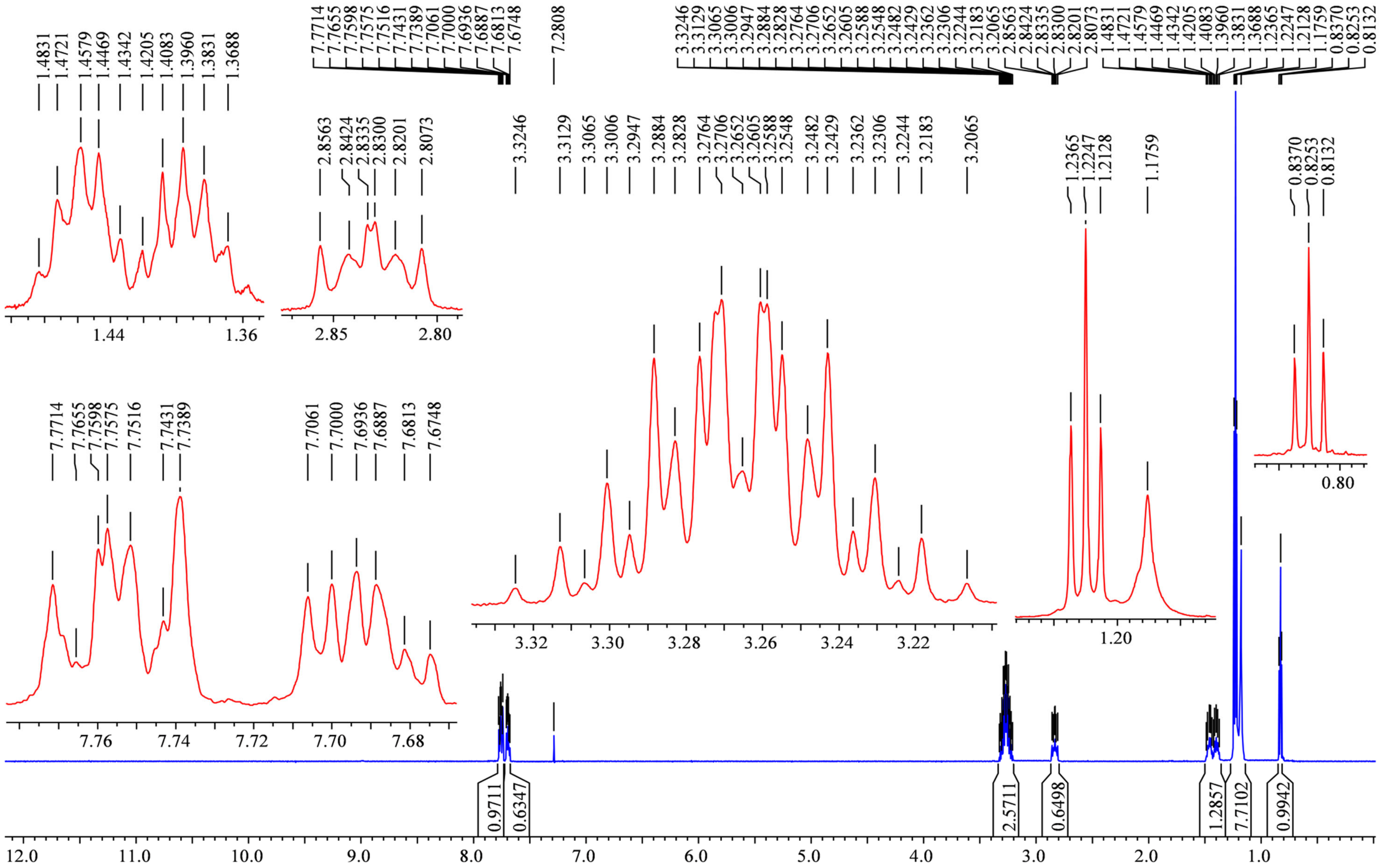

Figure 113S. ^{1}H NMR spectrum (600.0 MHz, $CDCl_3$) of $(Et_2N)Ph_2P^+–C_{10}H_{21}\ I^-$ (**6d**).

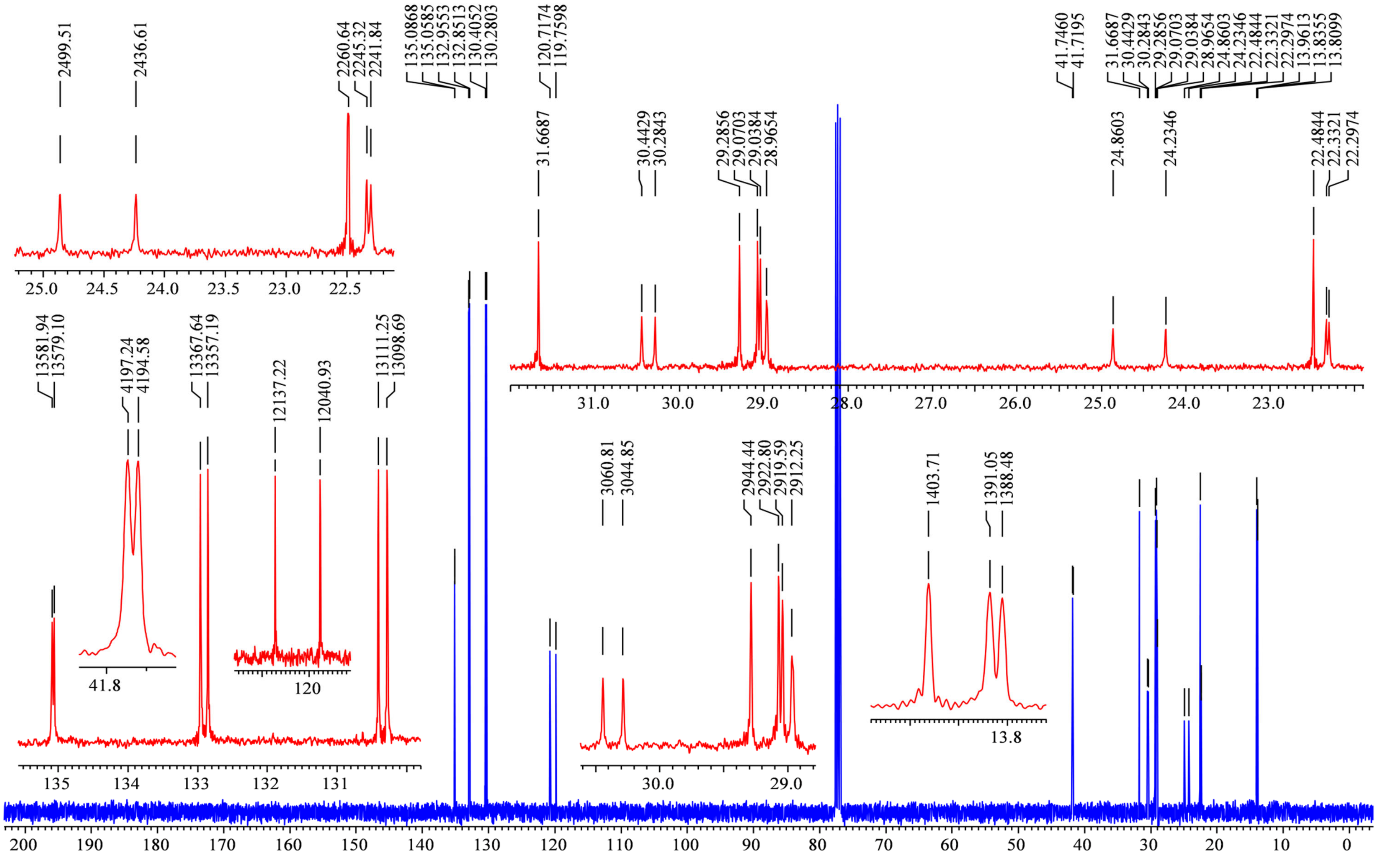

Figure 114S. ^{13}C-$\{^1H\}$ NMR spectrum (100.6 MHz, $CDCl_3$) of $(Et_2N)Ph_2P^+$–$C_{10}H_{21}$ I^- (**6d**).

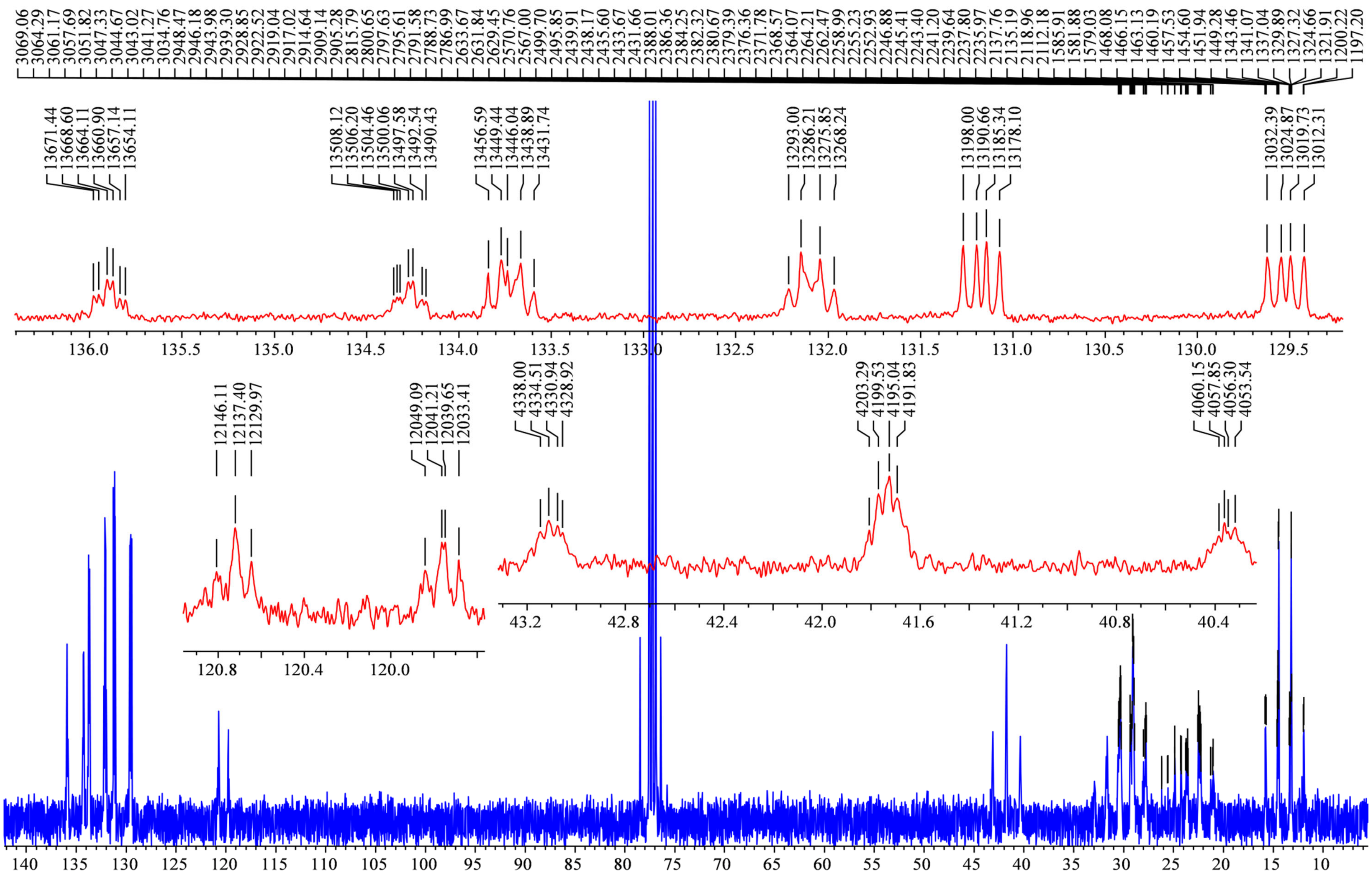

Figure 115S. ^{13}C NMR spectrum (100.6 MHz, $CDCl_3$) of $(Et_2N)Ph_2P^+–C_{10}H_{21}$ I^- (**6d**).

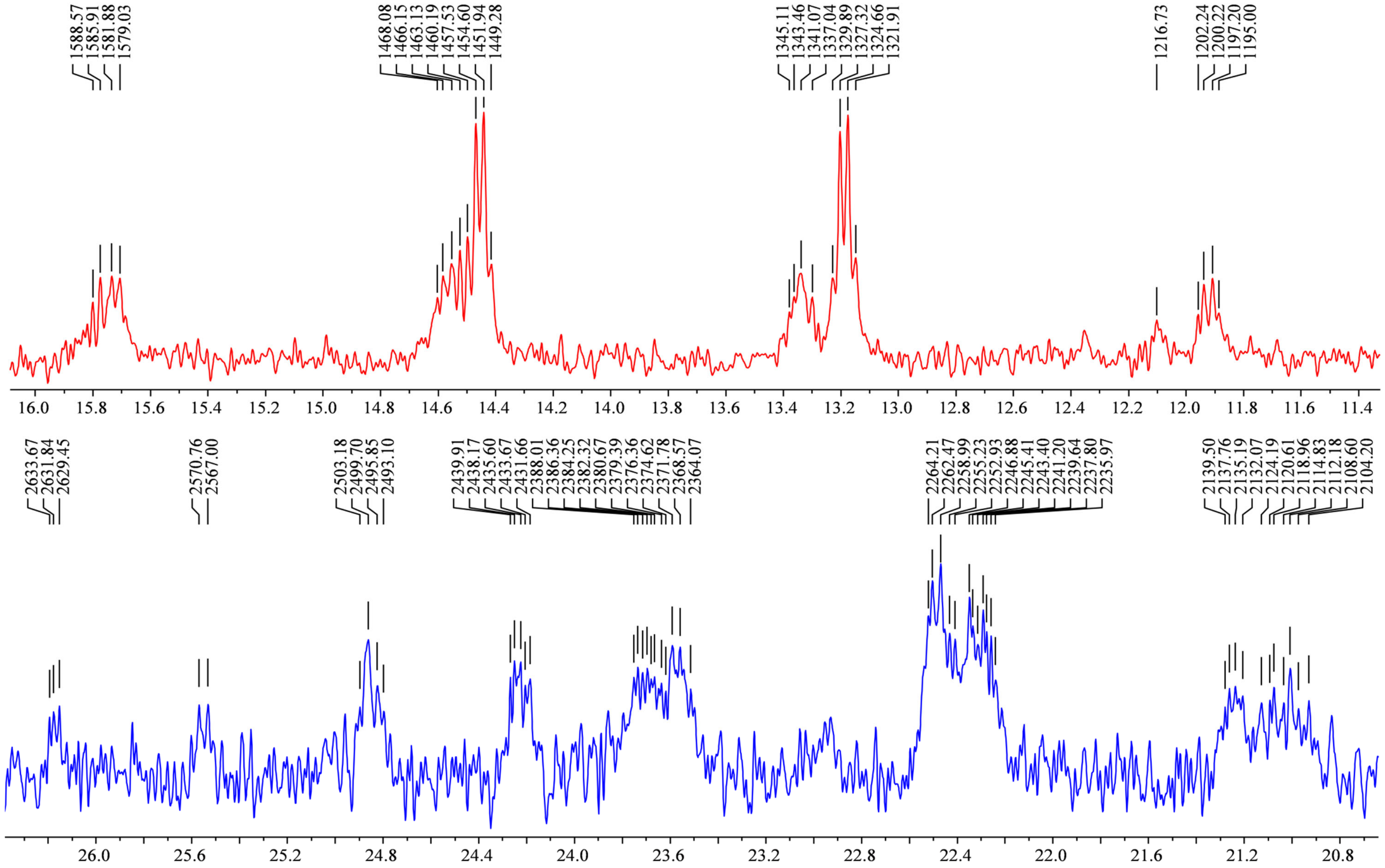

Figure 116S. High-field fragments of ^{13}C NMR spectrum (100.6 MHz, $CDCl_3$) of $(Et_2N)Ph_2P^+–C_{10}H_{21}\ I^-$ (**6d**).

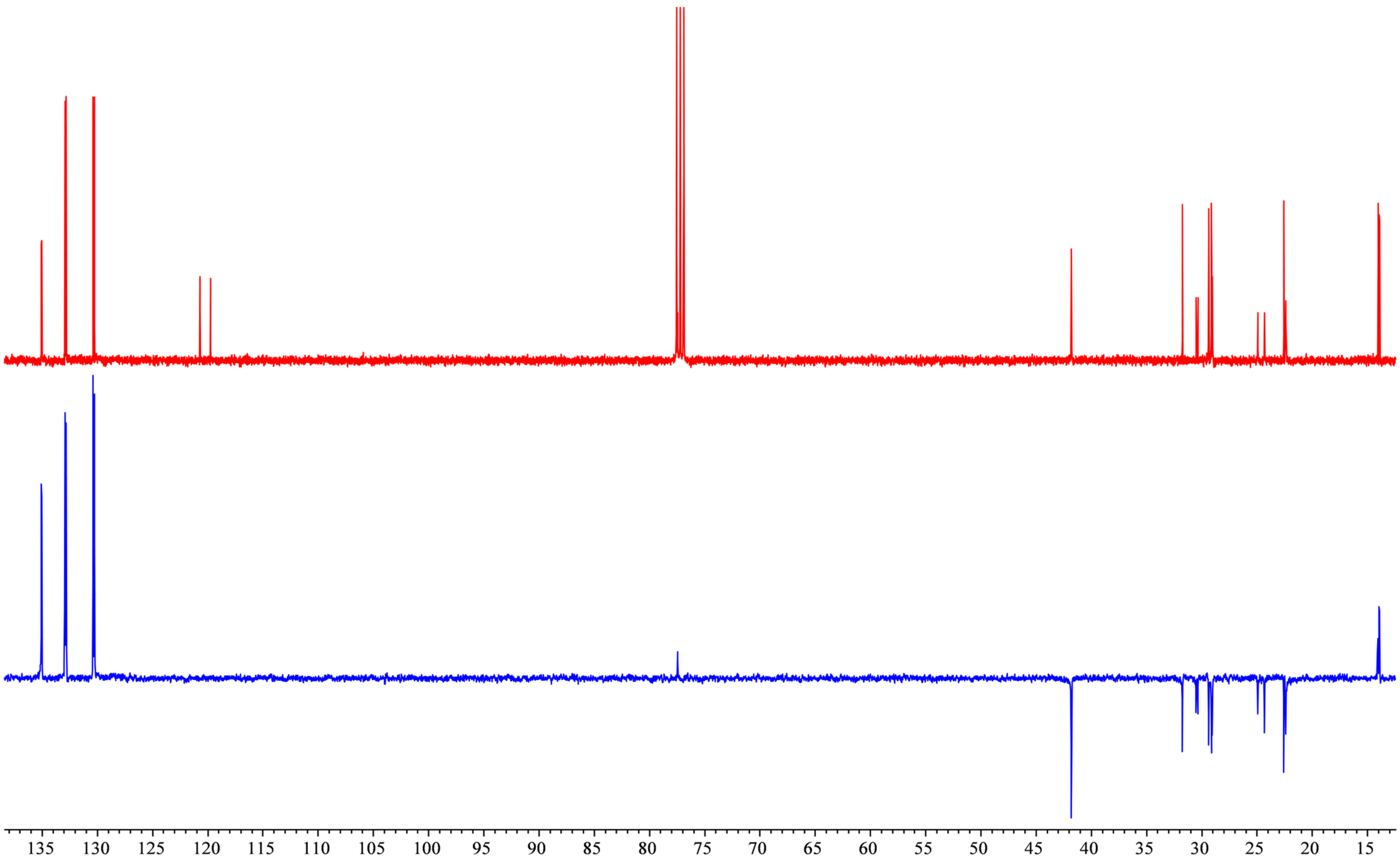

Figure 117S. ^{13}C-{^{1}H} and ^{13}C-{^{1}H}-dept NMR spectra (100.6 MHz, $CDCl_3$) of $(Et_2N)Ph_2P^{+}–C_{10}H_{21}\ I^{-}$ (**6d**).

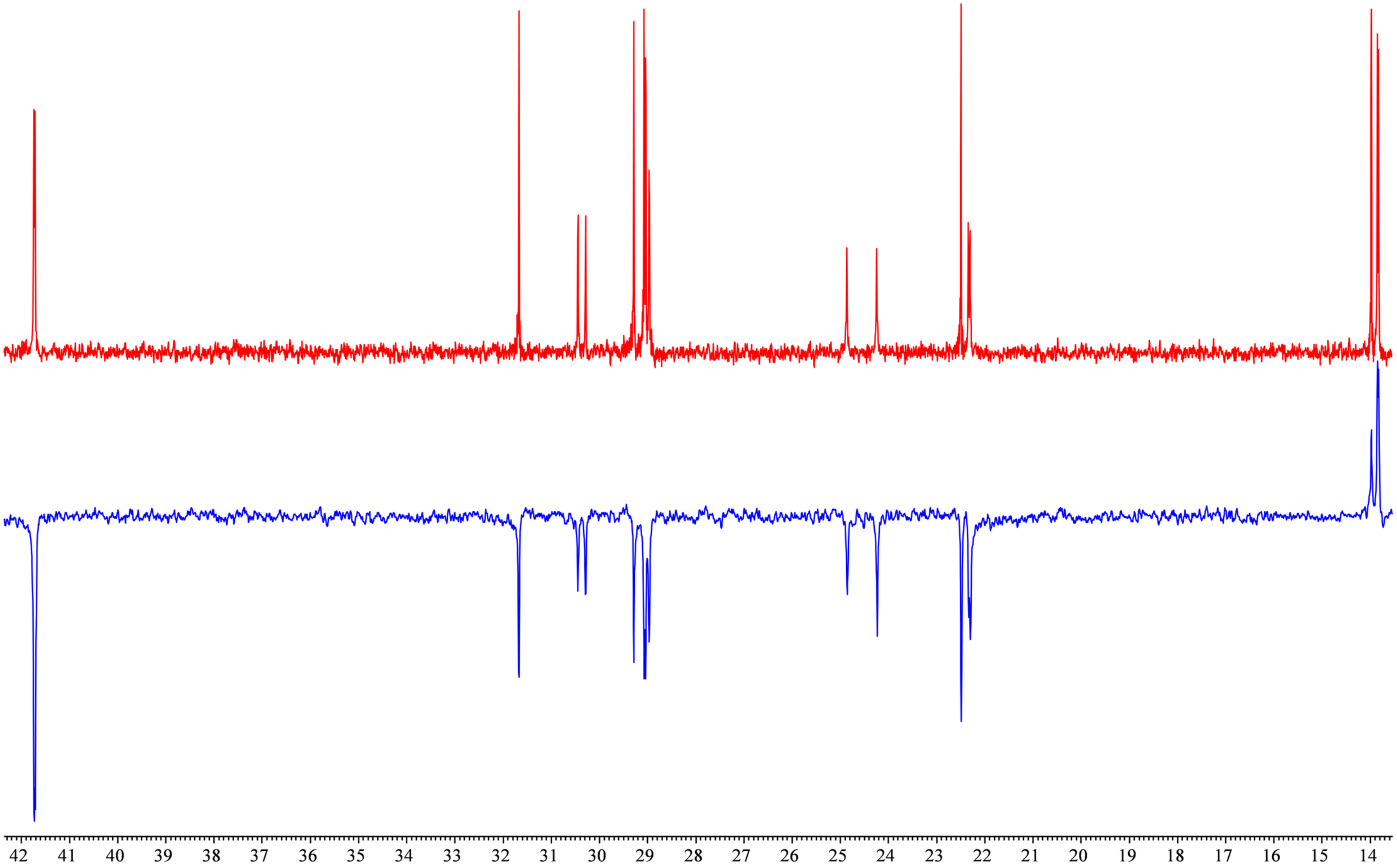

Figure 118S. High-field fragment of ^{13}C-{^{1}H} and ^{13}C-{^{1}H}-dept NMR spectra (100.6 MHz, $CDCl_3$) of $(Et_2N)Ph_2P^+–C_{10}H_{21}\ I^-$ (**6d**).

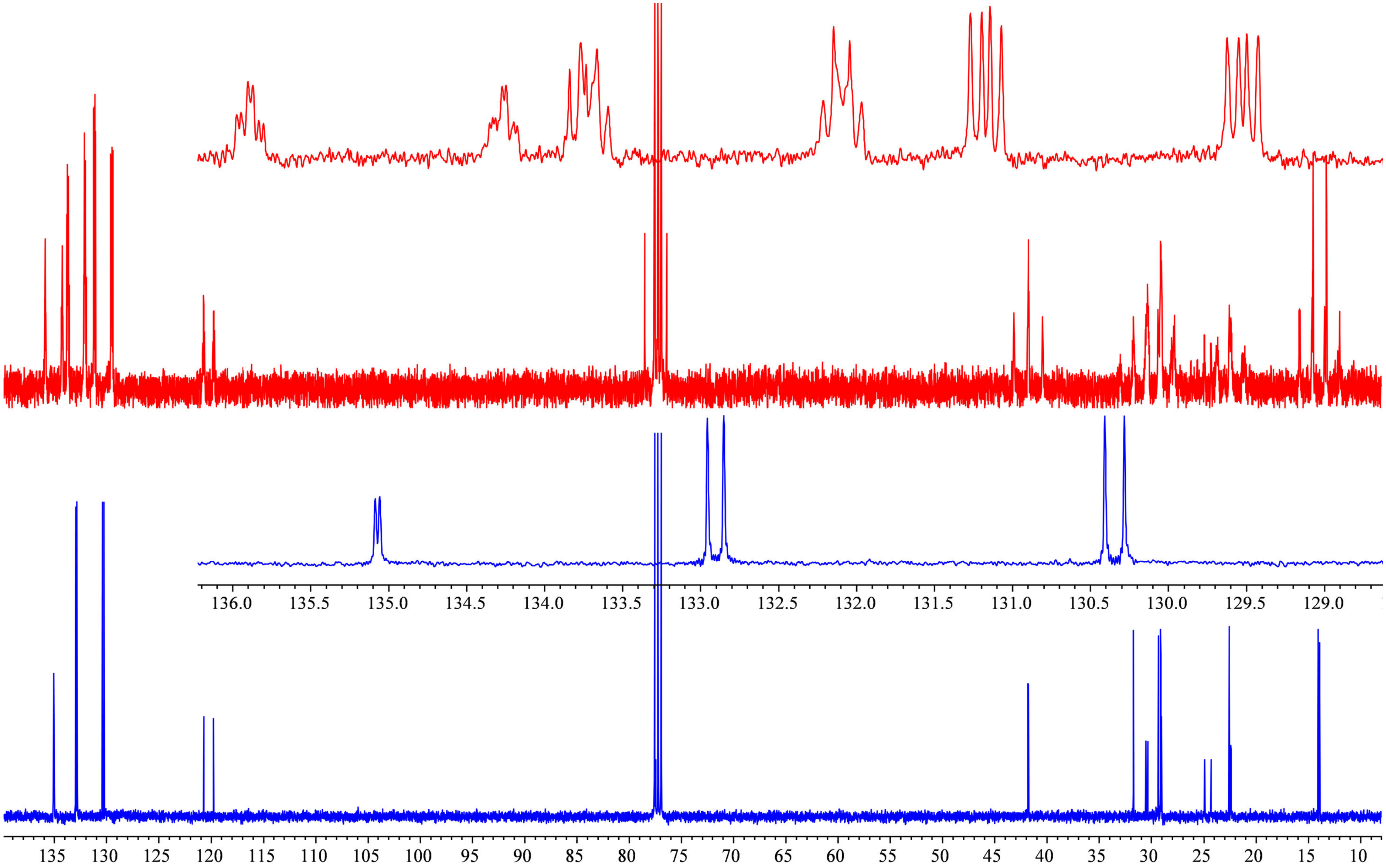

Figure 119S. ^{13}C-{^{1}H} and ^{13}C NMR spectra (100.6 MHz, $CDCl_3$) of $(Et_2N)Ph_2P^+–C_{10}H_{21}$ $I^–$ (**6d**).

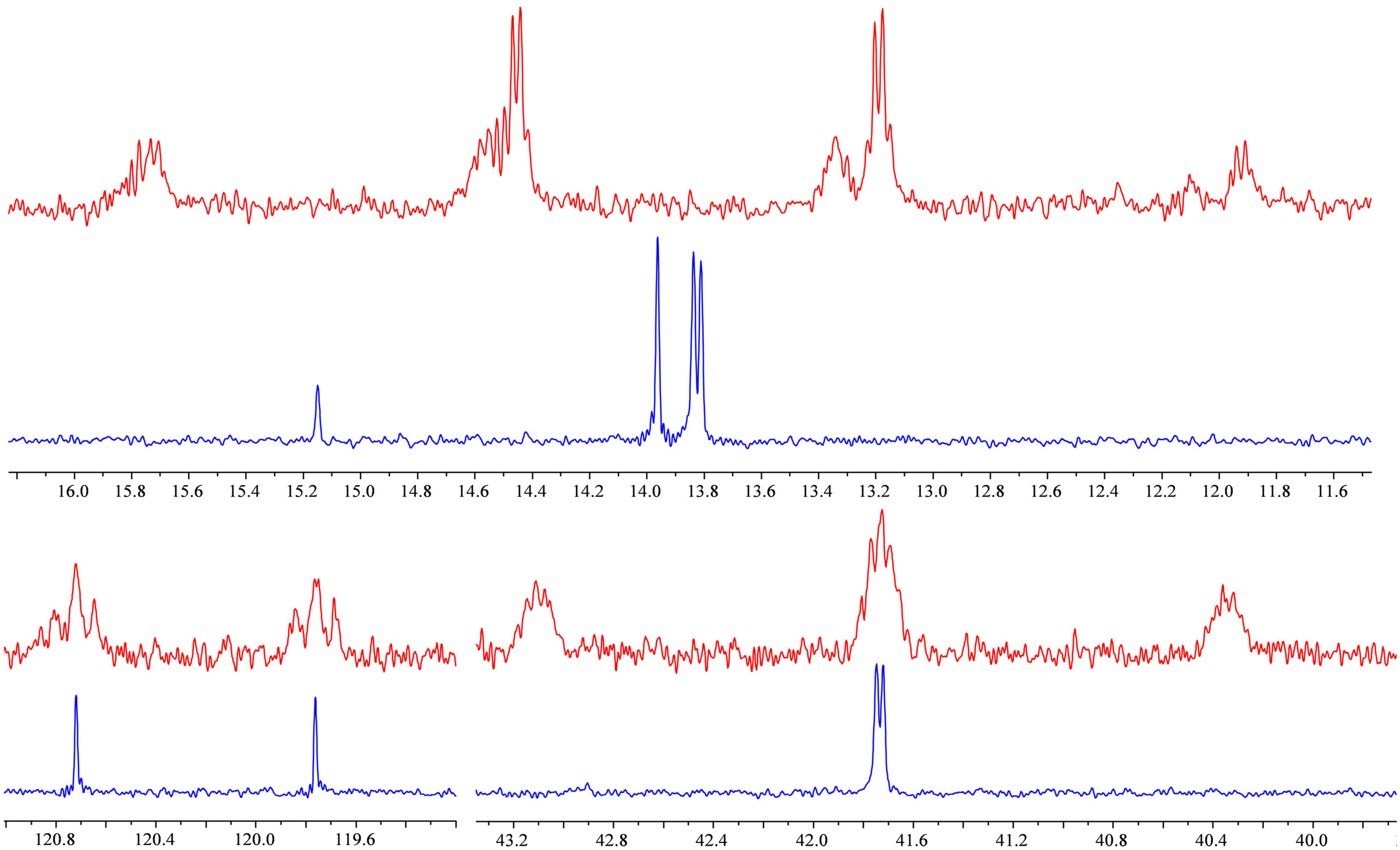

Figure 120S. The 119-121, 40-44 and 11-16 ppm regions of ^{13}C-{^{1}H} and ^{13}C NMR spectra (100.6 MHz, $CDCl_3$) of $(Et_2N)Ph_2P^{+}–C_{10}H_{21}$ $I^{–}$ (**6d**).

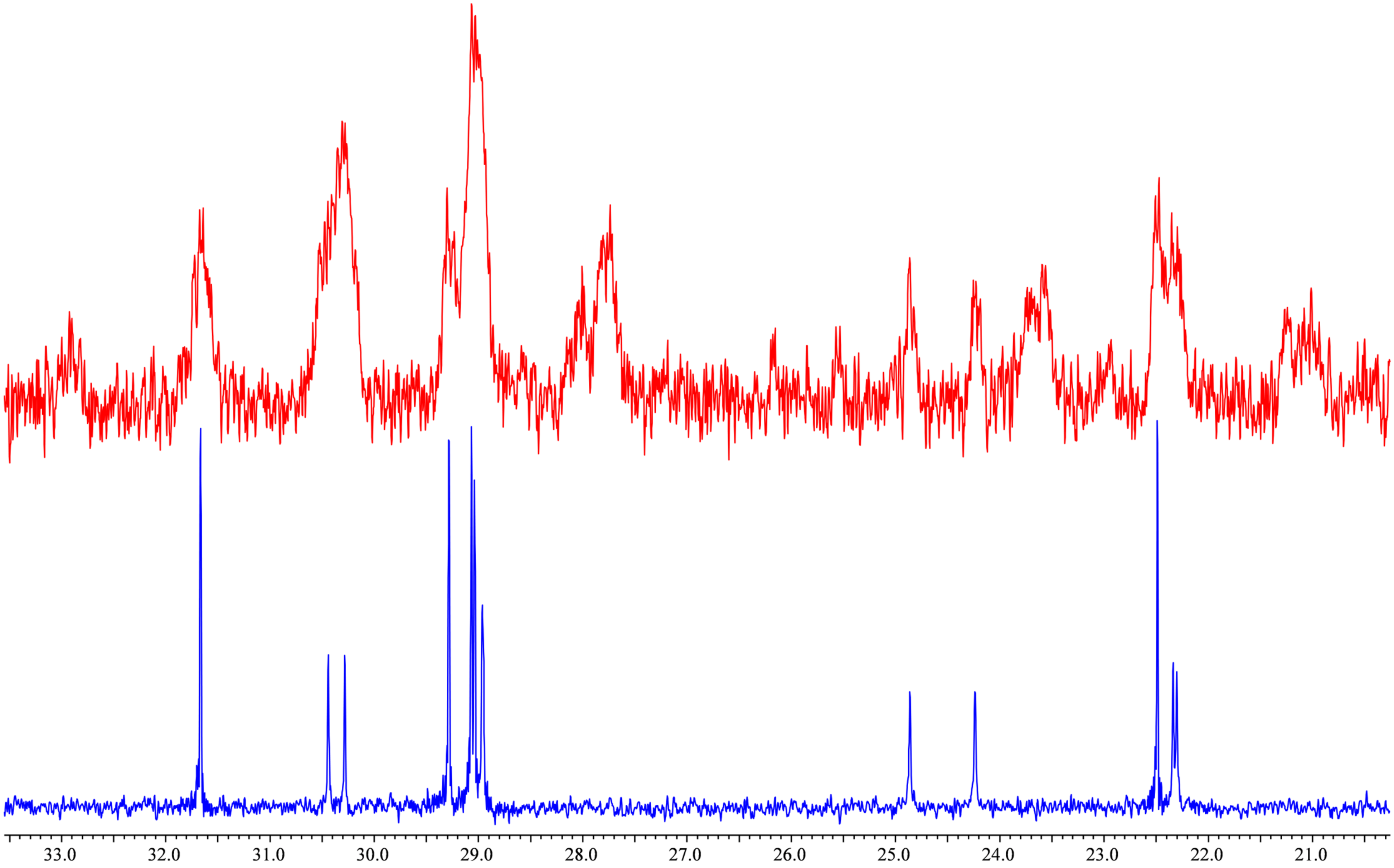

Figure 121S. The 20-34 ppm region of $^{13}C-\{^{1}H\}$ and ^{13}C NMR spectra (100.6 MHz, $CDCl_3$) of $(Et_2N)Ph_2P^{+}–C_{10}H_{21}\ I^{-}$ (**6d**).

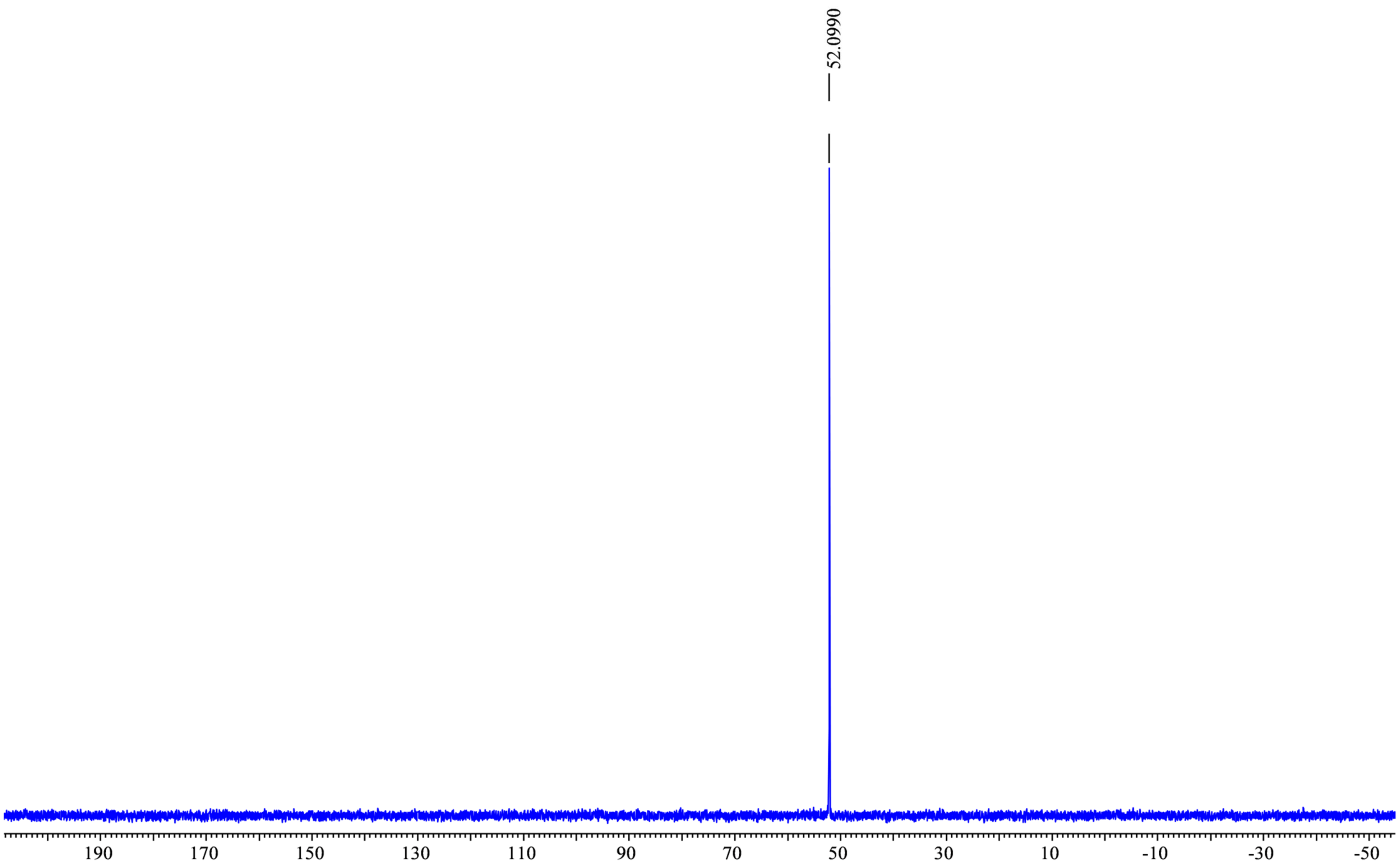

Figure 122S. ^{31}P-$\{^{1}H\}$ NMR spectrum (162.0 MHz, $CDCl_3$) of $(Et_2N)Ph_2P^{+}$–$C_{14}H_{29}$ Br^{-} (**6e**).

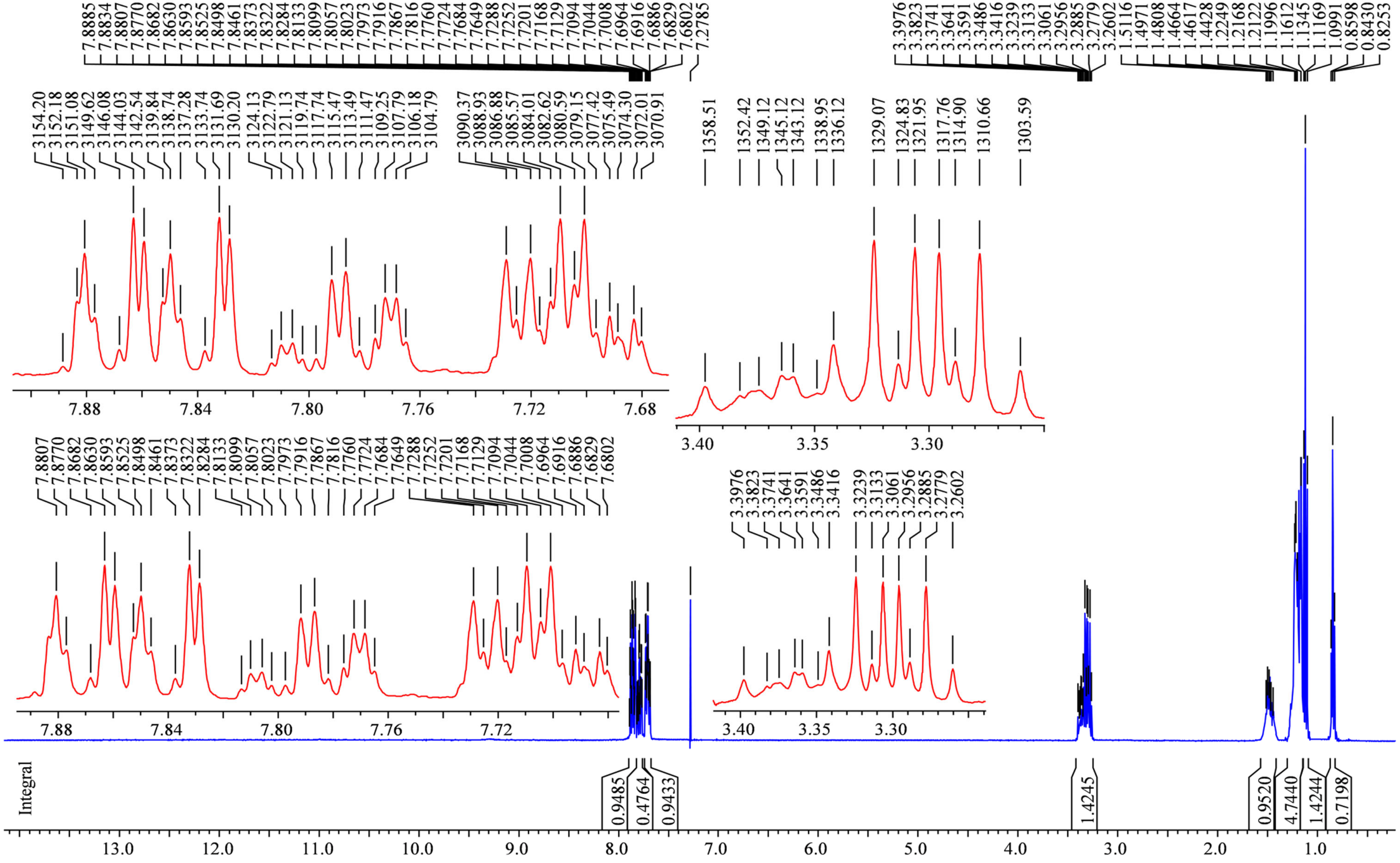

Figure 123S. ^{1}H NMR spectrum (400.0 MHz, $CDCl_3$) of $(Et_2N)Ph_2P^{+}–C_{14}H_{29}\ Br^{-}$ (**6e**).

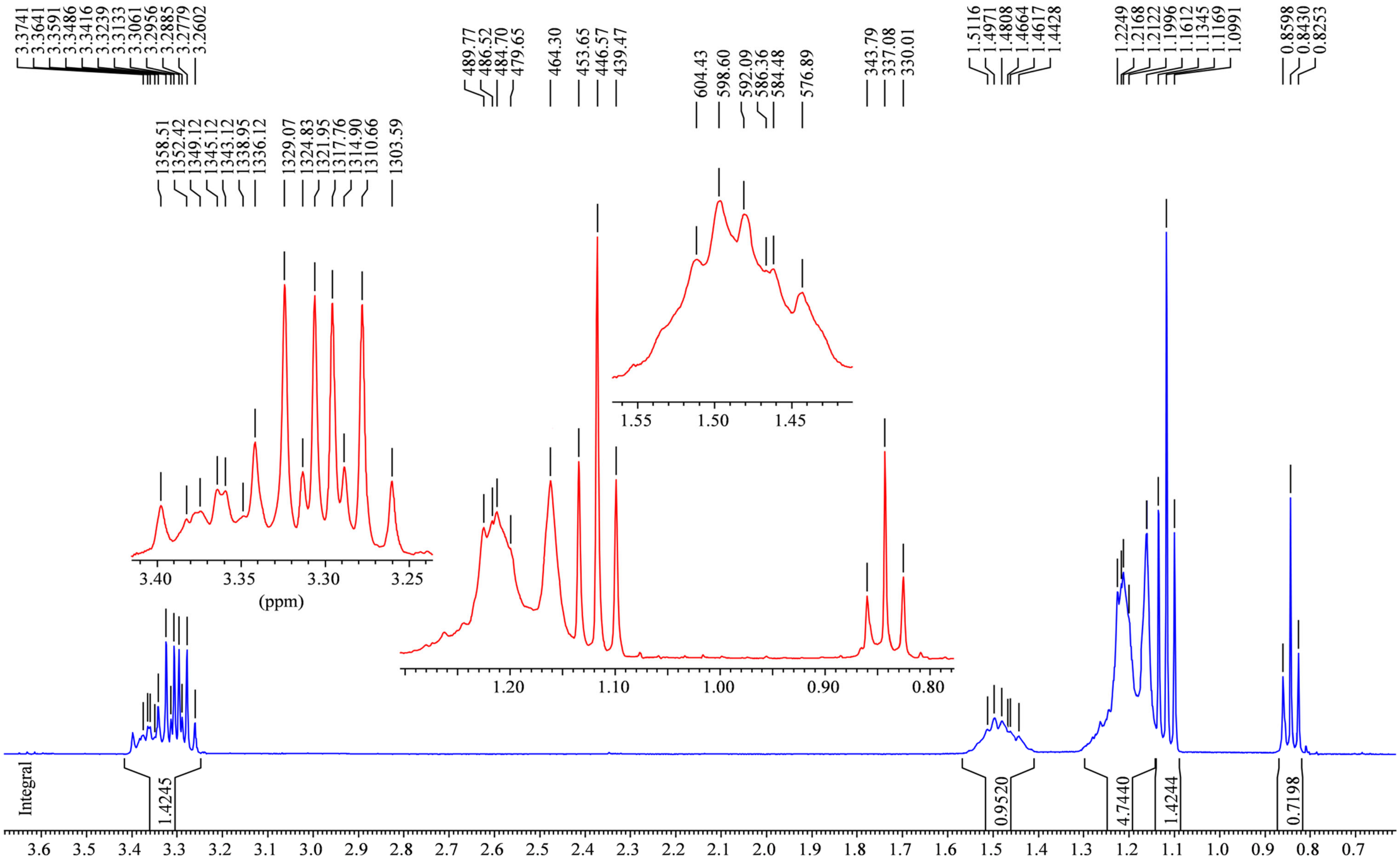

Figure 124S. High-field fragment of ^{1}H NMR spectrum (400.0 MHz, $CDCl_3$) of $(Et_2N)Ph_2P^+–C_{14}H_{29}$ $Br^–$ (**6e**).

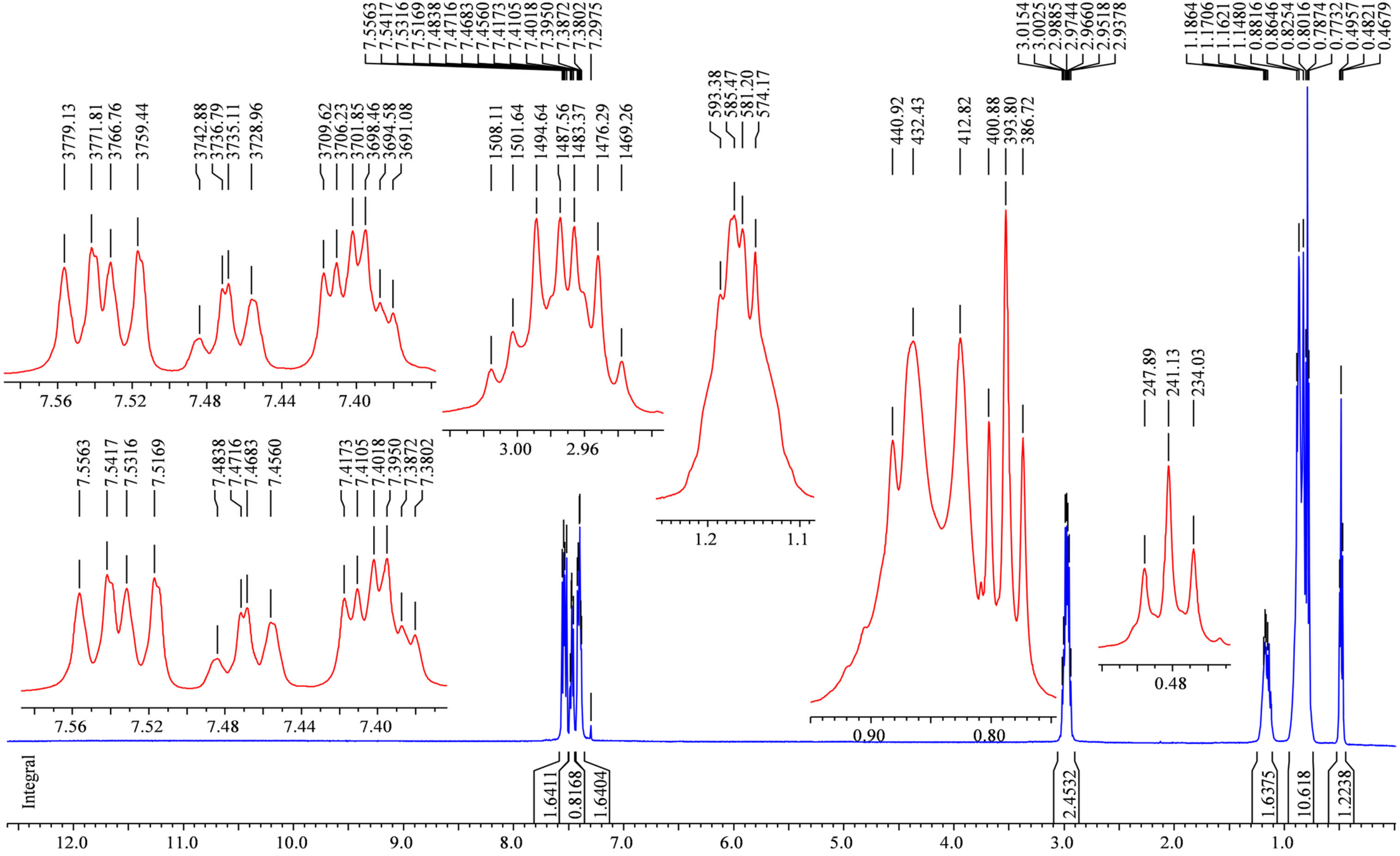

Figure 125S. ^{1}H NMR spectrum (500.0 MHz, $CDCl_3$) of $(Et_2N)Ph_2P^{+}–C_{14}H_{29}$ Br^{-} (**6e**).

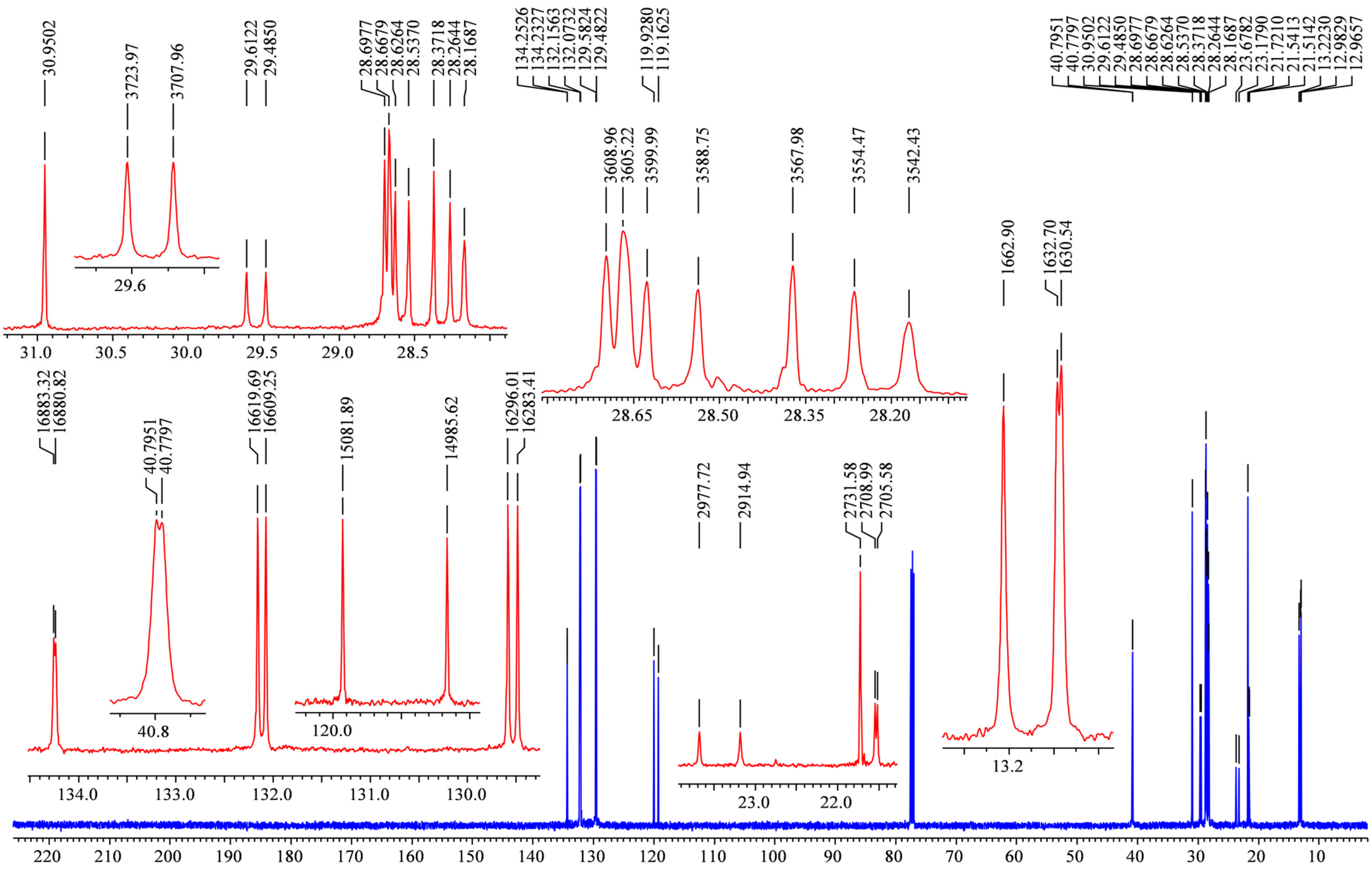

Figure 126S. ^{13}C-{^{1}H} NMR spectrum (125.8 MHz, CDCl$_3$) of (Et$_2$N)Ph$_2$P$^+$–C$_{14}$H$_{29}$ Br$^-$ (**6e**).

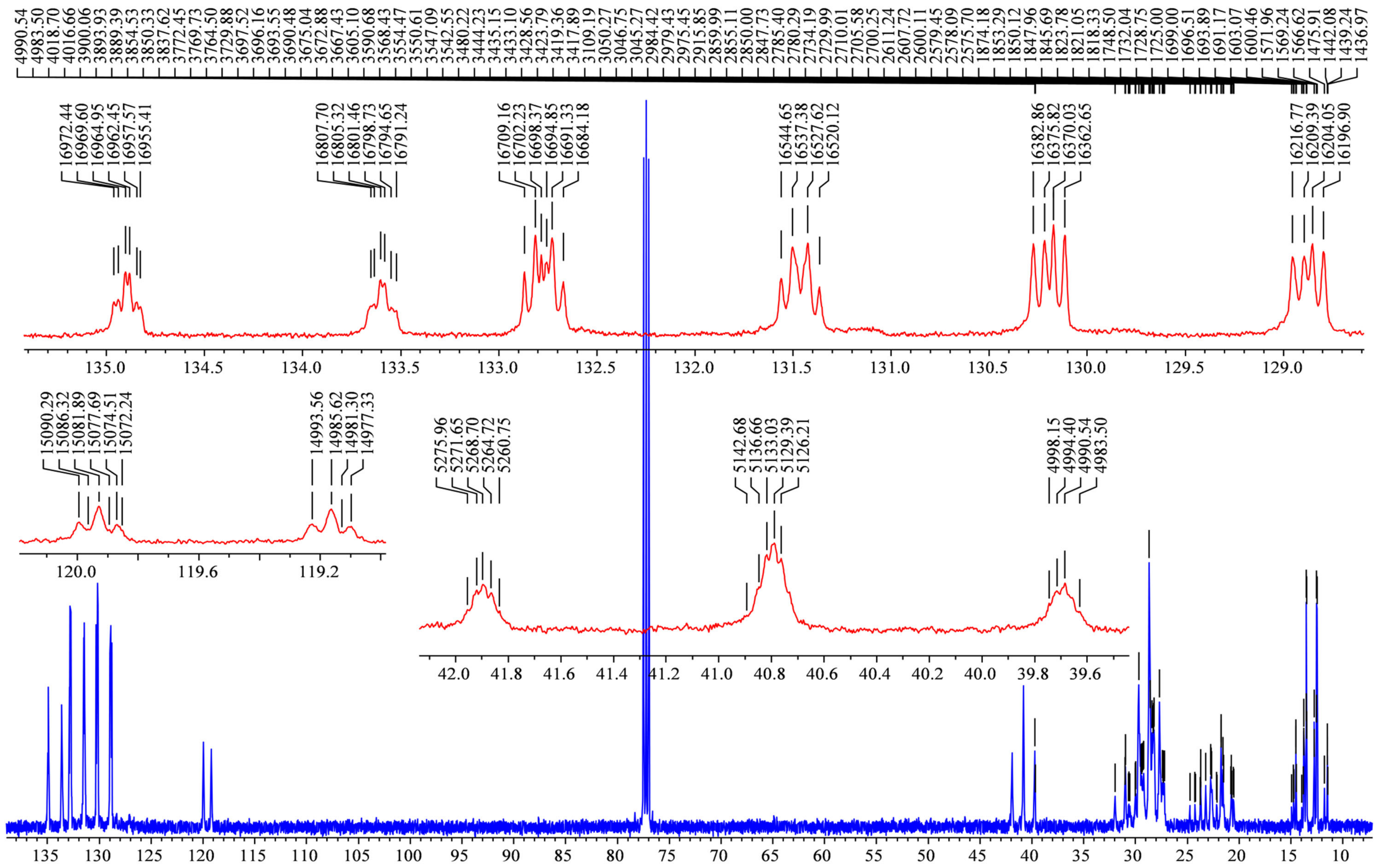

Figure 127S. ^{13}C NMR spectrum (125.8 MHz, $CDCl_3$) of $(Et_2N)Ph_2P^+–C_{14}H_{29}$ Br^- (**6e**).

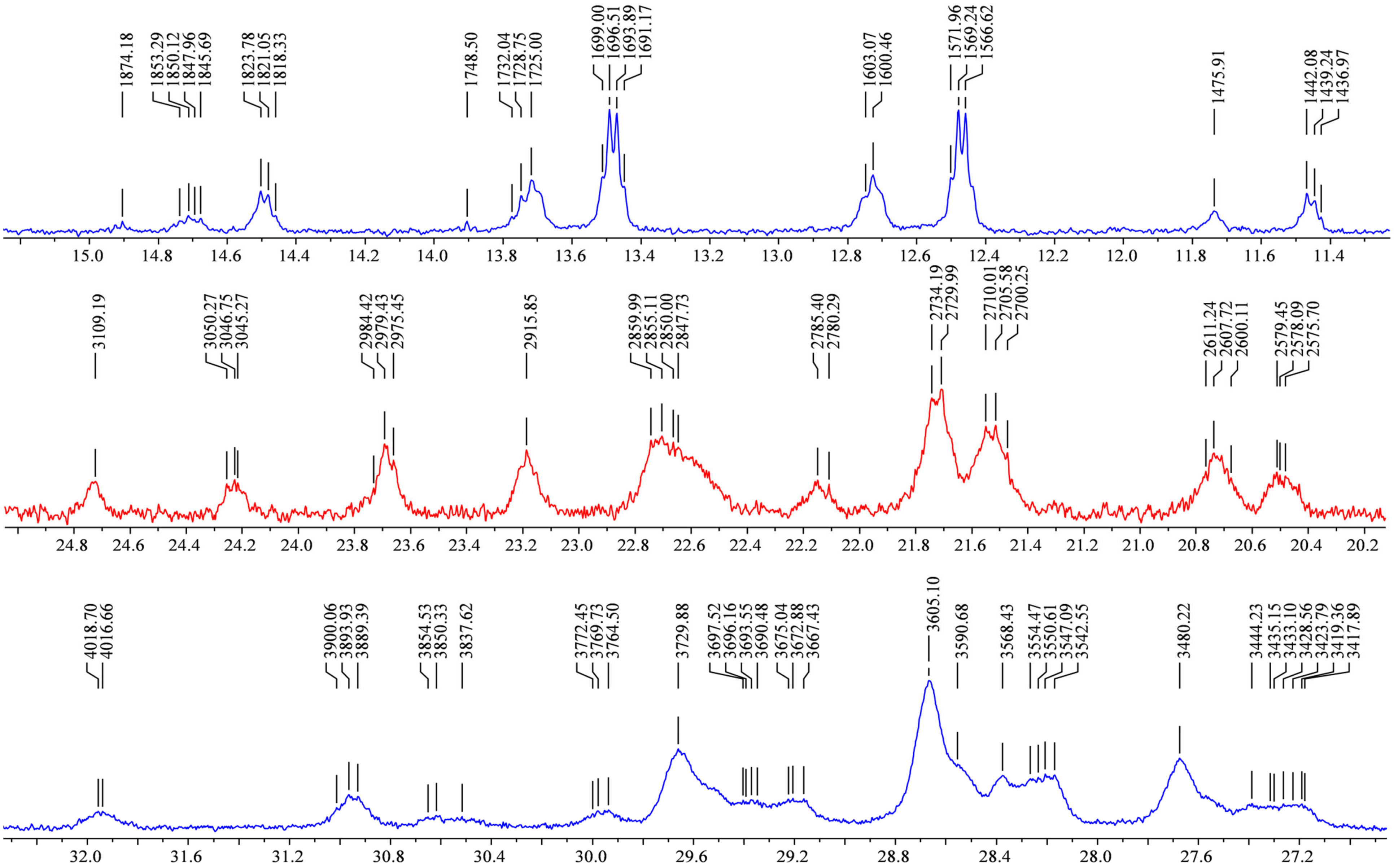

Figure 128S. High-field fragments of ^{13}C NMR spectrum (125.8 MHz, $CDCl_3$) of $(Et_2N)Ph_2P^+–C_{14}H_{29}\ Br^-$ (**6e**).

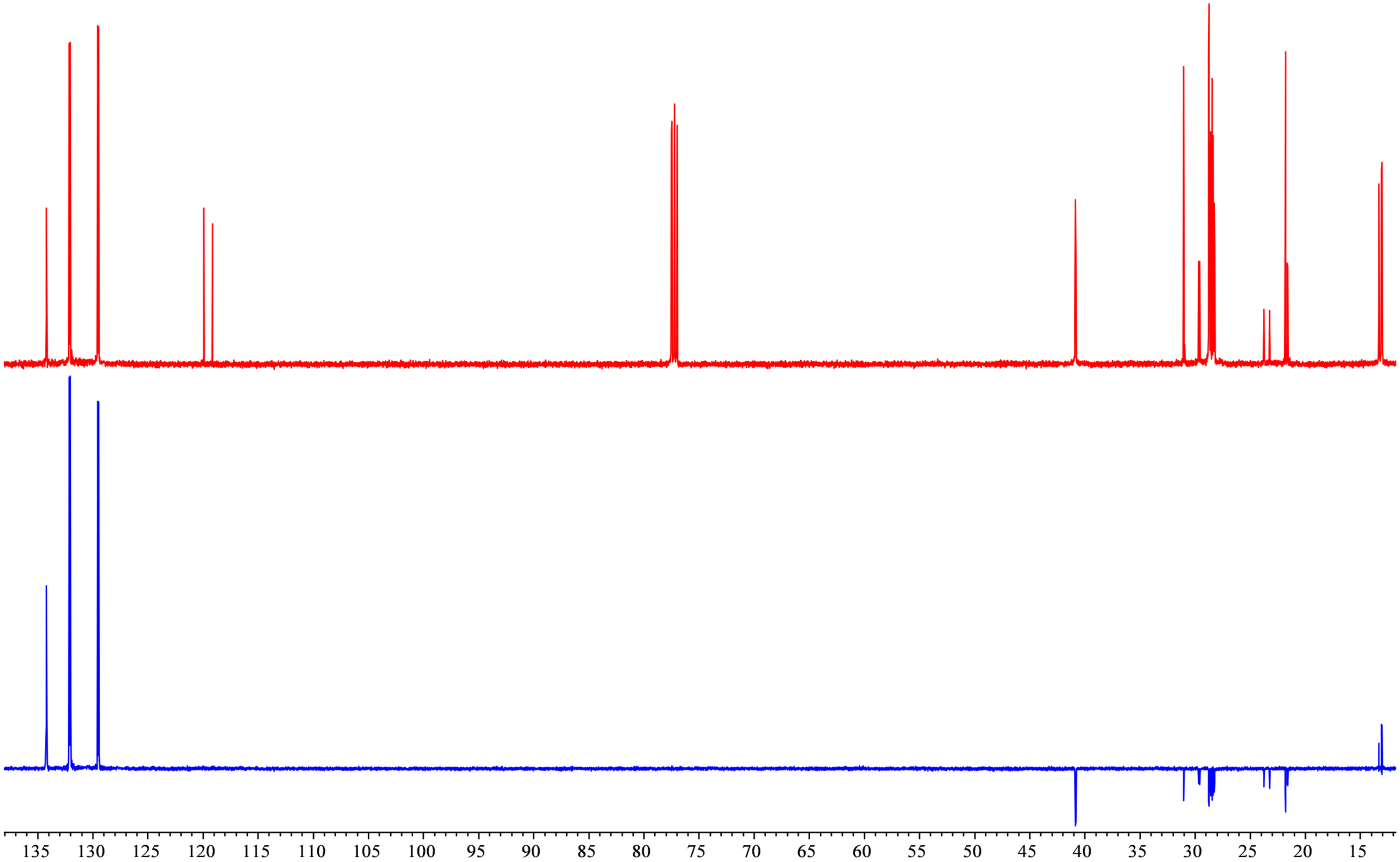

Figure 129S. $^{13}C-\{^{1}H\}$ and $^{13}C-\{^{1}H\}$-dept NMR spectra (125.8 MHz, $CDCl_3$) of $(Et_2N)Ph_2P^{+}–C_{14}H_{29}\ Br^{-}$ (**6e**).

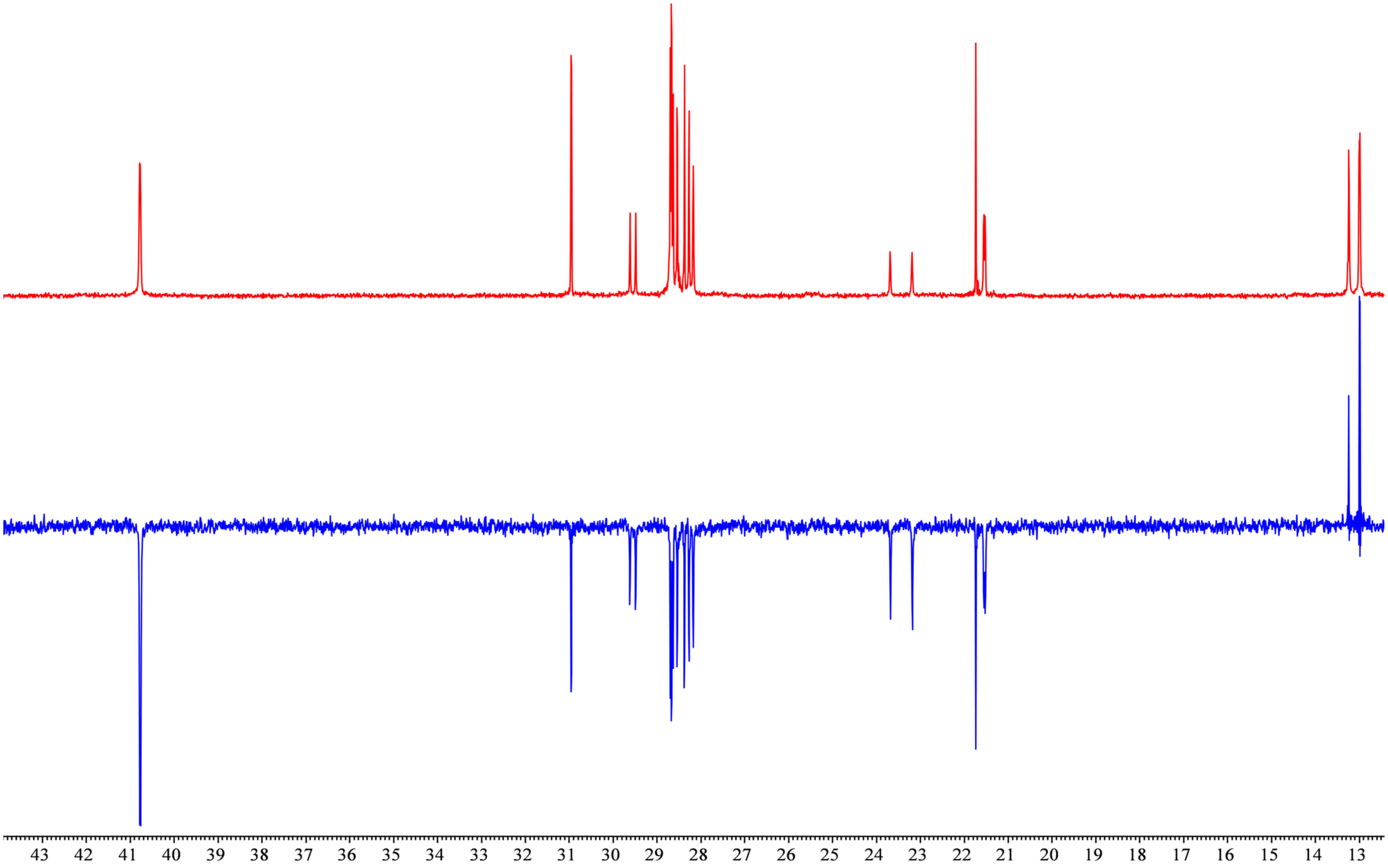

Figure 130S. High-field fragment of $^{13}C-\{^{1}H\}$ and $^{13}C-\{^{1}H\}$-dept NMR spectra (125.8 MHz, $CDCl_3$) of $(Et_2N)Ph_2P^{+}–C_{14}H_{29}\ Br^{-}$ (**6e**).

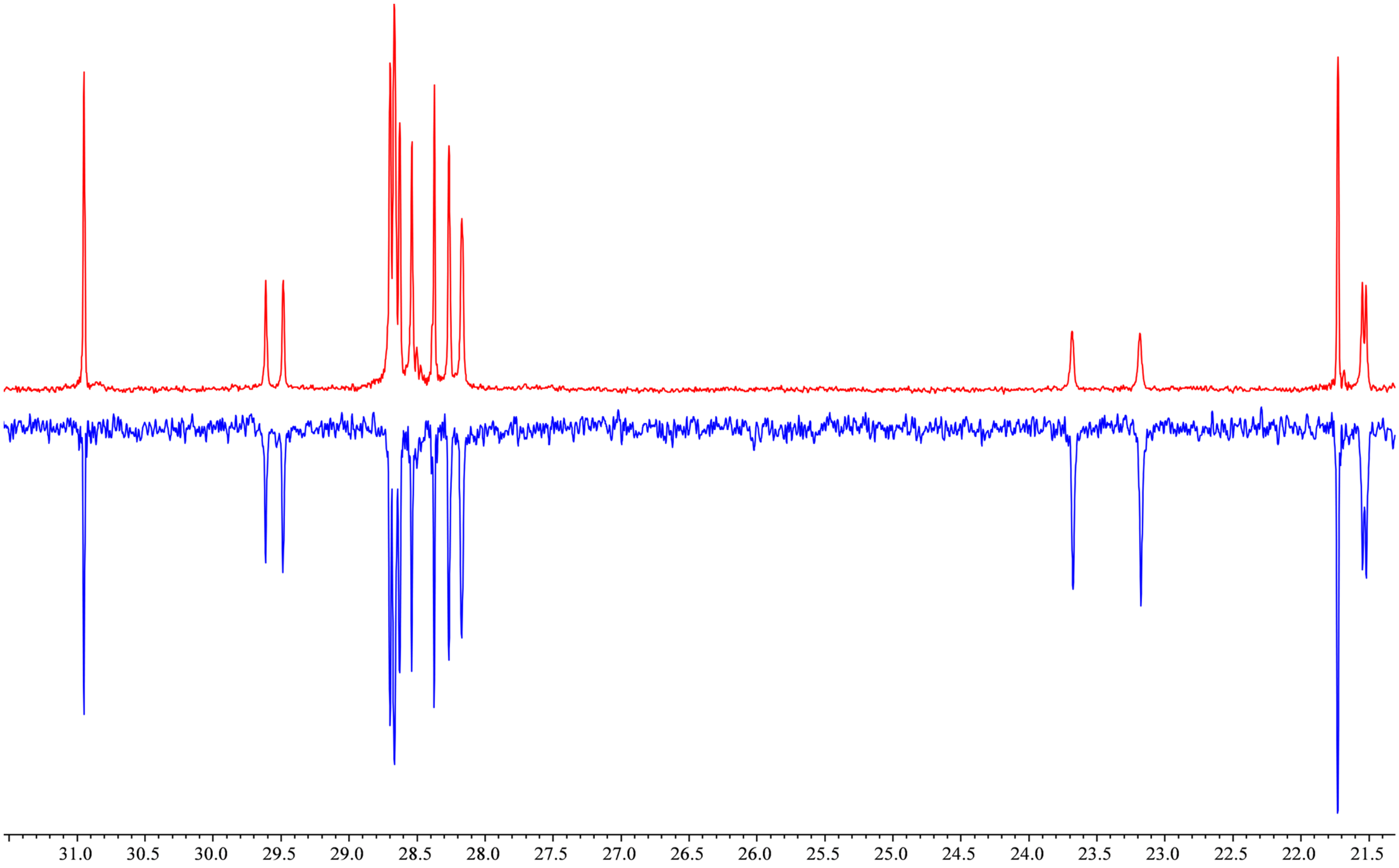

Figure 131S. The 21-32 ppm region of ^{13}C-{^{1}H} and ^{13}C-{^{1}H}-dept NMR spectra (125.8 MHz, $CDCl_3$) of $(Et_2N)Ph_2P^{+}–C_{14}H_{29}\ Br^{-}$ (**6e**).

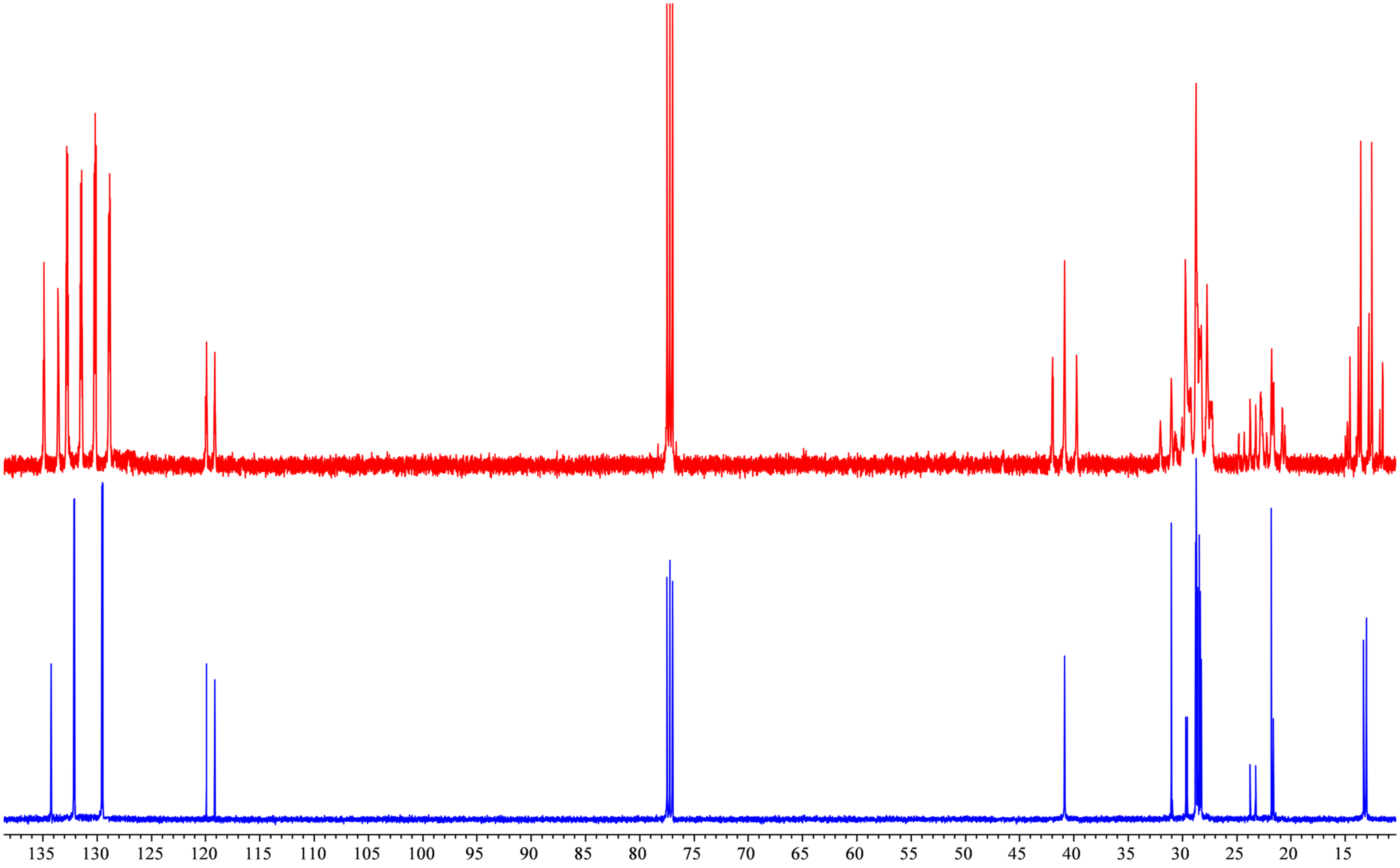

Figure 132S. ^{13}C-{^{1}H} and ^{13}C NMR spectra (125.8 MHz, $CDCl_3$) of $(Et_2N)Ph_2P^+–C_{14}H_{29}$ $Br^–$ (**6e**).

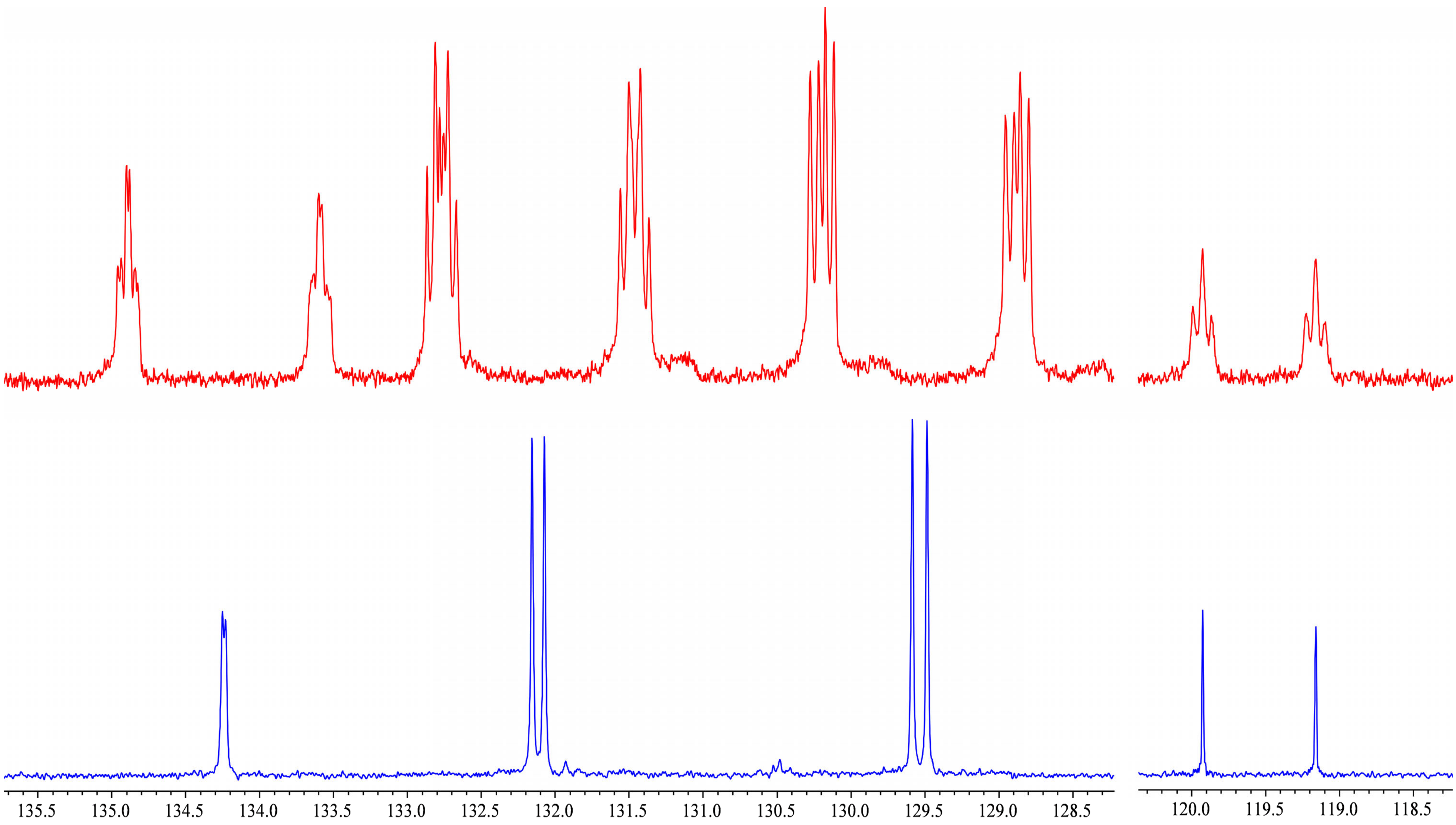

Figure 133S. Low-field fragments of ^{13}C-{^{1}H} and ^{13}C NMR spectra (125.8 MHz, $CDCl_3$) of $(Et_2N)Ph_2P^{+}$–$C_{14}H_{29}$ Br^{-} (**6e**).

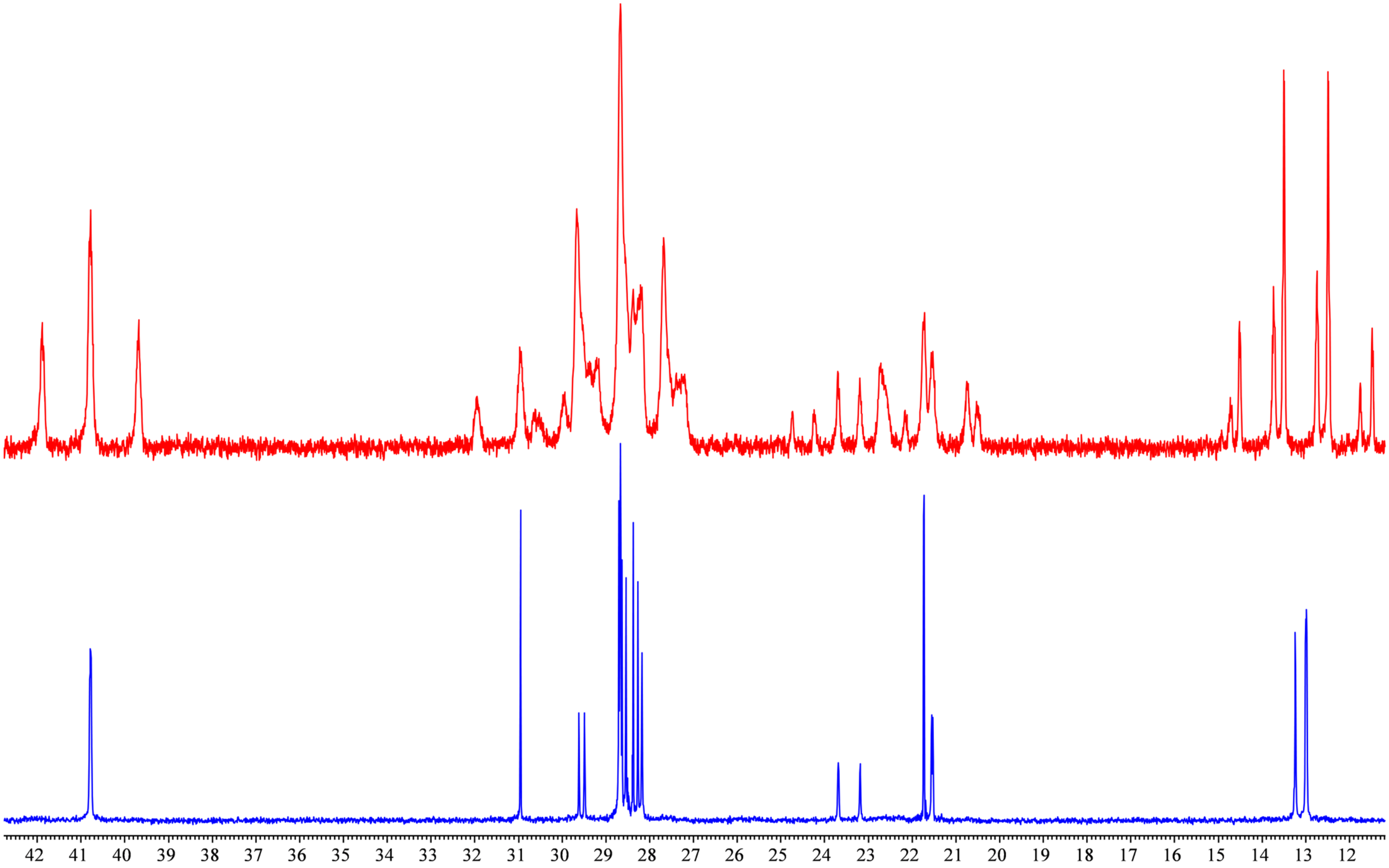

Figure 134S. High-field fragment of ^{13}C-{^{1}H} and ^{13}C NMR spectra (125.8 MHz, $CDCl_3$) of $(Et_2N)Ph_2P^{+}$–$C_{14}H_{29}$ Br^{-} (**6e**).

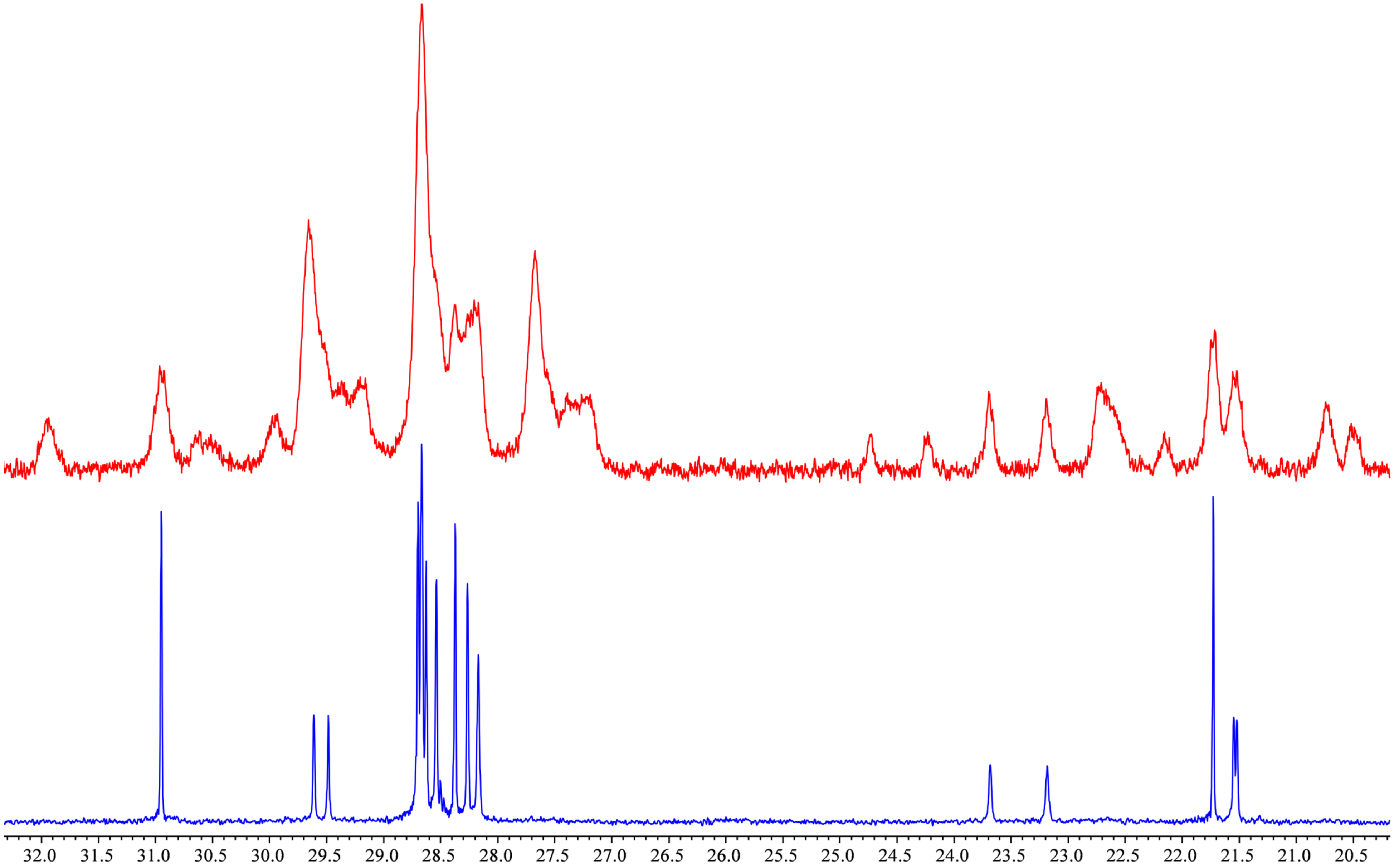

Figure 135S. The 20-33 ppm region of $^{13}C-\{^{1}H\}$ and ^{13}C NMR spectra (125.8 MHz, $CDCl_3$) of $(Et_2N)Ph_2P^+–C_{14}H_{29}\ Br^−$ (**6e**).

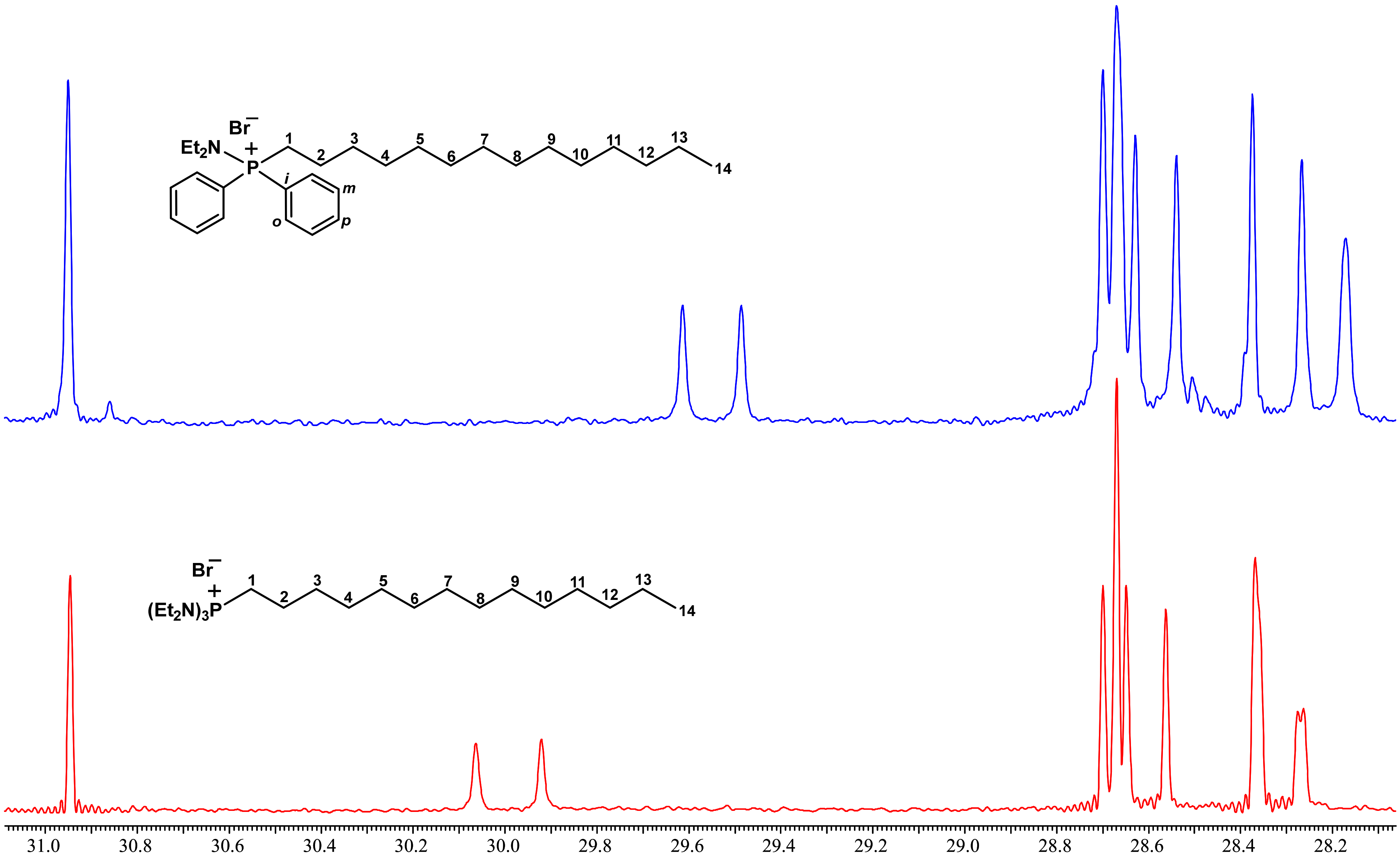

Figure 136S. The 28-31 ppm region of ^{13}C-{^{1}H} spectra (125.8 MHz, $CDCl_3$) of $(Et_2N)Ph_2P^+–C_{14}H_{29}$ $Br^–$ (**6e**) and $(Et_2N)_3P^+–C_{14}H_{29}$ $Br^–$ (**4e**).

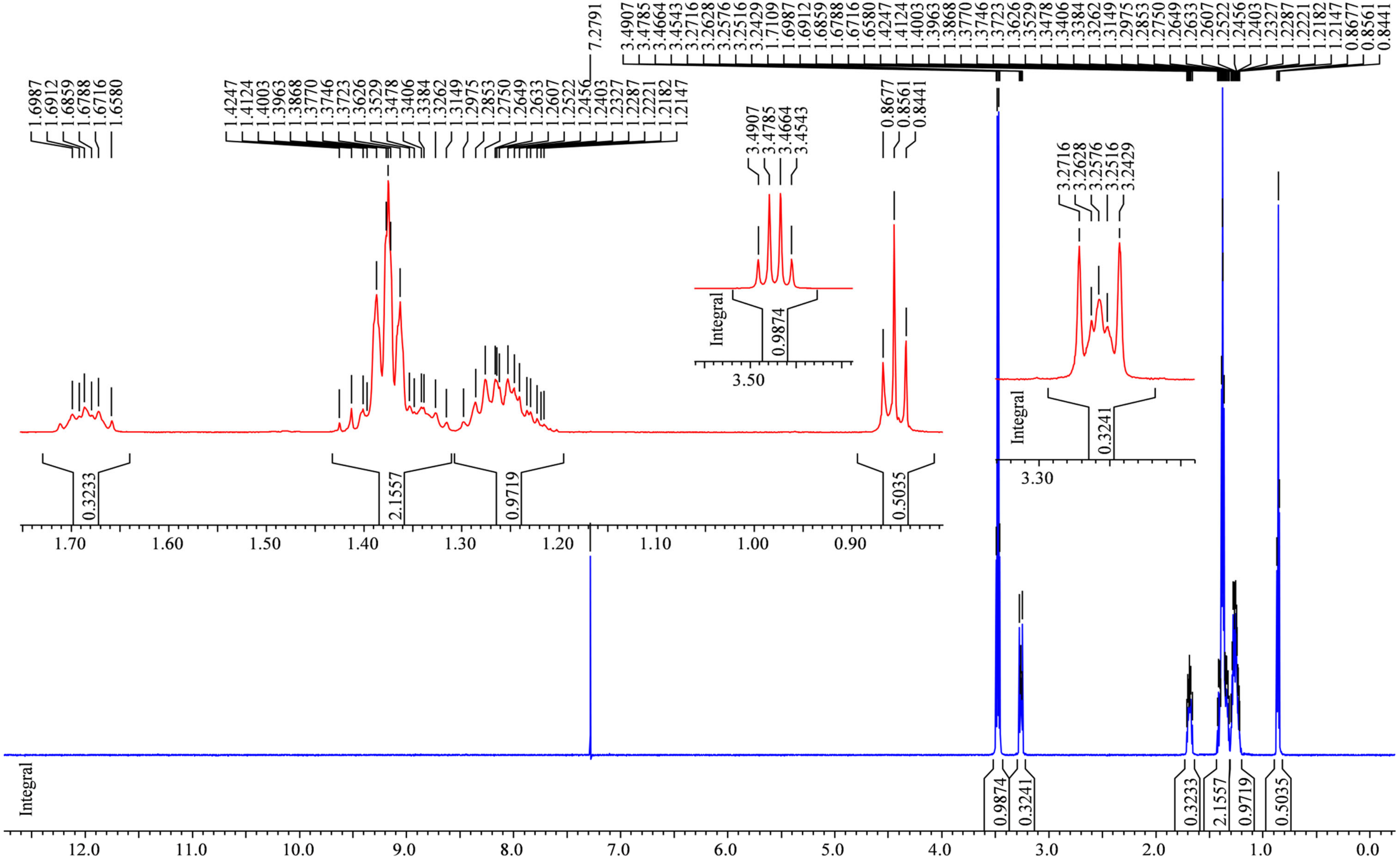

Figure 137S. ^{1}H NMR spectrum (600.0 MHz, $CDCl_3$) of $Et_3N^+–C_8H_{17}$ $I^–$ (**7**).

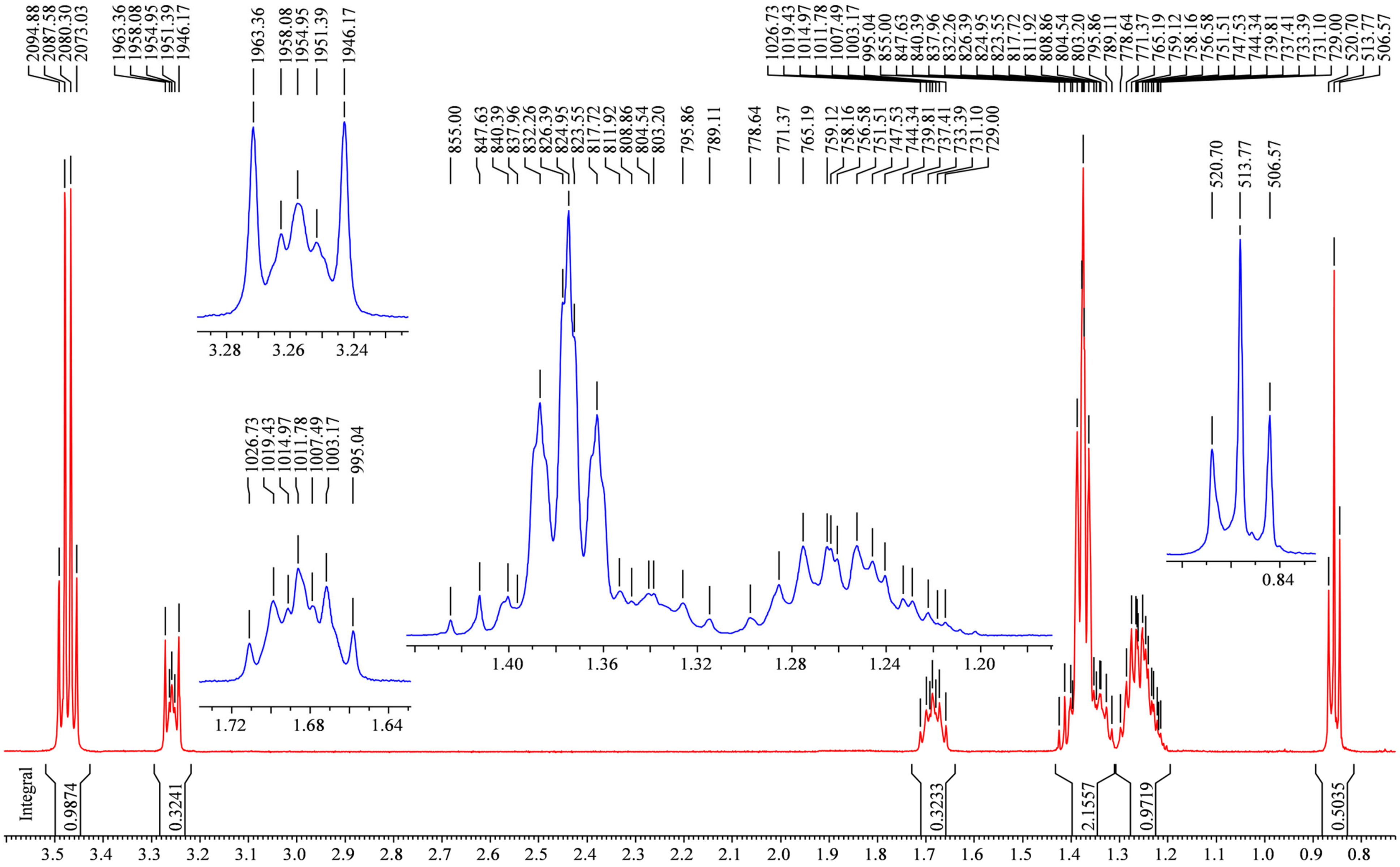

Figure 138S. High-field fragment of ^{1}H NMR spectrum (600.0 MHz, $CDCl_3$) of $Et_3N^+–C_8H_{17}\ I^-$ (**7**).

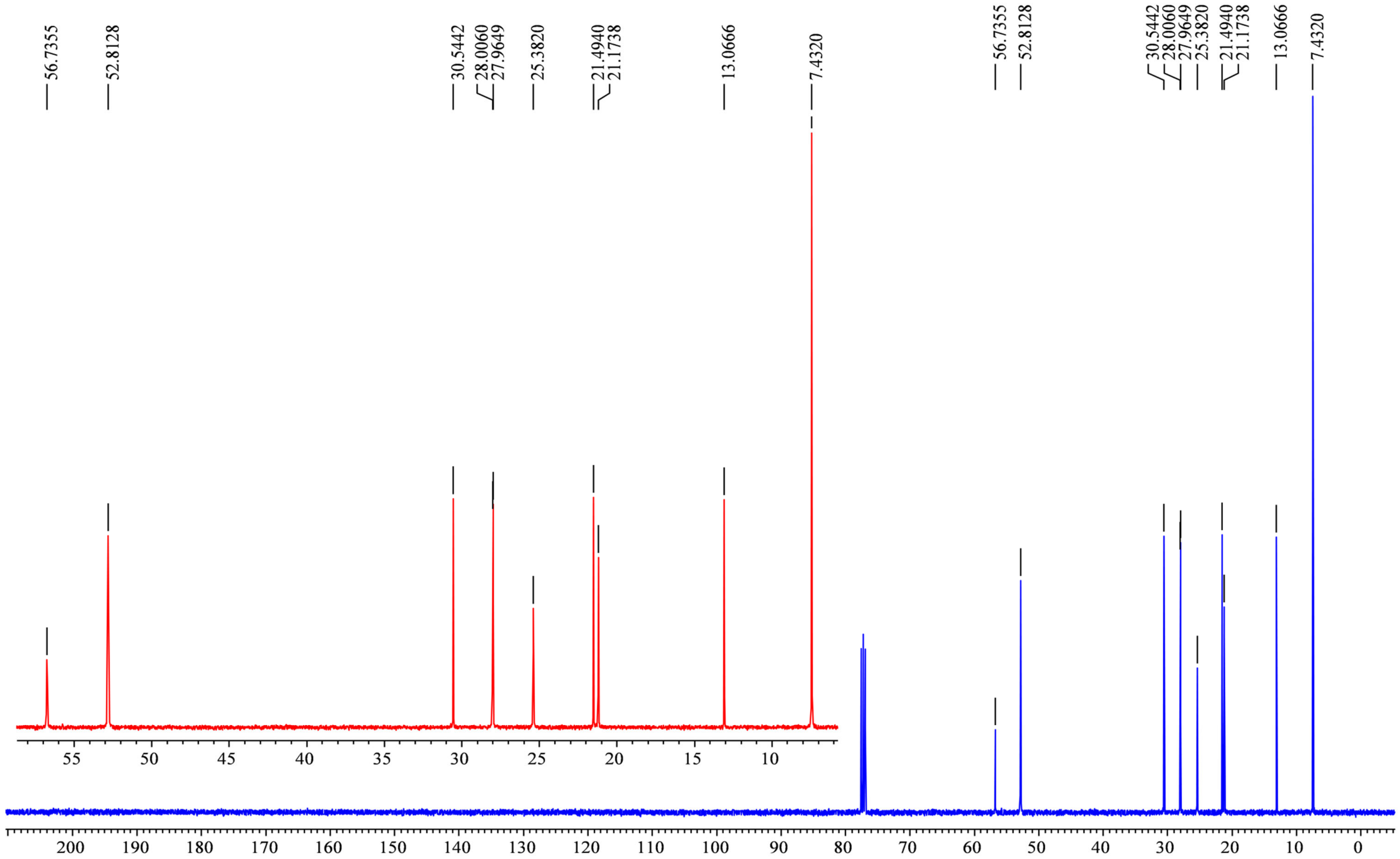

Figure 139S. $^{13}C-\{^{1}H\}$ NMR spectrum (100.6 MHz, $CDCl_3$) of $Et_3N^+–C_8H_{17}\ I^–$ (**7**).

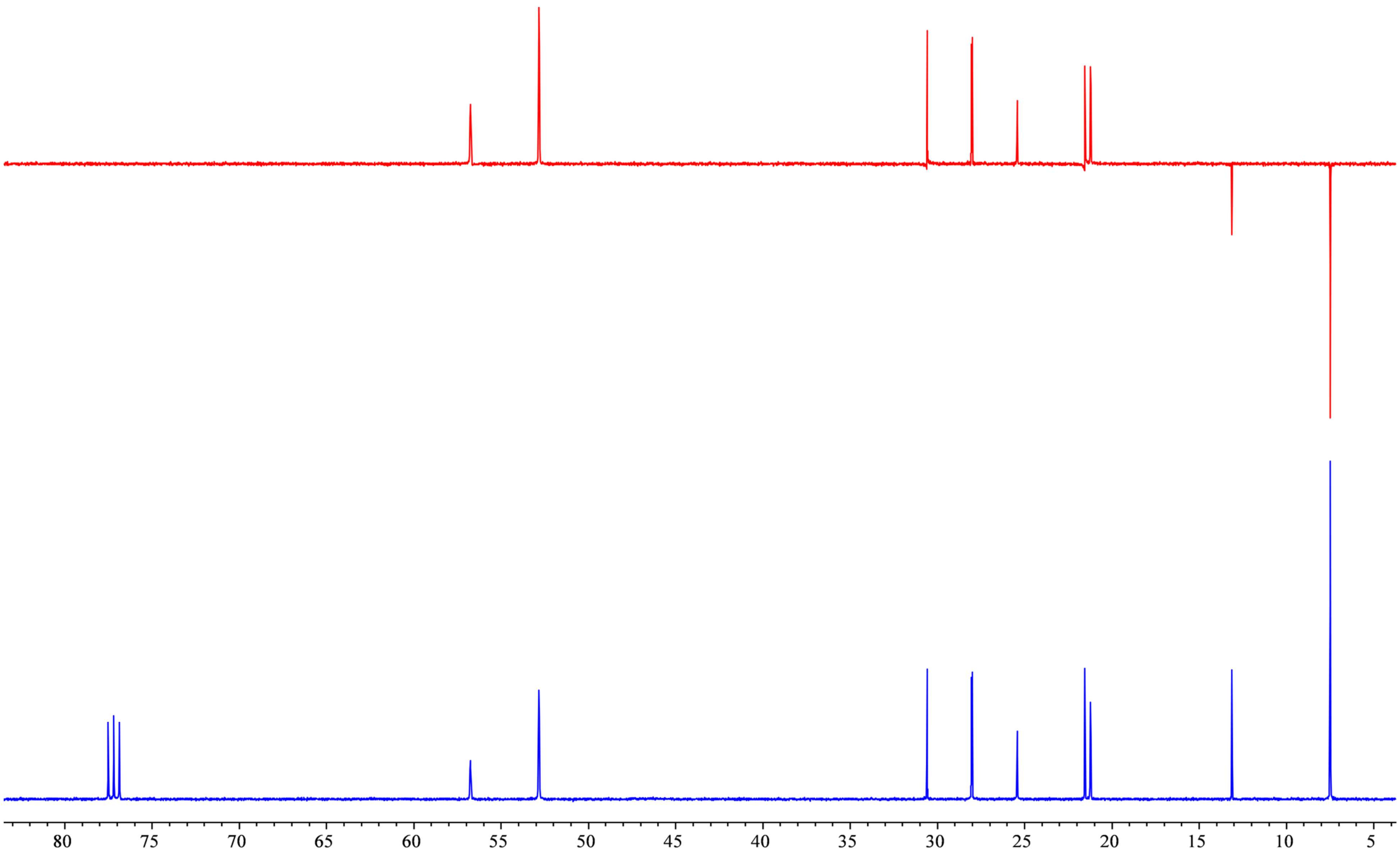

Figure 140S. ^{13}C-{^{1}H} and ^{13}C-{^{1}H}-dept NMR spectra (100.6 MHz, $CDCl_3$) of $Et_3N^+–C_8H_{17}\ I^-$ (**7**).

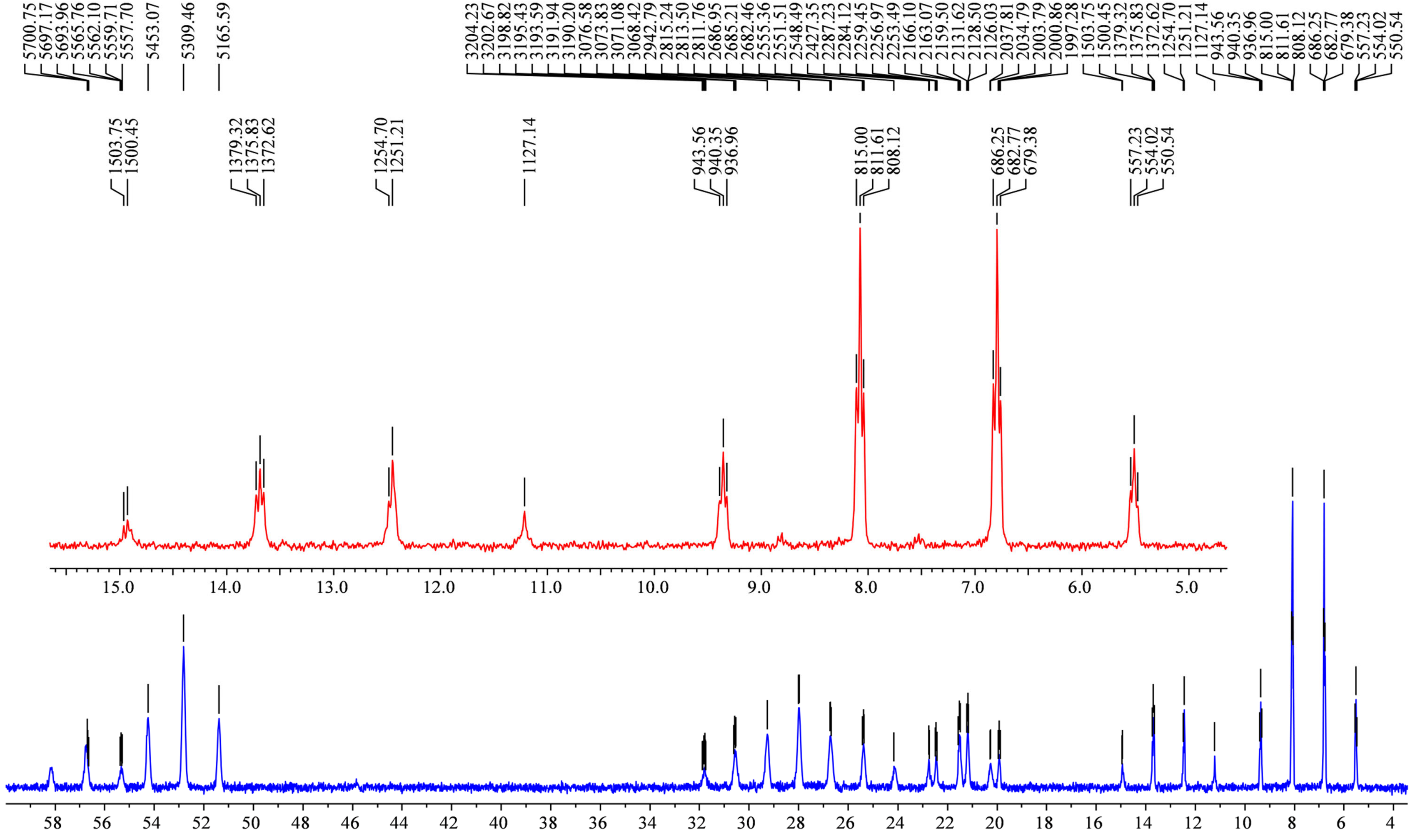

Figure 141S. ^{13}C NMR spectrum (100.6 MHz, $CDCl_3$) of $Et_3N^+–C_8H_{17}$ $I^–$ (**7**).

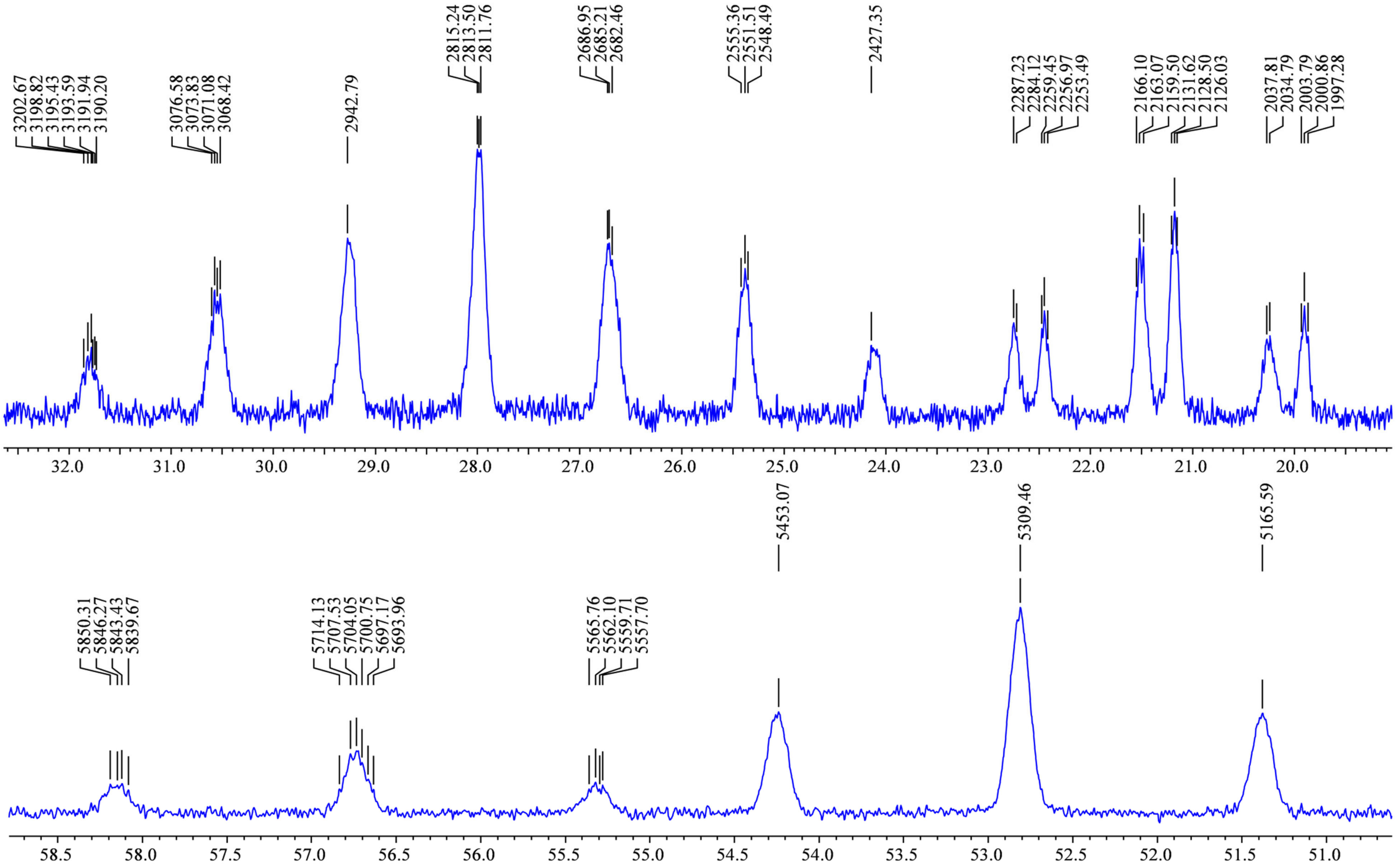

Figure 142S. The 50-59 and 19-33 ppm regions of ^{13}C NMR spectrum (100.6 MHz, $CDCl_3$) of $Et_3N^+–C_8H_{17}\ I^–$ (**7**).

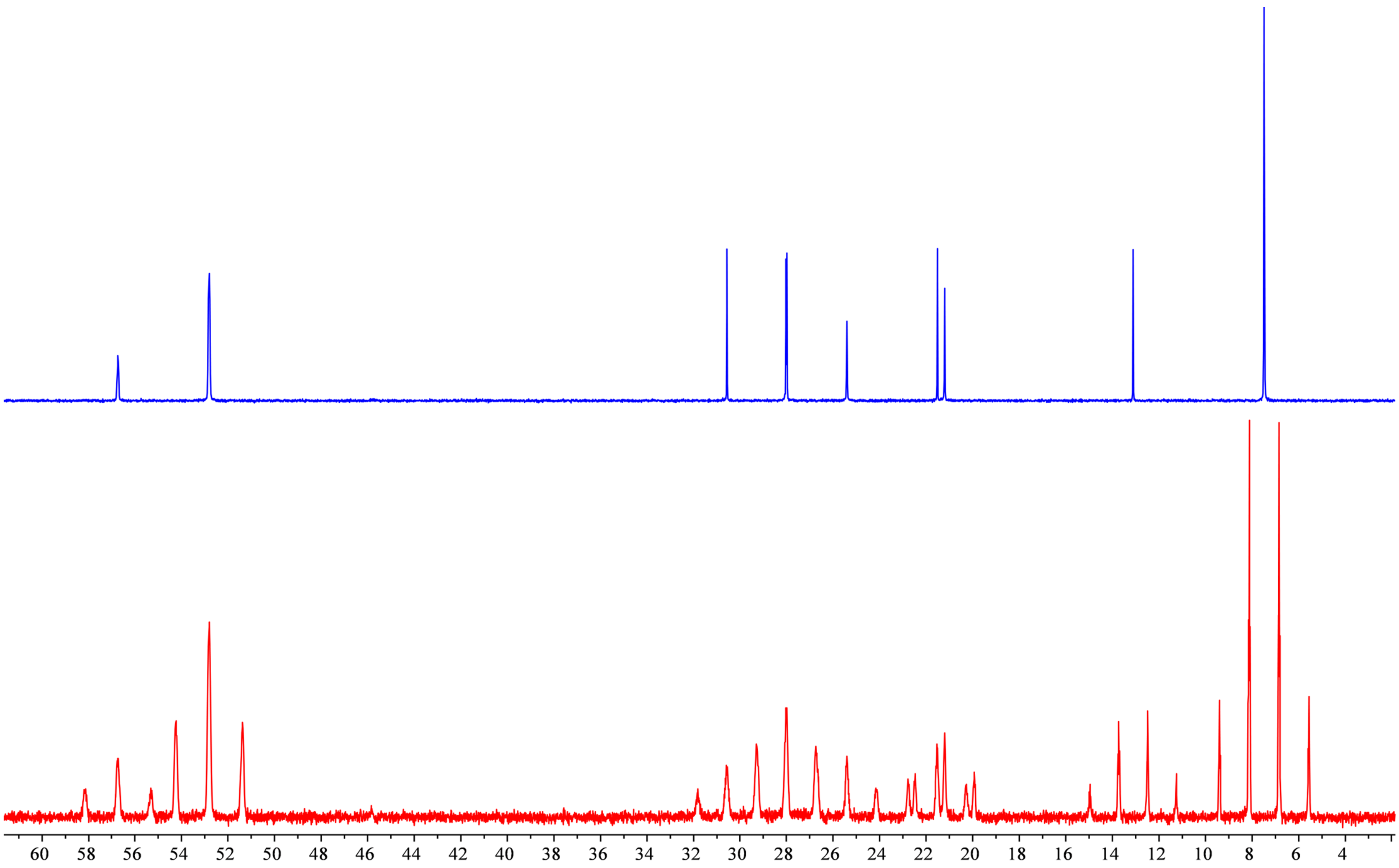

Figure 143S. ^{13}C and ^{13}C-{^{1}H} NMR spectra (100.6 MHz, $CDCl_3$) of Et_3N^+–C_8H_{17} I^- (**7**).

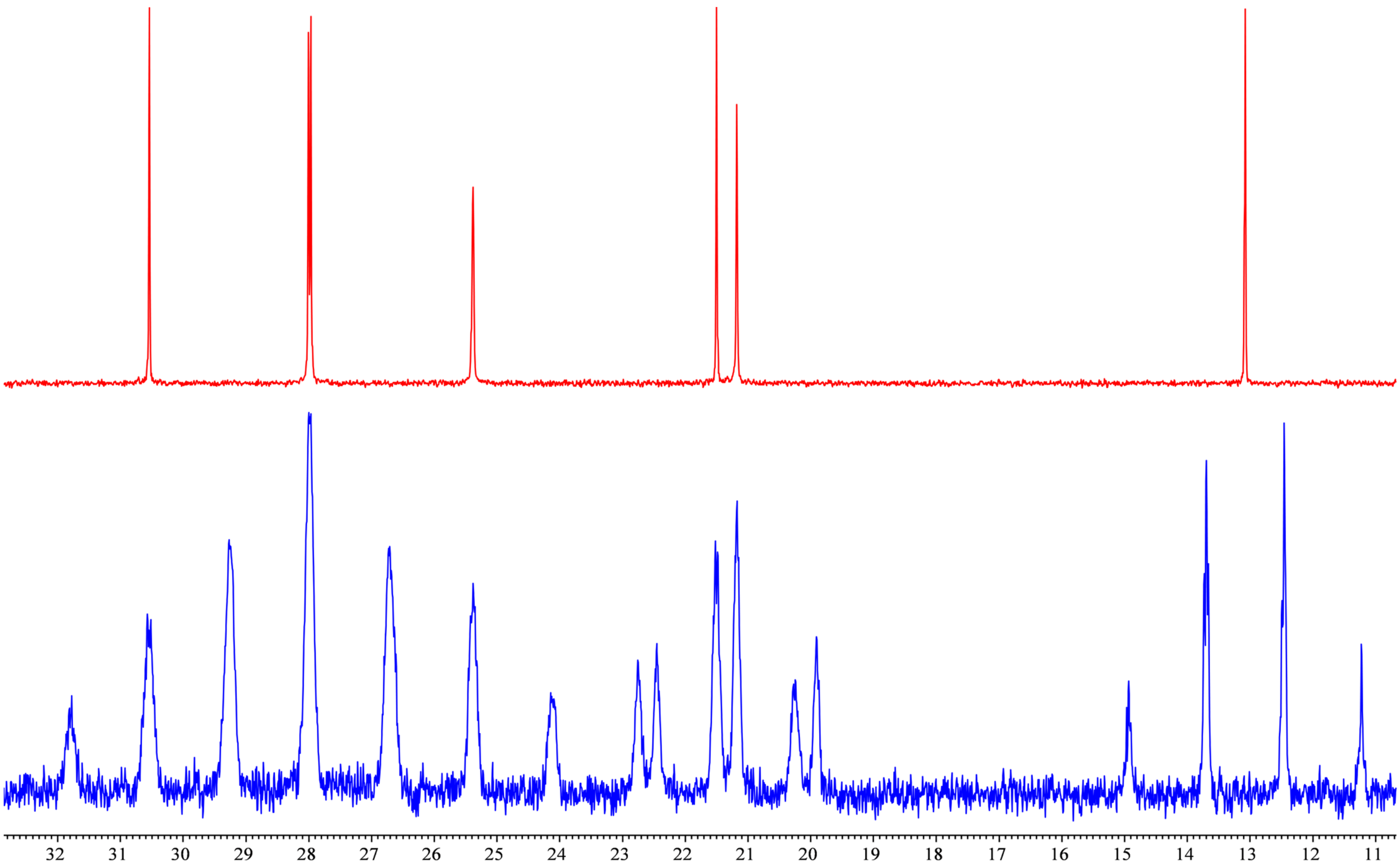

Figure 144S. The 11-32 ppm region of ^{13}C and ^{13}C-{^{1}H} NMR spectra (100.6 MHz, $CDCl_3$) of $Et_3N^+–C_8H_{17}$ $I^–$ (**7**).

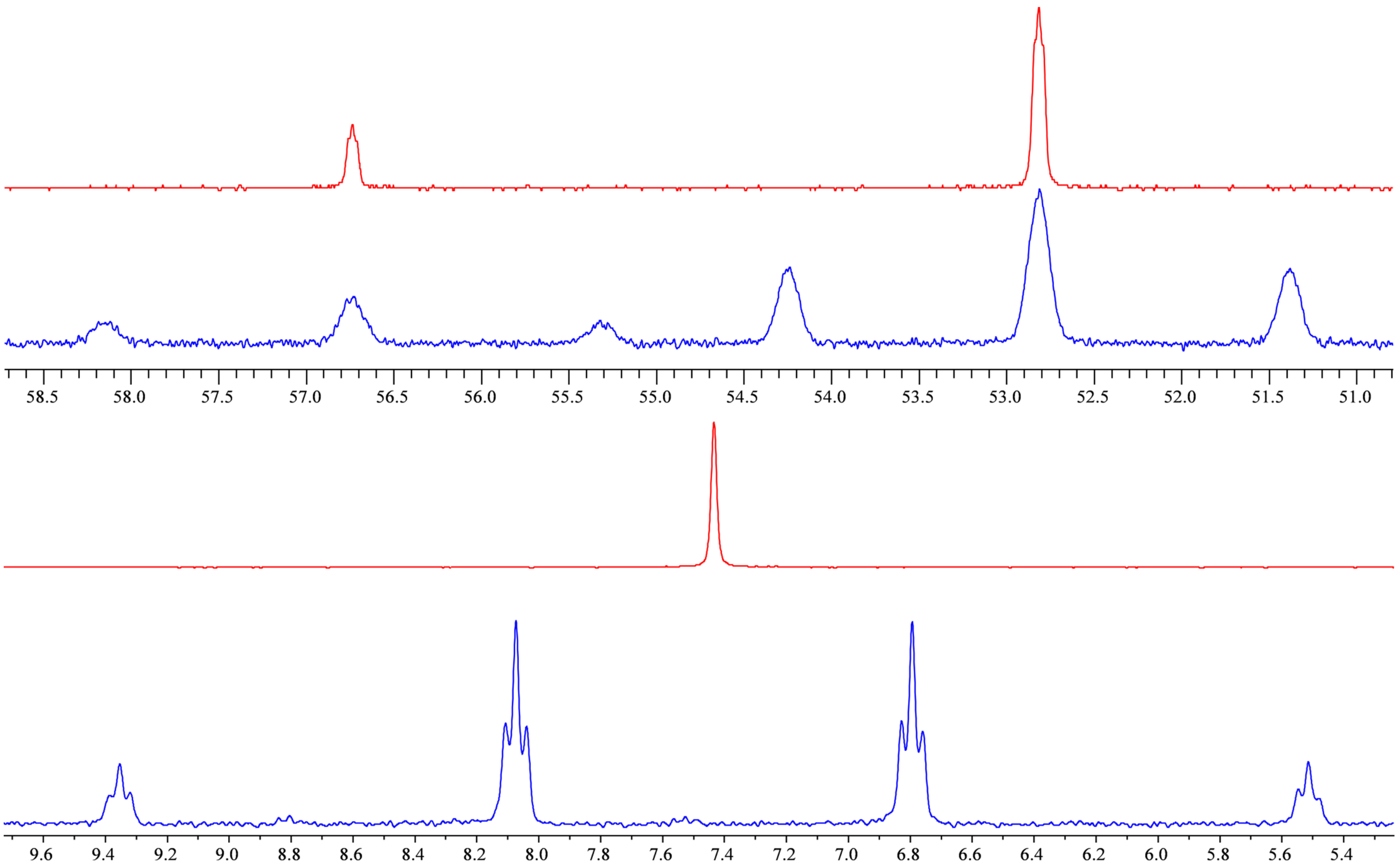

Figure 145S. The 5-10 and 50-59 ppm regions of ^{13}C and ^{13}C-{^{1}H} NMR spectra (100.6 MHz, $CDCl_3$) of $Et_3N^+–C_8H_{17}\ I^–$ (**7**).

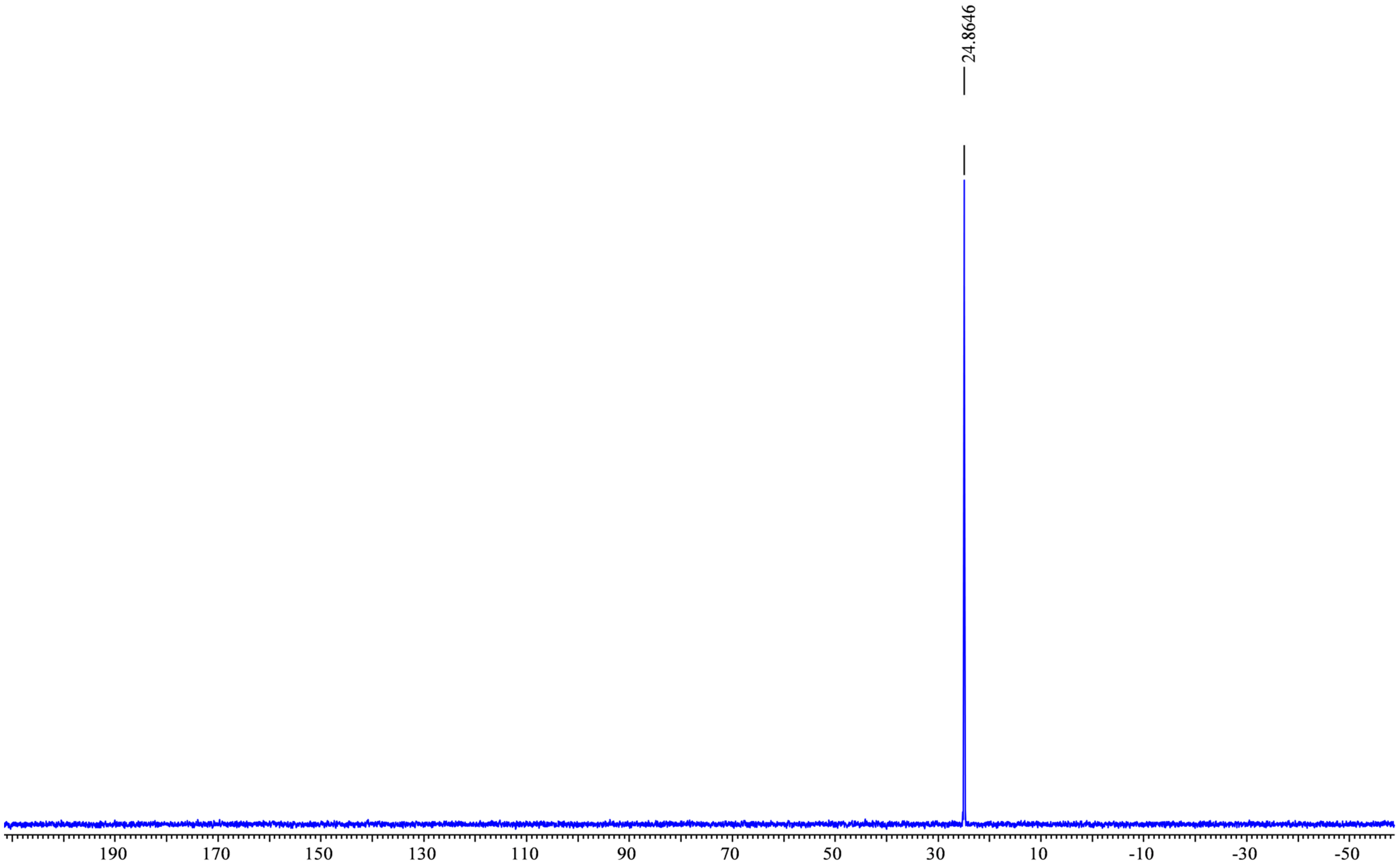

Figure 146S. ^{31}P-{^{1}H} NMR spectrum (162.0 MHz, $CDCl_3$) of $Ph_3P^+–C_8H_{17}$ $I^–$ (**8**).

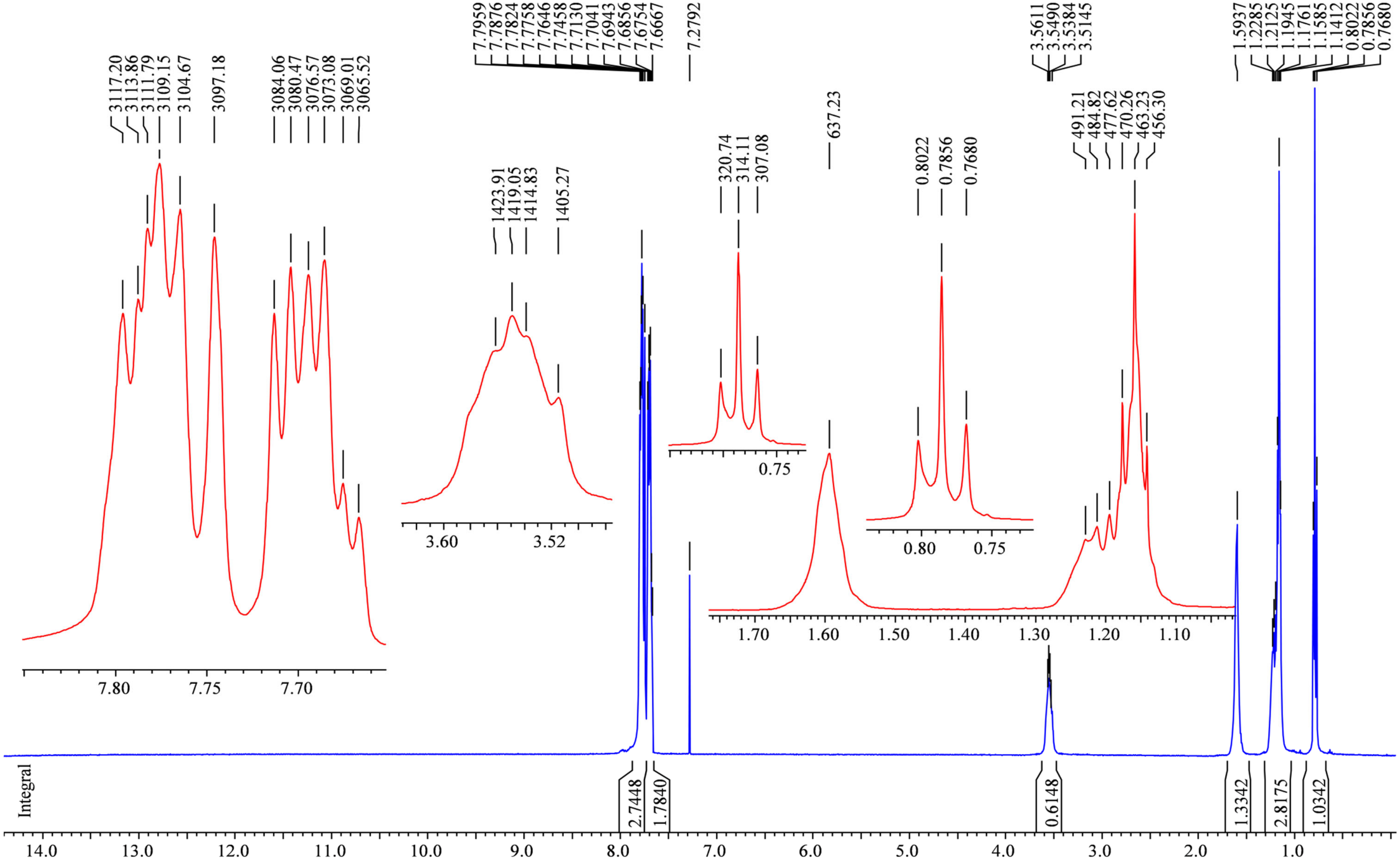

Figure 147S. ^{1}H NMR spectrum (400.0 MHz, $CDCl_3$) of Ph_3P^+–C_8H_{17} I^- (**8**).

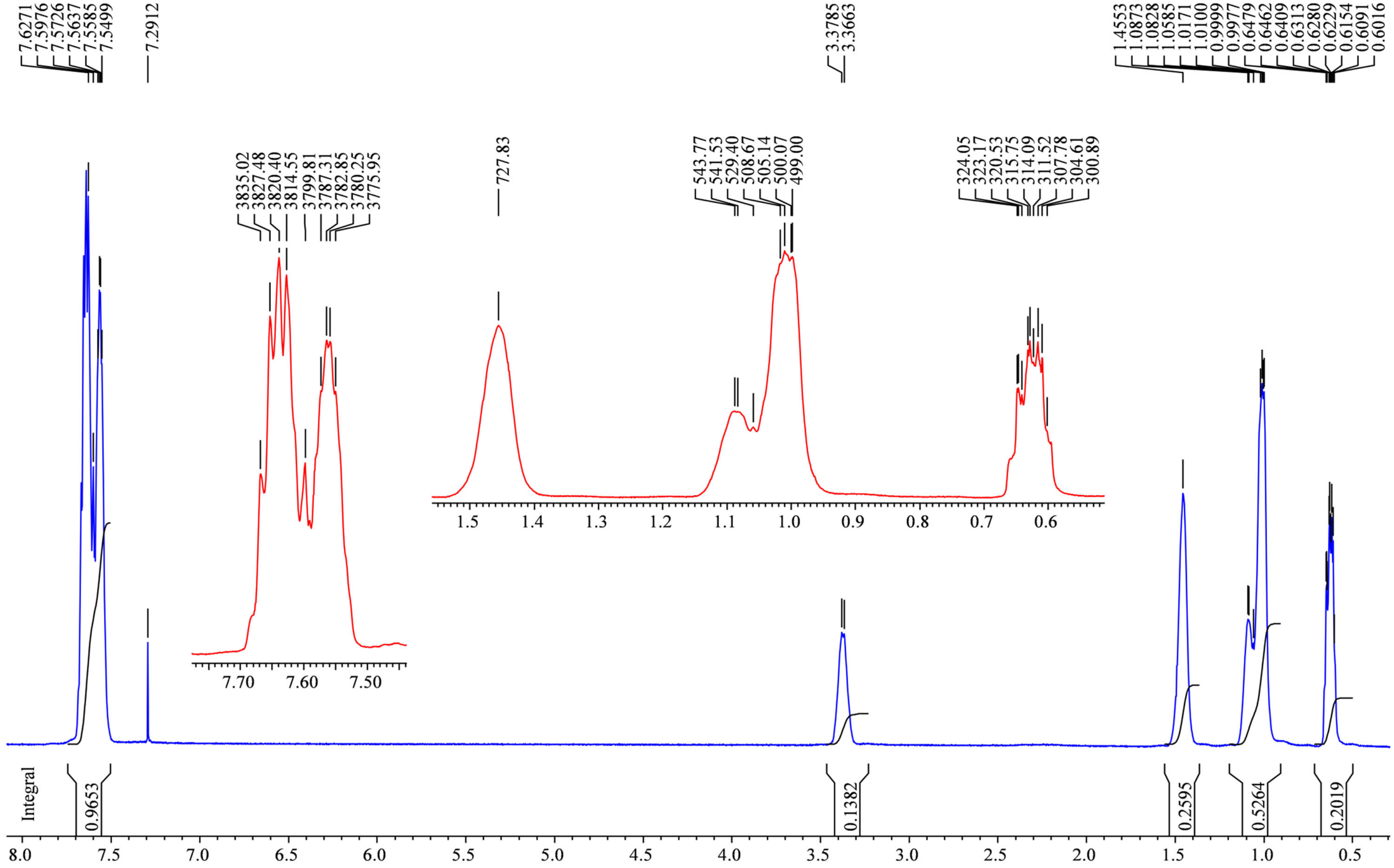

Figure 148S. ^{1}H NMR spectrum (500.0 MHz, $CDCl_3$) of $Ph_3P^+–C_8H_{17}\ I^-$ (**8**).

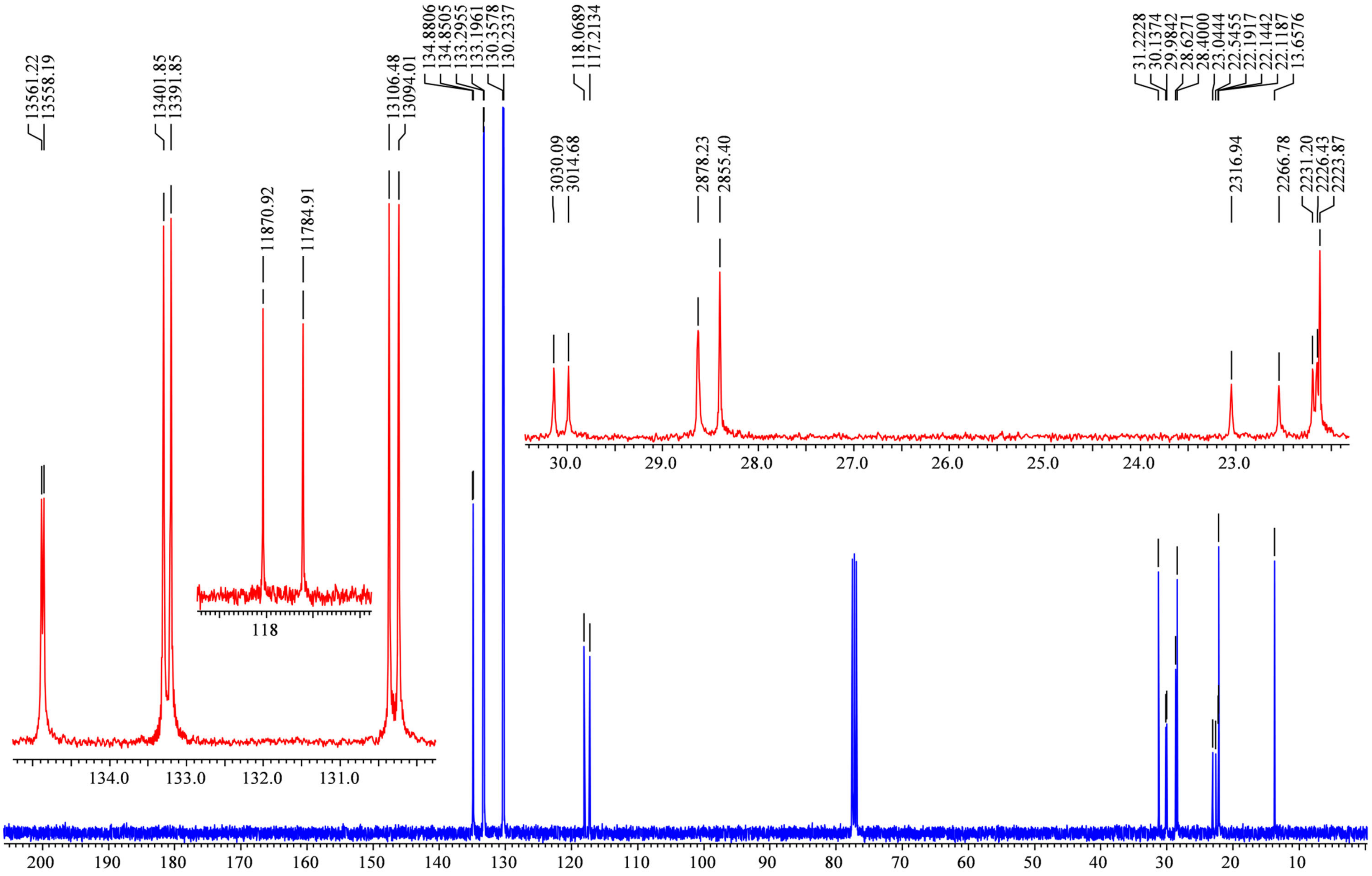

Figure 149S. ^{13}C-{^{1}H} NMR spectrum (100.6 MHz, $CDCl_3$) of $Ph_3P^+–C_8H_{17}\ I^–$ (**8**).

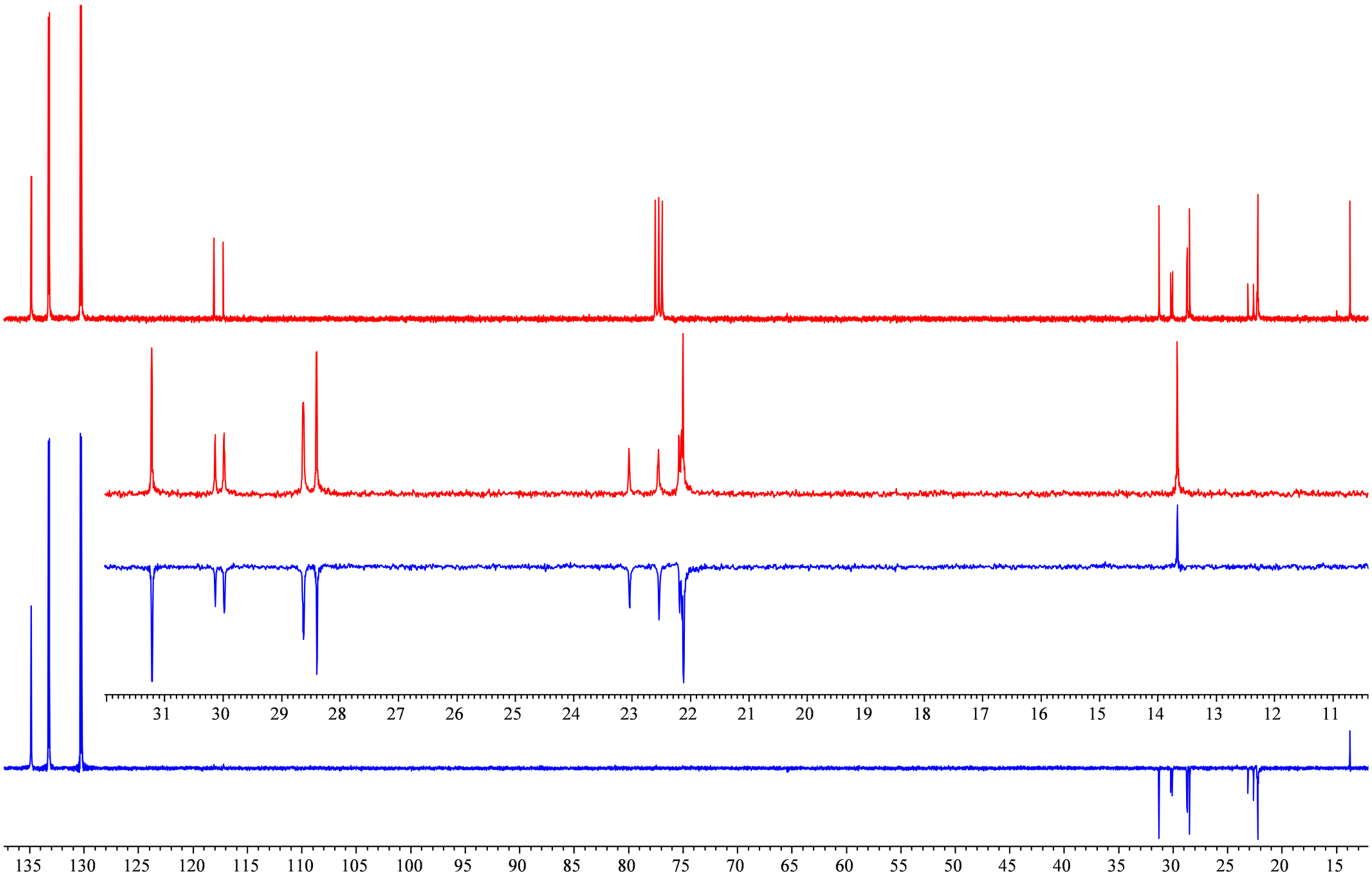

Figure 150S. ^{13}C-{^{1}H} and ^{13}C-{^{1}H}-dept NMR spectra (100.6 MHz, $CDCl_3$) of $Ph_3P^+–C_8H_{17}\ I^-$ (**8**).

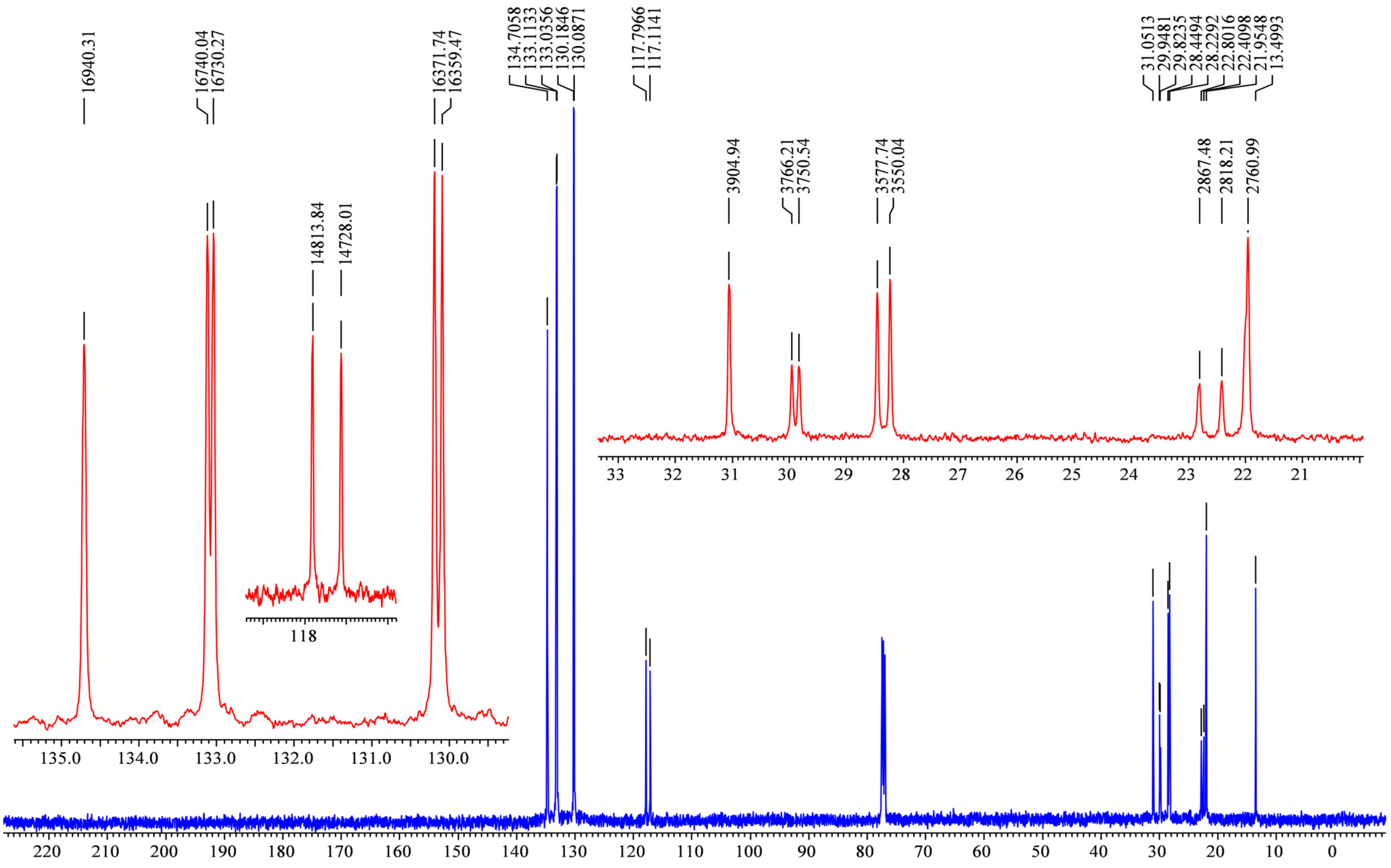

Figure 151S. ^{13}C-{^{1}H} NMR spectrum (125.8 MHz, $CDCl_3$) of $Ph_3P^+–C_8H_{17}\ I^−$ (**8**).

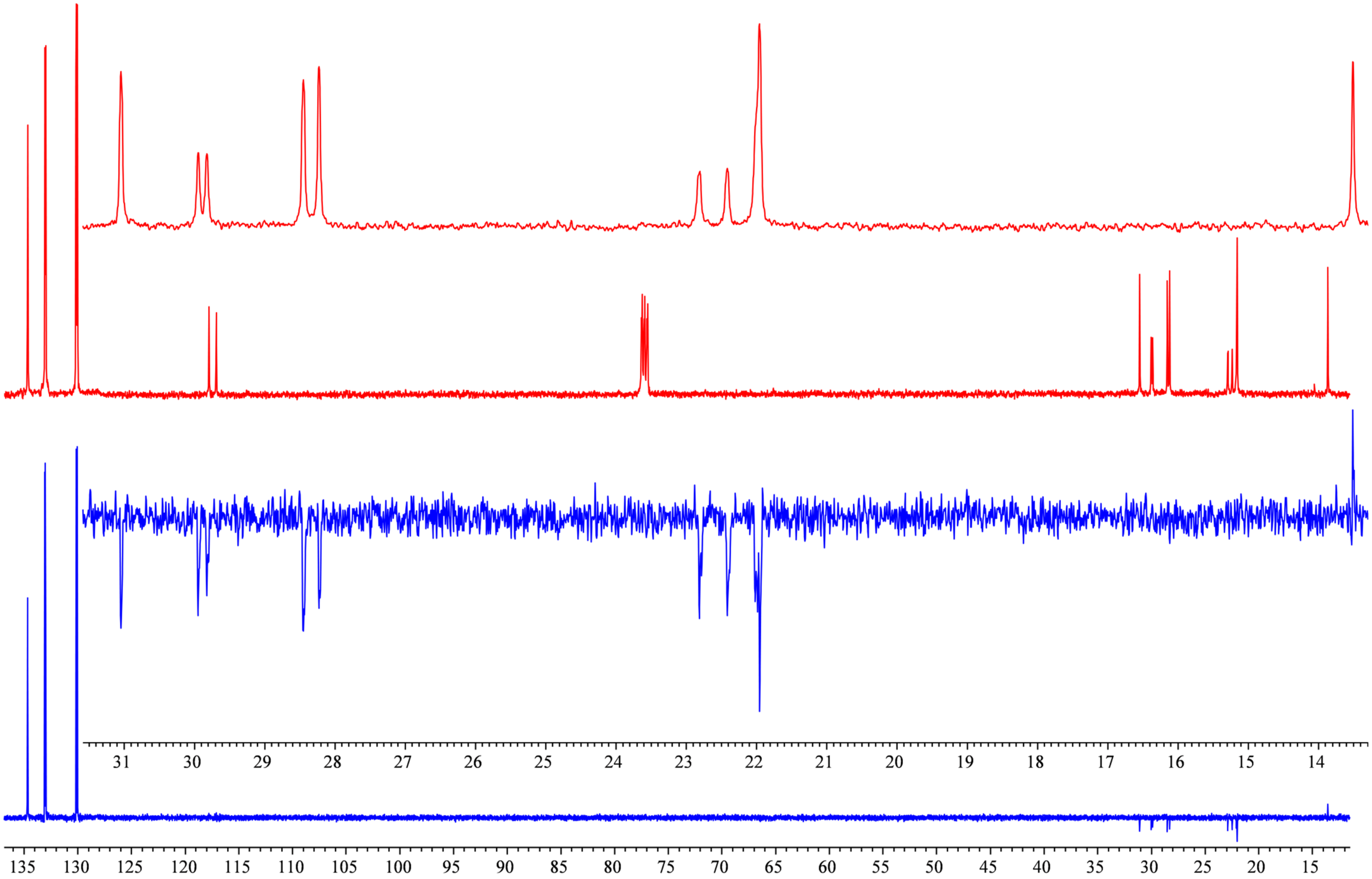

Figure 152S. ^{13}C-{^{1}H} and ^{13}C-{^{1}H}-dept NMR spectra (125.8 MHz, $CDCl_3$) of $Ph_3P^+–C_8H_{17}\ I^-$ (**8**).

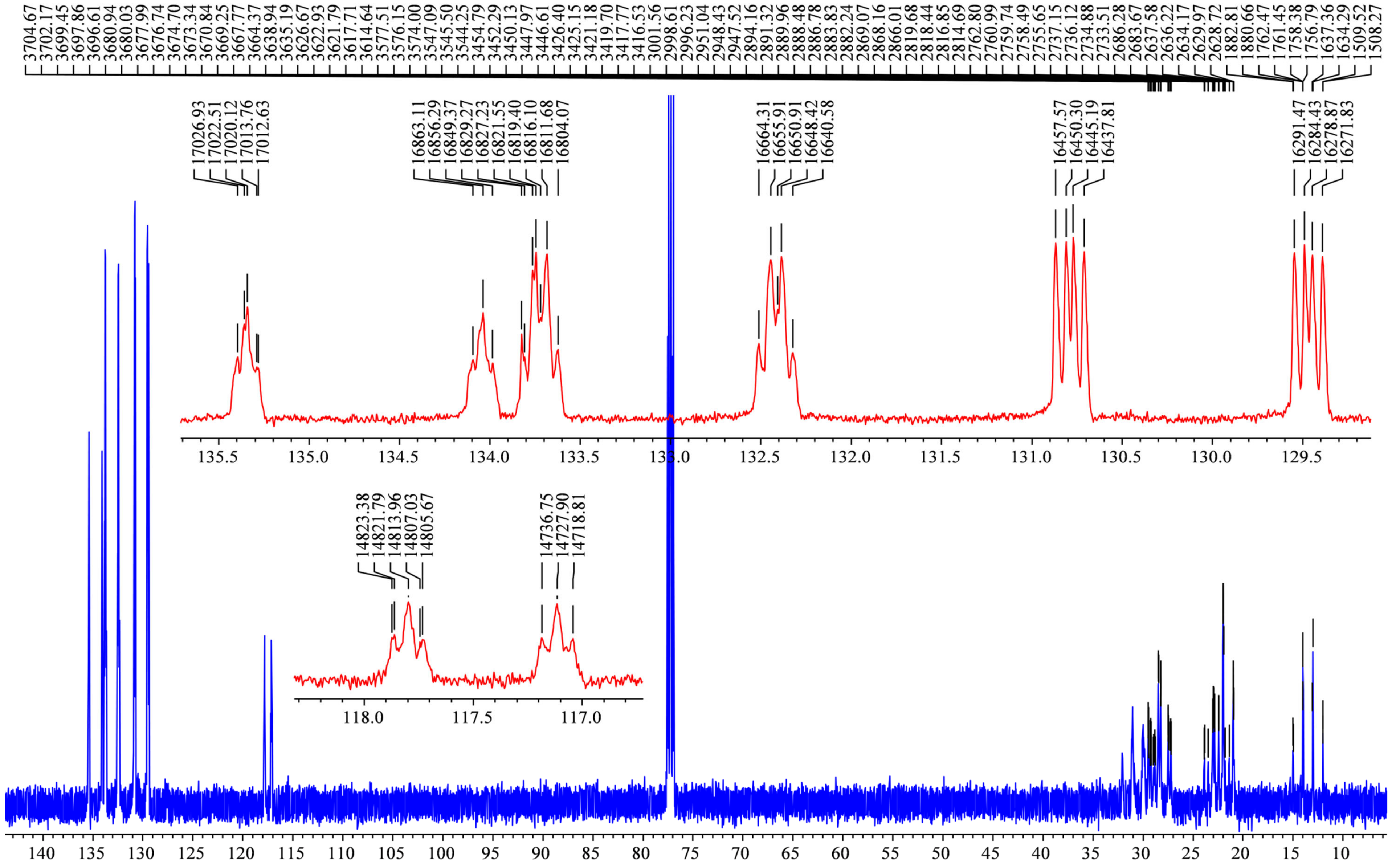

Figure 153S. ^{13}C NMR spectrum (125.8 MHz, $CDCl_3$) of Ph_3P^+–C_8H_{17} I^- (**8**).

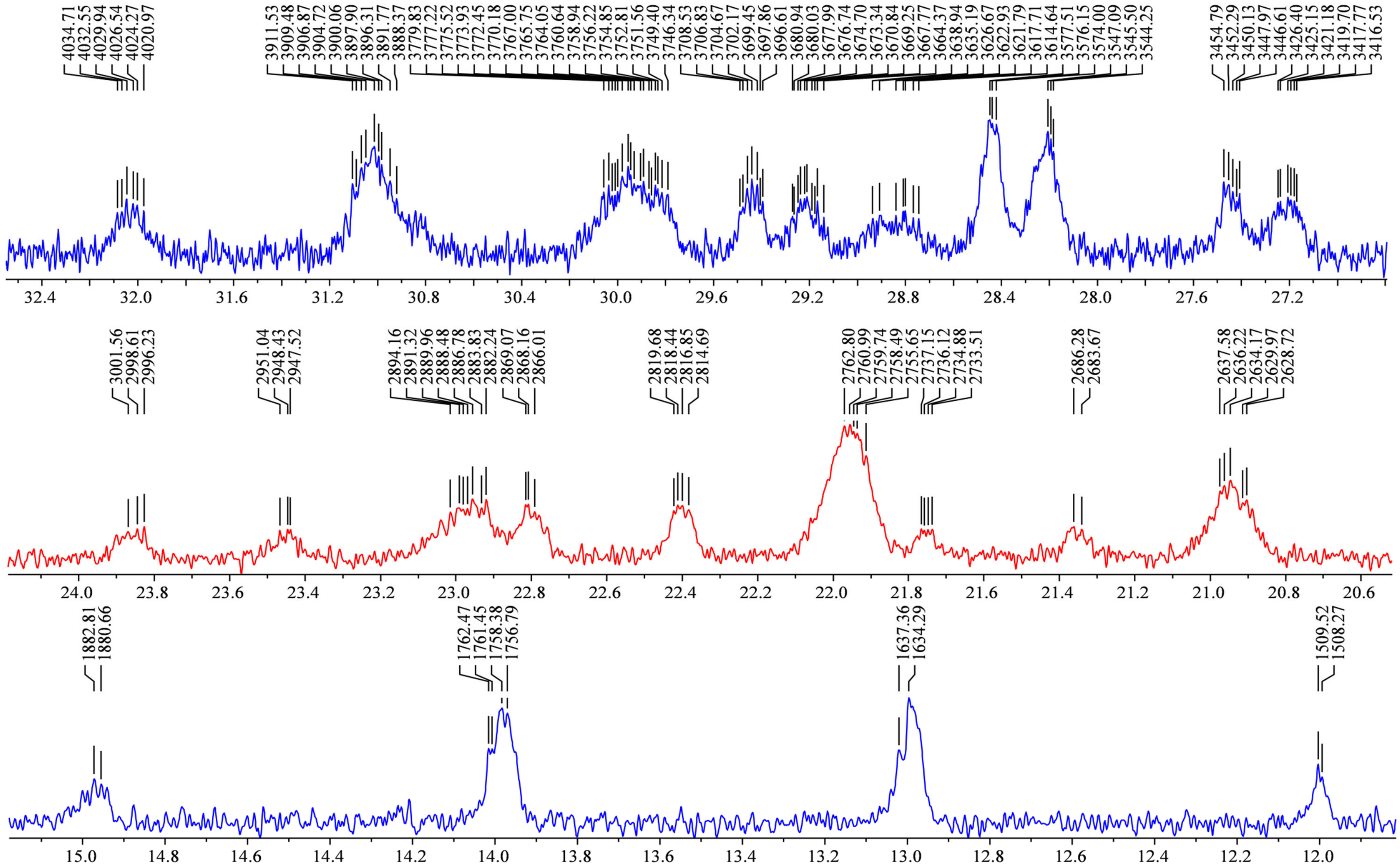

Figure 154S. High-field fragments of ^{13}C NMR spectrum (125.8 MHz, $CDCl_3$) of $Ph_3P^+–C_8H_{17}\ I^–$ (**8**).

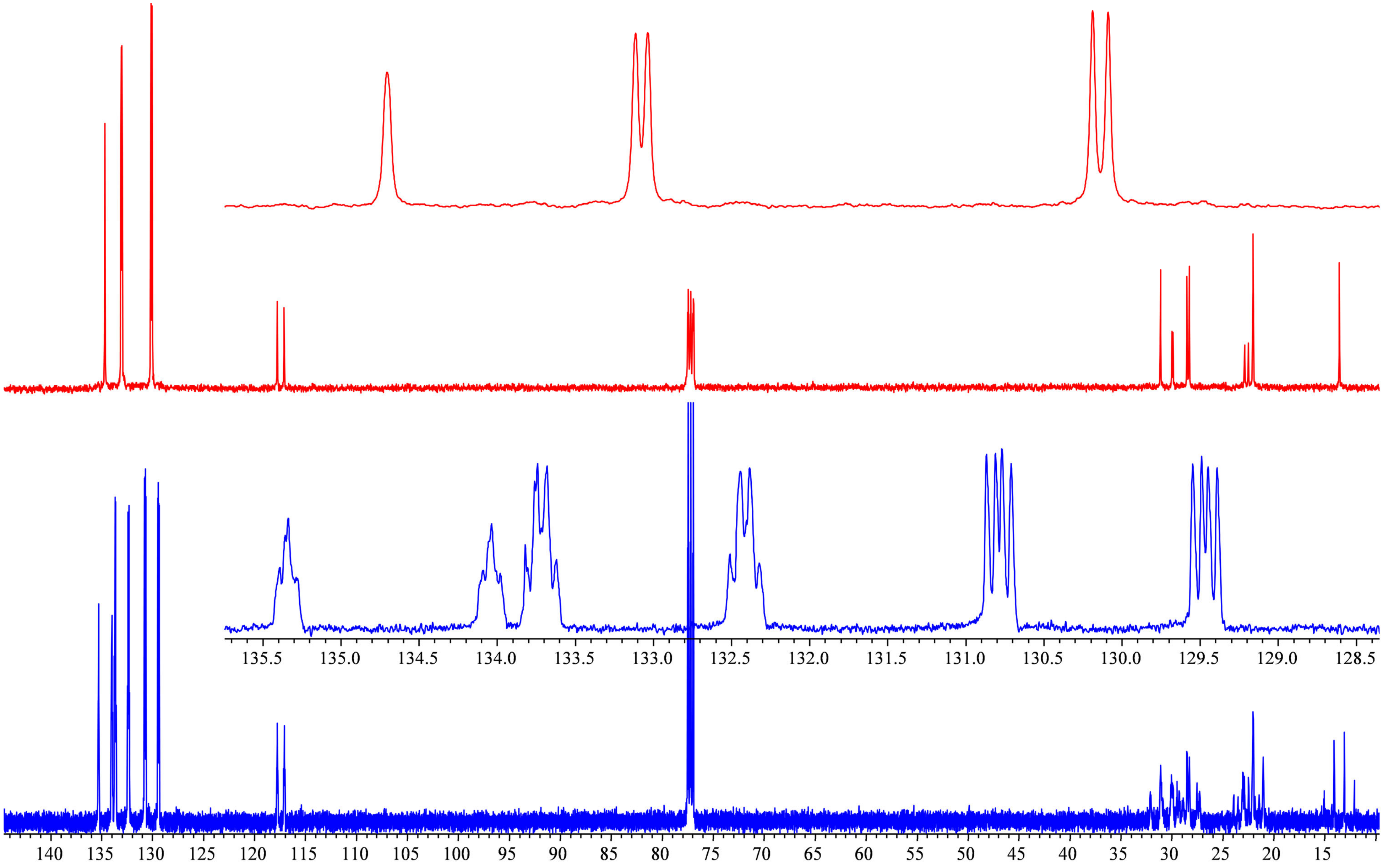

Figure 155S. ^{13}C and ^{13}C-{^{1}H} NMR spectra (125.8 MHz, $CDCl_3$) of Ph_3P^+–C_8H_{17} I^- (**8**).

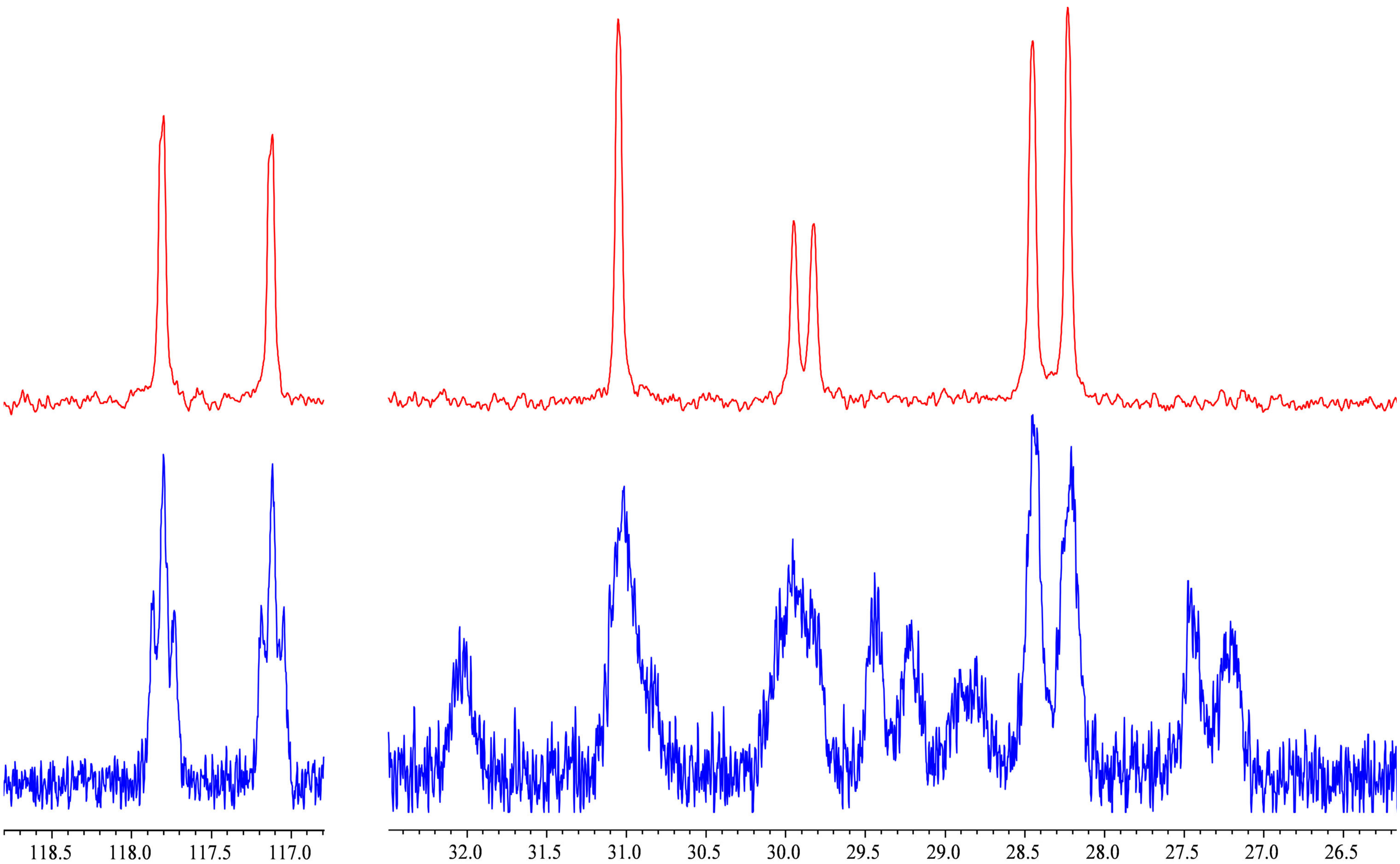

Figure 156S. The 117-119 and 26-33 ppm regions of ^{13}C and ^{13}C-{^{1}H} NMR spectra (125.8 MHz, $CDCl_3$) of $Ph_3P^+–C_8H_{17}$ $I^–$ (**8**).

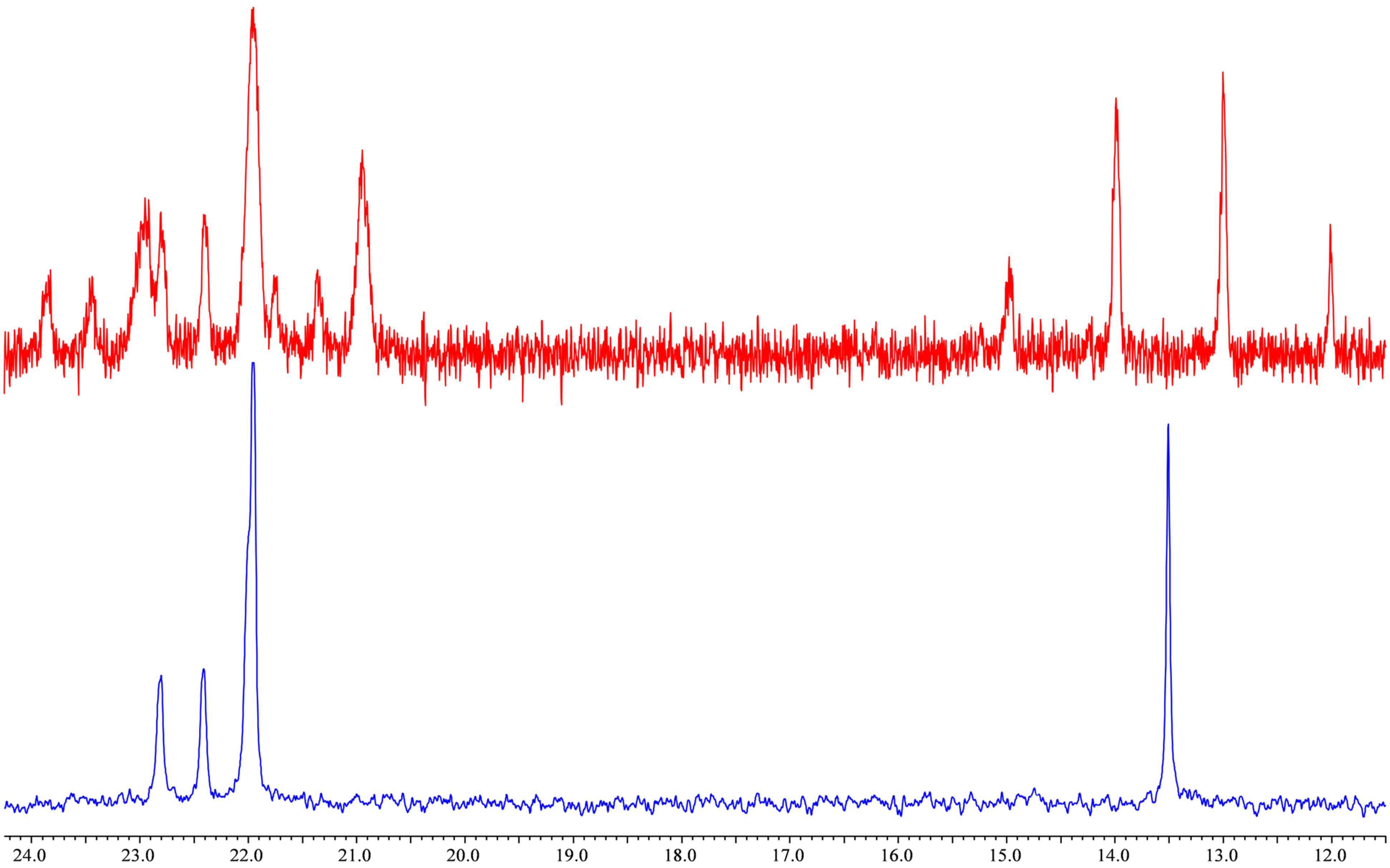

Figure 157S. High-field fragment of ^{13}C and ^{13}C-{^{1}H} NMR spectra (125.8 MHz, $CDCl_3$) of $Ph_3P^+–C_8H_{17}$ $I^–$ (**8**).

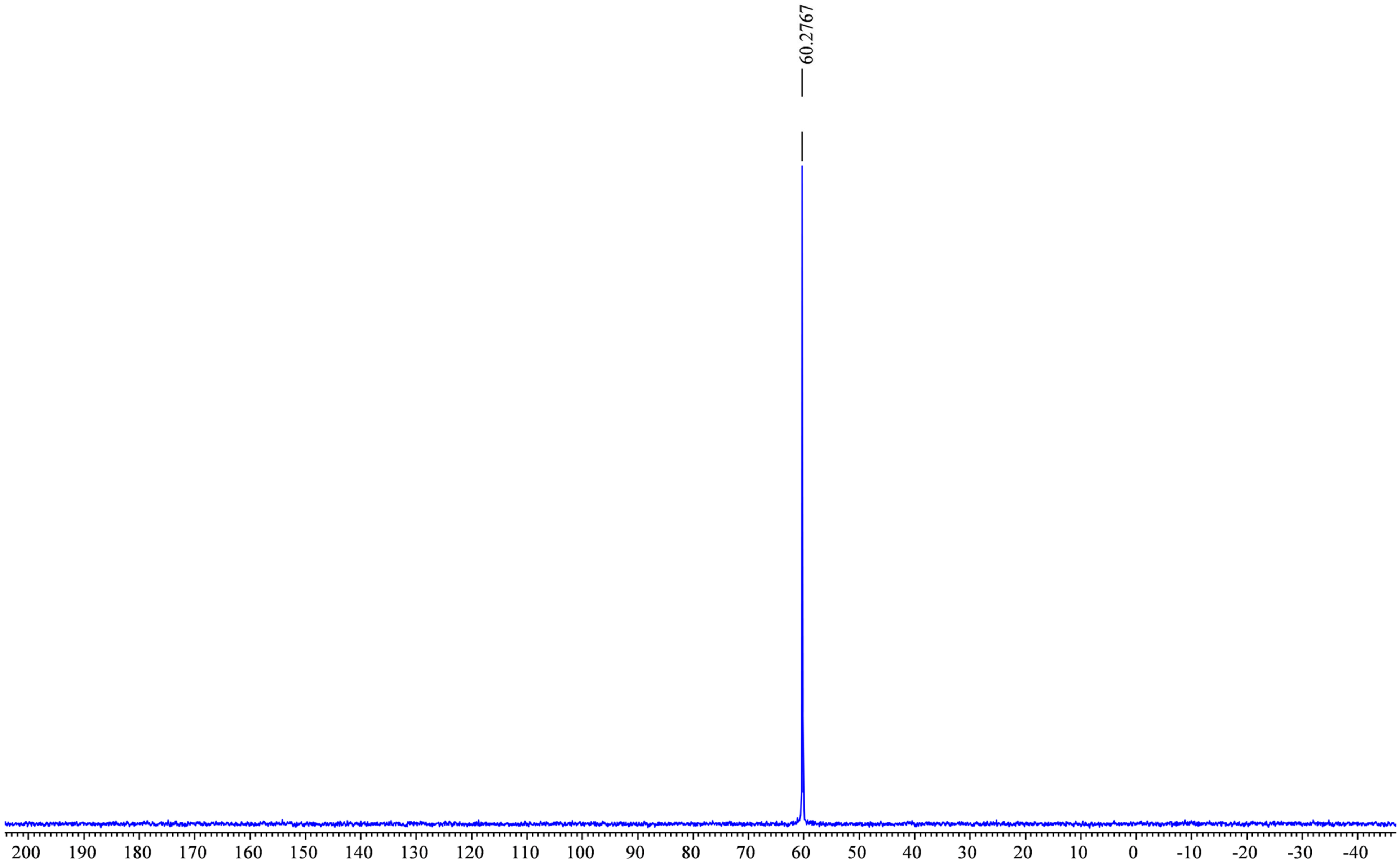

Figure 158S. ^{31}P-{^{1}H} NMR spectrum (162.0 MHz, $CDCl_3$) of $(Et_2N)_3P^+–C_6H_{12}–P^+(NEt_2)_3$ $2Br^–$ (**9**).

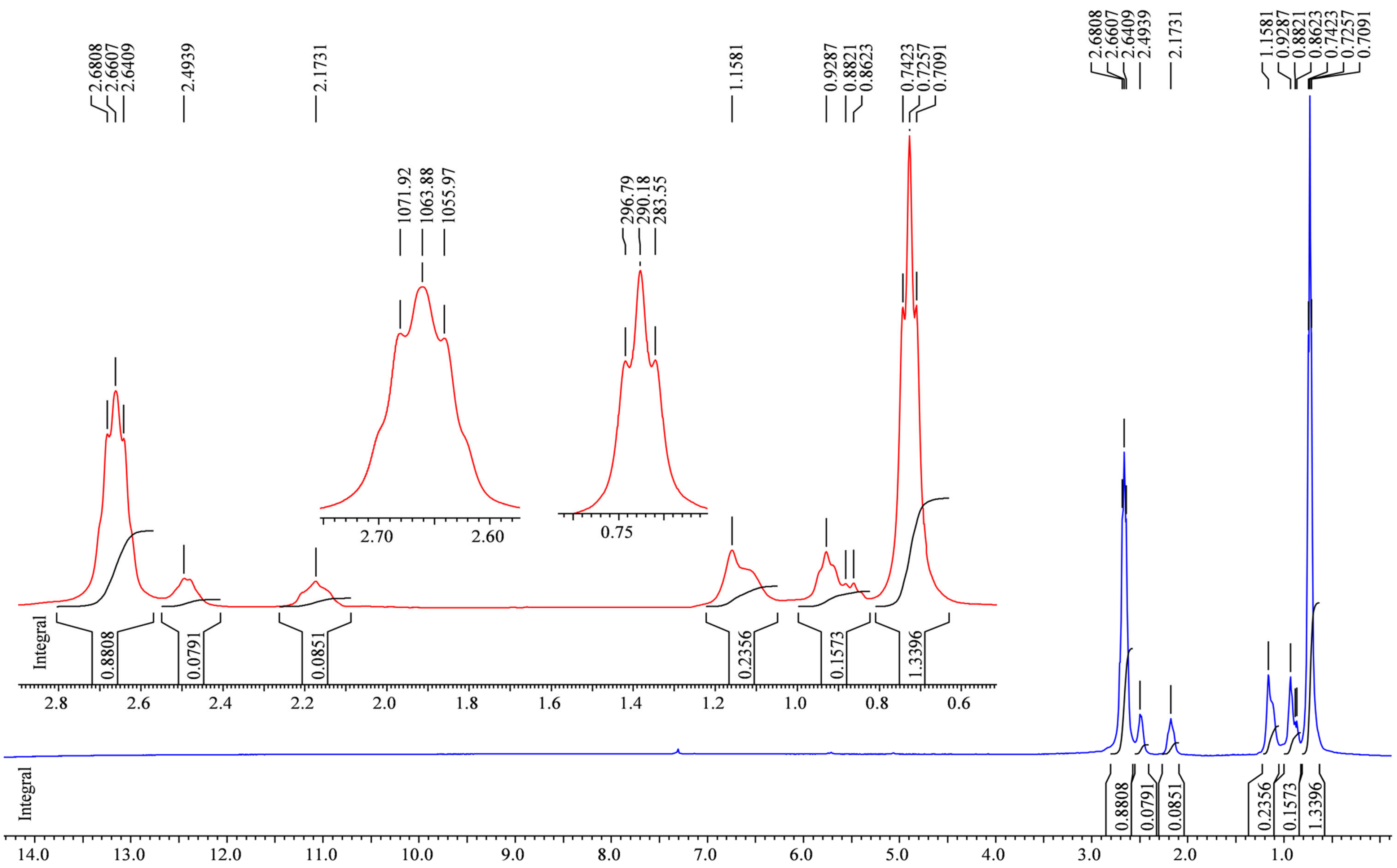

Figure 159S. ^{1}H NMR spectrum (400.0 MHz, $CDCl_3$) of $(Et_2N)_3P^+–C_6H_{12}–P^+(NEt_2)_3$ $2Br^−$ (**9**).

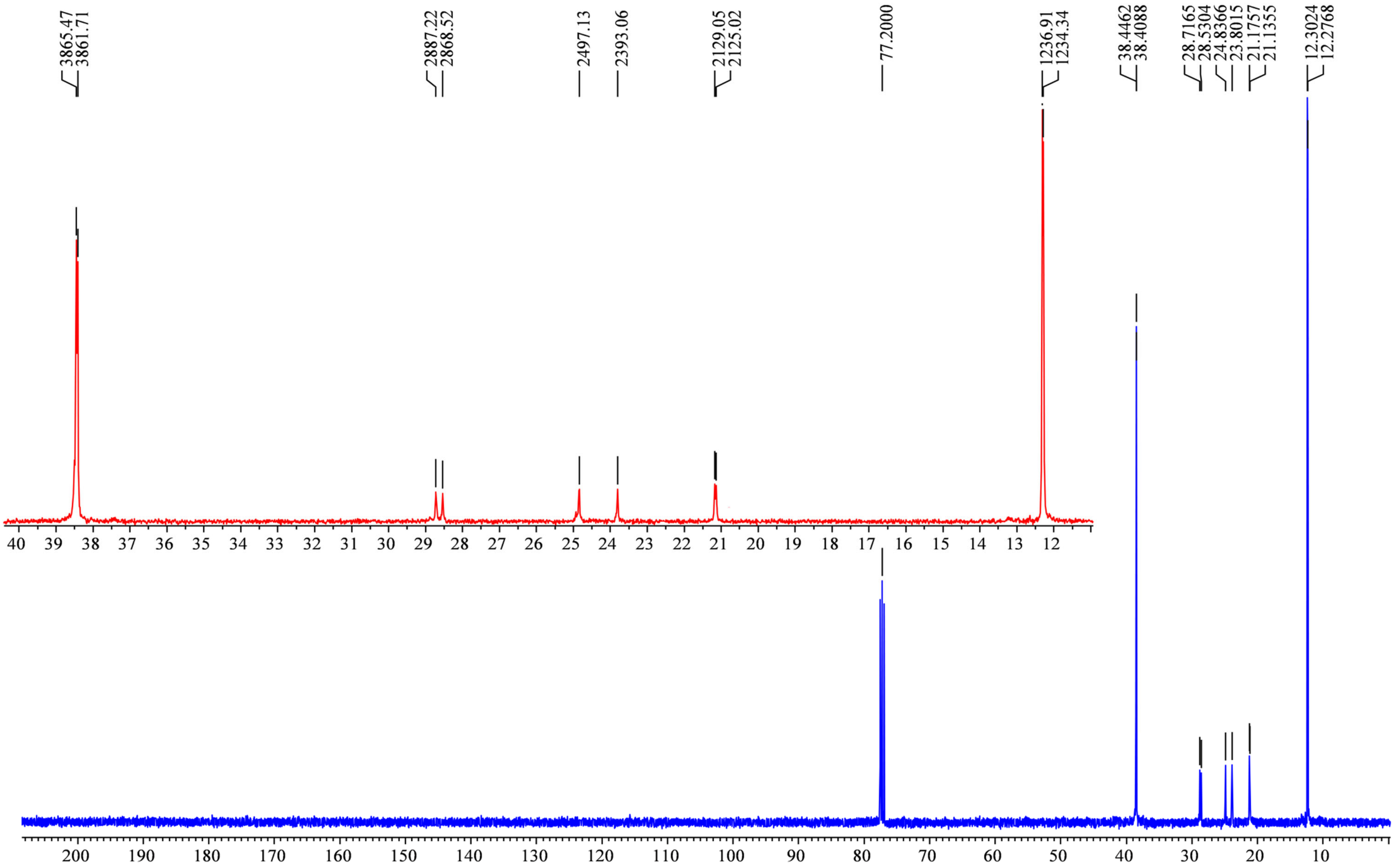

Figure 160S. ^{13}C-$\{^1H\}$ NMR spectrum (100.6 MHz, $CDCl_3$) of $(Et_2N)_3P^+–C_6H_{12}–P^+(NEt_2)_3$ $2Br^–$ (**9**).

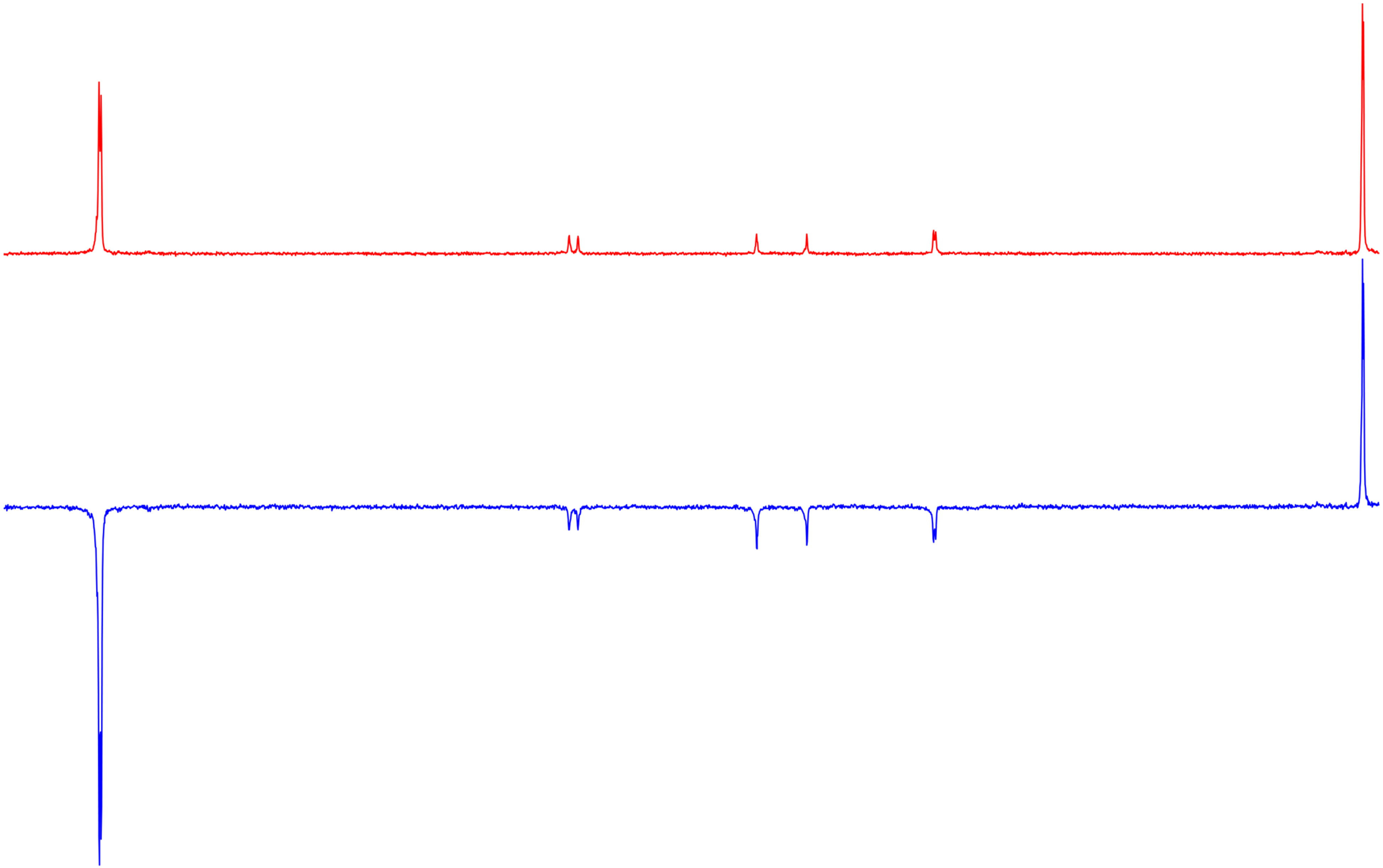

Figure 161S. ^{13}C-{^{1}H} and ^{13}C-{^{1}H}-dept NMR spectra (100.6 MHz, $CDCl_3$) of $(Et_2N)_3P^{+}–C_6H_{12}–P^{+}(NEt_2)_3$ $2Br^{-}$ (**9**).

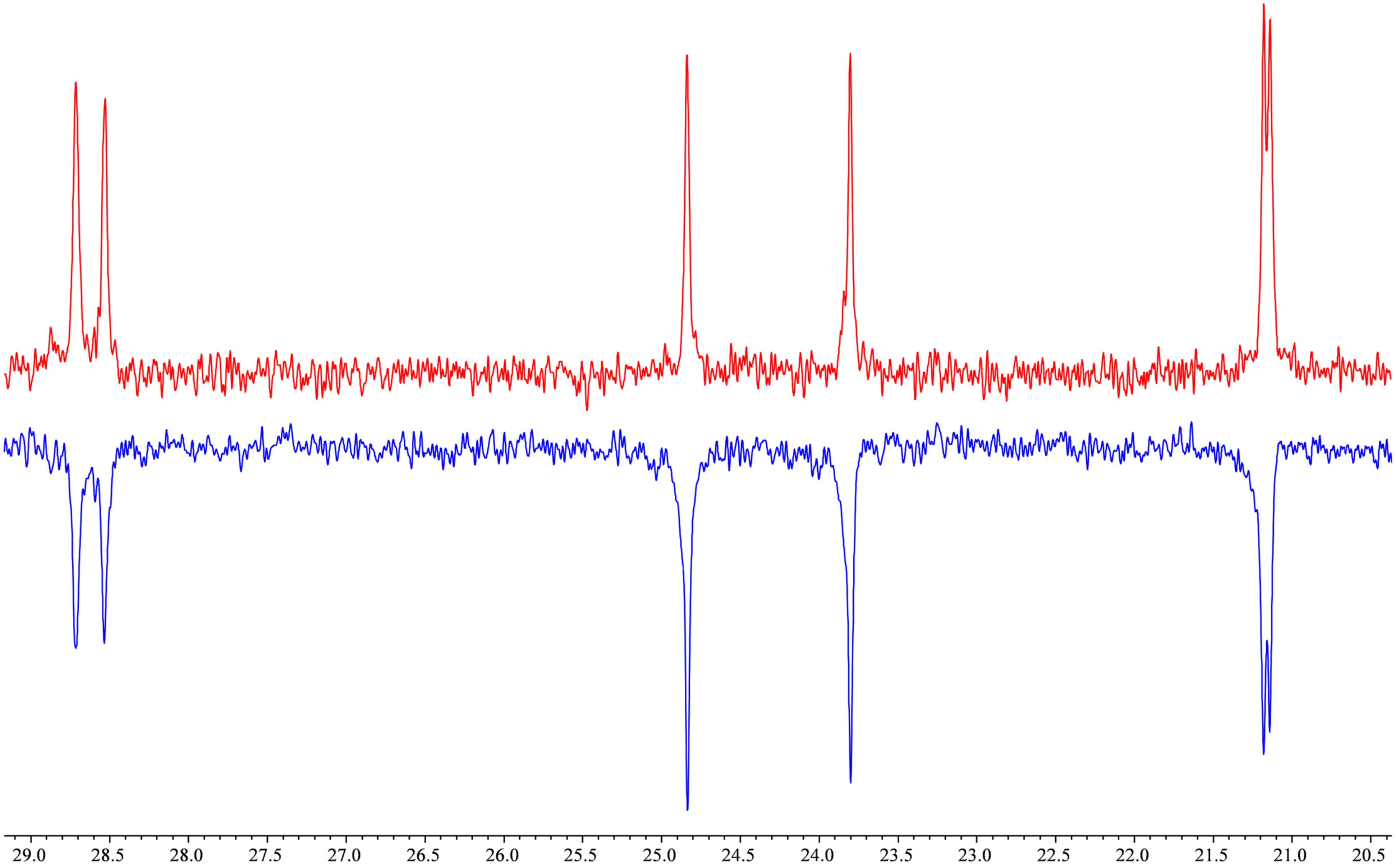

Figure 162S. High-field fragment of ^{13}C-{^{1}H} and ^{13}C-{^{1}H}-dept NMR spectra (100.6 MHz, $CDCl_3$) of $(Et_2N)_3P^+–C_6H_{12}–P^+(NEt_2)_3$ $2Br^-$ (**9**).

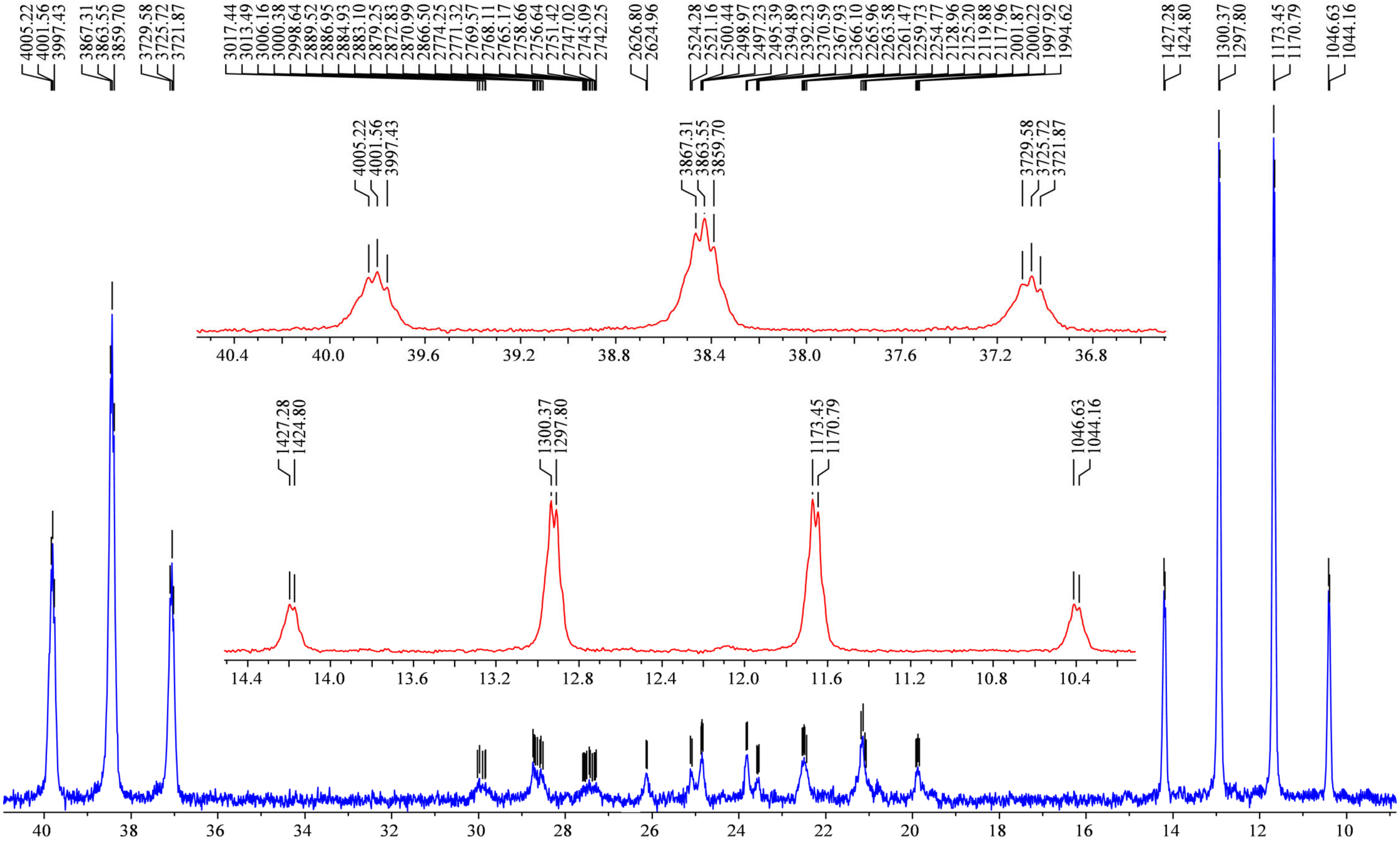

Figure 163S. ^{13}C NMR spectrum (100.6 MHz, $CDCl_3$) of $(Et_2N)_3P^+–C_6H_{12}–P^+(NEt_2)_3$ $2Br^–$ (**9**).

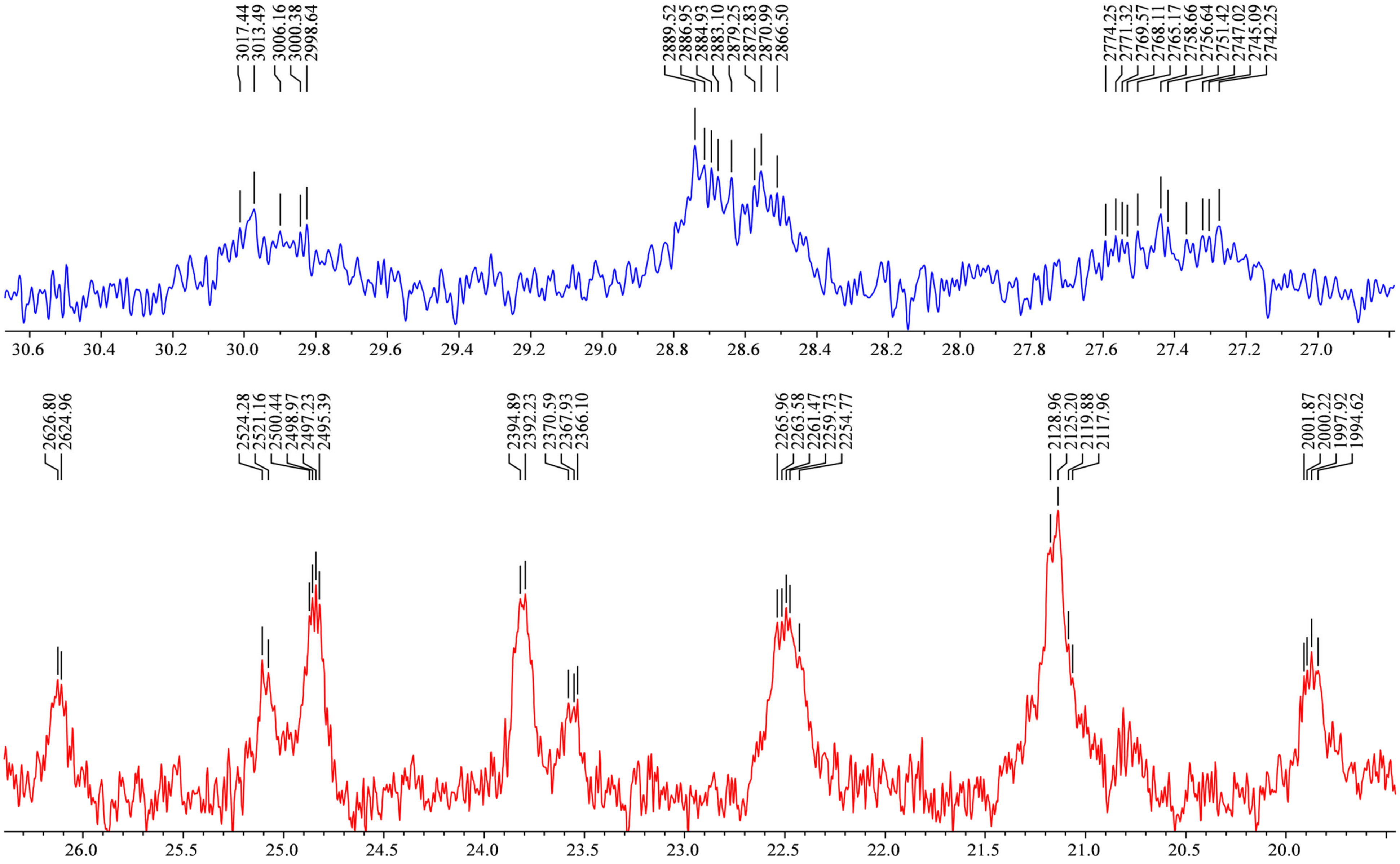

Figure 164S. High-field fragments of ^{13}C NMR spectrum (100.6 MHz, $CDCl_3$) of $(Et_2N)_3P^+–C_6H_{12}–P^+(NEt_2)_3$ $2Br^–$ (**9**).

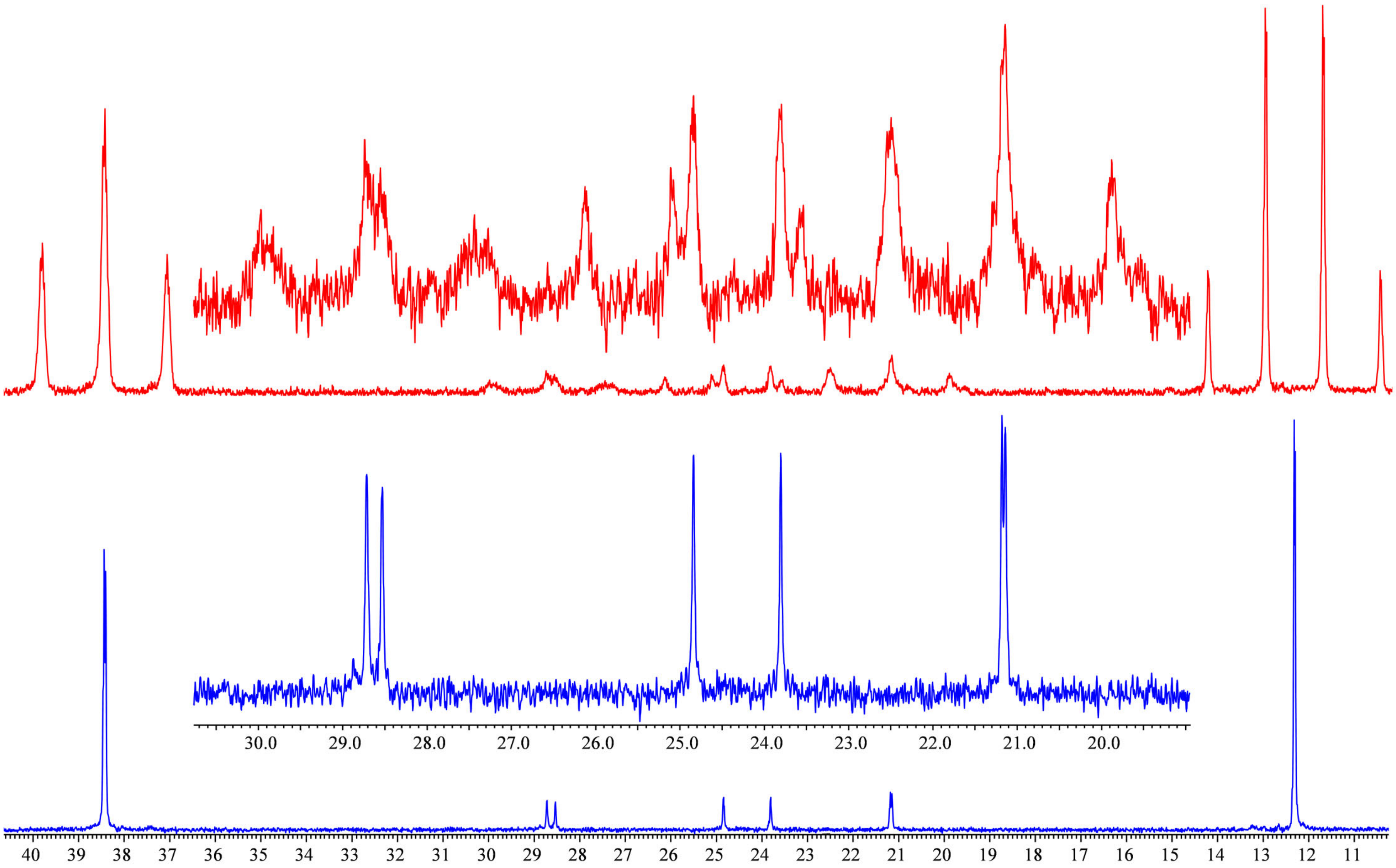

Figure 165S. ^{13}C and ^{13}C-{^{1}H} NMR spectra (100.6 MHz, $CDCl_3$) of $(Et_2N)_3P^+–C_6H_{12}–P^+(NEt_2)_3$ $2Br^–$ (**9**).

Table S1. Acute toxicity parameters $LD_{0\text{-}100}$ of compounds **4a**, **4b**, **4d**, **5a**, **5b**, **6b**.

Compound	LD_0, mg/kg	LD_{16}, mg/kg	LD_{84}, mg/kg	LD_{100}, mg/kg
**4a**	11.9	22.0	280.0	887.5
**4b**	11.0	16.4	68.0	95.0
**4d**	1.3	2.8	94.0	842.5
**5a**	12.3	24.4	184.0	872.5
**5b**	11.3	18.0	60.0	93.8
**6b**	15.4	44.4	240.0	375.0
